# SPOP-mediated K27-linked non-degradative ubiquitination of KCNN3 suppressing HCC progression via the CTCF-SATB1 axis

**DOI:** 10.1038/s41419-026-08765-3

**Published:** 2026-05-10

**Authors:** Ziqing Zhan, Yidong Ge, Jiaxin Shi, Hongze Liang, Yuxuan Li, Jiabei Jin, Gun Chen, Fengguang Zhai, Lili Kong, Yan Lin, Siyuan Wang, Litao Chen, Linlin Liu, Kuihao Chen, Pengrong Lou, Meng Ye, Xiaofeng Jin

**Affiliations:** 1 Department of Biochemistry and Molecular Biology, Health Science Center, Ningbo University, Ningbo, China; 2 Department of Radiotherapy and Chemotherapy, The First Hospital of Ningbo University, Ningbo, China; 3 Key Laboratory of Advanced Mass Spectrometry and Molecular Analysis of Zhejiang Province, School of Materials Science and Chemical Engineering, Ningbo University, Ningbo, China; 4 The Affiliated People’s Hospital of Ningbo University, Ningbo, China

**Keywords:** Oncogenes, Metastasis

## Abstract

The metastasis of hepatocellular carcinoma (HCC) cells remains a major obstacle to achieving favorable clinical outcomes, yet the underlying molecular mechanisms are still not fully understood. The dysregulation of ion channels is related to epithelial-mesenchymal transition (EMT) phenotype-related pathways, especially the aberrant function of K+ ion channels in HCC. In this study, we observed that the potassium-calcium-activated channel subfamily N member 3 (KCNN3/SK3/ KCa2.3) ion channels were significantly upregulated in HCC cells, promoting the migration and invasion of HCC in vitro and in vivo. Mechanistically, activation of the KCNN3 ion channel was found to enhance phosphorylation of the CCCTC-binding factor (CTCF), which in turn stimulates transcription of the EMT-related factor special AT-rich sequence-binding protein 1 (SATB1) via binding the “CCCTC” region within its promoter, thereby driving HCC cell migration and invasion. Furthermore, we identified that speckle-type POZ protein (SPOP), an E3 ligase adaptor, recognizes the SPOP-binding consensus (SBC) motif “ASSTT” (aa 250-254) in KCNN3 and mediates its ubiquitination via K27-linked ubiquitin chain. Notably, this type of ubiquitination does not induce KCNN3 turnover, but induced KCNN3 translocation from the cell membrane into the cytosol, thus suppressing KCNN3-mediated ion channel activity. Importantly, HCC-associated SPOP mutations or KCNN3-ΔSBC dramatically disrupt the SPOP–KCNN3 regulatory axis, accelerating HCC progression. These effects can be effectively counteracted by treatment with the KCNN3 channel inhibitor edelfosine and the calcium chelators BAPTA-AM, suggesting a promising therapeutic strategy for HCC patients.

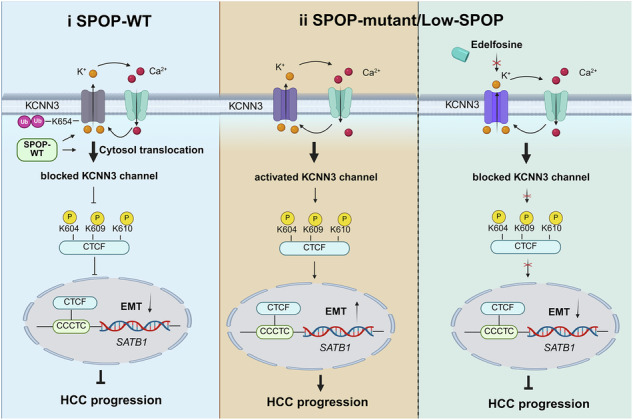

## Introduction

Liver cancer is the sixth most common malignancy worldwide and the third leading cause of cancer-related deaths, with metastasis being the leading cause of liver cancer treatment failure and patient death [1]. Hepatocellular carcinoma (HCC) cells exhibit a typical epithelial-mesenchymal transition (EMT) phenotype, which is closely related to the occurrence, development, and metastasis of tumors [2–4]. Emerging research has revealed the significant role of ion channels in cancer, and some molecules have been approved for cancer treatment targeting the components related to these ion channels. Notably, the dysregulation of ion channels is related to EMT-related pathways, among which K^+^ ion channels/Potassium channels (KCN) are also involved in the metastasis and progression of HCC [5–8]. The *KCNN3* gene encodes a potassium-calcium activated channel subfamily N member 3 (KCNN3/SK3/ KCa2.3), belonging to the *KCNN* family, which includes four genes: *KCNN1, KCNN2, KCNN3 and KCNN4*, corresponding to KCa2.1, KCa2.2, KCa2.3 and KCa3.1, respectively, and they combine to form homologous or heterotetramers [9]. KCNN3 has ion channel activity, which can bind calmodulin to form cell membrane tissue components, participating in ion transport (including K^+^ ions), synaptic transmission [10], development and growth of nerve cells [11], and calcium activation of potassium channel activity [12]. KCNN3 is mainly located in the cytoplasm and lipid rafts [13], and participates in the regulation of neuronal excitability, and its abnormality can lead to the occurrence of diseases such as atrial fibrillation and Zimmermann-Laband syndrome [14]. Hyperpolarization caused by KCNN3 promotes Ca^2+^ entry and regulates intracellular Ca^2+^ concentration, promoting the EMT in breast, bladder, colon, and prostate cancer cells [15–17]. In addition, low expression of KCNN3 may affect drug resistance in ovarian cancer [18]. However, the role of KCNN3 in HCC remains unclear, and related studies are quite limited.

Speckle-type BTB/POZ protein (SPOP) is a substrate-binding adaptor of the Cullin 3-RING E3 ubiquitin ligase complexes (CRL3) [19], which contains an N-terminal MATH domain, internal BTB domains, and a C-terminal nuclear localization signal (NLS). SPOP proteins are located in both the nucleus and cytoplasm [20]. The evolutionarily conserved MATH domain is responsible for substrate binding, and the vast majority of cancer-associated SPOP mutations are detected in this region. Almost all substrates of SPOP contain one or more typical or atypical SPOP-binding consensus (SBC; φ-π-S-S/T-S/T; φ-nonpolar, π-polar amino acid) motifs that are bound by the MATH domain. SPOP can mediate the ubiquitination and degradation of androgen receptor (AR), estrogen receptor (ER) or non-degradative polyubiquitination of INF2, MyD88, HIPK2, P62 and 53BP1 [19–25]. SPOP functions as a tumor suppressor in a variety of human malignancies, such as prostate, lung, colon, stomach, and liver cancers, while it plays a carcinogenic role in kidney cancer, suggesting that SPOP functions in a cancer-type-specific manner [26, 27], and other knowledge of SPOP in HCC is quite limited.

In our study, we found *KCNN3* was significantly up-regulated in HCC and was closely related to the clinical grades and stages of patients, promoting the migration and invasion of HCC. Mechanistically, we found that KCNN3-mediated ion channel activation can promote the phosphorylation of CTCF, thereby inducing the transcription of the EMT-related factor SATB1, enhancing the HCC progression in vitro and in vivo. Moreover, SPOP can recognize the KCNN3-250 “ASSTT” 254 motif and mediate non-degradative ubiquitination via K27-linked ubiquitin chain, inducing KCNN3 translocation from the cell membrane into the cytosol, thus suppressing KCNN3-mediated ion channel function. Notably, HCC-associated SPOP mutations or KCNN3-ΔSBC dramatically disrupt the SPOP-KCNN3 signaling axis, triggering the progression of HCC, and these effects can be effectively counteracted by treatment with the KCNN3 channel inhibitor edelfosine and the calcium chelators BAPTA-AM.

## Results

### KCNN3 promotes migration and invasion of HCC

**Fig. 1:**
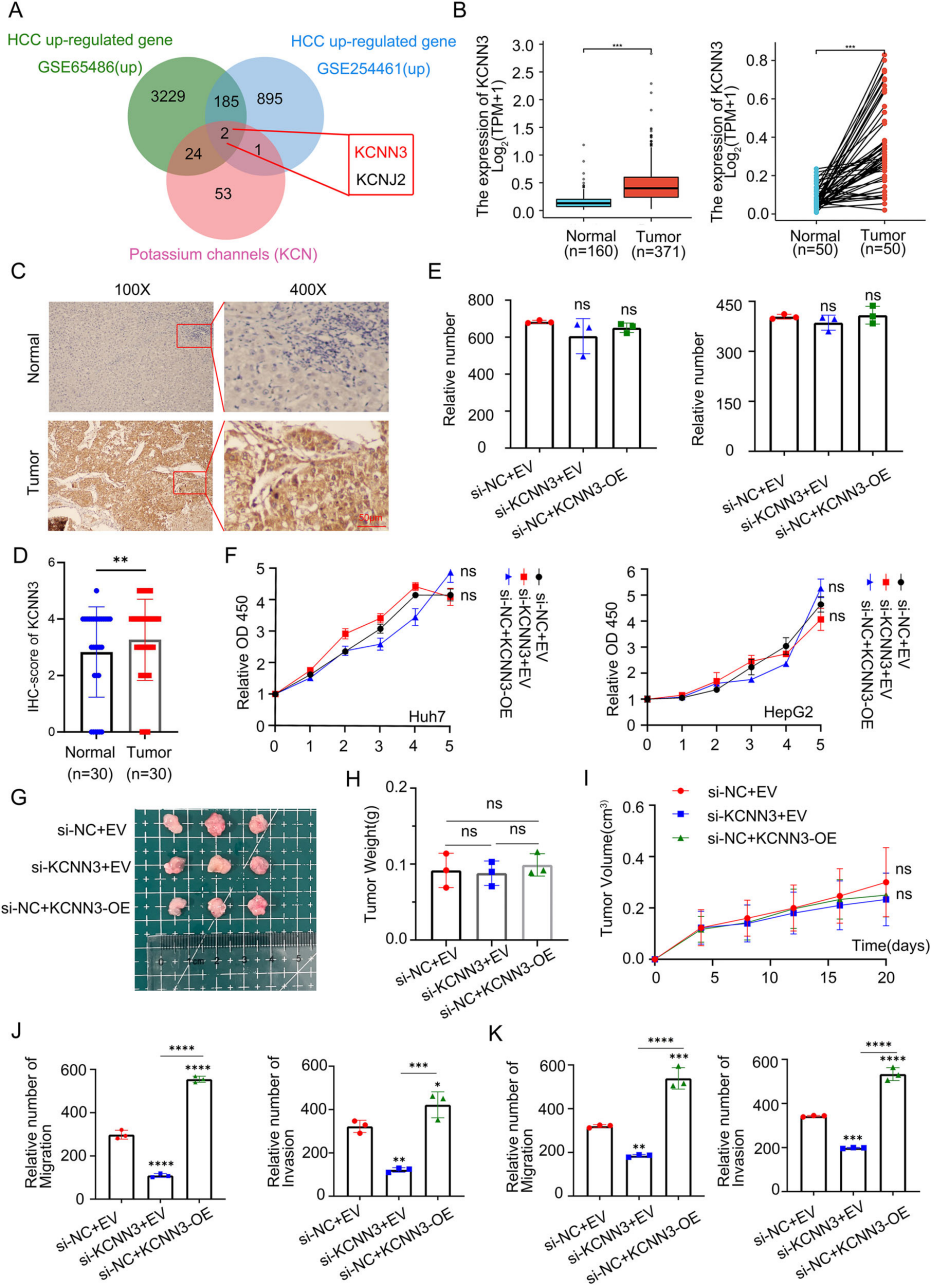
KCNN3 promotes migration and invasion of HCC, without proliferation. **A** Venn diagram illustrating the overlap between potassium channel genes, differentially expressed genes from the GSE65486 dataset (Tumor vs. Adjacent Normal), and the GSE254461 dataset (Hepatocellular Carcinoma vs. Normal Liver Tissue). This analysis identified two common upregulated genes, *KCNN3* and *KCNJ2*, with KCNN3 showing particularly significant upregulation. **B** In the TCGA database, the paired and unpaired analysis of KCNN3 at mRNA levels in HCC. **C** IHC showed representative images of KCNN3 expression levels in paraffin-embedded HCC and normal liver tissues. **D** Statistical graph of IHC in C (Normal, *n* = 30; Tumor, *n* = 30). Scale bar, 50 μm. The results were analyzed using a paired t-test. **E**, **F** Colony formation assays and CCK8 assays had shown that KCNN3 does not affect the proliferation of HCC in vitro. **G**–**I** Subcutaneous tumor formation in mice proved that KCNN3 did not affect the proliferation of HCC in vivo. **J** Statistical data of transwell (migration and invasion) assays after transfection of si-NC + EV, si-KCNN3 + EV and si-NC + KCNN3-OE in Huh7 cell lines. **K** Statistical data of transwell (migration and invasion) assays after transfection of si-NC + EV, si-KCNN3 + EV and si-NC + KCNN3-OE in HepG2 cell lines. The differences between the two groups were analyzed using Student’s *t* test, and multiple comparisons were performed using two-way analysis of variance (ANOVA). All data are shown as means ± SD (*n* = 3). *p < 0.05, **p < 0.01, ***p < 0.001.

Cell metastasis of HCC remains a major clinical challenge [28]. Previous studies have shown that cell metastasis is associated with EMT-related pathways as well as K^+^ ion channels dysregulation [5, 29, 30], but the underlying mechanism remains unclear. To determine this, we first investigated the online GEO database, GSE254461 (*P-*value < 0.01, Log_2_FC > 0) and GSE65486 (*P*-value < 0.01, LogFC>0) to obtain the upregulated gene set in HCC. Through an intersection analysis of potassium channel genes with publicly available HCC datasets (GSE254461 and GSE65486), we identified two consistently upregulated genes: KCNN3 (Log2FC = 2.96 in GSE254461; LogFC=0.21 in GSE65486) and KCNJ2. KCNN3 demonstrated the most pronounced upregulation among all overlapping genes (Fig. 1A). KCNN3 promotes the EMT of breast, bladder, colon, and prostate cancer cells [15–17], as well as drug resistance in ovarian cancer [18]; however, the underlying role in HCC remains largely unclear. First, we found that KCNN3 was upregulated in HCC and correlated with the clinical characteristics of HCC using the TCGA database analysis (Fig. 1B) (Supplementary Fig. 1A, B). Consistent with this, the results of immunohistochemistry (IHC) of 30 pairs of paraffin-embedded HCC and paired normal liver tissues showed that the expression of KCNN3 protein was upregulated and highly positively stained in a large percentage of HCC tissues compared with paired normal liver tissues (Fig. 1C, D). We then constructed stable HCC cell lines with KCNN3 overexpression or knockdown (Supplementary Fig. 1C, D), and found that KCNN3 did not affect HCC proliferation in vitro and in vivo (Fig. 1E–I). Notably, the overexpression of KCNN3 dramatically promoted the migration and invasion of HCC, and vice versa (Fig. 1J, K) (Supplementary Fig. 1H, I). Together, these findings indicate that KCNN3 promotes migration and invasion of HCC.

### Identification of KCNN3 as a novel SPOP interactor

**Fig. 2:**
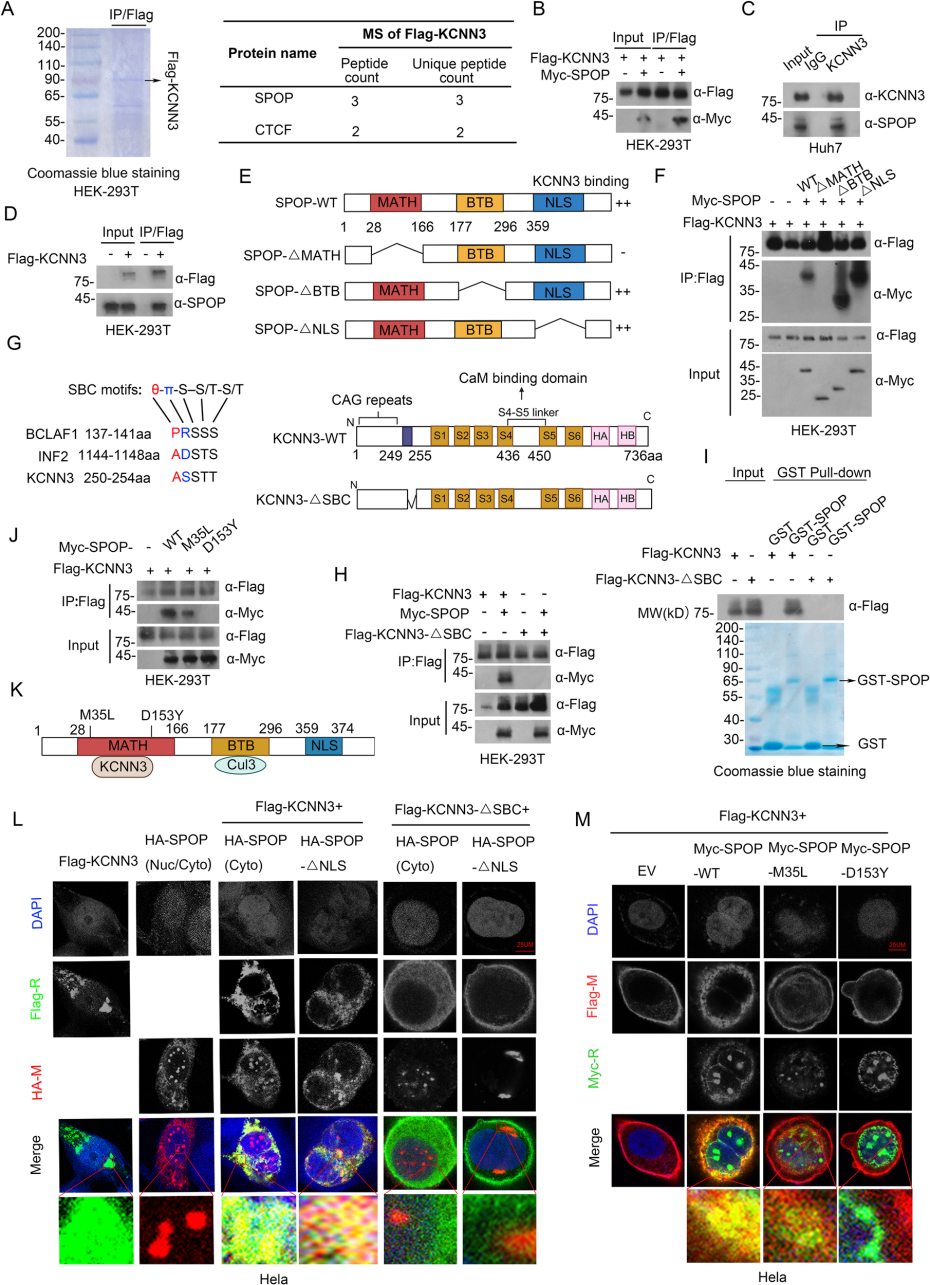
Identification of KCNN3 as a novel SPOP interactor. **A** Flag-KCNN3 plasmid was transfected into HEK-293T cells, and the Flag-KCNN3 protein complex was obtained through CO-IP of anti-Flag antibody and stained by Coomassie Blue staining (Left). The MS of Flag-KCNN3 showed the potential interacting proteins of KCNN3, including SPOP (Right). **B** Transfected with FLAG-KCNN3 and co-transfected with Myc-SPOP in HEK-293T cells, obtained whole cell lysates (WCLs) and CO-IP samples and tested by Western blotting. **C** WCLs and CO-IP samples of anti-KCNN3 antibody were obtained from Huh7 cells and tested by Western blotting (**D**) Transfection of Flag-KCNN3 into HEK-293T cells, obtained WCLs and CO-IP samples and tested by Western blotting. **E** Structure diagram of wild-type SPOP (SPOP-WT) and its domain truncates (SPOP-ΔMATH、SPOP-ΔBTB、SPOP-ΔNLS) (**F**) WCLs and CO-IP samples of anti-Flag antibody were obtained from HEK-293T cells transfected with Flag-KCNN3 and/or not co-transfected with Myc-SPOP-WT/ΔMATH/ΔBTB/ΔNLS and detected by western blotting. **G** Structure diagram of KCNN3 wild-type and SBC deletion (250“ASSTT“254). **H** WCLs and CO-IP samples of anti-Flag antibody were obtained from HEK-293T cells transfected with Flag-KCNN3 and/or Myc-SPOP and/or Flag-ΔSBC. **I** Bacterially expressed GST-SPOP or GST-bound glutathione-Sepharose beads were incubated with bacterially expressed Flag-KCNN3. Bound Flag-KCNN3 was detected by western blotting using an anti-Flag antibody. GST and GST-SPOP were detected by western blotting and Coomassie Blue staining. **J** WCLs and CO-IP samples of anti-Flag antibody were obtained from HEK-293T cells transfected with Flag-KCNN3 and/or co-transfected with Myc-SPOP-WT and HCC-associated SPOP mutants (Myc-SPOP-M35L/D153Y) and detected by western blotting. **K** Structural diagram of HCC-associated SPOP mutants (Myc-SPOP-M35L/D153Y). **L** Representative cellular immunofluorescence images of HeLa cells stained with α-FLAG-R, α-HA-M, and DAPI after transfection with the indicated plasmids. Scale bar, 25 μm. **M** Representative cellular immunofluorescence images of HeLa cells stained with α-FLAG-M, α-Myc-R, and DAPI after transfection with the indicated plasmids. Scale bar, 25 μm.

To explore the upstream regulating proteins of KCNN3, we obtained the KCNN3 protein complex by stable expression of Flag-KCNN3 in HEK-293T cells, and found a potential interaction between KCNN3 and SPOP by high-throughput affinity purification mass spectrometry (AP-MS) analysis (Fig. 2A). A previous study showed that SPOP functions as a tumor suppressor in HCC [26]. To verify that SPOP is a bona fide KCNN3-interacting protein, we co-expressed Flag-KCNN3 and Myc-SPOP plasmids in HEK-293T cells and then conducted co-immunoprecipitation (CO-IP) analysis with Anti-DYKDDDDK Affinity Beads, and found an interaction between these two exogenously expressed proteins (Fig. 2B). Similarly, we confirmed that KCNN3 interacts with SPOP at the endogenous level by CO-IP utilizing an anti-KCNN3 antibody in the cell lysate from Huh7 cells (Fig. 2C). Furthermore, the CO-IP experiment showed that Flag-KCNN3 could capture endogenous SPOP (Fig. 2D). To identify the domains in which SPOP interacts with KCNN3, we generated three deletion truncates of SPOP, including SPOP-ΔMATH, ΔBTB, and ΔNLS (Fig. 2E) [20]. CO-IP analysis showed that SPOP-WT, ΔBTB, and ΔNLS effectively interacted with KCNN3, but not the SPOP-ΔMATH (Fig. 2F), which was also confirmed by IF experiments (Supplementary Fig. 1H), suggesting that SPOP interacts with KCNN3 via the MATH domain. Moreover, we explored the domains of KCNN3 that interacted with SPOP. Previous studies have shown that there is one or more SPOP-binding consensus (SBC) motifs in known SPOP substrates, such as BCLAF1 and INF2 [21, 31]. We performed a protein motif search for the KCNN3 protein sequence and found only one perfectly matched SBC motif (250“ASSTT”254). To confirm whether this potential motif is needed for SPOP-KCNN3 interaction, we constructed the SBC domain deletion mutant plasmid Flag-KCNN3-ΔSBC (Fig. 2G), and indeed Flag-KCNN3-ΔSBC could not bind to SPOP compared to the Flag-KCNN3 by CO-IP, GST pull-down and IF, respectively (Fig. 2H, I–L), indicating that the interaction is dependent on this motif.

A previous study has shown that most SPOP mutations detected in HCC occur primarily in the MATH domain [32]; therefore, we constructed HCC-associated SPOP mutations, including M35L and D153Y (Fig. 2K), and the binding capacity of SPOP-M35L was weakened, while SPOP-D153Y was eliminated compared with SPOP-WT by CO-IP and IF assays (Fig. 2J/M).

### SPOP mediates K27-linked non-degradative ubiquitination of KCNN3 at the K654 site

**Fig. 3:**
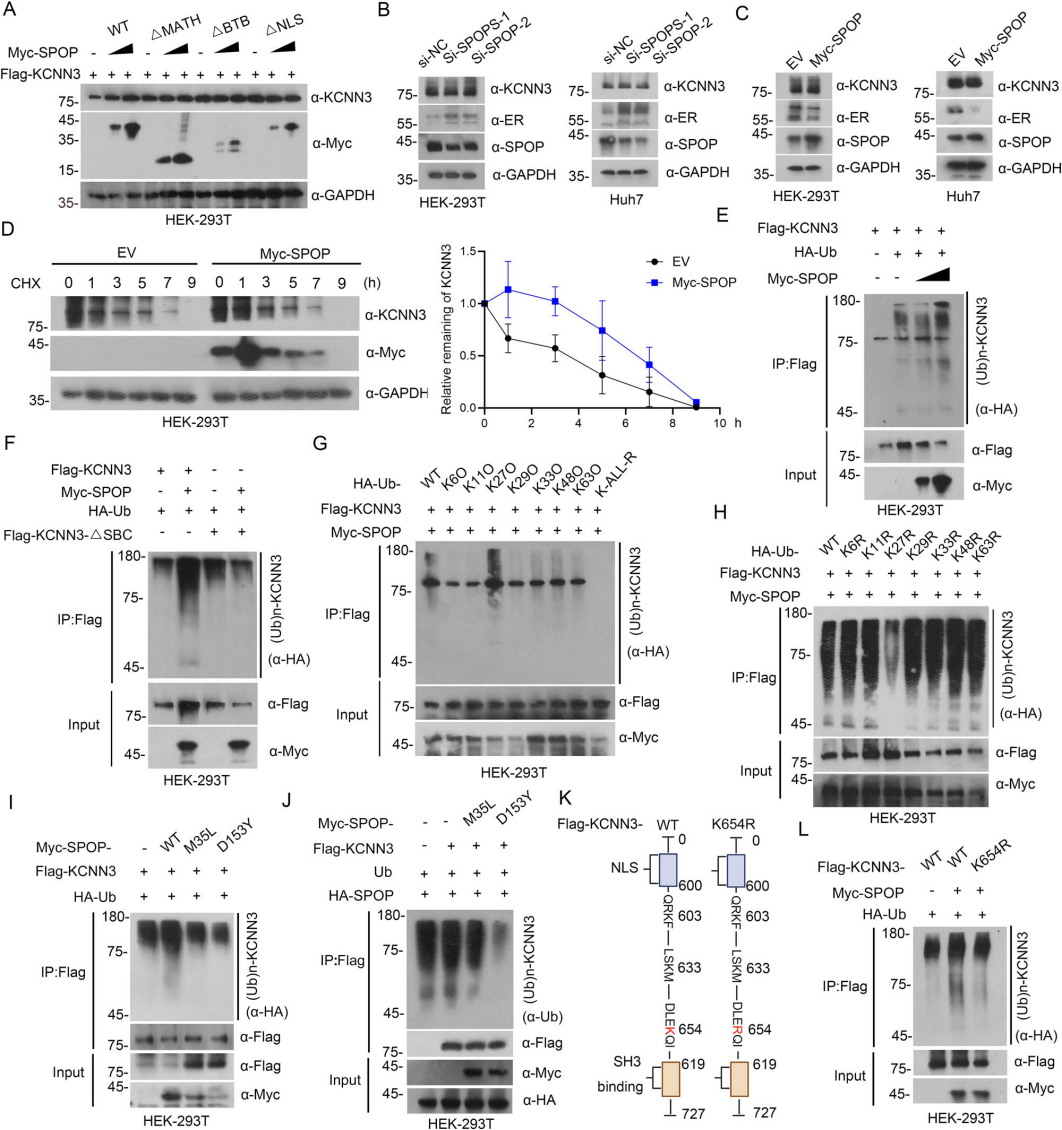
SPOP mediates K27 non-degradative ubiquitination of KCNN3 at K654 site. **A** HEK-293T cells were transfected with Flag-KCNN3 and/or without co-transfected with Myc-SPOP-WT/ΔMATH/ΔBTB/ΔNLS, obtained WCLs and detected by western blotting. **B** HEK-293T cells and Huh7 cells were transfected with si-SPOP-1/2, obtained WCLs and detected by Western blotting. **C** HEK-293T cells and Huh7 cells were or not transfected with Myc-SPOP, obtained WCLs and detected by western blotting. **D** HEK-293T cells were transfected with Flag-KCNN3 and/or co-transfected with Myc-SPOP and obtained WCLs at different time points after treatment with 50 μg/ml cycloheximide (CHX) and detected by western blotting. The quantification was normalized to GAPDH. Statistics of KCNN3 protein half-life (Right). **E** WCLs and CO-IP samples of anti-Flag antibody were obtained from HEK-293T cells transfected with Flag-KCNN3, Myc-SPOP and HA-Ub, and detected by western blotting. **F** WCLs and CO-IP samples of anti-Flag antibody were obtained from HEK-293T cells transfected with Flag-KCNN3, Myc-SPOP, HA-Ub, Flag-KCNN3-ΔSBC, and detected by western blotting. **G** WCLs and CO-IP samples of anti-Flag antibody were obtained from HEK-293T cells transfected with Flag-KCNN3, co-transfected with Myc-SPOP and HA-Ub mutants (K6O, K11O, K29O, K33O, K48O, K63O and K-all-R) and detected by western blotting. **H** WCLs and CO-IP samples of anti-Flag antibody were obtained from HEK-293T cells transfected with Flag-KCNN3, co-transfected with Myc-SPOP and HA-Ub mutants (K6R, K11R, K29R, K33R, K48R, and K63R), and detected by western blotting. **I** WCLs, and CO-IP samples of anti-Flag antibody were obtained from HEK-293T cells transfected with Flag-KCNN3, HA-Ub and/or co-transfected with Myc-SPOP-WT/M35L/D153Y, and detected by western blotting. **J** WCLs and CO-IP samples of anti-Flag antibody were obtained from HEK-293T cells transfected with Ub, HA-SPOP and/or co-transfected with Flag-KCNN3, Myc-SPOP-M35L/D153Y, and detected by western blotting. **K** Structural model diagram of mutant Flag-KCNN3-K654R (**L**) WCLs and CO-IP samples of anti-Flag antibody were obtained from HEK-293T cells transfected with HA-Ub and/or without co-transfected with Myc-SPOP and Flag-KCNN3-WT/K654R. The differences between the two groups were analyzed using Student’s *t* test, and multiple comparisons were performed using two-way analysis of variance (ANOVA). All data are shown as means ± SD (*n* = 3). *p < 0.05, **p < 0.01, ***p < 0.001.

Given that SPOP is an E3 ligase adaptor of the CUL3 family [20], we then explored whether SPOP could promote the ubiquitination and degradation of KCNN3. Surprisingly, the overexpression of SPOP or HCC-associated mutations did not downregulate KCNN3 protein levels (Fig. 3A). Consistent with these findings, *SPOP* knockdown by short hairpin RNA (si-SPOP) in HEK-293T cells and Huh7 cells increased the protein levels of known degradative substrates, such as ER, but not the KCNN3 (Fig. 3B), and vice versa (Fig. 3C). Consistent with this, SPOP did not affect KCNN3 turnover (Fig. 3D) (Supplementary Fig. 2D, E). However, SPOP could mediate polyubiquitination of KCNN3 dramatically in a dose-dependent manner (Fig. 3E) (Supplementary Fig. 2F), while failing in KCNN3-ΔSBC (Fig. 3F). Together, these results indicate that KCNN3 is a non-degradative substrate of SPOP. We then examined the SPOP-mediated ubiquitination linked chains of KCNN3, utilizing a set of ubiquitin mutants that contain a single K-R mutation in each of the seven lysines in the ubiquitin, as well as a lysine-free ubiquitin mutant(K-ALL-R) in which all lysines are replaced by arginine. Ub (K-ALL-R) mutants eliminated SPOP-mediated KCNN3 ubiquitination, ruling out the possibility that SPOP contributed to multiple monoubiquitination events in KCNN3 (Fig. 3G). The expression of Ub-K6R, K11R, K29R, K33R, K48R, and K63R did not dramatically change ubiquitination of KCNN3, suggesting that they are largely dispensable for SPOP-mediated KCNN3 ubiquitination. Notably, a modest reduction in KCNN3 ubiquitination was observed in Ub-K27R (Fig. 3H). Furthermore, we chose to use a series of reciprocal mutants that contain only one lysine, and the other six lysines were mutated to arginine, and found that K6O, K11O, K29O, K33O, K48O, and K63O Ub mutants significantly eliminated SPOP-mediated KCNN3 ubiquitination, whereas K27O retained the ubiquitination of KCNN3 (Fig. 3G). Together, these results indicate that SPOP-mediated non-degradative ubiquitination of KCNN3 occurs mainly via K27-linked chain. Compared to the SPOP-WT, HCC-associated SPOP mutations exhibited loss-of-function in mediating K27 ubiquitination of KCNN3 (Supplementary Fig. 1G) (Fig. 3I, J).

To further study the ubiquitination modificated sites of KCNN3, we enriched KCNN3 treated with SPOP-mediated ubiquitination for MS analysis, and found that K603/633/654 sites on KCNN3 might be the potential site (Supplementary Fig. 2A), and constructed the mutants, including KCNN3-K603A, K603/633 A, and K603/633/654 A (Supplementary Fig. 2C). Compared with the ubiquitination level of KCNN3-WT, the ubiquitination levels in KCNN3-K603A and K603/633 A remained almost the same, but that in KCNN3-K603/633/654 A was dramatically reduced (Supplementary Fig. 2G). Additionally, we constructed a KCNN3-K654R mutant (Fig. 3K), and indeed, the ubiquitination is completely abolished (Fig. 3L). Notably, this site was also confirmed by the database (https://www.phosphosite.org/proteinAction.action?id=19367&showAllSites=true) (Supplementary Fig. 2B). Altogether, these results indicate that SPOP can mediate K27-linked ubiquitination of KCNN3 at the K654 site, in a non-degradative manner.

### SPOP-mediated K27-linked ubiquitination of KCNN3 reduces its membrane localization, suppressing its role in ion channel activity

**Fig. 4:**
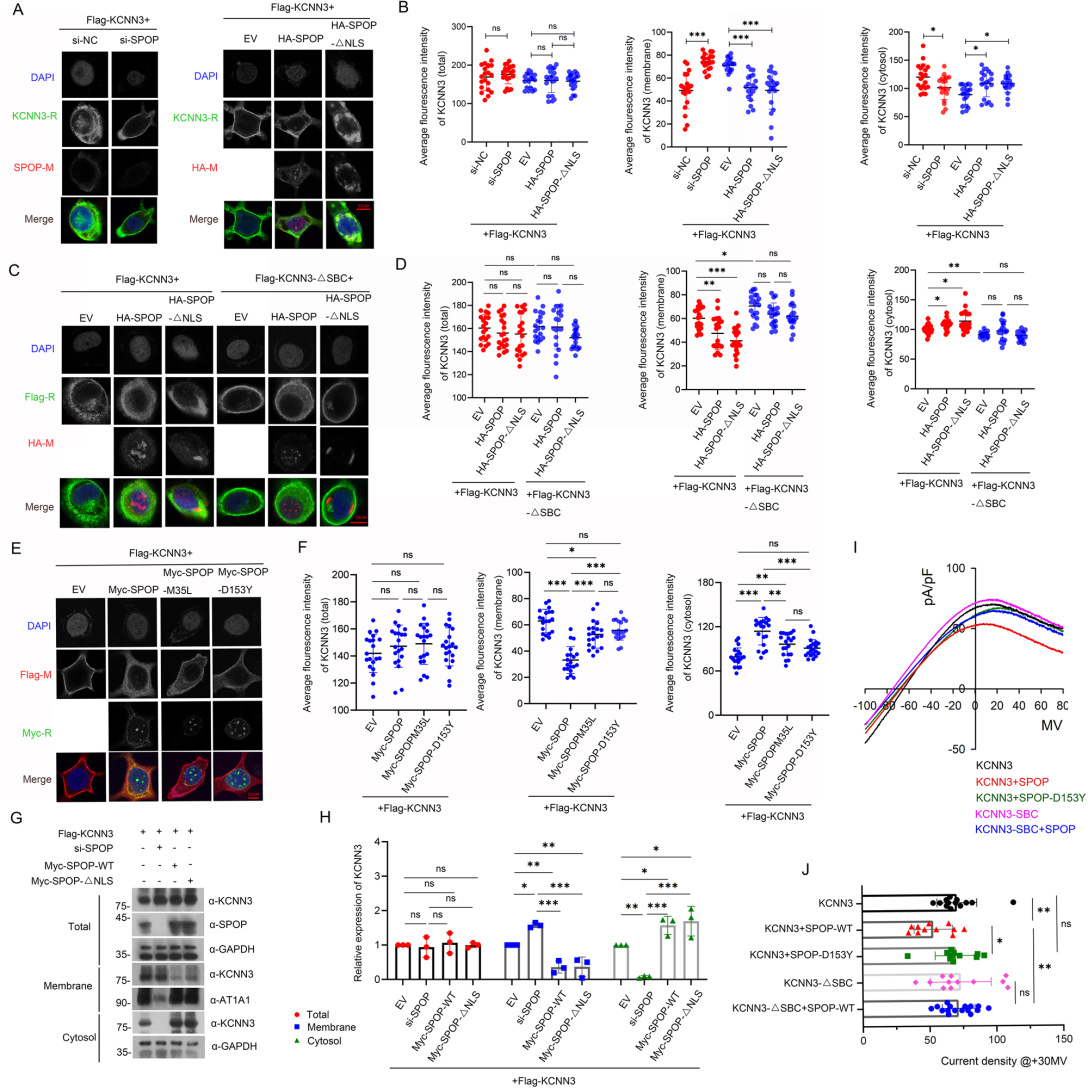
SPOP-mediated K27 ubiquitination of KCNN3 reduces its membrane localization, suppressing its role as an ion channel. **A** Representative cellular immunofluorescence images of Huh7 cells stained with α-KCNN3-R, α-SPOP-M, α-HA-M and DAPI after transfection of the indicated plasmids. Scale bar, 25 μm (**B**, **D**, **F**). The statistics of 20 cells in each group, and the total/membrane/cytosol fluorescence intensity of KCNN3 were analyzed. **C** Representative cellular immunofluorescence images of Huh7 cells stained with α-Flag-R, α-HA-M and DAPI after transfection with the indicated plasmids. Scale bar, 25 μm. **E** Representative cellular immunofluorescence images of Huh7 cells stained with α-Myc-R, α-Flag-M, and DAPI after transfection with the indicated plasmids. Scale bar, 25 μm. **G** HEK293T cells transfected with Flag-KCNN3, si-SPOP, and Myc-SPOP-WT/ΔNLS were treated with the Membrane and Cytosol Protein Extraction Kit. Please refer to the instructions provided for this procedure. Western blotting was performed on the extracted membrane, and cytoplasmic proteins, **H** Image J was used for quantitative analysis of protein bands in plasma membrane isolation assays three times. **I** Patch-clamp assays were performed in HEK-293T cells transfected with the indicated plasmids. **J** Statistical result of patch clamp assays in each group. The differences between the two groups were analyzed using Student’s *t* test, and multiple comparisons were performed using two-way analysis of variance (ANOVA). All data are shown as means ± SD (*n* = 3). *p < 0.05, **p < 0.01, ***p < 0.001.

KCNN3 is mainly located on lipid rafts of cell membranes for ion shuttling [33, 34], and our previous study showed that non-degradative ubiquitination changes the location of substrates [21]. To detect this, an IF assay was utilized to analyze the change in the membrane localization of KCNN3. According to the statistics of 20 cells in each group, we found that the total fluorescence intensity of KCNN3 did not change whether SPOP was knocked down or overexpressed, which was consistent with the fact that SPOP does not mediate the degradation of KCNN3. Notably, the fluorescence intensity of KCNN3 decreased mainly on the cell membrane, but increased in the cytosol in the SPOP-OE (HA-SPOP and HA-SPOP-ΔNLS) group. This phenotype was more significant in the SPOP-ΔNLS group, but not in the SPOP-KD (si-SPOP) group (Fig. 4A, B). Consistent results were found in the plasma membrane isolation assay; SPOP knockdown and overexpression of SPOP-WT and SPOP-ΔNLS did not change the total protein expression of KCNN3, but the protein expression of KCNN3 in the membrane was increased, while the protein expression in the cytosol was decreased in the si-SPOP group, and vice versa in the SPOP-WT and SPOP-ΔNLS groups (Fig. 4G, H). The experiments were repeated three times (Fig. 4G) (Supplementary Fig. 2H, I). Therefore, we concluded that the SPOP-mediated ubiquitination of KCNN3 dramatically reduces its membrane localization. Notably, since KCNN3-ΔSBC lost the localization with SPOP, SPOP did not reduce the membrane localization of KCNN3-ΔSBC compared to KCNN3 wild-type (Fig. 4C, D). Similarly, SPOP-D153Y did not reduce the membrane localization of KCNN3 compared to the SPOP wild-type (Fig. 4E, F). Additionally, we investigated whether the location of KCNN3 influenced its role as an ion channel. Utilizing patch-clamp assays, we found that SPOP can reduce KCNN3-mediated membrane potential, but not in the KCNN3-ΔSBC group or in the HCC-derived SPOP-D153Y mutation group (Fig. 4I, J). Together, we conclude that SPOP-mediated ubiquitination of KCNN3 dramatically reduced its membrane localization, resulting in the inhibition of ion channel function.

### KCNN3 promotes HCC EMT by inducing *SATB1* transcription via phosphorylation of CTCF

**Fig. 5:**
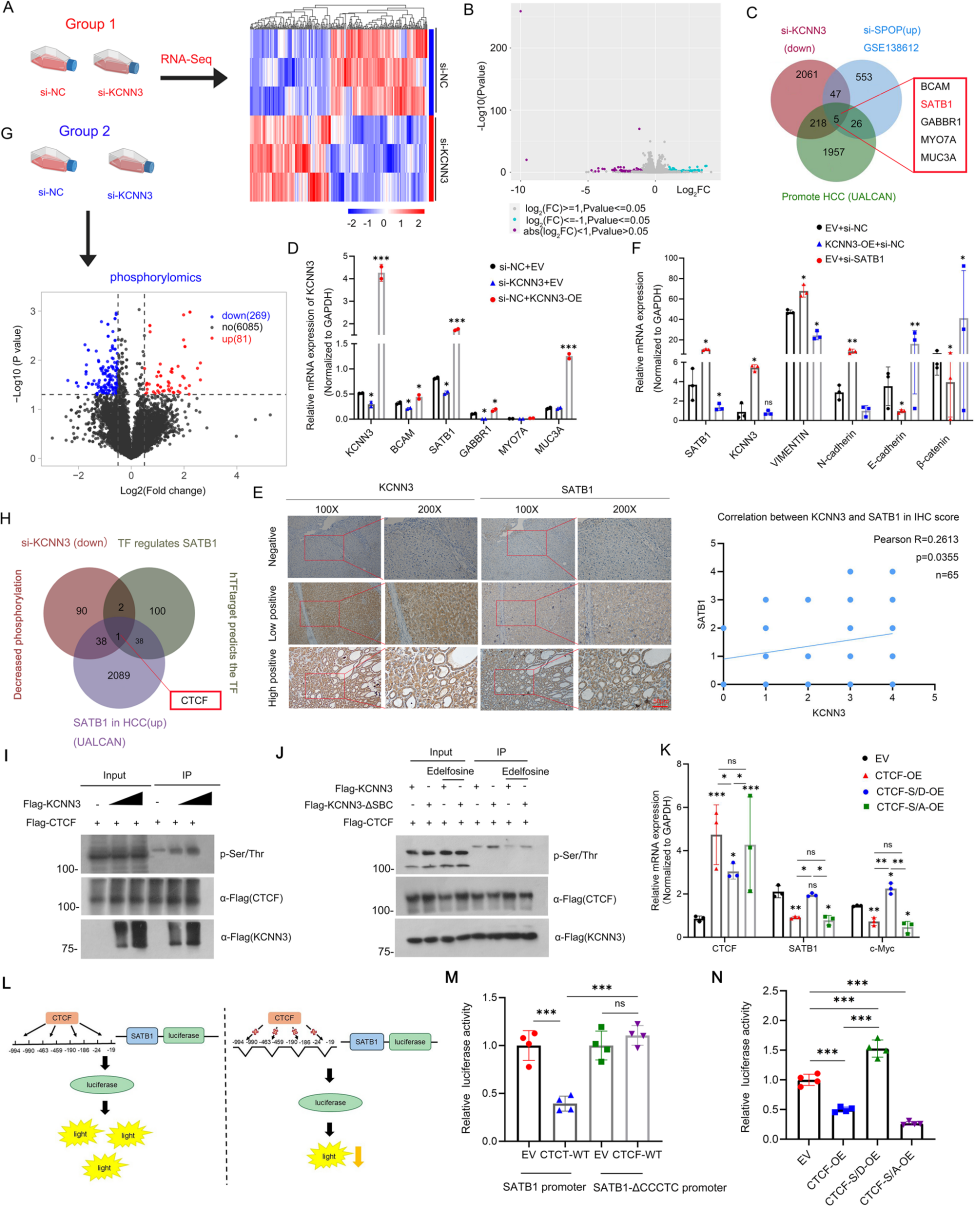
KCNN3 affects the transcription of *SATB1* by influencing the phosphorylation of CTCF, which promotes HCC EMT. **A** Heat maps of RNA-seq showed differential gene expression after KCNN3 knockdown in Huh7 cells. **B** Volcanic maps show differential genes after knocking down KCNN3 in Huh7 cells. **C** Venn diagram shows the intersection of down-regulated genes after KCNN3 knockdown, up-regulated genes after SPOP knockdown and medium-high expression genes of HCC in UALCAN (https://ualcan.path.uab.edu/analysis.html), overlapping with the five common genes *(BCAM, SATB1, GABBR1, MYO7A* and *MUC3A)* (**D**) Detected the mRNA level of the five common genes in Huh7 cells by RT-qPCR. **E** IHC demonstrated representative images of the different expression levels of KCNN3 and SATB1 in paraffin-embedded HCC tissues. Scale bar, 200 μm (Left). Correlation analysis between expression of KCNN3 and SATB1 in HCC (*n* = 65) (Right). **F** Detected the correlation between *SATB1* and EMT markers at mRNA levels in Huh7 cells by RT-qPCR. **G** Volcano map of phosphorylomics showed differential phosphorylated protein expression after KCNN3 knockdown in Huh7 cells. **H** Venn diagram shows the intersection of down-regulated phosphorylated protein after KCNN3 knockdown, the potential transcription factors regulating *SATB1* in the hTFtarget (wchscu.cn) database, Genes positively related to *SATB1* in HCC in TCGA database (The Cancer Genome Atlas Program (TCGA) - NCI), overlapping to the common transcription factor CTCF. **I** WCLs and CO-IP samples of anti-Flag antibody were obtained from HEK-293T transfected with Flag-KCNN3, Flag-CTCF, and detected by Ser/Thr phosphorylated and α-Flag antibodies by western blotting. **J** WCLs and CO-IP samples of anti-Flag antibody were obtained from HEK-293T transfected with Flag-KCNN3, Flag-CTCF, Flag-KCNN3-ΔSBC and/or without dealing with Edelfosine and detected by Ser/Thr phosphorylated and α-Flag antibodies by western blotting. **K** Detected the effects of CTCF-WT and its mutants (CTCF-S/A-mut and CTCF-S/D-mut) on SATB1 and c-Myc transcription in Huh7 cells by RT-qPCR. **L** SATB1-Lucference model of CTCF protein transcriptional regulation. **M** HEK-293T cells were transfected with the SATB1-Lucference or SATB1-Lucference mutant and/or without co-transfected with Flag-CTCF plasmids. After 24 h, the luciferase activities were measured by luminometer **N** HEK-293T cells were transfected with the SATB1-Lucference reporter and indicated plasmids. After 24 h, the luciferase activities were measured by a luminometer. The differences between the two groups were analyzed using Student’s *t* test, and multiple comparisons were performed using two-way analysis of variance (ANOVA). All data are shown as means ± SD (*n* = 3). *p < 0.05, **p < 0.01, ***p < 0.001.

To investigate the potential targets affected by KCNN3, we identified differentially expressed genes in *KCNN3*-knockdown HCC cells using RNA-Seq (Fig. 5A, B). To further explore the relationship between these genes and SPOP in HCC, we analyzed the up-regulated genes after SPOP knockdown and HCC-promoting differential genes from the NCBI database (https://www.ncbi.nlm.nih.gov/guide/dna-rna/) and TCGA (https://www.cancer.gov/ccg/research/genome-sequencing/tcga) databases. In conjunction with our previous RNA-Seq results, we tried to integrate the three datasets to determine if there were any overlapping genes and pathways that could serve as promising targets, and identified five potential targeting genes, including *SATB1, BCAM, GABBR1, MYO7A* and *MUC3A* (Fig. 5C). To further verify these results in Huh7 cells after *KCNN3* knockdown or overexpression by RT-qPCR, *SATB1* was found to be more significantly regulated by *KCNN3* (Fig. 5D). In addition, gene correlation analysis also confirmed that *SATB1* was positively correlated with *KCNN3* by the analysis of the TCGA database (Supplementary Fig. 3A). To validate the results of the TCGA analysis, we used IHC to evaluate the protein expression of KCNN3 and SATB1 in paraffin-embedded HCC tissues (65 pairs of specimens). The results showed that the protein expression of KCNN3 and SATB1 in HCC was positively correlated (Pearson’s R = 0.2613, *p* = 0.0355) (Fig. 5E). Moreover, we performed the Kyoto Encyclopedia of Genes and Genomes (KEGG) pathway analysis and found that *SATB1* was closely correlated with ion channels (Supplementary Fig. 3B–F), which is similar with *KCNN3* (Supplementary Fig. 3G, H). Consistent with a previous study [35], we also confirmed by RT-qPCR that *SATB1* increased *VIMENTIN* and *N-cadherin, which* positively correlated with EMT, while decreasing *E-cadherin* or *β-catenin* in HCC (Fig. 5F). By mining the Gene Set Enrichment Analysis (GSEA) enrichment pathway, we found that *KCNN3* and *SATB1* were also enriched into the Wnt pathway (Supplementary Fig. 3I, J) [36].

Given that EMT-related transcription factors can be triggered by phosphorylation [37], previous studies have shown that ion channels can affect signal transduction by modulating the phosphorylation homeostasis [38–40]. Therefore, we conducted phosphorylation proteomic analysis of *KCNN3*-knockdown HCC cells to identify targeting proteins with phosphorylation changes, and found 81 up-regulated phosphorylated proteins and 269 down-regulated phosphorylated proteins (Fig. 5G). We overlapped 269 down-regulated phosphorylated proteins, together with hTFtarget (wchscu.cn) database, analyzing the potential transcription factors of *SATB1*, as well as the TCGA database showing a positive relationship with *SATB1* in HCC, and found the potential multivalent transcriptional suppressor CTCF (Fig. 5H). CTCF, a CCCTC-binding factor, is a known transcriptional suppressor of c-Myc, but its inhibitory effect is reduced after phosphorylation [41, 42], possibly because of the fact that phosphorylation of CTCF reduces its DNA-binding activity [43]. Indeed, we confirmed that the phosphorylation of CTCF was positively regulated by KCNN3 (Fig. 5I), which is regulated by the activity of the KCNN3 channel, independent of its expression level (Fig. 5J). Similarly, to determine whether CTCF phosphorylation and SATB1 transcription are regulated by Ca^2+^, we employed the calcium chelator (BAPTA-AM). ChIP-qPCR assays subsequently revealed that calcium chelation alters both CTCF phosphorylation and SATB1 transcription in a manner consistent with the regulatory pattern of KCNN3(Fig. S3N, O). Phosphorylation proteomic results indicated that Ser 604/609/610/612 in CTCF are the promising phosphorylation sites that may be regulated by KCNN3-mediated ion channels (Supplementary Fig. 3K, L). We constructed the corresponding CTCF mutants, including the phosphorylation-dead mutation (CTCF-S/A-mut), or the phosphorylation-mimic mutation (CTCF-S/D-mut) (Supplementary Fig. 3M). Compared to CTCF-WT, CTCF-S/A-mut further suppressed the transcription of *SATB1*, while CTCF-S/D-mut promoted this effect (Fig. 5K), with the oncogene *c-Myc*, a targeting gene of CTCF, acting as a positive control here [44]. To directly validate CTCF binding to the predicted CCCTC region in the SATB1 promoter, we performed a dual-luciferase reporter assay. We cloned the 2000-bp region upstream of the SATB1 transcription start site into the pGL2-basic vector. And four binding sites containing the core CCCTC regions were identified in the promoter of the SATB1 from NCBI, located within the regions –994 to –990, –463 to –459, –190 to –186, and –24 to –19, respectively. Therefore, we generated a corresponding construct with the four CCCTC regions deleted (SATB1-ΔCCCTC) (Fig. 5L). The ectopic expression of CTCF significantly enhanced the activity of the wild-type SATB1 promoter. In contrast, this transactivation was completely abolished when the CCCTC regions were deleted. These results demonstrate that CTCF exerts its transcriptional regulation on SATB1 by specifically binding to this functional CCCTC region within its promoter (Fig. 5M). To further investigate whether CTCF phosphorylation affects its binding, we performed a dual-luciferase reporter assay using CTCF phosphodeficient (S/A) and phosphomimetic (S/D) mutants. The phosphomimetic mutant (S/D) enhanced the activity of the wild-type SATB1 promoter compared to CTCF-WT, whereas the phosphodeficient mutant (S/A) had an inhibitory effect (Fig. 5N). These results suggest that CTCF phosphorylation promotes SATB1 transcription, which is consistent with our RT-qPCR data.

### HCC-associated SPOP mutants “abrogate” the inhibitory effect of SPOP-WT on KCNN3-induced progression in HCC via the CTCF-SATB1 axis

**Fig. 6:**
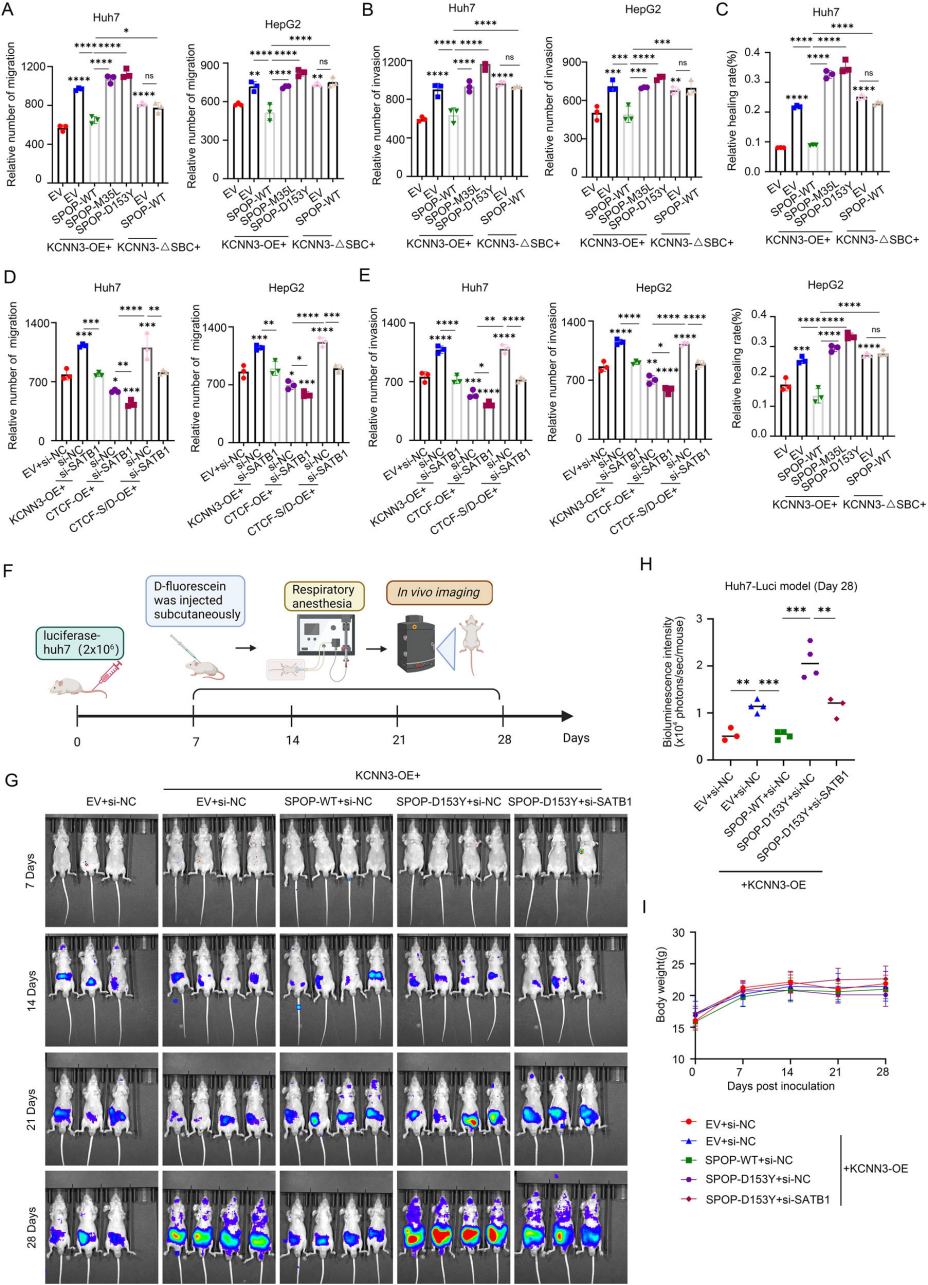
HCC-associated SPOP mutants “abrogate” the inhibitory effect of SPOP-WT on KCNN3-induced progression in HCC via the CTCF-SATB1 axis. **A** Statistical data of migration assays after transfection of indicated plasmids in Huh7(Left) and HepG2(Right) cell lines. **B** Statistical data of invasion assays after transfection of indicated plasmids in Huh7(Left) and HepG2(Right) cell lines. **C** Statistical data of migration assays after transfection of indicated plasmids in Huh7(Top) and HepG2(Bottom) cell lines. **D** Statistical data of invasion assays after transfection of indicated plasmids in Huh7(Left) and HepG2(Right) cell lines. **E** Statistical data of wound healing assays after transfection of indicated plasmids in Huh7(Left) and HepG2(Right) cell lines. **F** The flow chart of mice in vivo imaging. **G** Representative in vivo images of mice at 7, 14, 21 and 28 days after injection of luciferase-Huh7 cells transfected with the indicated plasmids. **H** The statistical data of bioluminescence intensity measured by Living Image and GraphPad Prism. **I** The body weights of the mice were monitored throughout the experiment. All data are shown as means ± SD (*n* = 3). *p < 0.05, **p < 0.01, ***p < 0.001. The differences between the two groups were analyzed using Student’s *t* test, and multiple comparisons were performed using two-way analysis of variance (ANOVA). All data are shown as means ± SD (*n* = 3). *p < 0.05, **p < 0.01, ***p < 0.001.

To verify the effect of SPOP on KCNN3-induced EMT of HCC, we found that SPOP-WT inhibited KCNN3-induced HCC progression, which was eliminated in the KCNN3-ΔSBC and SPOP-M35L/D153Y groups in vitro (Fig. 6A–C) (Supplementary Fig. 4A). Consistently, we established the HCC xenograft model (Huh7-Luci model) by inoculating BALB/c nude mice with luciferase-labeled Huh7 cells(luciferase-Huh7) of different groups (denoted as Day 0) (Fig. 6F). Bioluminescence imaging revealed that the bioluminescence signals of mice in the KCNN3-OE and SPOP-D153Y groups were enhanced over time compared to those in the control group (EV+si-NC), particularly on day 28 (Fig. 6G, H). In addition, neither obvious weight loss nor other adverse events were observed throughout the process (Fig. 6I). Therefore, we suggest that KCNNE-OE promotes metastasis of HCC in vivo, which can be significantly inhibited by SPOP-WT, but not SPOP-D153Y, and the progressive effect of the SPOP-D153Y group could be dramatically counteracted by SATB1 knockdown.

We further explored to verify the role of the KCNN3/CTCF/SATB1 signaling axis in the EMT of HCC. On the one hand, in order to investigate whether KCNN3-induced EMT promotion is dependent on SATB1, we verified that the KCNN3-induced EMT promotion can be dramatically suppressed by the knockdown of SATB1. Western blotting confirmed the efficiency of the two independent si-SATB1-1/2 (Supplementary Fig. 4B), we used si-SATB1with the good effect for the knockdown of SATB1 in the subsequent experiments. CTCF-WT inhibited EMT in HCC, and further inhibited EMT via SATB1 knockdown (si-SATB1). However, CTCF-S/D-mut promoted EMT in HCC, which was consistent with the KCNN3-OE group (Fig. 6D, E) (Supplementary Fig. 4C). On the other hand, in order to confirm whether the effect of CTCF phosphorylation on HCC EMT is dependent on KCNN3, we selected Edelfosine, a specific inhibitor of KCNN3 [45]. Firstly, we detected the median effect concentration (EC_50_) of Edelfosine is 14.25 μM in Huh7 and 12.29 μM in HepG2 (Supplementary Fig. 5A, B). Intriguingly, Edelfosine efficiently inhibited KCNN3-induced EMT, and the suppressive effect of CTCF-WT on EMT was further inhibited by Edelfosine, while the promoting effect of CTCF-S/D-mut on EMT could not be suppressed by Edelfosine (Supplementary Fig. 5C–E). Together, we identified that KCNN3-induced EMT in HCC is partly dependent on the CTCF-SATB1 signaling axis, and KCNN3-mediated phosphorylation of CTCF triggered the transcription of SATB1.

## Dicussion

Cell metastasis of HCC largely limits the clinical prognosis. We used RNA-Seq combined with the GEO online database analysis and found that the KCNN3 channel was significantly increased in HCC. Moreover, the expression of KCNN3 in HCC was higher than that in para-cancer tissues, and in vitro and *vivo* functional experiments showed that KCNN3 promoted the migration and invasion of HCC. To investigate the underlying mechanism of KCNN3 in HCC, we identified the interaction between the E3 ubiquitin ligase connector SPOP protein and KCNN3 through mass spectrometry analysis, CO-IP, GST pull-down and IF experiments. Furthermore, we found that SPOP mediated K27-linkage ubiquitination of KCNN3 without affecting its protein levels. Previous studies by our group and others have shown that non-degradative ubiquitination modifications may alter protein activity, subcellular localization, and protein-protein interactions, etc, playing a key role in a variety of cellular processes, including cell signaling, DNA damage repair, cell cycle regulation, and immune responses [46, 47]. Consistent with this, we found that SPOP-mediated K27-linkage ubiquitination of KCNN3 alters the membrane localization of KCNN3, as well as its ion channel function on the cell membrane, which was confirmed by IF and patch-clamp experiments. Studies have shown that ion channels affect downstream signaling and phosphorylation [38, 48–50]. To determine the downstream signal affected by KCNN3, we utilized RNA-seq and phosphorylated proteomic analysis in KCNN3-silenced HCC cells, and found that the decreased KCNN3 on the cell membrane reduced the phosphorylation of CTCF and transcription of *SATB1,* thereby inhibiting the occurrence of EMT. Besides, we further explored the potential kinase responsible for the phenotypic changes induced by KCNN3 knockdown. Our analysis revealed 23 downregulated phosphorylation-related kinases, among which two emerged as potential regulators of CTCF: MAPK3 (ERK1) and PRKD1 (PKD1). Regarding ERK1, it preferentially recognizes the (S/T)-P motif. CTCF, as a nuclear DNA-binding protein, contains multiple S/T-P sequences in its unstructured regions, providing a plausible “motif basis” for ERK-mediated phosphorylation. Moreover, upon activation, ERK rapidly translocates into the nucleus and modulates transcription factors and chromatin-associated proteins—consistent with the nuclear localization of CTCF [51]. As for PRKD1, the canonical PKD family consensus motif is L‑R‑x‑x‑S/T. CTCF also contains several such sequences in its linker regions. PKD1 is known to enter the nucleus and phosphorylate nuclear targets such as HDAC5 (at Ser259/Ser498), leading to 14-3-3 binding, nuclear export, and altered transcriptional output—supporting its role as a relevant kinase in nuclear protein and chromatin regulation [52]. While these bioinformatic and mechanistic insights suggest plausible pathways, further experimental validation will be necessary to identify the specific kinase(s) functionally coupling KCNN3 activity to CTCF phosphorylation.

In addition, studies have shown that promoter methylation is a key mechanism regulating gene activation, as supported by existing literature [53]. Our analysis of si-KCNN3 RNA-seq data revealed that KCNN3 is significantly enriched in the methionine metabolism pathway (hsa00270), which is involved in SAM synthesis (Fig. S4D). This finding provides a plausible mechanistic link through which KCNN3 may influence SATB1 gene activation.

HCC-derived SPOP mutants M35L and D153Y decreased the interaction with KCNN3, thus resulting in an increase in KCNN3 on the cell membrane, promoting HCC EMT. Notably, we found that the migration and invasion of HCC cells can be counteracted by KCNN3 knockdown or its inhibitor, Edelfosine. While our previous work established that SPOP-mediated degradation of PD-L1 is critical for immune surveillance in HCC [31], and independent studies have revealed its tumor-suppressive role in cholesterol metabolism [54], the present study uncovers a distinct mechanism. Here, we demonstrate that SPOP also exerts a tumor-suppressive function via the KCNN3-CTCF-SATB1 axis. Notably, this function is mediated not by its canonical role in promoting degradation, but through a novel mechanism involving non-degradative ubiquitination that modulates KCNN3 activity. These findings significantly broaden the functional repertoire of SPOP in tumor suppression. However, our research mainly focuses on the mechanism by which KCNN3 is activated as an ion channel on the cell membrane in HCC. The mechanism of elevated KCNN3 in HCC still needs further study.

Oxaliplatin is a first-line chemotherapeutic agent widely used in transcatheter arterial chemoembolization. It is one of the most commonly used chemotherapeutic agents for transcatheter arterial chemoembolization, hepatic arterial infusion, and the systemic treatment of HCC. However, its clinical efficacy is substantially limited by both intrinsic and acquired resistance[28]. To investigate this resistance, we established oxaliplatin-resistant Huh7 and HepG2 cell lines (OXA-R-Huh7 and OXA-R-HepG2). The half-maximal inhibitory concentration (IC50) of oxaliplatin was 10.47 μM in parental Huh7 and 5.976 μM in HepG2 cells, which increased to 60.28 μM and 29.33 μM in the corresponding resistant lines, respectively (Supplementary Fig. 6A–C). We identified KCNN3 as a factor associated with oxaliplatin resistance and further confirmed its role in promoting resistance (Supplementary Fig. 6D, E). Knockdown of KCNN3 attenuated the migratory and invasive capabilities of oxaliplatin-resistant HCC cells (Supplementary Fig. 6F–I). Consistent with KCNN3 upregulation in oxaliplatin-resistant HCC, we observed that wild-type SPOP suppresses KCNN3-driven migration and invasion in these cells, but not the HCC-associated SPOP-D153Y mutation, and treatment with Edelfosine could counteract these malignant phenotypes (Supplementary Fig. 6J–L). Moreover, baseline KCNN3 expression was elevated in oxaliplatin-resistant Huh7 and HepG2 cells relative to their parental counterparts (Supplementary Fig. 6M). Nevertheless, the precise mechanisms underlying KCNN3-mediated oxaliplatin resistance and its upregulation in resistant HCC remain to be elucidated.

Moreover, a previous study has found that KCNN3 promotes migration and invasion of breast, colon, and other tumors, partly by causing cellular Ca^2+^ influx. For example, cAMP-PKA inhibits KCNN3 channels by reducing Ca^2+^ entry and cancer cell migration by regulating the KCNN3-Orai1 complex [13]. SigmaR1 chaperones drive the migration of breast and colorectal cancer cells by regulating KCNN3-dependent Ca^2+^ homeostasis [15]. Ca^2+^ homeostasis in tumor cells is closely related to EMT, but its underlying mechanism remains unclear [55]. Our study revealed that altered membrane localization of KCNN3 activates some downstream signal transduction, such as phosphorylation of CTCF, thereby affecting *SATB1* transcription and ultimately leading to EMT. Given that the phosphorylation may lead to changes in nuclear localization [56]. Consistent with the previous study [44], we found that the fluorescence intensity of CTCF in the nucleus did not change in the si-KCNN3 groups (Supplementary Fig. 5F), and the fluorescence intensity of CTCF-S/D-mut did not change compared to the CTCF-WT groups (Supplementary Fig. 5G), indicating that the increased transcription of *SATB1* may not be caused by phosphorylation-mediated changes in CTCF location. It has been shown that LINC00346 interacts with CTCF, which prevents CTCF from binding to the c-Myc promoter, thereby alleviating CTCF-mediated c-Myc inhibition [41].

Finally, we found that Edelfosine, a specific inhibitor of KCNN3, can significantly suppress the migration and invasion of OXA-R HCC. Given that the abnormally elevated activity of the KCNN3 channel in OXA-R HCC, whether Edelfosine can be combined with oxaliplatin in the treatment of drug-resistant HCC needs further research.

## Methods and materials

The RT-qPCR primers, siRNA sequences and construction primers for the KOD-Plus Mutagenesis Kit information used in this study are listed in Table S1, antibody information is listed in Table S2 and chemicals are listed in Table S3.

### Plasmid construction and transfections

For over-expression and lentiviral packaging, pCMV-KCNN3-3*Flag, pCMV-Vector, PMD2G, and PSPAX2 were purchased from MiaoLing plasmid platform. si-KCNN3 and si-NC were purchased from QingKe Biotechnology (Shanghai, China) and used for gene knockouts. Sources of SPOP-related plasmids have been previously described [23]. KCNN3 and SPOP promoter plasmid mutants were generated using the KOD-Plus-Mutagenesis kit (TOYOBO) according to the manufacturer’s instructions. All constructs were validated by DNA sequencing.

### Cell culture, transfection, and lentivirus infection

HEK-293T and HeLa cell lines were obtained from Haixing Biosciences (Suzhou, Jiangsu, China). Human hepatocarcinoma cell lines (HepG2 and Huh7) were obtained from Procell Life Science & Technology (Wuhan, Hubei, China). Human liver cell line (Huh7- luciferase) was obtained from Nuobo Biological Company (Hangzhou, Zhejiang, China). HEK-293T, HeLa, Huh7- luciferase, HepG2 and Huh7 cells were cultured in Dulbecco’s Modified Eagle Medium (DMEM, Meilunbio, China) with 10% Fetal Bovine Serum (FBS, Standard Quality, OriCell, China). All cells were grown at 37 °C with 5% CO_2_. For transfection, cells were transiently transfected with plasmid and siRNAs, using Lipo6000 Transfection Reagent (Beyotime, Shanghai, China) according to the manufacturer’s protocol.

### Western blotting (WB)

Cells were lysed with RIPA lysis buffer (High) supplemented with protease inhibitors on ice for 30 min. Lysates or immune-precipitates were subjected to SDS-PAGE, and proteins were transferred to nitrocellulose membranes (GE Healthcare Sciences). Membranes were closed in Tris-buffered saline (TBS, pH 7.4) containing 5% skim milk and 0.1% Tween 20, washed twice in TBS containing 0.1% Tween 20, and incubated overnight at 4 °C with primary antibody, followed by the secondary antibody for 1 h at room temperature. The target proteins were visualized using an enhanced chemiluminescence (ECL) system (Santa Cruz Biotechnology). WB was performed 2–3 times from at least two independent experiments, and representative images are shown. The original Western blots are shown in Supplementary Materials S3.

### In vitro ubiquitination assays and co-immunoprecipitation (CO-IP)

HEK-293T and Huh7 cells were transfected with HA-ubiquitin and/or the indicated constructs. 36 h after transfection, cells were treated with MG-132 (20 μM) for 8 h before harvesting, then lysed in RIPA lysate (High) and frozen at –80 °C. For CO-IP, the protein lysate was centrifuged at 12,000 × rpm for 20 min. The supernatant was removed and incubated with anti-Flag M2 agarose beads (Sigma, USA) or with recombinant protein G sepharose beads (Saier, China) at 4 °C overnight. The bound beads are then washed three times with BC100 buffer (20 mM Tris–Cl, pH 7.9, 100 mM NaCl, 0.2 mM EDTA, 20% glycerol) containing 0.2% Triton X-100. Proteins were eluted with Flag peptide for 4 h at 4 °C or by boiling in SDS-PAGE solution. The ubiquitinated forms of KCNN3, as well as the immunoprecipitated pull-down proteins, were detected by western blotting using anti-HA antibody, anti-ubiquitin antibody, or anti-KCNN3 antibody coupled with other labeling antibodies.

### Quantitative real-time reverse transcription PCR (RT-qPCR)

Total RNA was isolated from the indicated cells using TRIzol reagent (Tiangen, China), and cDNA was reverse-transcribed using the HiScript® II 1st Strand cDNA Synthesis Kit (Vazyme, China), according to the manufacturer’s instructions. PCR amplification was performed using SYBR Green PCR Master Mix Kit (Vazyme, China). All quantifications were normalized to the levels of the endogenous control group GAPDH.

### GST pull-down assay

GST fusion proteins were immobilized on glutathione–sepharose beads (Amersham Biosciences, USA). The beads were washed using pull-down buffer (20 mM Tris–HCl pH 7.5, 150 mM NaCl, 0.1% NP-40, 1 mM DTT, 10% glycerol, 1 mM EDTA, 2.5 mM MgCl2, and 1 μg/ml leupeptin). The beads were incubated with recombinant protein tagged with His for two h before being washed five times with binding buffer. Finally, the beads were resuspended in a sample buffer, and the bound proteins were subjected to SDS-PAGE and Western blot analysis.

### Immunofluorescence (IF) assay

For cellular immunofluorescence, the cells were placed on a chamber slide and fixed with ice formaldehyde for 5 min. After washing with PBS, it was incubated with 0.2% Triton X-100 at 4 °C for 5 min. Then the cells were washed with PBS, blocked with 0.5% BSA for 1 h, and incubated at 4 °C overnight with the primary antibody. After PBST washing, the fluorescently labeled secondary antibody was coated, and DAPI was re-stained at room temperature for 1 h. The cells were visualized and imaged using a fluorescence microscope (Nikon Ds-Ri2, Japan). Fluorescence intensity was analyzed by ImageJ and analyzed by GraphPad Prism software (v8.0).

### Analysis of cell membrane fluorescence intensity

The images are analyzed by ImageJ:

1. Process–Filters–Gaussian Blur
2. Cell segmentation: MorphoLibJ Segmentation, Choose Border Image and Tolerance, Create Mask
3. Get membrane ROI: Morphological Filters, Choose Gradient, Create Selection, Measure ROI.

### Analysis of integrated fluorescence signal in the nucleus

The images are analyzed by ImageJ:

1. Image-Color-Split channels
2. Image-Adjust-Threshold
3. Process-Binary-Watershed
4. Analyze particles-ROI manager-Show all

### Electrophysiology (Patch-clamp assays)

HEK293T cells were cultured in dishes (430166, Corning) used for KCa2.3 current recording. The cells were placed into a cell chamber mounted on the stage of an inverted microscope (Olympus, IX70, Japan), and allowed to settle to the bottom of the cell chamber before being superfused with bath solution (2 ml/min). Whole-cell current was recorded with an Axopatch 200B amplifier and Clampex 10 software (Axon Instruments, USA). Glass electrodes were pulled with a Brown-Flaming puller (Model P-97, Sutter Instrument, CA), and the resistance of the electrodes was 2–3MΩ when filled with pipette solution. Pipette potentials were zeroed before the pipette contacted the cells. After a gigaohm seal was obtained by negative suction, the cell membrane was ruptured by gentle suction to establish a whole-cell configuration. The electrical signal was low-pass filtered at 5 kHz and stored on the hard disk of a computer. All experiments were conducted at room temperature.

The bath solution contained NaCl 140 mM, KCl 5.4 mM, MgCl2 1 mM, CaCl2 1.8 mM, Glucose 10 mM and HEPES 10 mM; pH adjusted to 7.3 with NaOH. The pipette solution contained K-aspartate 110 mM, KCl 20 mM, Mg-ATP 5 mM, GTP 0.1 mM, HEPES 10 mM, EGTA 5 mM; pH adjusted to 7.2 with KOH. All chemicals were purchased from Sigma-Aldrich. ML133 (1222781-70-5) was dissolved in DMSO and prepared as a 3 or 10 mM stock solution stored at −20 °C.

### Plasma membrane isolation assay

HEK293T cells were treated with the Membrane and Cytosol Protein Extraction Kit (P0033, Beyotime, China). Refer to the instructions for the procedure. A Western blot was performed on the extracted membrane and cytoplasmic proteins, and ImageJ was used for quantitative analysis of protein bands.

### Protein half-life assays

For the half-life study, 20 μg/ml cycloheximide was added to the medium. At the indicated time points, the cells were collected, and protein abundance was measured by WB.

### Tissue samples and immunohistochemistry (IHC)

Archived formalin-fixed, paraffin-embedded HCC samples were collected from patients diagnosed with primary hepatocellular liver cancer after radical HCC resection at Affiliated LiHuiLi Hospital of Ningbo University from January 2020 to August 2022. Malignant disease or having received preoperative treatment (chemotherapy and/or radiotherapy) was excluded from this study. The retrieval of tissue and clinical data was approved by the Institutional Review Board of Ethics of Ningbo University (NBU-2022-123).

A total of HCC tissues were cut to 4 μm thickness, heated at 65 °C for 2 h, dewaxed in xylene, and rehydrated in a series of graded ethanol. Antigen repair was performed by heating tissue sections in ethylenediaminetetraacetic acid buffer (pH 9.0) for 10 min using a pressure cooker and then cooling to room temperature. After 30 min with peroxidase blocking reagent (3% H_2_O_2_ solution), tissues are washed 3 times with PBST solution and incubated overnight at 4 °C in a humidified chamber with KCNN3 primary antibody (Abmart, TD13233, China) or SATB1 primary antibody (Abmart, T55078, China). After washing the tissue Sections 3 times with PBST, they were incubated with HRP polymeric anti-mouse/rabbit secondary antibody for 30 min. The antibody assay was visualized using the DAB assay kit (Solarbio, #G1212, Beijing, China) to visualize the antibody detection. Slides were re-stained with hematoxylin.

All samples were reviewed by two independent pathologists experienced in the evaluation of IHC who did not know the clinical outcome of these patients. We assessed the percentage of positively stained cells and the intensity of staining to determine KCNN3 and SATB1 expression semi-quantitatively. The percentage of positively staining cells was scored as follows: 0, 0; 1, <50%; and 2, > 50%. The intensity of staining was graded as follows: 0 (no or weak staining = light yellow), 1 (moderate staining = yellowish brown), and 2 (strong staining = brown). The total score for KCNN3 and SATB1 expression was the sum of the percentage of cells scored against positive staining and the intensity of staining score, and a total score from 0 to 4 was assigned. For statistical analysis, the final score was a combination of the independent scores assigned by the two pathologists reported in this study. Any differences in scores were resolved by discussion between the two pathologists.

### Phosphorylomic analysis

We transfected Huh7 cells with si-KCNN3 and si-NC, respectively, and performed KCNN3 knockdown and control treatment. Each group had three replicates. We have deposited the phospho-proteomics data in PRIDE; the corresponding accession number is PXD070980.

1. enzymolysis

Protein extract was treated with 10mM DTT at 95 °C for 3min and alkylated using 25 mM iodoacetamide at room temperature for 30min while being protected from light and subjected to clean up and digestion with single-pot solid phase-enhanced sample preparation (SP3) beads. Briefly, two types of Sera-Mag SpeedBead carboxylate-modified magnetic particles (hydrophilic particles 45152105050250, hydrophobic particles 65152105050250; Cytiva, USA) were used. These beads were combined at a 1:1 (v/v) ratio, washed twice with distilled water, and reconstituted in distilled water at a concentration of 50 µg solids/μl. 60 μl of $P3 beads was added to 300 μg of protein in lysis buffer, followedby 100% acetonitrile (ACN) to bring the final concentration to 70% (v/v). with mixing for 10 min. The supernatant was discarded, and the pellet was washed with 80% ethyl alcohol and 100% acetonitrile (ACN). The beads were then resuspended in 100 μl of 50 mM Tris-HCl (pH 8.0) with trypsin (V5113, PromegaUSA) at a ratio of 1:50 (to protein) and mixed gently at 37 °C overnight to digest proteins. After digestion, beads were pelleted by centrifugation (20,000 × *g*, 1min, 24 °C) and supernatants containing peptides were transferred. Peptides were acidified with TFA to 1% final concentration and desalted using in-house-made stage tips packed with Cl8 disks (Empore) according to [57].

2. Enrichment of phosphorylated peptides

3. Liquid chromatography-mass spectrometry analysis

nanoElute system (Bruker Daltonics) and timsTOF HIBmuker Daltonics) were used for analysis

### RNA sequencing (RNA-seq)

Total RNA was isolated from each sample using the RNA mini kit (Qiagen, Germany). RNA quality was examined by gel electrophoresis and with Qubit (Thermo, Waltham, MA, USA). For RNA-sequencing, 1 μg of total RNA was used for library construction. Sequencing libraries were generated using VAHTSTM Stranded mRNA-seq Library Prep Kit for Illumina®. The libraries were sequenced as 150 bp paired-end reads using Illumina Novaseq6000 according to the manufacturer’s instructions by the commercial service of Genergy Biotechnology Co., Ltd. (Shanghai, China). The raw data of all samples had been submitted to the Sequence Read Archive at the National Center for Biotechnology Information (http://www.ncbi.nlm.nih.gov/sra).

### RNA-Seq data analysis

The raw data were handled by Skewer, and data quality was checked by FastQC v0.11.2. The read length was 2 × 150 bp. Clean reads were aligned to the Human genome hg38 using STAR. StringTie. The expression of the transcript was calculated by FPKM (Fragments Per Kilobase of exon model per million mapped reads) using Perl. Differentially expressed analysis between matched tumor and normal samples was performed by the R package DESeq2 with the likelihood ratio test option. Differentially expressed genes exhibiting two-fold changes and Benjamini and Hochberg-adjusted *P* values ≤ 0.05 were selected. If the gene DESeq2 normalized read count value was close to 0, a log_2_ transformation was performed after adding 1. The expression values were visualized by the R package pheatmap. Then DEGs were chosen for function and signaling pathway enrichment analysis using the GO and KEGG databases. The significantly enriched pathways were determined when *P* < 0.05, and at least two affiliated genes were included. PPI analysis of differentially expressed genes was based on the STRING database, which includes known and predicted Protein–Protein Interactions. And the network was established according to the known interaction of the selected reference species. GSEA is a computational approach to determine if a pre-defined Gene Set can show a significant, consistent difference between two biological states. The genes were ranked according to the degree of differential expression in the two samples, and then the predefined Gene Sets were tested to see if they were enriched at the top or bottom of the list. Gene set enrichment analysis can include subtle expression changes. We use the local version of the GSEA analysis tool; Hallmark and KEGG data sets were used for GSEA independently.

### Luciferase reporter assay

The promoter region was cloned and inserted into the luciferase reporter vector pGL4.10, which was purchased from Qingke Biotechnology, Shanghai, China. Luciferase reporter assay was performed according to the manufacturer’s protocol (Vazyme, China, Cat No: DD-1205-01). Briefly, the constructed reporter vector was transfected into Control- or CTCF-overexpressing HEK-293T cells using Lipo6000TM transfection reagent (Beyotime, China, Cat No: C0526). At 48 h post-transfection, the cells were lysed for luciferase measurement and firefly luciferase activity was normalized to Renilla luciferase activity.

### ChIP assay

For the ChIP assay, the procedure was performed using the BeyoChIP™ Enzymatic ChIP Assay Kit (Beyotime, P2083S, China) according to the manufacturer’s instructions. In brief, cells grown in 10 cm dishes were fixed with 1% formaldehyde and subjected to chromatin fragmentation by ultrasonication. The fragmented chromatin was immunoprecipitated with CTCF antibody (Abclone, A19588, China) overnight at 4 °C, using IgG (Proteintech, B900620, China) as a negative control. Protein A/G magnetic beads (Beyotime, P2083S, China) were then added and incubated with the samples for 2 h at 4 °C. The bead-bound complexes were washed stepwise with low salt immune complex wash buffer, high salt immune complex wash buffer, LiCl immune complex wash buffer, and TE buffer (all from Beyotime, P2083S, China). After elution with Elution Buffer (Beyotime, P2083S, China), the cross-links were reversed by treatment with 5 M NaCl, 0.5 M EDTA, 1 M Tris (pH 6.5), and 20 mg/ml proteinase K. The DNA was purified using a DNA purification kit (Beyotime, D0033, China) and analyzed by PCR and real-time PCR to determine the enrichment of the SATB1 promoter region. Each ChIP experiment was repeated in at least four independent assays.

### Cell proliferation assay

The cell proliferation rate was determined using Cell Counting Kit-8 (CCK-8) (Dojindo Laboratories, Japan) according to the manufacturer’s protocol. Briefly, cells were inoculated onto 96-well plates at a density of 1000 cells per well. During the incubation period of 0–6 days, 10 μl of CCK-8 solution was added to the cell cultures and incubated for 2 h. The OD value of each well was measured at 450 nm using a microplate absorbance reader (Bio-Rad, US). Each assay was performed in triplicate.

### Colony formation assay

Huh7 and HepG2 cells were seeded in 6-well plates containing 1500 individual cells per well in triplicate. After 2 weeks of incubation, the cells were fixed in 100% methanol for 5 min at room temperature and then stained with Giemsa dye for 20 min (Solarbio, China).

### Wound-healing assay

Huh7 and HepG2 cells were seeded in 6-well plates (Costar, Corning, US) and cultured to 80% confluence. Monolayers of cells were damaged by removing the culture insert and rinsed with PBS to remove cellular debris. After treatment with Mitomycin C for 1 h (5 μM) (GLPBIO, #GC12353, CA, USA), the medium was replaced with fresh serum-free DMEM. Images were acquired using a fluorescence microscope (Nikon Ds-Ri2, Japan) at 0 and 48 h after migration. The area of wound-edge healing was calculated between 0 and 48 h.

### Transwell (Migration and invasion) assay

Huh7 and HepG2 cells were precultured in a serum-free medium for 48 h. For the migration assay, 4 × 10^4^ cells were inoculated into the upper side of a modified Boyden chamber (8.0 μm, #3342, Corning, NY, USA), and the lower chamber was filled with medium containing 5% FBS. After 24 h, we carefully removed non-migrating cells from the upper chamber with a cotton swab and stained and counted migrating cells in nine different areas below the filter. For the invasion assay, 6 × 10^4^ cells were inoculated into the upper side of a modified Boyden chamber (8.0 μm, #3342, Corning, NY, USA), which was coated with matrix gel/fibronectin (BD Biosciences, USA), and the lower chamber was filled with medium containing 5% FBS. After 48 h, we carefully removed non-migrating cells from the upper chamber with a cotton swab and stained and counted migrating cells in nine different areas below the filter. Photographs of the stained cells were taken with a microscope (magnification: 200×).

### Xenograft mouse assay

We obtained NOD-SCID mice of 4–6 weeks old (strain t001492), weighing 15–25 g, from GemPharmatech (Nanjing, China) for the in vivo xenograft mouse assay. 3 copies of si-KCNN3 Huh7 cells and 6 copies of si-NC Huh7 cells (3 × 10^6^ cells/ml) were prepared in advance, 2 days before the experiments. 6 copies of si-NC Huh7 cells (3 × 10^6^ cells/ml) were respectively transfected with FLAG-KCNN3 (KCNN3-OE) and EV (empty vector) plasmids. 3 copies of si-KCNN3 Huh7 cells (3 × 10^6^ cells/ml) were transfected with the EV plasmid. After digestion and centrifugation, 3 cells from each group were resuspended with 300 μl PBS and then added with 300 μl matrix gel (“Mogengel”, China, cat#082704). All Huh7 cells were divided into three groups of si-NC + EV, si-NC + KCNN3-OE and si-KCNN3 + EV, with 3 mice in each group. Groups were subcutaneously transplanted to the right side of 9 mice (200 μl of cell suspension per mouse). We measured the tumor volume every 4 days starting from the 4th day after transplantation and ending at 16 days. The formula was calculated as: tumor volume = (long × wide^2^) × 1/2. At the end of the experiment, the tumors were imaged and weighed after the mice were euthanized. All animal experiments were conducted according to the protocol approved by the Animal Protection Committee of Ningbo University. All animal experiments were conducted in accordance with protocols approved by the Institutional Animal Care and Use Committee at Ningbo University Health Science Center (AEWC-NBU-2023-186).

### In vivo imaging

All animal experiments were conducted in accordance with protocols approved by the Zhejiang Chinese Medicine University Laboratory Animal Research Center (Ethics number:16372). To establish the HCC model, BALB/c nude mouse (sex: male, Supplier: Shanghai Slack Company, specification (weight or age in days, weeks, months): 9–15 g (3–5 weeks). Mice were divided into 5 groups (4 mice per group). Each group was inoculated with 2 × 10^6^ huh7 cells expressing luciferase(luciferase-huh7) through the tail vein (day 0). On day 7, D-fluorescein (150 mg/kg) was injected intraperitoneally, followed by respiratory anesthesia, and administered by non-invasive whole-body imaging system (IVIS Spectrum; PerkinElmer, Waltham, USA) in vitro imaging of mice. In vitro imaging was performed at 7, 14, 21, and 28 days after caudal intravenous injection. Fluorescence intensity was analyzed by the Living Image software.

### Statistical analysis

Statistical calculations were performed using GraphPad Prism software (v8.0), and images were analyzed and quantified using ImageJ software (v1.46). All data are shown as the mean ± SD of experiments repeated at least three times. The differences between the two groups were analyzed using Student’s *t* test, and multiple comparisons were performed using two-way analysis of variance (ANOVA). * represents *p* < 0.05, ** represents *p* < 0.01, *** represents *p* < 0.001, no significance (ns) indicates *P* ≥ 0.05.

## Supplementary information

Supplementary materials2

Original Data

Supplementary materials1

## Data Availability

The datasets and computer code produced in this study are available in the following databases: The Cancer Genome Atlas (TCGA): https://portal.gdc.cancer.gov/. Gene Expression Omnibus (GEO): https://www.ncbi.nlm.nih.gov/geo/. Timer2.0: http://timer.cistrome.org/.
