# Supplementary material for: SPOP-mediated K27-linked non-degradative ubiquitination of KCNN3 suppressing HCC progression via the CTCF-SATB1 axis
Source: Cell Death Dis. 2026 May 10;17(1):612. doi: 10.1038/s41419-026-08765-3 (PMC13324860; doi:10.1038/s41419-026-08765-3)
Supplement: Supplementary file 1 — Supplementary materials2 [file 41419_2026_8765_MOESM1_ESM.pdf]

Original Data

Figure1A		
**ion channels**	**HCC differential gene GSE65486(up)**	**HCC differential gene GSE254461(up)**
HTR3A	PTPRC	ITGA6
HTR3B	FAM5B	DCAF4L2
HTR3C	HMCN1	MDK
HTR3D	ESRRG	RBM24
HTR3E	PAPPA2	KCNJ5
ASIC1	CEP350	CD34
ASIC2	C20orf26	CNNM1
ASIC3	PLXNA2	PPP4R3C
ASIC4	RGS5	TERT
ASIC5	ASTN1	FAM133A
ANO1	MED12	MELK
ANO2	C1orf129	SLC7A11
ANO3	FAM78B	CASC9
ANO4	C1orf27	CDKN2A
ANO5	NME7	PLVAP
ANO6	DNM3	CDKN2C
ANO7	F13B	ANLN
ANO8	TNR	CENPF
ANO9	CR2	DCAF8L2
ANO10	FCER1A	GABRD
MIP	RGL1	SIX1
AQP1	RXRG	RACGAP1
AQP2	TNN	NDUFA4L2
AQP3	CLSTN2	LINC02241
AQP4	KCNT2	SEMA7A
AQP5	SPTA1	LEF1
AQP6	NCALD	GTPBP2
AQP7	JARID1B	LOC102724813
AQP8	ADCY8	E2F7
AQP9	FCRL5	UBD
AQP10	CD244	LINC02476
AQP11	ZPLD1	CSMD1
AQP12A	APOA2	HS3ST2
AQP12B	TADA1L	XAGE1B
BEST1	SPATA17	XAGE1A
BEST2	USH2A	FRG1EP
BEST3	CACNA1E	JCAD
BEST4	MUC1	PIK3IP1
CACNG1	FASLG	ST8SIA6-AS1
CACNG2	FLJ46284	TOP2A
CACNG3	MACROD2	TMEM98
CACNG4	BRP44	GBAP1
CACNG5	DPT	MATN3

CACNG6	C1orf49	LINC02882
CACNG7	C1orf32	SPINK5
CACNG8	TNNT2	KIF2C
CACNA1A	SLC30A10	MKRN3
CACNA1B	CFHR5	CHRM4
CACNA1C	C1orf112	KCNJ2
CACNA1D	PPM1H	DUXAP9
CACNA1E	RAD21	OLR1
CACNA1F	FLJ43585	LOX
CACNA1G	ETV3	SPP1
CACNA1H	ADAM15	LOC107984118
CACNA1I	CD5L	CDH13
CACNA1S	ZNF512	FBXO43
CACNA2D1	SERPINC1	THY1
CACNA2D2	SLC26A7	FIGNL1
CACNA2D3	C20orf74	FERMT1
CACNA2D4	CRB1	MTMR11
CACNB1	BAT2D1	CBX2
CACNB2	C1orf21	CPNE2
CACNB3	ATP1A2	HOXA10
CACNB4	KIFAP3	ESM1
CATSPERB	CRP	FZD6
CATSPERD	CAPN2	LINC01446
CATSPERE	FAIM3	MKI67
CATSPERG	LMX1A	FAM83D
CATSPERZ	TBX19	LAMA3
CATSPER1	PBX1	MMP24
CATSPER2	C1orf19	CDC20
CATSPER3	IGSF9	ZIC2
CATSPER4	FLAD1	LOC105377896
CFTR	SELL	LOC105374673
CLIC1	ASPM	DUXAP8
CLIC2	COL14A1	CDCA5
CLIC3	DDR2	TNFRSF19
CLIC4	SLC30A8	EPHB2
CLIC5	TTN	HOXA3
CLIC6	XCL1	ODAM
CLCNKA	C1orf65	STRIP2
CLCNKB	SNTB1	KIF23
BSND	KCNAB1	DIPK2B
CLCN1	CHI3L1	SPARCL1
CLCN2	NBPF10	HSPA12B
CLCN3	FAM5C	MYEF2
CLCN4	NLGN1	LOC102723347
CLCN5	MAEL	BIRC5
CLCN6	FMO4	CAP2

CLCN7	FAM129A	C8orf33
CHRNA1	PAK3	NDC80
CHRNA2	TPR	HJURP
CHRNA3	NOS1AP	GTSE1
CHRNA4	STK3	PRR5L
CHRNA5	SEC16B	CEP55
CHRNA6	LAD1	ASPM
CHRNA7	LOC642924	LAMA4
CHRNA9	MOSC1	MYBL2
CHRNA10	ARHGEF11	UBE2T
CHRNB1	TOMM40L	TP73
CHRNB2	UAP1	PLEKHA8
CHRNB3	EDEM3	TLX1
CHRNB4	SERTAD4	HMGN4
CHRND	C1orf105	PRC1
CHRNE	VPS13B	TGM3
CHRNG	TMEM55A	KIFC1
CNGA1	GRHL2	ZNF432
CNGA2	NAV1	PPP1R35-AS1
CNGA3	NBPF1	TBX4
CNGA4	GLT25D2	RRM2
CNGB1	PTPN14	RASGRF1
CNGB3	COPA	TMEM169
HCN1	EIF3H	ZNF707
HCN2	CR1	TTK
HCN3	SAMD12	DUXAP10
HCN4	POGK	SFN
GABRA1	NOV	PODXL
GABRA2	MR1	LOC105377267
GABRA3	C12orf40	SEMA3B
GABRA4	SDC2	ALDH3A1
GABRA5	UBR5	N4BP3
GABRA6	RABGAP1L	SLC7A6
GABRB1	CAMSAP1L1	CDT1
GABRB2	CHD1L	STMN1
GABRB3	CFH	NYAP2
GABRD	NIBP	DAPK2
GABRE	C8orf53	BUB1
GABRG1	HSD11B1	NKD1
GABRG2	CCDC19	LINC02835
GABRG3	SAC	LOC105379271
GABRP	DECR1	LOC105369315
GABRQ	ODF1	CD200
GABRR1	PIGR	NARF
GABRR2	CSMD3	COL4A1
GABRR3	CD46	SCN4A

GJA1	C4BPA	RBMY1A1
GJA3	LOC400794	ZNF229
GJA4	F5	LOC107987401
GJA5	ACCN1	BEND5
GJA6P	NAALADL2	SPC24
GJA8	SLAMF6	E2F8
GJA9	RAD54B	KIFC2
GJA10	RFWD2	PDGFD
GJB1	PTGS2	COL15A1
GJB2	LAMC2	PPP1R35
GJB3	CD1A	MEX3A
GJB4	NEK7	GBA1
GJB5	KCNJ10	CPLX2
GJB6	WDR70	LEF1-AS1
GJB7	PRELP	THBS4
GJC1	SPRR1A	DIAPH3
GJC2	SELE	NIHCOLE
GJC3	SELP	EXO1
GJD2	DCBLD2	LOC105375166
GJD3	C1orf114	DLGAP5
GJD4	IMPG2	KIF18B
GJE1	RALGPS2	RBMY1B
GRIA1	ATP6V1G3	LOC105376436
GRIA2	NUF2	ANKRD65
GRIA3	RGS21	ZNF572
GRIA4	PLA2G4A	CTTNBP2
GRID1	RGS16	PHYHIPL
GRID2	ACBD6	TMEM52B
GRIK1	KCNK2	FABP4
GRIK2	TMEM9	GMNN
GRIK3	PIGM	TARBP1
GRIK4	EFCBP1	KIF14
GRIK5	CADM3	NETO2
GRIN1	PRCC	FAM83H
GRIN2A	C1orf107	PAQR4
GRIN2B	FLJ16641	RGSL1
GRIN2C	COG2	RXRG
GRIN2D	FMO2	MAD2L1
GRIN3A	DENND1B	UGT2B11
GRIN3B	SLC25A44	PKMYT1
GLRA1	AKT3	CCDC170
GLRA2	GPA33	ZNF836
GLRA3	CD247	BUB1B
GLRA4	ENPP2	NEK2
GLRB	RASAL2	NOTCH3
HVCN1	ABL2	VWF

ITPR1	FLJ43860	CDK1
ITPR2	SLC9A9	TNFAIP8L2-SCNM1
ITPR3	CD1E	PTTG1
KCNMA1	PPM1L	CEP41
KCNN1	RGS4	NUF2
KCNN2	ANKRD45	LOC100294145
KCNN3	PFKFB2	PBK
KCNN4	KIAA1661	WDR76
KCNU1	C1orf116	RBM12B
KCNJ1	C1orf98	CDKN3
KCNJ2	TMEM79	ZNF792
KCNJ3	MGST3	CCNB2
KCNJ4	FCRL6	UCK2
KCNJ5	GPR110	SCNM1
KCNJ6	TMCO1	ADCK5
KCNJ8	PRG4	PARPBP
KCNJ9	KIRREL	CDCA8
KCNJ10	UCHL5	SHISA4
KCNJ11	SH2D1B	PLCG1
KCNJ12	IL19	RFX5
KCNJ13	RGS18	DEPDC1
KCNJ14	FCRLB	EBF1
KCNJ15	MMP16	SLC24A5
KCNJ16	SDCCAG8	PRC1-AS1
KCNJ18	C8orf38	CXCL10
KCNT1	DARC	IQGAP3
KCNT2	NFASC	STXBP6
KCNK1	POU2F1	ST14
KCNK2	C1orf9	LRRC14
KCNK3	CFHR4	CLSPN
KCNK4	LOC440704	LOC105379064
KCNK5	CCT3	LNCOC1
KCNK6	SLC9A11	MAP3K7CL
KCNK7	PDC	KCNN3
KCNK9	LAMC1	LOC105371664
KCNK10	FMOD	GPR158
KCNK12	NMNAT2	APLN
KCNK13	PLCH1	SCAMP3
KCNK15	ASH1L	TONSL
KCNK16	AIM2	NCAPH
KCNK17	ZBTB41	ACTN2
KCNK18	PEA15	CCDC34
KCNA1	GPATCH2	DTL
KCNA2	IVNS1ABP	ANKRD29
KCNA3	CSRP1	TTL
KCNA4	KLHL20	RASGRF2

KCNA5	SLC2A2	ZNF404
KCNA6	PGBD5	KIF15
KCNA7	HHAT	MSH2
KCNA10	GPR116	KIF18A
KCNB1	CD48	DSCC1
KCNB2	FMO6P	GDPD1
KCNC1	MAPKAPK2	TBC1D22A
KCNC2	KCNN3	USP54
KCNC3	TAF5L	TK1
KCNC4	FLJ46134	ZWINT
KCND1	TFB2M	CDK5R1
KCND2	VPS72	CEP68
KCND3	TNFRSF11B	SLC12A1
KCNF1	ATF6	ZNF468
KCNG1	C8orf70	COL7A1
KCNG2	CDH17	PI15
KCNG3	LOC643723	AXIN2
KCNG4	NTRK1	DDX53
KCNH1	EXT1	MCM10
KCNH2	ANGPT1	LINC02875
KCNH3	PMF1	HOXD4
KCNH4	DPM3	LCN2
KCNH5	KCNQ3	NELFE
KCNH6	MRPS28	CELF6
KCNH7	ZFAT1	TNFRSF4
KCNH8	CD1D	CDC6
KCNQ1	PGCP	EMCN
KCNQ2	ANGEL2	BMP4
KCNQ3	TRAF3IP3	ZNF385D
KCNQ4	KCNH1	ARHGAP11A
KCNQ5	KIAA1429	KIF20A
KCNS1	PLD5	LOC105369147
KCNS2	CAMK1G	EBF2
KCNS3	PAG1	DEPDC1B
KCNV1	MDFIC	LOXL2
KCNV2	TNNI1	LOC105372666
P2RX1	FMO5	CENPA
P2RX2	MDM4	RARRES1
P2RX3	PRRX1	PIK3C2B
P2RX4	ZNF678	ZSWIM5
P2RX5	OSGIN2	LRRC45
P2RX6	CYB5R1	KIAA1549
P2RX7	RELN	BCAT1
RYR1	CAPN9	MTBP
RYR2	BCAN	ZNF623
RYR3	GJA5	CENPM

SCNN1A	TOR1AIP2	RHBDL3
SCNN1B	SMYD3	CHEK1
SCNN1D	RNF19A	CD163L1
SCNN1G	ABCB1	LINC01311
NALCN	TROVE2	LOC105378589
SCN1A	PLEKHA6	SLC26A6
SCN2A	CDYL	AIFM3
SCN3A	ALDH9A1	RHBG
SCN4A	OTUD6B	TRIL
SCN5A	C1orf218	HOXD9
SCN8A	PI4KB	FRMD3
SCN9A	KIF26B	TREM2
SCN10A	SCYL3	PROCA1
SCN11A	TMEM64	NSMCE2
SCN1B	NSMCE2	CCNA2
SCN2B	TNFSF4	SLC22A11
SCN3B	IL10	RPL23AP82
SCN4B	FLVCR1	PMFBP1
TRPA1	STX6	TRIM45
TRPC1	KHDRBS3	MILIP
TRPC2	GRB14	ZNF366
TRPC3	MATN2	WASHC5
TRPC4	LOC284577	TROAP
TRPC5	ZNF706	KIF11
TRPC6	BLZF1	FAM3B
TRPC7	MPZL1	TM4SF19-AS1
MCOLN1	DKFZp434B1231	SCIN
MCOLN2	CR1L	TMEM255A
MCOLN3	NPL	LGR5
TRPM1	LCE1E	PCNA-AS1
TRPM2	SYT14	ZNF322
TRPM3	ATP1B1	VSIG10
TRPM4	SIPA1L2	RECQL4
TRPM5	DCST1	PYCR3
TRPM6	PPOX	MIR937
TRPM7	HIST1H2AC	LOC101928775
TRPM8	CENPF	RASL12
PKD2	IL7	CCM2L
PKD2L1	FCRL1	LOC728743
PKD2L2	SLAMF8	LINC02055
TRPV1	ARPC5	CEP131
TRPV2	ATP1A4	SMC2
TRPV3	CD55	LOC112268238
TRPV4	PROX1	ENTREP3
TRPV5	TSNARE1	SPC25
TRPV6	MFSD4	AP5Z1

TPCN1	ACSS1	FOXH1
TPCN2	TDRD5	PLOD3
VDAC1	CDC73	LOC101930112
VDAC2	RNPEP	ZEB1-AS1
VDAC3	LOC389676	DNAH12
LRRC8A	IRF6	TCF7
LRRC8B	C20orf39	EHMT2-AS1
LRRC8C	SYT2	KIF4A
LRRC8D	ITGA6	ASAP2
LRRC8E	C1orf76	ZNF696
ZACN	C1orf125	H4C15
	TMEM183A	E2F1
	KIAA0859	CCNE1
	IMMP2L	SERPINI1
	RPS6KC1	C21orf58
	DIAPH2	CAPN11
	IFI16	CBX8
	VSIG8	LOC105373690
	IQWD1	TTC9
	PIK3C2B	SPSB2
	XPR1	RHOBTB1
	GALNT2	TRIB2
	FMO9P	VPS28
	SLC19A2	CDKN2B-AS1
	CDC42BPA	CDC45
	KIAA1804	SLC7A6OS
	TRPS1	KCNMB2-AS1
	ALS2CR7	TEX41
	FIGN	COMMD4
	FER1L6	GTF2IRD1
	SLAMF7	ZBTB38
	VPS54	RHEBL1
	PM20D1	HMMR
	UCK2	DCAF13
	OR12D3	ANKRD13B
	C8orf37	COL8A1
	OLFML2B	BOP1
	LRRC52	FOXM1
	MARK1	ASIC1
	MID1	UHRF1
	DARS2	HOXB7
	CNBD1	PRR11
	DDEF1	POLR2K
	VWA3B	VLDLR
	PKHD1	ZSCAN9
	DNAH8	MND1

TRIM67	LOC105377979
RAB4A	C9orf152
C1orf55	GINS1
PEX19	DNMT3B
CNIH3	VIRMA
C1orf26	EFNA4
INTS7	FRS3
FLJ30838	OLFML2B
TGFB2	JOSD2
DNAH14	ZNF101
ARHGEF9	ECT2
CREG1	HES6
ARHGAP15	STXBP4
MRPL55	TOB2P1
RIPK2	LOC102724727
FCRL4	LOC101929479
SLC25A32	KITLG
NPAL2	PLK1
LOC286144	FBXL18
PKHD1L1	ZNF543
FAM63A	CKLF
CTSE	CCNB1
PLEKHF2	CXCL11
LMNA	TAF6
UBE2E2	LOC107985532
DTL	SOX18
FCGR2B	SHCBP1
RGS1	TEDC2
DEPDC2	ARHGEF39
CACYBP	MSH5
IL24	TTLL7
CD160	GNAZ
KCNK9	CDC25C
APCS	PIF1
VANGL2	LSM2
FMO1	EHMT2
DPY19L4	SHLD1
OR6K6	RPS6KL1
ZNF124	NR2C2AP
LALBA	TIGD5
NR5A2	NUSAP1
PKIA	FUT2
PLCB1	SACS
CLIC5	NUP37
C4BPB	TAF1A
ELK4	PIAS3

MPZ	LOC101927209
CASQ1	ABCC10
C20orf3	CENPI
CTHRC1	MICB
HAS2	PTK2
NCOA2	CCDC103
FMNL2	COL4A2
SLC4A10	MCM8
INTS8	CCDC24
ANKS1B	NCAPG
FABP4	FAM111B
SLC35F3	DNAL4
TCEB1	EFCAB7
LRRN2	TDRKH
RGS7	OTOAP1
FAM135B	ITGA2
TMOD4	LOC105375957
DERL1	UBAP2L
NCF2	GLUL
C1orf150	SIRT7
DISC1	DBF4B
DEPDC6	MECOM
DKFZp686F0839	POLQ
HIVEP1	FRG1DP
FMO3	SEMA3F
BCL9	NEDD1
ZNF804B	ATP6V1C1
PTK2	MSH5-SAPCD1
SPTLC3	SNHG20
C1orf204	SP5
TSPAN12	MED22
LMBRD2	LOC107985050
CD1B	CASK
STMN2	CHRM3
PEAR1	ZNF300
OR6Y1	STIL
MRPL13	LSM4
ETNK2	LMOD1
KCTD3	ADAMTS5
ANXA13	AURKB
LOC376693	MORN2
LYNX1	KCNE3
JRK	ANO2
C20orf23	CRNDE
C17orf67	RASL10B
SLCO5A1	LOC107986819

C1orf25	CCL18
IKBKE	SPDL1
SND1	NAT14
NVL	IRF5
ZHX2	CCNF
RBM12B	CYTH3
MSTO1	LRRCC1
CENPL	BCAN-AS1
ARNT	NES
LAMB3	LOC112268256
AZIN1	ZNF845
FLJ43505	EIF3D
DYRK3	DLG5-AS1
PGBD2	CKAP2L
MOSC2	DBNDD1
KIF21B	ZNF184
RGS22	CBX4
F11R	ZNF544
DENND3	LOC107986871
FAM89A	TMTC2
HIST1H3D	LIPE
ADAMTSL4	TSSK6
NCSTN	APOBEC3B
LYPLAL1	NSUN5
SPEF2	ZNF528
DCC1	AURKA
RSRC1	ZNF501
FKSG49	LOC105378138
ILF2	PABPC4L
SCYL1BP1	TMEM145
RAB3GAP2	TRAIP
TTC27	CELSR1
RCAN2	BAIAP2-DT
LELP1	LINC02315
DUSP12	ARMC9
STAB2	GPAA1
FAAH2	ZKSCAN5
CFHR2	APBB1
PPP1R15B	TMC7
TCF4	LOC105379272
EXO1	ZSCAN16
REN	HSDL1
RUNX1T1	LOC102723724
RBM35A	DCK
LIX1L	SPA17
FLJ35530	H2BC20P

MED30	HELLS
CST4	FAM72A
UQCRB	GOLGA2P10
C1orf110	SFRP4
NBN	SCRIB
ACVR1	HLA-H
DPYS	SANBR
HRNR	CFAP53
TDRD10	MC1R
FARS2	PSMG3
RALYL	TCF19
LOC149773	ZBTB12
PYHIN1	LOC105373218
PTPRV	HAGLR
ZNF333	FAT1
SH3BP5L	TRIP13
TRAF5	ZNF385C
ATP2B4	SLC41A3
PARP8	OTUD6B
SLC26A5	ZNF284
RNASEL	MCM3
LRP1B	SEMA5B
CTSK	POLG2
C1orf106	ARL4A
HDGF	H4C14
CXorf6	SOSTDC1
ZNF672	TAF2
GPR161	TCOF1
MTDH	CKLF-CMTM1
HIST2H2AC	RAD51AP1
VSIG4	SIRT6
SNAPIN	PECAM1
PIAS3	SKA1
PEX5L	CCT6A
LCE2B	RNF43
C1orf14	CD109
COL22A1	LZTS1
SOX13	CCDC142
NUDT17	E4F1
GTDC1	BARD1
PHLDA3	SGO2
GRM3	ZNF737
CUX2	APOBEC3B-AS1
MNDA	PPP1R16A
LOC286094	UBR5
LMOD1	CCEPR

TATDN3	EDIL3
OR10J1	ANKRD27
SMCP	BCAP31
LHX9	NEB
ZBTB10	GRK3
COLEC10	FBXO32
SRGAP2	THADA
DUSP10	LINC02994
C8orf34	SVBP
SLC26A9	MCMDC2
HEPH	POLD1
ZFHX4	COMMD5
SKP2	ZNF550
VASH2	TXNRD1
ANXA9	TCTN1
VAMP4	RHBDD3
HS6ST2	SKA3
ZFPM2	FAM13C
ATAD2	CACNB3
MBNL3	TRIM16
DUSP23	ATAT1
C1orf2	RASSF4
C1orf53	C1orf131
GMDS	AIFM2
GON4L	CDK5
DCST2	ZFP69B
DKFZp686O1327	TCHP
VPS45	ALDH1L2
C1orf61	PLK4
HFE2	FOXS1
ABI3BP	SMAD3
USF1	SLC1A4
CYP39A1	ZNF252P-AS1
KIAA1614	TIGD7
ARHGAP25	BMS1P18
S100A2	ADAMTS7
LOC730100	TG
POP1	TRPC6
WWP1	USP46
PIK3R4	TP53I3
EPRS	ZNF551
ITLN1	DLG5
IQGAP3	NOTUM
CD1C	BAMBI
UBE2E3	H2AC11
KCNMB3	HMGB2

GLRX2	MSTO1
NEDD9	ZBTB22
C1orf95	VPS13B
LGALS8	DPCD
FAM71A	ZNF826P
FBXO32	LOC105369468
OTUD7B	NXPH3
CA13	LAMC1
TTC13	DUT
TIPRL	CPVL-AS2
THEM4	CDCA3
ZNF704	NEDD4L
GALNT13	CCHCR1
BAI1	LOC105378281
hCG_1990170	GPX8
PRUNE	ALKBH6
RPTN	RNF215
PAK7	GM2A
AQP10	CCND3
FZD6	SCX
LCE2A	PPOX
BFSP1	ADCY10P1
RCSD1	COL4A2-AS2
GATAD2B	ZNF28
TG	LOC102725051
LY9	SIGLEC15
SLC6A6	HCP5
STAU2	MYBPC1
CCNO	MARCHF9
ZNF322A	ZMYM1
CD34	ZNF841
NES	CDKN2B
ADAMTS4	TUBA1B
CALB1	RFXANK
CD86	LOC102724135
ST3GAL1	BMS1P22
RBBP5	VPS45
WDSOF1	DNAAF5
SOAT1	ZNF226
NPHP3	BZW2
ANKMY2	LINC01151
PCNXL2	TM4SF19-DYNLT2B
RYR2	MYO19
TP53BP2	CDCA4
S100A12	PCLAF
OBSCN	LEMD2

RGS8	INCENP
ITLN2	IL6ST-DT
MEF2D	LOC107984851
TMEM67	UBQLN4
LEMD1	HCG27
CKS1B	SLC38A6
SNX16	MCM5
MSI2	TEX22
C1orf198	DAXX
ZBTB20	LOC441666
RIMS2	CENPL
KCNB2	CEMIP
GLRA2	LOC100287290
PPP2R5A	SPIRE2
MCF2L2	ZNF517
OXR1	PACC1
PEX11B	NOX4
SH2D2A	TOX3
CD84	AKIP1
SPRR2E	TAOK2
LGTN	MNS1
HIST2H2AB	PPP1R2
NUP210L	CENPE
SLAMF1	CENPW
TMEM58	MAP6D1
IPO9	ANKRD36
JPH1	P4HA2
OAZ3	SIX4
FAM91A1	ZNF74
FLJ39080	RPUSD1
MLZE	GJC1
C1orf31	VPS72
TOR1AIP1	LOC107987237
LQK1	PDGFB
NPHS2	AP4M1
LOC149643	MED25
NRSN1	ANGPT2
RRM2B	THSD1
FCER1G	ZNF765
LEMD2	ZNF251
BTG2	GLI4
CNTN1	ZNF736
PFTK1	TPM1-AS
TOR3A	OIP5
LPP	RUVBL2
LSM2	DPH7

EHBP1	UTP23
TNRC4	WDR35
ACP6	SSBP4
PPP1R12B	EIF4ENIF1
FAM92A1	ARL2-SNX15
ELF3	FANCE
OFCC1	LINC00504
S100A7A	PYGO2
ADAMTS12	GCNT4
RRP15	BUD31
PCP4L1	CAPG
PVT1	TECPR1
C1orf92	GOLGA2P7
C6orf195	IDO1
GDF6	SBSPON
KIF6	PCNA
ARHGAP30	SPAG4
ATXN1	RGS5
ANKRD35	C1QTNF3
EYA1	KIF22
PKP1	GTF2IP4
LAPTM4B	RFC3
WDR21C	H4C9
ATP8B2	LOC100130027
CHMP4C	CCDC77
BAALC	METTL18
IGSF8	YDJC
NBPF11	EVPL
FCGR3A	LOC105372310
LACTB2	H3C10
SP4	WASHC2C
LTA	H2BC11
GEM	CRYBG2
CRNN	CKAP2
HORMAD1	WASHC2A
QSOX1	HRCT1
CEP170	BRIP1
SLAMF9	FAM72C
RPL30	KIF7
DNAJC5B	PLXDC1
FAM49B	OLFML2A
RABIF	PCDH17
MTX1	LINC03034
MYOG	GSTO2
EPHA6	CLCN2
KIAA0907	SLFN12

TSPYL5	MIR4707
SFT2D2	MTFR2
FCRLA	PSPH
MYH15	H2AC8
PVRL4	CILK1
ECM1	PDK1
HCN3	CXCL9
IFT80	SOX9
HRSP12	RASD2
GPRASP1	CDPF1
LGR6	JRK
EPHB1	FANCF
KHDRBS2	SEMA3G
LENEP	LOC112267872
LOC129607	CPSF1
GTF2H1	DNAJC6
MRPL24	BTN2A1
FLJ14213	TRAF1
RP13-102H20.1	GPNMB
CD2AP	CFAP43
PSEN2	ACLY
APOA1BP	ZNF600
LOC646627	LOC105379566
FCGR1A	LOC101929240
TMEM71	LOC105375434
MTBP	LOC107983998
OR2L13	WDHD1
BCAS1	DOK7
AHCTF1	HLA-A
EFNA1	OXLD1
CRISPLD1	GAS2L3
FYB	SRGAP2B
SHC1	TESMIN
RFX5	YEATS4
ZHX1	RGMA
FAM19A2	RBL1
KY	COL4A2-AS1
NUCKS1	NEK5
ZNF496	HSPBP1
SMYD2	RHBDF2
RD3	LIN9
IRF2BP2	ANKRD18CP
PPP1R9A	MPV17L2
DDX59	ZNF525
MBNL1	COG2
S100A7	RNF144A

PIP5K1A	DCSTAMP
LOC729026	LOC107986710
SETDB1	LL21NC02-21A1.1
UBE2W	LOC646762
PPM2C	EXOSC9
ZNF364	LOC100505715
LCE2C	NDC1
CADPS2	EPHX4
C8orf76	EMC3-AS1
FMN2	ORC6
YOD1	C20orf27
SELENBP1	WHRN
GLULD1	TMOD2
KLHL12	RFC4
GUK1	LOC101927345
RBMS1	CBR3
SLC7A13	MIIP
HLA-G	FBF1
SPRR2D	PDZK1IP1
TNFSF18	HSPB1
NRXN1	DNASE1L2
JARID2	LINC00205
RIT1	XPO5
CHRM3	TMEM67
GSDMDC1	HAUS4
TCHHL1	SLC35B3
SPAG1	NABP2
C8orf47	ZNF324B
RAB22A	TGFB2
NKAIN3	CENPU
TAF2	SF3B4
CA10	ZNF775
ENPP4	ADAM15
RIPK5	ZNF503-AS2
FAM83A	MESP2
FCGR2A	BORCS6
KCNMB2	GJA1
IL1RAP	SNCAIP
C8orf32	LPCAT2
GPR39	ONECUT2
LOC51057	CHEK2
PFKP	ACE2
SCHIP1	LOC107987264
BPNT1	NOL12
BTN3A1	LINC00638
C2orf47	LOC101929140

COX6C	SLC5A4
PRPF3	TMPO-AS1
ZNF341	ZWILCH
DEDD	TRIM31
GRM8	SLC52A2
RICTOR	YPEL3-DT
SLC24A3	ANAPC7
BBS9	HOXB-AS3
OTC	HAGHL
ADORA1	LOC107984567
MGC4473	NPNT
RAB3C	SUCO
EGFR	DZIP3
C1orf56	HOPX
ADAM22	ZNF7
OPTC	PDXK
PYCR2	ORMDL2
NCKAP1L	TXNDC5
SDHC	ZFP30
SPRR3	MEOX2
SLC9A2	HSPB11
HNF4G	CHTF18
ASPH	GSDMD
ITGA10	DCAF16
WDR49	CLIC5
HYDIN	RNF157
LOC441376	H1-10-AS1
UBE1DC1	IQCG
PDYN	ZNF208
NSL1	LOC401261
CACNA1S	CCT3
CTSS	SGO1
SLC2A13	DROSHA
TAGLN2	LOC105375798
PDE11A	FAM72B
ZC3H11A	ZBTB9
NR1I3	BLOC1S5-TXNDC5
NUAK2	RALA
HSPA6	BRAT1
PHACTR1	MVB12A
C1orf132	MINCR
VPS41	LRRC77P
LOC339400	GALNT10
HIST1H2AH	LOC105379854
SEMA4A	SRGAP2
FBXO28	GNB1L

TMCC2	RCN2
ELMO1	DCDC2
VRK2	TMEM50A
KCNJ9	ZKSCAN4
IER5	SRGAP2D
EXOC2	UBE2S
PCTK3	WWC3
WDR42A	DDIAS
CRIM1	ZNF222
RAPGEF3	GSK3B-DT
RGS13	NCAPD2
C7orf10	BPGM
DNAJB8	SCN4B
TMEM74	HHIPL2
SSR2	DTNA
WDR64	LOC105375728
UHMK1	EZH2
MAL2	PALD1
AMPH	ZNF225
AMMECR1	PDE1C
BTN2A1	TCF3
MAGEA8	SLC51B
C1orf85	LOC101928188
IKZF1	G6PC3
PARD3B	NETO1
GPR37L1	SLC38A11
KMO	TMEM253
TRIM46	PES1
PPP1R11	ZNF174
AR	ZFP62
TLR5	FBXW10B
LY6K	FAM228B
ZNF670	DPY19L2P2
CPNE4	ZIC4
LOC340184	CIP2A
ARHGEF2	CEP97
TNP1	FAM91A1
TBCE	LZTR1
SULF1	ASF1B
ERC2	SAMD13
ARF1	YPEL1
EFNA4	CEP89
FLJ23049	SPATS2
C1orf66	GTF2IP1
ENAH	CSPP1
TMEM65	LOC102724542

PGLYRP4	NRF1
PDE1A	BTN3A1
THBS3	MAPT
ADAR	ZMIZ2
C1orf182	SNRPB
CEACAM21	SPATA33
SUSD4	C1orf74
TATDN1	LINGO1
IARS2	TESC
TXNIP	ATP1A2
KCND2	MMS22L
KPRP	ZNRF3
NDUFS2	CEP250
PRDX6	STX1A
S100A10	TMEM209
DCX	ARPIN
COL21A1	TRMT6
GREM2	CS
STXBP4	KDM4D
SLC41A1	EIF3H
NRBP2	RAD51
MGC39715	LOC107985034
C1orf186	NRAS
RHBG	FAM220A
ACPP	FANCD2
MTERFD1	EPRS1
PBXIP1	IL12RB2
MYST2	PIGU
DGKK	NCK1-DT
RAB3IP	MPPED2
CNTN2	SLC39A13
FLJ45872	IFT81
RC3H1	STARD3
COL20A1	BCAS4
AFF2	IGSF21
C1orf51	BROX
ANKRD46	KL
KIAA0460	HSF1
OR5U1	FGF13
WDR67	RNASEL
ZFAND3	ZBTB7C
CHRDL1	TFAP2A
RORC	CACNB4
LRRK2	CASP8AP2
SCAMP3	REXO5
LOC646168	TRAPPC9

AHCTF1P
NPR3
RNF2
KCNH7
ECOP
PTPN7
POLR3C
CNIH4
TARBP1
NNT
C8orf57
IQUB
PELI1
MYBPH
NUDCD1
FCRL2
MDS1
KISS1
NEK2
HTR2C
TRIM17
NIT1
SYT11
ANKRD30A
EFR3A
FBXO43
LSAMP
MTSS1
C6orf85
WISP1
TM7SF4
PTPRT
IMPA1
C1orf75
SPAG16
CREB3L4
RBM8A
EFNA3
FLJ42969
MYT1L
OR6N2
C1orf58
TRHR
PDE1C
COL5A2
KIF14
FLVCR1
LOC101930100
ZNF30
LOC107986126
P4HA2-AS1
ANGPT1
SLC25A12
TCEANC2
L3MBTL2
TSPAN15
RHNO1
LOC105377507
MZT1
BMS1P17
PLP2
SMPX
HASPIN
GPRC5B
RBM42
HGH1
NTAQ1
TCF15
SH3PXD2B
C20orf96
LOC105371763
INKA2
TRIM11
PBX2
RFX8
GIHCG
RNASEH2A
ARHGEF15
TMEM267
SUV39H1
CHODL
ACTG1P25
IFT52
CDH24
COQ7-DT
HNRNPD-DT
CYB561D1
DPY19L2
ZNF676
TM4SF18
HCG18
TRIM3

OSR2	LOC105377265
RCOR3	DUSP18
MYLK	TEAD2
TDRKH	VASH2
SPRR1B	C17orf80
LPGAT1	SNX27
S100A8	B3GALNT1
KIAA1833	PDCD2L
SPATA16	C20orf204
hCG_1814486	SLC13A3
S100A11	PLCD3
IGSF1	HEXA-AS1
PTDSS1	C1orf216
PUF60	TP53I11
LOC286189	MLLT3
HSD17B7	DOC2A
LOC285954	CCDC85A
UBQLN4	GSTCD
MOBP	LOC107985544
CCL28	LOC107984421
MBOAT1	NEK3
PARP1	NARS1
SH3YL1	ORC1
RPESP	MCM7
YWHAZ	BORA
PID1	ZNHIT3
C1orf96	CA12
MAD1L1	MID1IP1-AS1
LOC200383	SNX15
ATF3	FBXL6
OPLAH	LYPD1
KIAA0196	PTPRG
SLC44A4	BSN-DT
LCE3E	EFNA3
TRPA1	TCF20
MYOC	CLUAP1
SMG5	NUDCD1
B4GALT3	HRAS
UBE2T	AP1M2
LRP2	ZNF793-AS1
RNF139	ACTR5
PRL	NUP107
CHRAC1	SEPTIN8
DISP1	VEGFD
CNGB3	ZNF691
YY1AP1	PLCB3

PLCXD3
GML
ZFAND1
PRKAB2
ACTA1
FLG
CDC42SE1
ARID4B
FAM20B
SRI
TDGF3
UFC1
SEMA6C
G0S2
CTNNA2
GHR
GDAP1
FBXO4
KCNK5
PPFIA2
TAS2R40
LHFPL1
LOC730574
TAP1
ODZ1
MKS1
DTD1
TMEM117
SNX27
C5orf17
ZNF659
WWTR1
DHRS9
ZBTB37
POLR3GL
ANKFN1
CDH19
MGC29891
ABCD2
THBD
PYGO2
SLC23A2
C1orf101
CHIT1
ABCF1
DEGS1
MAFG
DBN1
TRIM16L
DPP4-DT
DLL4
CRIM1
PXYLP1
TACC3
CENPX
ZNF223
NUP62CL
P3H4
VWA7
CASTOR3P
ABCA7
CASTOR2
LOC112268450
PAQR8
PRIM2
PRTFDC1
NDRG3
CENPK
CORO1B
ARHGAP11B
PTN
KRTCAP2
NUDT1

NPR1
SLC45A4
GPD2
BNIPL
PABPC1
APH1A
TSPAN7
UNQ6975
FSHR
NDRG1
LRRC4C
IL1RAPL2
ARMC10
S100A16
C1orf192
SLURP1
ABCC3
TP53INP1
MEP1A
ANKRD5
ZNF187
FLG2
TRAM1
VSIG1
SF3B4
HSFX1
GOLIM4
TACR1
ZNF648
CRABP2
ABCB10
TMEM164
CAMK2B
GPR20
MCM3
SRGAP1
ANKRD15
EBAG9
LOC642587
NUAK1
TNIK
SLC45A3
FNDC3B
HAPLN2
UTX
SLC27A6

TARS2
HCN1
LOC200810
OR2T2
KIAA1324L
LOC149134
KIAA1303
RHAG
CPNE3
CLASP2
LRP12
SDPR
EPHX1
LY6D
TTC25
PFN2
PAQR9
CRISP1
IL6R
MLLT11
EIF2C2
ABRA
SPRR2C
SPRR4
C5orf33
PSMB4
SV2A
DHX9
LOC286109
DMD
C7orf53
C1orf77
NSBP1
DGKG
OR11A1
PPFIA4
CST9
GINS1
ZNF695
PXMP3
MGAT4A
C20orf72
PFDN2
THNSL2
GARS
GOLSYN

DYNC1I1
CACNA2D3
FDPS
PASD1
HHLA2
GABRG3
INTS3
ZNF521
SEMA5B
KCNK1
DAP3
THEM5
C1orf43
LCE2D
PPHLN1
WDR26
ZFP41
COG5
DOCK4
OPHN1
TMEM132E
AFF3
DKFZp686L14188
C6
RP1
ENPP5
EGLN1
FLJ16478
GBA
KPNA4
NOTCH2
KCNMB4
ACMSD
TCHH
TPD52
RSPO2
CHCHD3
CAT
KRTCAP2
SEPT9
S100A13
LOC339529
BATF3
NUP133
SKAP1
HDX

SCN7A
ATP6V0D2
FLJ44451
RAB11FIP4
GPR89B
MAF1
FAM19A4
LRRC16
MSC
BMP5
C1orf124
TANC2
FH
PIWIL1
RUNX2
DKFZP564C196
PDE7A
NEK11
USP30
SLC17A1
DAAM2
NRCAM
C1orf57
PMS1
DPP10
ISG20L2
DENND4B
MED12L
C17orf32
FHL1
ZNF281
SPEG
TRIB1
PCSK2
HAX1
AGTR1
PTPRZ1
PMP2
NDUFB9
DST
AOF1
CXorf22
PKLR
C1orf54
LOC149157
TEDDM1

FAM84B
EDARADD
NEB
KIAA1843
CA14
BBX
CABC1
PLEC1
LOC285965
RTEL1
COLEC11
LRFN2
ATP2B3
CPS1
GTPBP10
SLC13A1
CHD6
NSF
BMX
C1orf67
TBC1D5
PDZK1
MGAT5
RBP2
TBC1D20
KCNJ16
VARS
OR9H1P
HBP1
LOC348801
WNT9A
OSMR
PRKCE
EFCAB2
C1orf142
ARHGAP6
SI
ABCA5
IL17F
NQO2
USP49
CPNE8
DYSF
BRPF3
POGZ
ZNF238

ARID2
PFKM
LRRCC1
CPA6
SLC10A5
UBE2Q1
CDKAL1
LCE5A
ANKIB1
SUPT3H
EGFLAM
WNT3A
PSMD4
RAG1AP1
PHF20L1
S100A5
HDAC8
LRRC6
ARL5A
ROBO2
TIMM17A
OLA1
ABCA10
GABRA3
PRIM2
UBAP2L
KIAA1383
SLC13A4
C1orf74
IGHV1-69
TJAP1
NAP5
LOC286190
KLF10
DGKI
ZNF16
LYSMD1
XDH
ANP32E
ADSS
GRIA3
KALRN
USP21
CUGBP2
FAP
LAGE3

ENY2
GCNT2
SYCP2L
PHACTR3
ELK1
LYST
RHBDD1
ZSCAN16
CP
TXNDC13
ATP6AP2
C1orf156
ARID5A
C1orf131
HIST1H3G
FOXP2
UCHL5IP
TLR8
ACAA1
FGF12
ARV1
BRE
FHIT
RLBP1L1
SLC17A4
LOC647107
SCN1A
C1orf157
FCAMR
FAM49A
CLK3
SNTG2
OR2M4
AHR
OR4D2
TFAP2A
ALK
DPY19L1
PTGER4
EHMT2
DNAH5
CYP7B1
IVL
EYA2
PRKCQ
PMVK

MYLIP
CA3
LOC284702
C20orf46
PART1
TEX11
CMAH
BOLA1
KLHDC9
RAB25
SMARCA1
ITGA4
KLHDC6
LBR
ETV1
SKIP
VEPH1
EEF1D
SLC8A1
FAM82B
GALNT1
DGKE
CHCHD6
ACBD3
PDE7B
LIN9
EDA
CRTC2
BTBD9
HIST1H3I
KLHDC8A
PLCB4
DNER
LOC285819
ITPKB
LOC285778
CEP70
KIAA0319
MAGI2
GULP1
TLK1
P2RY10
TMEFF2
C1orf121
LAX1
FZD1

TPM3
ULK4
LMOD2
THSD7B
CD99L2
GALNT14
SMC6
C10orf97
REC8
NAB1
TMC2
PSMC2
FABP5
PDZD2
TUFT1
C20orf103
JTB
GFOD1
C8orf31
IGF1
PI15
GOLGA4
SIRT5
FLJ42562
JMJD4
F13A1
C2orf34
MPN2
C2orf13
TXNDC6
MAL
C8ORFK36
MYT1
ZNF692
HEATR1
DAB2
CGN
PRLR
ZSCAN23
LHX4
PLXNA4
SNRPE
C10orf68
C1orf71
OR2T1
TNFRSF21

NIPBL
RAPGEF4
C20orf166
LCE1B
TMEM108
SLC26A3
ZNF707
PLEKHO1
DMTF1
C6orf12
FAM83H
BAI3
RFX4
DNAH1
RP4-691N24.1
AKAP4
INPP4A
RBMS3
GAP43
GPBAR1
TRIM11
FLJ25439
SRPK2
SRD5A1
SPAG4L
NF1
CA1
HRH3
XK
GFRAL
TBR1
EPPK1
ACAD11
KIAA1604
C1orf100
ZNF533
TRIM58
ZC3H3
COBLL1
FAM110B
APOBEC2
FLJ43752
SLC41A3
PGLYRP3
RXFP4
HIST2H2BE

SHE
NOTCH4
SLC41A2
CA8
STK39
CERKL
C6orf32
OXCT1
GOLT1A
TFAP2B
STOM
LARS2
PDCD10
LCE1C
UGT3A1
GABRA2
CDH6
SLC16A2
NID1
LY96
CDCP1
SEPP1
ITGA7
LZTFL1
GOLPH3L
ZNF449
NFKBIZ
KCNK16
CYSLTR1
IMMT
CHM
LOC149832
LOC26010
DOM3Z
RHOT1
BCL11A
MGC13057
CCDC141
C8orf55
MPP2
TR2IT1
ZNF804A
RAD17
GLI4
FGF13
C6orf86

KIAA0748
CSTL1
RGS2
TERF1
LOC729085
SOX4
DIRC3
HOXB5
TMEM166
ERBB4
TSNAX
MTR
GLI2
OR5BF1
ZNF133
ITGA1
RNASEH1
WDR60
RNF43
SNAP25
GRM7
MGC16121
PTP4A3
LYSMD4
TRMT12
MTMR11
SGK3
ABCA9
C2
NENF
WIF1
IFIH1
MYO1A
E2F5
C18orf17
WIPF1
FLJ22536
LHFPL3
SPAM1
CCDC85A
TAAR6
IL18RAP
RUSC1
CDH2
UBE2DNL
NLRP3

TIPARP
GLI3
SLC39A1
EXOC6B
SCRIB
DLG2
RBM33
PLEKHA8
FRMD4A
NRP2
PAX3
KRT222P
RECQL4
TAPBP
KIAA1715
SLFN5
TRIM55
GNPAT
PRKCA
RAB13
AZI1
NXPH1
PILRA
RDH10
PSORS1C1
FLJ21986
CASR
LY6H
ZDBF2
GPR137B
ABCA12
NSUN3
POLN
ADCYAP1R1
GJA8
HPS3
CA2
GRM4
ZNF184
DHX35
TMEM63A
ZNF687
C6orf52
TOM1
PNMT
C6orf65

LYPD6
E2F6
WFDC8
CHIC1
RAB2A
GPATCH4
CDK6
GALNT9
OPN5
XKR4
GPLD1
PDK1
EXOC4
XKR9
EIF5A2
MYC
UNC45B
LOC348808
DTNBP1
FBXL13
SRBD1
TNFAIP8L2
STXBP5L
BRD2
SNRPC
CDH4
NACAP1
DSCAM
C12orf42
FLJ10489
LOC645676
CAPSL
ABCA8
C8orf43
RPL8
LOC646324
MSN
MCL1
LOC643517
B3GALNT1
MRPS23
TBX5
TTC35
YIPF5
TASP1
EDN1

MTFR1
TRIM39
LOC284576
SYT13
KCNK15
HAO1
ARMC4
C3orf26
KIAA0133
CCNB3
PDE4D
KIAA1688
KCNK17
ZDHHC15
HECW2
UXT
LOC284757
CTNNBL1
ZHX3
STXBP6
GRB2
OR2G2
ETV5
FLJ36032
LOC130576
MGC4859
ZNF638
RAB40A
WFDC5
KCTD20
PAQR6
SYNPR
UNC50
TYR
C3orf28
GALNT11
COL11A2
CRCT1
SNTG1
MAPK14
MUT
TRNT1
OVOL2
ZNF572
KRTAP4-10
SLC45A2

SLC1A2
ERO1LB
NDUFS4
ZNF75
COL3A1
RYK
BMPER
XIRP2
TXNRD3
MMD
MRPS21
CYLC1
EIF3E
RSAD2
E2F3
IL1RAPL1
NHS
ORC5L
TTLL4
CDH12
AACS
POU6F2
TMEM16K
ATP13A4
ST3GAL6
KIAA0427
MEX3A
ARPP-21
RIPK1
C20orf177
PSMD14
NDP
LASS6
CLCN5
ZCCHC16
GLIPR1
SHROOM4
OSR1
MCART6
CHRNB2
GADL1
CHN1
ATP1A3
ITGB8
PFDN4
C6orf105

HSD17B4
SLC7A14
CBLB
POLS
USP6NL
TTC21A
OXSR1
COL4A6
NUP153
NNMT
SMAD3
ENSA
SOX6
ANKRD34
TNF
ALCAM
C14orf23
TMLHE
MAPBPIP
SATB2
BET1
TXNDC5
MGC42090
DNAI2
PAQR8
DACH2
LEMD3
IHPK3
SCN10A
LOC441383
PCDH11X
MCM2
C20orf4
PANX1
TMEM100
PAIP2B
CHRM2
S100A9
SOCS5
PGS1
DEFB126
PLXNA3
ZNF250
OSGEPL1
DPY19L2P4
LSM8

BGN
KLHL4
NRP1
NSMAF
LOC339874
RIOK1
C2orf55
MAP3K7IP3
C1orf35
TSTA3
C6orf10
PRDM14
RTDR1
C1orf111
PLSCR4
PDZRN4
IGLV2-14
VPS28
HMGCLL1
EXOC8
FARSB
IL1F8
SNRPB2
TMEM45A
DLD
AKR1C3
MAML2
ENOX2
TIGD5
MYO3B
CCT6B
LEPREL1
C20orf71
CACNA2D1
LOC148638
STK4
PLCL2
ITPR3
POLA1
ARL8A
MGC42157
TPMT
GIMAP5
ADAM23
CHD7
SDCBP2

STK35
AUTS2
PON1
SP100
ZNF650
NDUFA12L
PSMF1
VIPR2
RHPN1
LOC401463
CTCFL
ABCB11
CENTG3
LOC130940
TRIM40
DTNB
ZNF800
ZNF451
NAPRT1
NOSTRIN
MRPL35
PLS1
LMBRD1
COPS5
TMEM182
KIAA1486
GLP1R
FRMD7
MRPS14
CUBN
CLRN1OS
PRKAG2
ST7
FMR1
LYRM4
ATP2B1
DZIP3
FRMPD4
NOX5
MBOAT2
MSL2L1
CST8
TMEM14A
WSCD2
VN1R5
USP32

EEFSEC
ZNF311
SYT7
PFKFB1
LOC221710
SLC16A7
TMEPAI
DUSP22
DCBLD1
GALNT5
TOX
TM4SF20
HECW1
ANKS1A
FN1
MAP3K1
ABHD12
GPR115
MDGA1
RPS6KA6
PCNA
BZRAP1
FLJ13231
SLC6A4
NXF3
RANBP9
KCNV1
PTPN4
RPS23
RARB
CREB3L2
LAMB1
ABP1
CPSF3
DDX1
C2orf48
PVRL3
LOC286186
XYLT2
ZFYVE20
SELV
RAB5C
KRTAP9-4
UBD
CSAG2
RREB1

MYO1B
PCSK1
ARX
MAPRE2
CPNE5
HSF5
ZKSCAN4
LOC286052
IQSEC1
TBX20
TNS3
LOC91948
LAMB4
SCGN
SLC17A3
LOC286442
MFI2
CACNA1D
PTPRR
BIRC7
TBC1D19
TRIM27
C6orf106
HLA-DPA1
LUZP2
ITM2C
SLC27A3
C3orf21
BPHL
MEIS1
AGR2
NEU1
VBP1
PIGF
PLCL1
GPR112
LARP6
DOCK3
RNF182
ZSWIM2
CD36
FATE1
TOP1MT
DFNA5
TBC1D7
C7

SSH1
PARP10
BZW1
ARP11
ENTPD3
FSIP2
PRELID2
THRB
RPS27
LOC92270
JPH2
FLJ43692
ARFGEF1
C7orf51
CCDC132
DVL3
LST1
ARL15
MINA
SOX3
ING3
DPP6
TMEM110
PHLDB2
MTM1
NEBL
PIN4
KIAA1267
DCDC2
C8orf45
LMBR1
RSBN1L
ZMYND11
KCNQ5
BMP6
CENTG2
KATNAL2
PGAM2
ANGPT4
CCDC14
FGF10
LYCAT
BAZ2B
HYAL4
ACTN2
FAM46C

DEFB110
CRH
NCOA6
GSPT2
LOC441383///ZNF252
LOC151171
CD83
FLJ34048
LBA1
TRPM8
C6orf136
COX4I2
POFUT1
KIAA2022
PTPLB
CALCOCO2
CABLES2
LOC728730
HNRNPU
CNTN4
GGPS1
TRPC1
RFC4
PRICKLE3
UGT1A8
C1orf80
CCT7
CXorf40A
DEFB112
RUNDC3B
FGD2
AGPS
LOC151121
ZNF337
RENBP
LOC286187
IWS1
tcag7.1314
PAH
CXorf62
IL20
EME1
SLC15A2
LOC286467
IGF2BP2
HRASLS5

HIST1H1A
COBL
TREML2
MAK
FLJ41649
PIR
GNG4
SPOPL
CXorf59
C6orf142
CXorf27
MED10
RTP1
CDH18
PEPD
MIXL1
RNF8
PPP1R16A
HDAC4
CALCR
DEK
KIF4A
EXT2
RFFL
DTWD2
MRPL30
VSX1
ARMC8
FXR1
C3orf55
SLC35B3
ADCY1
SQLE
ITCH
STEAP4
FXC1
LEFTY2
SLCO4A1
CSAG1
GPR98
CNTNAP2
CALCRL
DKFZp667G2110
DKFZP564O0823
TMEM106C
C6orf64

LCE3D
C7orf23
CPOX
SIRPD
BTN2A3
RNASEN
LOC727993
TMEM28
GHSR
DSP
NAV2
SNX19
MCCC1
7A5
GFM1
LOC157381
GPAA1
MFSD1
PRX
PDHX
BIRC6
BCL6
SGEF
LIFR
PYCRL
SLIT3
DLG1
FLJ21062
MERTK
DLEC1
CTNND2
FLJ26484
KIF5C
WDR75
ZNF517
FAM36A
FOXP3
FAM62B
FETUB
IMPAD1
TFEB
COMMD5
TH1L
BDNFOS
ALDH1L1
KIAA1946

BBOX1
ADIPOR1
ZNF711
GLB1
GAS5
C6orf138
ZNF452
ZNF574
CRISP2
SEC23B
MRPL3
NPAS3
ZNF7
ACADL
PPP1R3A
C2orf3
ARMCX5
RARRES1
MTRR
TEX28
CALU
C14orf21
AGBL1
KRT36
GPR158
BHLHB9
PLP1
SUCLG2
ATAD2B
DNAJC13
MPP6
ELMO2
TTLL9
ACSL3
DYSFIP1
FAM83D
EEF1E1
PLCXD2
SCCPDH
UBR2
TGM2
ABCF2
LYN
FAM79B
LRRC58
TRIM15

IKZF2
NOL7
NAG
WDR52
DDAH1
SLC35F1
CCDC3
C6orf130
ACTL6A
FLJ11236
MGAT4C
GPR23
YTHDF3
BAIAP2
NESPAS
RNF144B
TRIM6
EWSR1
ITGA11
LOC158960
HGF
MAP1D
CLLU1
TMEM131
KCTD8
HLA-DQB2
TMTC3
ARHGAP26
EPC2
DDC
CSPP1
C5orf28
IQCA
SRP9
OGDH
ATP11C
FYCO1
CLASP1
CPSF4
FLJ39349
C1orf69
UNC5CL
SCN2A
RAI14
BCAP29
NRK

LRRTM4
ZNF366
MGLL
SERPINI2
NCOA3
DIRC1
TSHZ3
SPATC1
SEMA5A
MTMR1
HDAC9
EDIL3
KIAA0241
ANKRD55
LOC399947
SERPINI1
RBPJL
PLD1
POF1B
AKAP14
COL29A1
STRN
DRD5
BOP1
ARHGEF6
KIAA1462
ECEL1
TNNC1
LOC440983
NMNAT3
COX7A1
B3GALT1
KIAA1706
GSTA4
WIPI1
COL4A5
NAT13
ZAK
ZNF623
OSBPL10
BXDC2
ALDH1A2
MASP1
TUBB2B
TXNDC3
ARHGAP12

IL31RA
SUMF1
LOC285577
TRIM42
ACACA
FLJ25076
MGC61571
KLHL34
LRRC28
GCG
STARD3NL
TMEM163
C3orf44
ASB15
SIRPG
LY6E
ASB1
RPGR
ZNF669
PENK
RHOU
LOC157503
NDUFA5
ABCB4
MAP7D3
CARD6
TANK
HSPB3
MARCO
BAT2
PPP1R1C
TBC1D8B
DIS3L2
CSTF1
SATB1
PLEKHB2
FBXL4
DPP4
BTN3A3
NFKBIL2
MYO3A
SPANXN2
SUB1
KIAA0226
OSBPL6
C20orf174

DPCR1
CPSF1
LRRC24
COL6A3
CDH10
NETO1
SLC26A4
SDCBP
ANKAR
ZKSCAN3
TARP
SERPINE2
LANCL2
CHL1
FLJ16686
GDAP1L1
WDR87
LOC440104
GSK3B
ARMC1
HDAC7A
GCET2
ANTXR1
CYC1
OPRK1
PEX6
TNXB
TNS1
FKBP9
ZNF34
WT1
KIF23
ARL14
KIN
AYTL2
DUSP19
DNMT3B
CLPB
TMEM14C
HLTF
ADD2
ABCA13
SCN9A
POLR1A
ADAM17
ATP1B3

IDS
ALS2CR11
PCTP
SEL1L2
SPATA20
PPP2R3A
SH3KBP1
ZNF165
MGC87042
KLHL13
FLJ31818
XRCC4
ISL1
GLUL
ZDHHC11
CHN2
AMACR
TBC1D16
CCDC58
CROT
SLC38A4
CYP11B1
CLDN12
HPS4
GPR1
DNAH7
C6orf1
EPB41L4A
CD96
OSBPL3
STARD8
COL6A6
UNQ846
FIGNL1
SYPL1
PPP1R9B
CPEB4
GUCY2F
NXF5
IFT57
ZDHHC9
PROS1
MORC4
EVI1
LOC339788
EPAS1

ADARB2
EGFL6
CACNA1G
PKP4
WFDC12
SCIN
LOC728194
THOC5
TENC1
WRNIP1
SLC9A7
KIF13A
KCNN2
CXorf57
SH3BGRL
RPP40
COL28A1
SLC25A36
TMEM189-UBE2V1
TNMD
WFDC10B
LEFTY1
HEATR6
BAT5
FLJ44815
LOC285636
FAM83B
B3GNTL1
GEFT
IQCB1
C1orf115
IL13RA2
PCLO
ZNRD1
45720
FAM134B
TP53AP1
SYNGAP1
LOC285949
UBQLN2
GRIA1
TUBB
CDH26
AGXT2
GOLPH3
PTPRG

SLC33A1
ZBP1
TTRAP
ZFP91-CNTF
MRPS33
CBFA2T2
ENTHD1
ADRA1D
C6orf107
MTCP1
HCLS1
TAG
IMP4
LOC653110
PLAGL2
CHST11
TBC1D23
NR1I2
AADACL2
ADHFE1
TSGA14
SDK1
LRCH2
NAPB
OSTN
LOC285768
NOS3
MTMR8
MAP4K4
SLC26A8
ST18
FAM107B
FLJ46688
BEST3
WDR7
SEMA3D
BTN2A2
C1QTNF3
SYTL2
CNKSR2
MAPK15
GGTL3
FLJ36157
CAP2
LOC727973
RAB23

KYNU
TSC22D3
PHC3
FBXL20
SLC38A2
ZCWPW2
PAIP1
MRPS22
ABTB2
F8
PCMTD2
CDK5
RALY
HSF1
FLJ42117
GMPR
RND3
TGM3
PAX1
GSTA3
SLC6A14
FAM82A
ZBTB22
STAG1
HLF
RP6-213H19.1
FOXR2
RNF152
HNMT
ZFP161
HTR5A
ARL6IP6
MYH7B
KSR2
TFPI
ARD1A
FLJ90723
TCTE1
RPA3
FAM130A2
FOXP4
SNX10
ADCK5
PPP1R12A
IL8RB
TUBB1

TMEM70
VSTM2L
C6orf89
LUZP4
FLJ27502
FAM19A1
CDH7
TDRD6
TGFBR2
CENTD1
CSF2RB
TRERF1
SPSB4
SLCO2A1
TP63
FAM50A
PAK1IP1
PITPNB
OR2H1
GJC2
ADAMTS20
POT1
AGT
BRCTD1
CXorf31
AP3B2
MYLC2PL
KIAA0828
CYP11B2
DOCK10
CPB1
PSKH2
CLIC1
FLJ44838
SPTBN1
TSSC1
ANKH
CADPS
TTPA
RNF128
MLN
BRCC3
HLA-DMB
FASTKD2
GNAI1
UTS2D

hCG_1806964
ZNF192
SCN3A
KCNQ2
APOOL
SAMD9L
RANBP3L
PLA2G7
DCC
DHX15
SGPP2
THEM2
TMBIM4
HIBCH
PITPNM1
CAB39
SCRN1
CXorf41
ATXN7L1
PSMA2
BPIL3
PSCA
FABP1
CHST8
PTER
TRIM38
SLC17A2
TRPV5
SNAG1
ITPR1
CCDC46
CCNY
GEMIN8
CLDN16
BTK
GREB1
CEBPA
ARSB
SERGEF
PTPRN2
CRHR1
KCNB1
PARP9
SLC25A39
DHX57
LAMA2

C3orf17
TERT
ATRX
MAGEE2
LOC729179
LOC154907
PREX1
EFHC1
GLO1
ATP13A5
NLRP13
NEK10
EIF5B
BRAF
SLC25A27
ATG7
PLA2R1
GATAD1
HMGN4
LIMCH1
RNF144A
PRPF18
CACNA2D2
MRPL2
CYB5R4
COL2A1
NXPH2
NCOR2
CDR2L
EGFL11
KIFC1
IL1RN
SFMBT2
RGAG1
KLF14
SNPH
GGH
CCDC88A
TINAG
TSHZ2
GAB3
RP11-217H1.1
GRIK5
C18orf12
SESTD1
TOX2

LGR5
LRRIQ1
VEGFA
LOC205251
GPR15
TSP50
ALKBH4
DGKB
B3GALNT2
ZDHHC3
ALX1
ATIC
ELOVL2
FKBP5
MME
SLC25A19
NFYA
C8orf33
CHST12
FLJ43663
SGCE
PIP4K2A
GPM6B
GPR133
ABCA6
KCNH2
MOV10L1
CDC5L
RTN4IP1
PDIA5
SMARCAL1
B4GALT5
RNF39
CUEDC1
OR12D2
KRT32
SIRPB1
KCNC2
FBXW8
MAGI1
DTX1
KIF15
CHSY-2
PTN
FARP2
KCNH8

FLJ23861
EPB41L2
FLJ44968
TRAM2
MGAM
BCHE
TAF8
NAP1L1
SNX8
MED17
NELL1
SERPINB6
TGFA
TMEM5
GRIK2
RBM23
PAM
MID2
AKAP9
MAP6
ACTR8
FLJ30594
PFDN6
ITGAV
CREB5
ACE2
PVRL1
NDRG2
RIPK3
TRPC2
RNF5
BLACE
ROBO1
NT5C1B
KIAA1524
CCDC93
RASSF2
PRPF40B
HELZ
ZNF197
PPP1R16B
IGBP1
PI16
SEPT10
C5orf22
CALCA

TIMP2
EN2
RTN4
PIK3R1
C20orf77
PDZD4
PIK3CG
TBXAS1
RPRM
PLXDC2
SPATS1
LYK5
ITGA3
SST
MICB
DBNL
CACNA1B
C3orf39
MRS2L
KCNJ2
TTC21B
GSTA5
TUBD1
DCN
VSNL1
NAP1L2
MPP7
ADCY6
AZI2
FXYD3
HSPC159
RNF32
PROKR1
LOC130951
HIST1H2BN
STK17A
TMEM168
LOC285216
AHRR
NR1D2
TMEM16C
C6orf128
MKKS
SSFA2
TMEM16F
FAM59B

MAGEC3
KIFC2
CNTN5
TBCD
MGAT5B
HACE1
PLSCR1
DSG3
TMEM103
EDN3
DTNA
LOC284260
CHRNB4
CYBB
ZFR
NRXN2
CD47
ID4
PLXND1
FGD5
KRT7
LOC285191
WWC3
FAM19A5
FLJ20160
EFNA5
TSHZ1
ANKRD44
ZPBP
ZEB2
ARMCX4
SPINK5
ITGA9
FMR1NB
FLJ37357
BMP7
CSH2
KCNE1L
AFAP1L1
FAM70A
SLC39A4
MOSPD2
TRA@
AOAH
ANKRD28
C2orf18

ADCY2
RAB17
SIPA1L3
LOC441009
CHMP2A
C11orf75
POLD2
GABBR1
CDC2L5
PLOD2
SR140
HIST1H2AL
LOC149684
PLAC1
FBXL2
PLSCR2
C2orf46
HIST1H3E
KIAA0087
PRKAR2B
IQSEC2
CD200R1
HCRTR2
TMEM139
SLC1A3
LGR4
BHLHB5
METRNL
BPIL1
MLPH
GPR149
KIAA1166
OR2W1
LOC221442
TMEM140
LOC284294
DOK6
ICK
CCR1
CAGE1
GIMAP8
C18orf1
ZNF536
MRC2
MTX2
CD300C

MCTP1
GZMK
LMTK2
DMKN
TMEM119
SUCLG1
BCAS3
NUDT3
TEX2
LY6G5B
SP3
PNPLA1
NHLH1
GABRB1
HIBADH
MAP2
PIP
WAC
TREM1
SNCB
ROPN1
TSC22D2
CXCR3
FAM50B
LOC349160
LOC647323
SENP7
C18orf26
TCEAL2
VARS2
ZMAT3
PHF6
SPACA3
CLIC2
PHF3
PPP1R1A
MGC15634
CENPQ
NRM
HRASLS
DIDO1
IRF4
PCK1
KIDINS220
RP5-1054A22.3
RHOQ

AMOT
SLC30A1
VCPIP1
TAS2R39
ZNF248
F2
PGM5
CABIN1
BRWD3
ACSS3
STAC
MKL1
IQGAP2
COX19
CBX1
CRBN
PHKA1
FLJ41278
PSD4
METTL8
PGAP1
ZNF318
H3F3A
PTH2R
BIN1
SPOCK1
ZC3H6
SSX7
RAB9B
HTR4
MSH5
SLC25A14
FLJ23172
LOC152118
DNAH11
WNT16
NUP62CL
C3orf35
SYT17
HIST1H1C
GNGT1
AKR1CL2
DGKD
SLC25A13
LAMB2
CXorf48

ZNF789
GABRE
PCDH19
BRD9
LANCL3
ADARB1
GPC3
DCDC5
CDC42EP4
ALDH1L2
DDX26B
GNAS
LYPD1
OPCML
WSB1
ZYX
SLC12A7
SLC38A1
GPC4
TBC1D1
TMEM44
DIRC2
RSU1
ROS1
TAF4
PIGU
ZNF193
FGD1
LOC400655
TFEC
LOC646588
ORMDL1
EFCAB1
TAF4B
TM7SF3
KCNIP4
MYL4
DNAJC10
DEFB114
SOX9
LMCD1
MYRIP
PNPLA8
CRNKL1
C2orf12
SBF2

INHBC
SCN11A
NANP
LOC348751
GAA
CYB561
ZNF277
SERPINB9
GUCA1A
PRRG1
IRS1
NLGN4Y
NELL2
DLGAP4
HDAC5
BOC
GABRR3
SLC9A4
SEC22C
U2AF1L4
FAM115A
OR2B2
BPI
SKAP2
WASL
HUWE1
MRPL12
SLFN12
DMN
BAZ2A
KIF18A
ZFP57
GCGR
NDUFB5
ST5
C20orf59
SNRK
SORCS2
C18orf34
PHF21A
GLS
TPK1
TCEAL1
STAT1
HCG18
LOC285771

PTPRA
LARP5
CUL7
HIST1H2AI
FAHD2B
HMG20A
GNAL
LRRC15
NUDT11
TSGA10
STRA8
NAP1L3
ELOVL7
KLF7
C6orf27
TTLL7
HPCAL1
FAM46D
GRAMD1A
ITGA2
SCG2
SLC9A10
HYDIN2
ACSL4
PGK1
MORF4L1
LOC729852
JAZF1
CCND3
RP5-1022P6.2
MSH2
TLE3
MGC33657
IGSF11
GBA3
ATRN
PTK7
TTC7A
FLNA
MAGEF1
C20orf19
XPNPEP2
RHBDL3
SLC6A18
MAOB
MRPS30

KSR1
45723
WDR51B
SLC14A2
LRRC14
BTBD11
CDCA7
AGXT
PLAG1
C3orf23
MARS
KIAA1648
PLEKHC1
CDKN1A
HTATSF1
C9
RMST
TRIO
CASK
HYPE
MRPL9
DAP
CYP24A1
C3orf53
RGS19
GPR172A
CCBE1
CXCR7
ADA
LY86
REEP5
CPVL
PXDN
RP11-218C14.6
HSP90AA5P
NDUFA12
NSFL1C
MCM10
ATG2A
KLHL29
NCR3
FAM110A
FAM133B
ST6GAL2
ITM2A
TTC15

TRIM44
USP15
LOC285026
FAM133A
FAM96A
CDC42SE2
DIP2C
MTMR12
ELF4
NOL4
ANLN
ALS2
SEMA3C
KRR1
SMPD4
XKR7
MUCL1
PPARG
RASA1
SPDEF
FLJ20323
GRAMD1C
GMEB2
IGSF10
HM13
LOC401312
TRDMT1
PMPCB
RIBC1
RAPGEF6
CARD10
PRDM11
ANKRD7
ELFN2
NCK2
CCL7
MGC39900
CYP3A43///CYP3A5
ZNF767
KIF21A
SFRS10
GIMAP1
TP53I11
RIMS1
BRIP1
STXBP2

RALA
AASS
C8orf46
EEA1
EIF2A
FBXL7
CRHR2
KIAA0355
MAP1B
CARD14
UCKL1
CCDC80
ICA1
SULF2
TAZ
GLB1L
PEX12
HIST1H3J
RABL3
GRIP2
UBE2O
RP11-145H9.1
GCLC
ST8SIA6
COPG2
CXorf42
TM9SF4
SERPINB1
HLA-DMA
FLJ23834
FSTL1
RGS20
FANCB
NR3C1
C6orf151
C17orf46
RUNX1
BRUNOL4
SYTL5
SYTL4
TPRXL
LOC137886
SGCD
FLJ40142
CTDSPL
EGFL8

NSUN6
KCTD16
LOC150763
ODZ2
ZNF532
CHRNG
EHMT1
GRK7
TRPC5
LOC153469
ANKRD13A
CCDC47
SLC9A3
TDRD12
INSC
ZEB1
SLC12A5
MCF2
SLC39A11
MALT1
DEFB119
COMMD2
C20orf42
VPS37B
ALDH5A1
DAGLB
KRT1
ATP11B
LOC646736
SLBP
FAM20A
CYP2B6
EFHC2
CEP290
KIAA0100
SLC4A3
FOXK1
MKLN1

Figure 1C

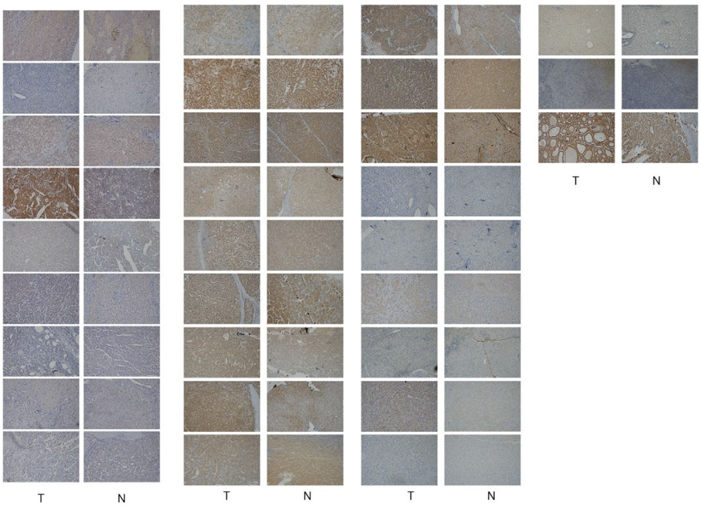

Figure 1D

Normal	Tumor
3	3
2	2
2	3
5	5
3	3
3	3
0	2
3	3
2	3
4	5
4	4
4	4
4	4
4	4
4	4
3	4
4	4
4	5
4	5
4	5
4	4
0	2
4	4
3	3
4	4
0	0
0	0
0	0
4	4
0	0

Figure 1E	si-NC+EV	si-KCNN3+EV	si-NC+KCNN3-OE
Huh7	680	652	671
	692	496	622
	677	668	659
HepG2	si-NC+EV	si-KCNN3+EV	si-NC+KCNN3-OE
	402	397	405
	412	401	437
	398	360	384

Figure1H	si-NC+EV	si-KCNN3+EV	si-NC+KCNN3-OE
tumor weight	0.069	0.09	0.089
	0.114	0.103	0.116
	0.092	0.071	0.092

Figure 1J	si-NC+EV	si-KCNN3+EV	si-NC+KCNN3-OE
Migration-Huh7	321	102	555
	293	107	541
	281	119	569
Invasion-Huh7	347	110	462
	329	130	353
	291	125	451

Figure 1K	si-NC+EV	si-KCNN3+EV	si-NC+KCNN3-OE
Migration-HepG2	323	189	505
	312	178	516
	325	187	595
Invasion-HepG2	345	199	506
	344	200	564
	339	196	529

**Figure 1F**		si-NC+EV			si-KCNN3+EV			si-NC+KCNN3-OE		
Huh7	0	1.037	0.938	1.025	1.068	0.938	0.994	1.016	1.047	0.937
	1	1.603	1.608	1.654	1.671	1.784	1.831	1.483	1.461	1.616
	2	2.387	2.331	2.379	3.108	2.886	2.782	2.330	2.551	2.272
	3	2.928	3.171	3.177	3.457	3.267	3.559	2.663	2.386	2.756
	4	4.167	4.111	4.234	4.378	4.581	4.361	3.763	3.418	3.230
	5	4.109	4.033	4.395	4.266	3.802	4.277	4.610	5.235	4.837
HepG2	0	1.116	1.013	0.870	1.034	1.000	0.966	1.016	0.996	0.988
	1	1.043	1.054	1.056	1.030	1.253	1.167	1.142	1.046	1.065
	2	1.378	1.479	1.248	1.976	1.777	1.328	1.489	1.654	1.679
	3	2.248	2.519	1.924	2.249	2.693	2.429	1.772	1.852	1.654
	4	2.909	3.414	2.810	2.670	2.863	2.689	2.262	2.348	2.483
	5	4.450	4.980	4.492	4.039	3.659	4.504	5.499	4.852	5.430

**Figure1**	tumor volume								
Day	si-NC+EV			si-KCNN3+EV			si-NC+KCNN3-OE		
0	0	0	0	0	0	0	0	0	0
4	0.19	0.13	0.05	0.17	0.15	0.05	0.17	0.11	0.07
8	0.23	0.16	0.09	0.2	0.16	0.06	0.22	0.12	0.09
12	0.29	0.2	0.11	0.25	0.2	0.09	0.28	0.18	0.13
16	0.36	0.23	0.15	0.3	0.22	0.11	0.3	0.25	0.15
20	0.45	0.26	0.19	0.32	0.26	0.12	0.33	0.26	0.16

Figure2A		
Gene_symbol	Peptides	Unique Peptides
MYH9	193	160
MYH10	182	146
SPTAN1	131	1
SPTAN1	131	1
SPTBN1	104	8
SPTBN1	97	1
MYH14	88	74
FASN	83	83
EIF3A	115	115
TJP1	70	69
PARP1	67	67
CLTC	65	65
RBM10	55	50
ACTN4	50	35
HSPD1	41	41
EPRS1	60	60
KCNN3	36	36
HNRNPM	48	48
FLNA	57	51
ENO1	33	31
IQGAP1	59	58
EIF3B	46	46
HSP90AA1	46	30
HSP90AB1	41	23
HSPA5	46	44
GANAB	42	1
HSPA8	41	34
ACTN1	48	30
STIP1	48	48
GANAB	42	1
EEF2	48	47
XRCC6	44	44
UBA1	39	39
TLN1	52	51
HSP90B1	58	56
KRT2	46	33
KRT1	41	37
ATP5F1B	25	25
PYGL	46	37
HSPA4	47	43
LRPPRC	55	55
PKM	37	37
HSPA9	37	36
ACLY	49	49
TNRC6B	40	40

CCT5	34	34
XRCC5	37	37
MTHFD1	48	47
CCT2	36	36
KRT10	40	34
HSPA1B	34	19
SF3B1	39	39
VIM	40	37
PRPF8	53	53
KRT9	34	33
VCL	49	49
VCP	45	45
IARS1	42	42
COPA	46	46
CCT3	33	33
FKBP4	37	36
LMNB1	38	36
TUBA1C	26	5
DOCK4	52	52
EIF3C	34	34
EIF4B	52	52
NCL	42	42
SCYL2	34	34
GAPDH	24	24
CAD	47	47
SNRNP200	44	44
DHX9	41	41
LARS1	41	41
ACTG1	25	9
SFPQ	33	32
YARS1	38	38
TRIM28	29	29
XPO1	36	36
KHSRP	33	27
SND1	42	42
PDIA4	36	36
NONO	31	28
CCT8	35	35
AARS1	39	39
EIF4A1	25	12
CCT7	31	31
TUBB	23	5
SMC3	42	42
IPO5	31	28
PRKDC	58	58
TUBA4A	23	1
TUBB4B	22	1

CAND1	37	34
ALDH18A1	31	31
MYH1	30	4
SMC4	44	44
TUBA3C	22	1
IMMT	32	32
PDIA6	32	32
SF3B3	36	36
MYO1D	42	42
GPI	31	30
EIF3L	36	36
HTATSF1	29	29
ATP1A1	30	17
HNRNPU	35	1
MARS1	29	29
MYH4	32	8
HNRNPU	35	1
ATP5F1A	36	36
TRAP1	36	36
DDX3X	35	34
RUVBL2	28	28
TUBB2B	22	1
TUBB2A	22	1
CHD4	37	37
NASP	24	24
MAP4	32	32
PDIA3	36	36
SF3B2	33	33
GTF2I	40	40
PGK1	30	30
PLS3	29	26
USP15	39	38
HSPH1	28	25
DDX17	32	27
PSMD1	27	27
KPNB1	21	21
UPF1	32	32
CKAP5	40	40
MATR3	35	35
LMNA	28	2
MSN	33	21
FUBP1	27	20
CSE1L	36	36
P4HB	31	31
EFTUD2	40	40
TKT	25	25
SMC2	39	39

CKB	21	19
MCM6	32	32
PDCD6IP	35	35
PABPC1	35	27
TCP1	28	28
TUBB4A	18	2
MYO6	39	39
PSMD2	26	26
SSB	27	27
HNRNPH1	18	9
DIS3	27	27
LMNA	27	1
MYH2	24	1
ANXA6	37	37
CANX	28	27
RPSA	15	15
ACTA1	22	3
GARS1	31	31
CALU	16	4
ASNS	25	25
GCN1	38	38
ILF3	27	22
HNRNPL	21	20
GART	35	35
TUFM	26	26
EEF1A1	22	11
SYNCRIP	25	21
HNRNPUL1	25	25
MCM5	30	30
EIF3D	22	22
HDLBP	35	35
CKAP4	27	27
DHX15	33	32
RRM1	26	26
TUBA8	15	1
RPA1	24	24
TFG	12	12
PTBP1	24	21
YLPM1	29	29
GMPS	26	26
DDB1	29	29
COPB1	29	29
MCM4	25	25
MCM3	31	31
ATIC	24	24
MYH11	30	1
NUP205	42	42

SERPINH1	20	20
GDI2	25	16
HNRNPA2B1	21	20
RPL4	28	28
BCLAF1	32	3
NPEPPS	33	33
MCM7	29	29
HNRNPK	24	24
DDX21	30	28
EIF3E	24	24
WARS1	24	24
CCT6A	26	26
RPN1	27	27
CALR	21	21
RARS1	31	31
DDX1	31	31
IMPDH2	19	17
SEC23A	22	21
TARS1	31	31
PPP2R1A	22	16
ACTA2	20	1
HYOU1	30	30
EIF3G	22	22
ACO2	28	28
DDX5	34	29
EEF1G	30	30
CCT4	25	25
UGGT1	32	32
RBM6	36	34
WDR1	19	19
EZR	34	20
HADHA	24	24
DCTN1	27	27
MCM2	27	27
U2AF2	19	19
EIF4G1	32	29
BCLAF1	29	1
LTA4H	24	24
HNRNPA1	16	13
PYGB	25	16
MYBBP1A	31	31
EIF3H	23	23
ATP2A2	26	26
IARS2	28	28
SHMT2	25	24
PSMD11	28	28
PGM1	26	26

NUP155	28	28
VARS1	26	26
CORO1C	19	18
COPB2	27	27
TMPO	22	13
EIF4A3	24	21
SLC3A2	23	23
ALDOA	21	19
NUP93	32	32
ALDH1L2	29	29
FUS	12	10
TUBB6	17	7
CSNK2A1	18	18
RPN2	18	18
DBN1	18	18
DARS1	25	25
LRRFIP2	17	16
IPO7	23	23
TJP2	32	31
DDX42	24	24
SMARCA5	28	18
PSMC5	19	19
LMNB2	28	26
UBAP2L	19	19
TMPO	18	9
TUBB3	17	3
PABPC4	23	14
KIF5B	25	18
G3BP1	16	15
ACTR1A	16	16
EPB41L2	31	27
SSRP1	24	24
ELP1	22	22
PPM1B	20	17
USO1	24	24
EEF1A2	18	7
SERBP1	23	0
SF3A1	26	26
CALU	14	2
AP2B1	24	13
MYH8	20	2
LDHB	19	17
EIF3I	16	16
RTCB	20	20
AIFM1	20	20
SERBP1	23	1
RPL3	26	26

NAA15	29	25
RCN1	15	15
SERBP1	23	1
ALDH7A1	18	18
SUPT16H	24	24
DDX39B	21	9
FARSA	20	20
IGF2BP1	24	22
PFKM	22	19
NAP1L1	12	10
COPG1	22	20
OLA1	18	18
TCOF1	24	24
NSUN2	26	26
HNRNPF	14	11
UMPS	16	16
HNRNPR	22	18
AHCY	19	19
PFKL	15	11
DHX30	27	27
CUL1	23	23
PFAS	22	22
QARS1	26	26
CS	16	16
HNRNPD	15	2
PSMC3	20	20
ALB	27	27
PSMD6	19	19
AP1B1	26	15
LONP1	23	23
EIF5B	20	20
NAMPT	19	19
HSD17B4	20	20
THRAP3	25	23
GDI1	19	10
VPS35	19	18
GLUD1	20	20
ECPAS	29	28
PHGDH	18	18
EIF4G2	27	27
DNM2	22	17
HNRNPA3	16	14
YBX1	14	8
SUPT5H	21	21
TFRC	21	21
PRMT5	23	23
IRS4	23	23

PSMD3	27	27
FUBP3	20	17
RUVBL1	19	19
PA2G4	22	22
ABCE1	18	18
DNM1L	18	18
FLNB	23	18
EIF3M	11	11
IDE	22	22
EDC4	21	21
ILF2	20	20
PSMC2	21	21
HSPA4L	22	19
XRN2	20	20
SMARCA4	21	21
ATP6V1A	22	22
LDHA	18	16
EIF3F	15	15
RCN2	13	13
NDUFS1	21	21
ATAD3A	27	8
PAICS	20	20
PRMT1	16	16
YBX3	13	2
MSH6	24	24
GSPT1	22	10
OAT	18	18
NCAPD2	22	22
YBX3	12	1
PUF60	18	18
NPM1	15	15
PSMD12	21	21
KRT14	18	7
RANGAP1	17	17
PFKP	16	13
NAT10	22	22
HSPA1L	16	1
DDOST	15	15
TTLL12	18	18
GIGYF2	18	18
MED23	21	21
AP2A2	21	16
PCK2	19	19
CTTN	19	19
RDX	25	10
HK1	26	22
SLC25A13	20	17

ADAR	20	20
XPO5	22	22
RBBP7	14	9
NUDC	21	21
MTREX	21	21
KRT6B	24	6
FH	20	20
PRPF6	20	20
EIF4A2	17	4
PRPF19	15	15
KHDRBS1	16	14
EIF2S2	17	17
LARP1	20	17
SCFD1	16	16
SEC31A	22	22
RBBP4	14	9
CSDE1	27	27
HNRNPD	14	1
KARS1	19	19
SMU1	13	13
KPNA2	16	16
USP5	17	17
SRP68	20	20
ATAD3B	24	5
TXNRD1	13	13
RAD50	23	23
RPS3	15	15
SEC24C	21	21
ATXN2L	20	19
NUMA1	19	19
MAT2A	18	18
RBM5	20	15
CTPS1	15	14
PRKCSH	19	19
AGO1	19	11
STOML2	15	15
KTN1	18	18
NELFE	12	12
ETF1	17	17
IPO4	20	20
PSMC1	19	19
ZC3HAV1	21	21
CCAR1	21	21
ATP1A3	14	1
RNPEP	15	15
ERLIN2	15	13
SF1	14	2

COPS4	14	14
CDC5L	17	17
NAP1L4	13	11
PSMC4	17	17
USP9X	23	23
MOV10	22	22
KRT8	21	12
NOP58	15	15
EPB41L3	18	14
PPAT	15	15
ESYT1	14	14
DCTN2	15	15
PSMC6	15	15
AGO2	20	14
GFPT1	19	19
DDX19A	16	16
YWHAE	17	14
TPM3	22	14
CYFIP1	23	10
G6PD	19	19
NSF	24	24
FLII	19	2
NUP133	20	20
SEPTIN2	12	12
BAZ1B	18	18
RPS6KA3	19	13
ZNF326	17	17
TPR	20	20
RECQL	16	16
RPS27A	13	13
UQCRC2	14	14
CLINT1	16	16
SNRNP70	22	22
LAP3	17	17
TNPO1	18	12
CBS	16	16
AMOT	15	14
HCFC1	19	19
MDH2	13	13
MSH2	20	20
SMC1A	22	22
ABCF1	16	16
CTNND1	16	16
SNX2	17	15
USP7	17	17
ARCN1	19	19
DSG2	12	12

TWF2	10	9
SF3A3	15	15
SAFB	20	14
PSMD4	12	12
HACD3	11	11
RRP12	23	23
CNOT1	20	20
UBA2	15	15
DPYSL2	13	13
CAP1	20	18
PCBP2	11	1
SF1	13	1
POLR2B	17	17
RBM14	19	19
STRAP	12	12
ABCD3	22	22
TUBG1	13	13
RSL1D1	19	19
FLII	18	1
SDHA	16	16
AP3D1	16	16
NCAPH	15	15
DDX46	20	20
HNRNPH2	16	8
EXOSC10	19	19
SARS1	15	15
NOC3L	14	14
CTNNA1	20	20
ALDH2	17	16
POLD1	21	21
TOP2A	21	13
PLBD2	16	16
CUL4B	23	15
SMARCA1	21	11
CTNNB1	14	12
NOP56	18	18
TPP2	21	21
API5	17	17
TMOD3	15	13
CLASP2	18	16
FANCI	21	21
AP3B1	22	18
EIF2S3	16	16
GTPBP1	13	13
RPL6	12	12
DYNC1LI1	16	16
TOP1	18	18

LBR	15	15
UBQLN2	9	6
SET	11	11
SART1	14	14
PUS7	16	16
RO60	15	15
RCC2	16	16
GOT1	12	12
CACYBP	10	10
XPO7	21	21
RAB3GAP2	19	19
GSS	14	14
STAT3	11	11
HADHB	19	19
KRT5	21	11
FXR1	11	10
NCKAP1	15	15
DHX38	18	17
SMARCC1	17	11
NDUFS2	12	12
AHSA1	16	16
ABCF2-H2BK1	18	18
LETM1	15	15
NARS1	16	16
NCBP1	15	15
AGO3	18	10
CARS1	18	18
PCBP1	12	8
NSFL1C	12	12
CCDC47	13	13
GLS	12	1
KLC1	16	12
IDH2	18	16
CNDP2	13	13
VAT1	12	12
ACBD3	11	11
ATXN10	16	16
UQCRC1	14	13
NUP153	16	16
HDGF	13	11
TCERG1	19	19
IPO9	14	14
GEMIN5	21	21
XPOT	17	17
HNRNPC	15	15
DDX6	12	12
LMAN1	13	13

KIFBP	13	13
SIN3A	16	16
EIF2A	16	16
TXLNA	11	11
EWSR1	8	8
RBM12	16	16
ALDH1B1	13	11
TOMM70	11	11
FARSB	17	17
NUP98	19	19
NUP107	19	19
STK38	13	11
FAM98B	9	6
PCBP2	11	1
HDAC1	10	4
PTPN11	15	15
GOT2	15	15
ACO1	18	18
SYAP1	12	12
HDAC2	12	6
PSAT1	17	17
PLAA	12	12
NXF1	15	15
HNRNPAB	14	14
ADSL	15	15
PGAM1	12	12
FAM98A	9	6
MTHFD1L	20	18
BAG6	14	14
NAE1	11	11
MYH3	14	1
STAT1	15	15
HARS1	14	14
GNL3	16	16
RPS3A	17	17
ENAH	15	15
PCNA	13	13
NUP210	17	17
IGF2BP3	15	12
ROCK1	17	15
MYO1C	16	16
ACADM	14	14
PRDX6	13	13
EPB41	17	16
TMEM43	11	11
AFG3L2	16	16
NCLN	9	9

TAF15	14	12
IDH1	15	13
SRRT	19	19
PPP6R3	16	16
CYFIP2	16	3
IGF2BP2	13	11
MYH7	12	2
USP14	13	13
UGDH	11	11
TEX10	17	17
PLOD1	13	13
NCAPG	16	16
DHX36	11	11
FAM120A	10	10
DLD	11	11
PRKAR2A	13	10
NOC2L	15	15
FLOT1	11	11
ACAT1	13	13
C1QBP	7	7
TPM1	16	7
CCAR2	15	15
CLPB	12	12
MARCKS	8	8
FAF2	10	10
YWHAZ	13	8
COPS2	17	17
DYNC1H1	20	20
GATAD2B	9	8
VRK1	12	12
FLOT2	12	12
MTA2	19	15
PHB2	13	13
RPL5	12	12
BSG	7	7
PRPSAP2	10	9
TPI1	10	10
GRWD1	9	9
COPG2	13	11
ACSL3	12	10
ANXA2	15	15
PKN2	15	15
CTH	11	11
DIAPH1	18	18
GBE1	14	14
CAPRIN1	10	10
STAG2	14	11

USP10	11	11
PGD	16	16
PITRM1	16	16
DNAJA1	13	13
AP2A1	15	10
ELAC2	11	11
U2SURP	15	15
CAPZA1	11	7
SRPK1	12	11
TIAL1	14	7
CKMT1A	14	12
MDH1	11	11
TSG101	12	12
RPS6KA1	13	7
PPP2R1B	11	5
SRP72	13	13
SUCLA2	11	11
UBQLN1	8	4
HBS1L	11	11
FEN1	14	14
FSCN1	13	13
ACTR2	12	12
ANXA5	14	14
PLIN3	11	11
DNAJC7	13	13
PMPCA	9	9
GTPBP4	16	16
KPNA6	9	2
SART3	13	13
PAFAH1B1	10	10
NNT	16	16
CPSF7	10	10
DEK	9	9
RBM39	14	14
CEBPZ	10	10
UBA6	16	16
DDX18	15	15
H1-2	13	5
DDX54	14	14
DLST	12	12
GLS	12	1
PSPC1	16	13
SPAG9	12	12
TBCE	10	10
WDHD1	14	14
CBR1	12	12
RPS6	10	10

KIF11	16	16
MMS19	14	14
ACTN2	11	1
CARM1	11	11
SEPTIN9	13	13
CPSF1	12	12
CAPN1	16	16
DARS2	14	14
DDX50	14	12
SNX1	12	10
MOGS	14	14
EIF5	13	13
AP1G1	16	16
AP1M1	11	11
HNRNPDL	11	9
UNC45A	12	12
PSMD13	12	12
SPTBN2	11	4
NUP54	10	10
OSBP	11	11
KDM1A	10	10
FKBP5	12	11
CPSF6	13	13
CNP	14	14
ANKFY1	16	16
DDX39A	14	2
PGM3	13	13
SEPTIN11	13	8
ACAT2	11	11
HNRNPH3	10	10
DPYSL5	13	13
TMOD1	11	11
RAN	10	10
TRMT10C	12	12
UBTF	14	14
MYO1B	16	16
SAE1	9	9
MAP2K1	11	6
FBL	9	9
TOR1AIP1	7	7
TRIP13	13	13
SEPTIN7	10	10
DHX29	12	12
HEATR1	18	18
VDAC1	10	10
KIF2A	15	14
PREP	12	12

RAD23B	10	8
PPM1G	12	12
AGL	14	14
GLA	8	8
PALS2	12	10
NT5DC1	10	10
HP1BP3	11	11
PPP2R2A	9	5
AARS2	12	12
PRPF40A	15	15
GSR	9	9
STK38L	12	10
CUL3	14	14
ERO1A	11	9
MMUT	11	11
PDS5B	16	14
RPLP0	13	13
CAPN2	15	15
GSPT2	13	1
RBM26	16	15
EHD1	13	12
GAPVD1	12	12
PRPSAP1	6	5
FAF1	12	12
RCC1	8	8
TOMM40	10	10
MYO18A	15	15
ME2	11	11
BMS1	14	14
COG4	11	11
PDCD4	10	10
SRP54	12	12
CPNE3	11	11
NOMO2	11	11
DDX27	15	15
NUP160	11	11
MRPS27	9	9
OXCT1	9	9
EEF1D	11	11
ACTBL2	11	3
CDC37	11	11
EIF3J	8	8
VDAC3	8	8
SAFB2	9	3
LRRC40	15	15
SEC24B	12	11
PC	10	10

MAP2K2	13	8
IDH3B	11	11
DDX56	12	12
KRT16	13	2
BUB3	7	7
STAU1	12	11
STK26	10	10
UBE3C	14	14
HNRNPUL2	14	14
UFL1	15	15
H1-4	11	3
YTHDF2	9	7
DPP3	9	9
NPLOC4	10	10
LARS2	13	13
TNPO3	9	9
ANXA11	13	13
NOC4L	8	8
ATP6V1B2	13	13
ADD1	12	10
ADSS2	11	11
ZC3H15	9	9
IPO8	14	14
OGA	14	13
PRPF3	11	11
SLK	9	8
TARS2	10	10
CDC73	14	14
AGPS	9	9
MYL6	9	7
CPSF3	8	8
TBCD	13	13
ATP2B1	12	6
HNRNPA0	4	4
MTDH	8	8
PNPT1	13	13
BZW1	16	14
ALDH9A1	13	13
NACA	5	5
KRT3	12	1
GAA	10	9
TARDBP	9	9
PAK2	11	8
TIMM44	9	9
PDS5A	11	9
NEFM	8	7
EMD	7	7

PYGM	10	1
FERMT2	10	10
CHERP	8	8
TOP2B	13	5
NEK9	13	13
EIF2AK2	14	14
DFFA	8	8
NMT1	8	7
PRMT3	8	8
VDAC2	9	9
SEC23IP	9	9
SUCLG2	12	12
ADH5	9	9
HDHD5	8	8
CSTF1	8	8
PTCD3	11	11
CAPZA2	10	6
PYCR2	6	6
RBM15	13	12
CTBP2	9	6
SNX9	10	10
ACTR3	8	8
SLC25A5	15	3
ACTL6A	6	6
USP39	11	11
MPI	7	7
TALDO1	13	13
CAT	9	9
RPS4X	17	17
GSN	10	10
DRG1	10	9
CSNK2A2	10	10
DAP3	10	10
ISYNA1	9	9
AGK	9	9
RPAP3	10	10
ZW10	11	11
THOP1	12	12
PTPN1	8	8
PAPSS1	10	10
NUP50	5	5
DDX23	14	14
PDE12	7	7
SACM1L	12	12
DNAJA2	11	11
P4HA1	12	12
FIP1L1	9	9

RTN4	7	7
OPA1	12	12
NUP85	11	11
KRT75	11	1
HAT1	9	9
SLC25A4	13	2
TPM2	12	2
RBM12B	15	15
GRSF1	8	8
GFM1	13	13
COPS3	10	10
YARS2	10	10
ELAVL1	9	8
NOP14	11	11
AP3M1	11	8
DCP1A	7	7
TXLNG	10	10
EIF2D	8	8
TKFC	9	9
KPNA5	6	1
SNW1	10	10
SLC25A24	14	14
CMTR1	13	13
CYB5R3	6	6
UBE2O	10	10
OGT	12	2
KRT18	12	10
PDHA1	14	14
DDX41	10	10
DDX47	12	12
DBNL	7	7
MCMBP	12	12
YTHDF3	9	5
TPM4	13	4
ANXA7	9	9
PPP1CA	8	4
PPP1R8	9	9
WDR77	6	6
RBMX	17	4
UBLCP1	9	9
YWHAQ	11	5
ST13	10	10
EMC1	13	13
DOCK7	13	13
PRPS2	8	4
PELP1	9	9
EEFSEC	10	10

ACADVL	11	11
PRPF4	10	10
APMAP	10	10
APPL1	7	7
PDHB	9	9
AP2M1	13	13
SEC63	10	10
TPP1	4	4
DKC1	13	13
PPIL4	8	8
WRNIP1	12	12
EIF2S1	9	9
POLDIP3	8	8
MLEC	8	8
POTEE	7	1
NANS	7	7
G3BP2	9	8
PSMA3	8	8
FAM50A	8	8
RBM4	9	9
UBA5	5	5
TUBGCP2	10	10
CTPS2	8	7
APEH	10	10
TSR1	13	13
ARMT1	9	9
EEF1B2	5	5
RRM2	7	7
DLAT	11	11
VANGL1	7	7
SMARCC2	10	4
SNRPA	7	7
PEPD	9	9
GALNT2	7	7
STRBP	11	6
ANAPC7	9	9
ACOT7	6	6
PANK4	10	10
GBF1	10	10
PLRG1	9	9
MRPS22	12	12
PPA1	7	7
SAMM50	11	11
BCCIP	7	2
NOP2	12	12
RAB3GAP1	8	8
CDK1	10	10

DNAAF5	11	11
PAPOLA	12	11
RACK1	9	9
HNRNPLL	7	7
PES1	17	17
NDRG1	5	5
SKIC3	8	8
SMCHD1	10	10
CSTF3	10	10
BPNT1	9	9
SHMT1	7	6
PACSIN2	9	9
RPL7A	10	10
EXOC4	9	9
KLC2	11	7
LRRC47	10	10
SUPT6H	11	11
DAZAP1	5	5
MTA1	10	5
HK2	10	6
CIP2A	9	9
AAAS	11	11
TIA1	13	6
PIGS	7	7
RNF40	9	7
KYAT3	9	9
SYMPK	13	13
BRIX1	6	6
EHD4	10	9
TBRG4	9	9
KCTD12	9	9
SLTM	6	6
PSMD7	8	8
CNN3	6	6
AFDN	9	9
ARHGEF2	11	11
ARIH2	6	6
MYH13	11	2
NT5DC2	8	8
SF3A2	7	7
LUC7L3	9	9
DPY19L1	8	8
MRPL37	8	8
SLC25A6	14	3
MAGED2	9	9
UBQLN4	7	5
NCOA5	7	7

ITGB1	9	9
SAMHD1	11	11
CORO7-PAM16	7	7
DNAJC11	10	10
RPL10	9	9
SEC24A	8	7
GNE	7	7
LYAR	9	9
ALYREF	5	5
PGM2	10	9
FKBP10	10	10
PSIP1	5	3
SRSF4	8	5
INA	8	7
TXNDC5	11	11
MRE11	11	11
TWF1	7	6
PIP5K1C	7	6
PRDX1	8	6
CAPZB	8	8
PPWD1	9	9
SARS2	9	9
DHX16	10	9
LARP7	11	11
BCCIP	6	1
THBS1	11	10
VPS4A	8	5
ZYX	7	7
NKRF	12	12
OGT	11	1
GTF2F1	8	8
PSMB4	6	6
DNAJA3	8	8
MCCC2	12	12
CWF19L1	7	7
WDR33	9	9
RBMXL1	14	1
ERP44	9	9
ILVBL	6	6
ESYT2	10	10
CYP51A1	11	11
PPP5C	9	9
SERPINB6	8	8
SH3GL1	7	7
EPHX1	11	11
HDGFL2	10	8
TNPO2	9	3

NUDCD1	8	8
PLOD3	9	9
VPS4B	7	4
FTSJ3	10	10
RBM34	8	8
PMPCB	8	7
ZFR	6	6
HELLS	11	11
TES	10	10
WDR26	8	8
NELFB	10	10
CFL1	7	7
CRKL	9	9
AIMP1	5	5
WBP11	7	7
RRP1B	7	7
SEPTIN6	8	3
SCAF4	8	5
NRDC	11	11
EPS15L1	9	9
RBM28	8	8
ATL3	8	8
GYS1	10	10
DNAJC10	9	9
SNX5	8	8
RPRD1A	5	4
CMAS	7	7
SLC1A5	9	9
SFXN1	8	8
GPHN	8	8
CTR9	9	9
NOL9	8	8
PSMA5	6	6
CSK	9	9
PRKAA2	11	8
PPP1R7	9	9
H1-3	9	2
SPART	7	7
MYO5A	10	8
GEMIN4	9	9
EML4	10	10
SEC23B	9	8
DNMT1	12	12
ARFIP1	4	4
FMR1	9	1
PDLIM1	5	5
CDH2	6	6

DNAJB11	5	5
SRPRA	9	9
BYSL	10	10
WASHC5	8	8
INTS7	9	9
HTRA1	7	7
RNF20	7	5
NUCB2	5	5
HEATR3	13	13
CPSF2	9	9
GTF3C5	10	10
RFC2	8	8
GPD2	8	8
PPIA	9	8
PDCD11	13	13
CTNNBL1	10	10
CSTF2	8	8
NAA25	8	8
PUM3	10	10
PUM1	9	9
PHB1	7	7
CORO1B	9	9
NUP214	7	7
RAE1	9	9
UAP1	9	9
POR	9	9
ACSL4	8	6
ALDOC	4	2
SLC25A3	9	9
CUL2	10	10
TBC1D15	9	9
NOLC1	11	11
FMR1	9	1
NHLRC2	12	12
TP53	9	9
XIAP	9	9
PEBP1	4	4
RPS2	10	10
CDC16	6	6
CTBP1	10	7
DPYSL4	7	7
PABPN1	6	6
POFUT1	5	5
PTBP2	9	8
RPL9	6	6
AKAP8L	5	5
SMC6	8	8

ACOT2	6	6
NDUFA10	7	7
LUC7L2	9	6
RAD21	7	7
THUMPD1	9	9
COQ8A	7	7
CPNE1	9	9
CAMK2G	9	5
CUL4A	11	3
GLRX3	9	9
PCMT1	7	7
GLYR1	6	6
PRDX4	7	6
BLMH	9	9
PPM1A	4	1
NME1	6	6
SEPHS1	8	6
POLA1	6	6
TRIM33	5	5
PCYOX1	8	8
NDUFV1	8	8
SMARCE1	7	7
DSP	11	11
PPP1CB	5	1
SLAIN2	7	7
OTUB1	6	6
SETD3	9	8
MAPK3	8	7
AHCYL1	10	10
RANBP3	9	9
STT3B	9	7
CSNK1D	7	2
KRT13	8	2
BROX	9	9
IPO11	10	10
PRKAR1A	9	9
RPS8	6	6
CLNS1A	4	4
CDK5RAP3	9	9
HEXA	10	10
AMPD2	10	10
DDI2	4	4
BRD2	8	7
DNPEP	7	7
ALCAM	5	5
QTRT2	6	6
PPP1CC	5	1

TUBAL3	4	1
UGP2	10	10
STXBP2	6	6
SCYL1	9	9
RANBP2	9	9
GNAI3	5	3
JUP	10	8
GET3	6	6
POGLUT3	7	7
XPO4	7	7
WDR48	5	5
ATXN2	5	4
TOM1	6	6
AKR1B1	7	6
GATAD2A	8	7
PSMA7	7	7
AAMP	6	6
STXBP3	9	9
ACAD9	8	8
PFKFB3	6	3
FBXO22	6	6
PSMA1	6	6
PDLIM5	7	7
SARNP	7	7
ALDH6A1	8	8
UTP25	8	8
HMBS	7	7
ALDH3A2	7	7
SDF4	7	7
ARHGEF1	4	4
TRMT1	6	6
LAS1L	5	5
ZPR1	6	6
PSMD14	6	6
PTBP3	7	4
FXR2	6	4
KPNA1	6	2
TBC1D4	9	9
PLCG1	10	10
POLD2	4	4
GALK1	6	6
SNX6	9	8
KIF5C	8	1
AAR2	5	5
RPTOR	5	5
TERF2IP	5	5
MAPRE1	5	5

TFCP2	3	2
CLUH	12	12
CPVL	7	7
SLC25A12	8	5
SWAP70	6	6
GPS1	9	9
METAP2	6	6
STT3A	11	9
EEA1	6	6
YWHAB	10	2
EIF2B4	6	6
GCDH	5	4
NCAPG2	7	7
STK39	5	3
DYNC1I2	5	5
SRSF11	4	4
OSBPL9	8	8
DNAJC2	8	8
TECR	8	8
PRPS1	6	2
PFN1	7	7
PLOD2	7	7
SPTLC1	4	4
WASHC2A	5	5
PAK1	7	4
PRPF31	8	8
RAVER1	7	7
GLMN	7	7
TBL1XR1	6	3
RTCA	6	6
UBA3	10	10
SENP3	7	7
SCAMP3	4	4
PPP1R12A	7	6
ZC3H14	6	6
XPNPEP1	9	9
OXSR1	7	5
NUDT5	5	5
YTHDF1	5	2
POP1	7	7
KRI1	5	5
UBP1	4	3
NUP62	4	4
EBNA1BP2	7	7
DCTN4	6	6
STRN3	8	7
ZMPSTE24	6	6

PRRC1	5	5
COASY	6	6
FOXK1	7	6
CDC27	7	7
PI4K2A	5	5
NVL	7	7
RBM17	7	7
CSNK1E	6	1
POGZ	7	7
POLR1B	6	6
TAGLN2	6	6
SRSF5	8	7
AGFG1	6	6
CHORDC1	7	7
SCRIB	9	7
IDH3A	7	7
OSBPL11	5	5
TRIM25	6	6
OGDH	7	7
ORC4	6	6
CPD	9	9
CKAP2	5	5
SEL1L	5	5
NLN	10	10
UBFD1	5	5
FLNC	8	1
YWHAG	8	1
WDR18	7	7
VPS36	5	5
RPS9	10	10
ERLIN1	7	5
GNL1	7	7
NADK2	7	7
VPS45	8	8
RPL23	5	5
BOP1	7	7
PSMA4	7	7
ACOT9	8	8
PPM1F	5	5
KPNA3	5	2
DHTKD1	5	5
BTAF1	7	7
NMD3	6	6
GNL2	4	4
JUN	4	4
TRIP12	6	6
STRN	5	4

APEX1	8	8
CALD1	7	6
BCKDHA	4	4
TBL2	4	4
SBF1	11	11
SRM	7	7
PBRM1	6	6
HPRT1	6	6
RPRD1B	5	4
AK2	5	5
CUL5	10	10
FDXR	8	8
ASPH	8	8
HLTF	4	4
GSTP1	6	6
NUFIP2	7	7
PPP2R5D	6	3
EIF2B3	6	6
SEC24D	9	9
DDX20	5	5
MAGED1	5	4
RBM27	6	5
ATP5F1C	6	6
KIF4A	8	8
POLD3	6	6
AKR7A2	4	4
RPL10A	5	5
PSME3	3	3
ASS1	9	9
DPF2	4	4
SLC4A1AP	7	7
INTS4	7	7
SDAD1	8	8
KDM3B	5	5
HSPB1	4	4
ALDH16A1	4	4
NOB1	7	7
ZRANB2	5	5
PRCC	4	4
SLC12A2	4	4
PDXDC1	8	8
GRHPR	6	6
PNN	7	7
STX12	3	3
HSPA14	7	7
ABCB10	5	5
RBFOX2	6	6

SUPV3L1	5	5
PRRC2A	6	5
ADNP	6	6
QKI	4	4
VPS26A	6	5
KIF22	4	4
HSDL2	6	6
PRDX2	7	6
PELO	6	6
WDR44	7	7
DRG2	7	6
CLASP1	7	5
COMP	5	5
MTA3	7	1
IRAK1	4	4
CAP2	6	4
HMGN5	3	3
UCHL5	6	6
ASH2L	5	5
SLC27A4	5	5
WDR12	4	4
LPCAT1	6	6
SMS	6	6
MTR	6	6
SHPK	5	5
RPL13	9	9
PSMA2	4	4
GSK3A	5	3
ATP6V1H	6	6
CDK11B	6	6
CEP170	5	5
WTAP	4	4
PM20D2	4	4
PRCP	5	5
ATP6V0A1	5	5
RIC8A	10	10
FANCD2	8	8
SURF4	3	3
CDV3	4	4
PLS1	5	2
SCARB2	7	7
ZNF512	7	7
PRKAA1	8	5
CELF1	6	6
RPL8	10	10
PUS1	5	5
KPNA4	4	1

GTPBP10	6	6
RBM25	9	9
MEPCE	6	6
CAMK2D	7	3
OCRL	7	7
MTOR	6	6
HMGB1	4	4
SUN2	4	4
DNM1	6	1
DHCR7	4	4
C3	6	6
MSH3	7	7
U2AF1	2	2
USP11	7	6
RPUSD2	3	3
HGS	6	6
RPL18A	8	8
IKBIP	5	3
ARHGAP1	7	7
RELA	5	5
TCEA1	7	7
STK3	5	1
RFC3	6	6
TRMT1L	8	8
DDX52	5	5
ENO2	4	2
RANBP1	4	4
PSMA6	5	5
MRPS31	5	5
ELMO2	5	5
BZW2	10	8
CNOT3	7	7
SNX27	10	10
EXOC7	4	4
YWHAH	7	2
MON2	8	8
IDH3G	5	5
RBM22	5	5
EARS2	4	4
NOL6	7	7
SP1	3	3
CFDP1	5	5
PRKAG1	4	4
TRIP6	3	3
MRPS35	5	5
RNF2	5	4
ECHS1	4	4

SRRM2	7	6
SRBD1	4	4
PARP2	5	5
PAIP1	4	1
RNGTT	6	6
NFS1	6	6
SARM1	4	4
GNAS	6	6
SMARCB1	4	4
EPS15	5	5
ATP2B4	7	1
POLDIP2	5	5
LUC7L	7	4
PIGT	9	9
C11orf58	2	2
EIF4G3	7	4
XRN1	4	4
GNB1	4	4
UROD	5	5
THOC2	8	8
TFIP11	6	6
VANGL2	6	6
IST1	5	5
FKBP3	5	5
GLB1	5	5
SCCPDH	6	6
RFC1	7	7
MYL12B	6	6
ZC3H11A	5	5
RPL15	4	4
ESD	4	4
SMARCD2	4	3
PPP2R2D	5	1
LSM14B	3	3
GRIPAP1	6	6
B3GLCT	4	4
CBL	5	5
ORC5	6	6
TAB1	5	5
PNP	6	6
USP48	4	4
STK4	6	2
NUP43	4	4
VPS33B	5	5
SGPL1	7	7
HSD17B10	4	4
GTF3C2	4	4

KRT74	4	2
LRRFIP1	4	3
RCOR1	4	4
PFKFB2	5	2
PXDN	6	6
TERF2	3	3
EYA3	6	6
CPT2	8	8
PPME1	7	7
CLPX	4	4
STXBP1	5	5
CCDC6	5	5
IQGAP2	7	6
GMDS	6	6
ENPP1	4	4
CA2	6	6
POU2F1	3	3
ATP1B3	4	4
DBT	4	4
STAU2	6	5
FAR1	5	5
ACAA2	5	5
PPP4R3A	7	7
RNH1	3	3
ADRM1	5	5
HM13	4	4
NCSTN	6	6
LIMA1	4	4
VTA1	4	4
PRKAR2B	4	1
GLG1	6	6
ITIH2	4	4
PICALM	7	7
PPP2CA	5	5
AGAP3	4	4
ZCCHC8	4	4
ERCC6L	6	6
WAPL	4	4
GNAI1	3	1
STAM	4	4
GIPC1	7	7
SEPTIN8	6	3
CLK3	3	3
UBXN1	4	4
PRKACA	5	5
PIP4K2C	5	5
ILKAP	5	5

CDC23	6	6
MAD1L1	4	4
ALDH5A1	4	4
GNAI2	3	1
KRR1	6	6
IWS1	5	5
SERPINB1	5	5
COPS5	4	4
THOC1	6	6
UHRF1	5	5
PTK7	5	5
TBL3	5	5
PSMB3	4	4
MYL11	4	4
ORC2	4	4
GPKOW	5	5
ILK	7	7
KRT19	7	1
TTI1	3	3
PTGES3L-AARSD1	5	5
NUP58	2	2
OSBPL8	4	4
TLE3	4	2
CD2AP	6	6
SMPD4	7	7
PURA	3	3
CLIC1	5	4
DNAJB1	8	8
SEMA3C	7	6
LSM14A	5	5
SMC5	5	5
FECH	6	6
SRPRB	3	3
RPS16	6	6
TELO2	7	7
ABCF3	4	4
MTMR1	6	6
AASS	4	4
RRP1	6	6
ADK	6	6
PLK1	4	4
NUCKS1	2	2
CRYZL1	3	3
TXNRD2	3	3
STAMBP	5	5
RNPS1	4	4
GSTO1	7	7

MAPK1	3	2
A2M	7	7
SUGT1	3	3
GLUL	4	4
LSS	5	5
ANP32E	3	3
CAND2	4	1
LMAN2	5	5
MYO5B	5	3
LIG1	5	5
ACIN1	6	6
BCS1L	6	6
NSMCE4A	5	5
ANP32B	6	4
PTK2	7	7
LARP4B	4	4
PDIA5	5	5
CDC45	6	6
PGP	3	3
RIOX2	7	7
RFC5	5	5
PRDX3	3	3
RPL18	4	4
CHUK	5	5
ARFGAP3	4	4
INTS5	4	4
RPL7	9	9
NELFA	4	4
RPS15A	6	6
GMPPA	3	3
MRI1	4	4
SLC7A5	3	3
NUDT19	4	4
ZC3H18	5	5
ARAF	5	3
MYEF2	5	5
SF3B4	4	4
UFD1	3	3
SH3GLB1	3	3
LARP1B	4	1
CAST	4	4
SNTB2	5	5
SLC25A11	6	6
COLGALT1	9	9
GSK3B	4	2
CDC123	7	7
CRNKL1	5	5

ITCH	5	5
PPP2R5C	4	1
STAG1	4	1
XRCC1	5	5
IK	6	6
LEO1	3	3
PARN	4	4
SORD	3	3
LTV1	6	6
CIAPIN1	4	4
CYC1	2	2
FNTA	5	5
AACS	5	5
POLR3A	5	5
GORASP2	5	5
PIP4K2A	3	3
MAP4K4	6	2
POLRMT	4	4
PPID	5	5
DHX57	7	7
ABI1	3	3
TPT1	3	3
MAPK9	5	5
AQR	7	7
CNIH4	1	1
IMPDH1	6	4
FAM91A1	5	5
ARIH1	3	3
CASK	3	3
NCDN	4	4
MYG1	5	5
RPL21	6	6
LRRC59	4	4
CAMSAP3	3	3
SRSF2	4	4
NXN	5	5
SMARCAD1	4	4
RPL12	3	3
DIAPH3	4	4
RINT1	4	4
NT5C2	4	4
AIMP2	2	2
RRP8	2	2
METTL16	4	4
HEXIM1	2	2
MAVS	2	2
FTO	6	6

LIG3	8	8
USP47	4	4
DYRK1A	4	4
PAIP1	4	1
CHID1	3	3
ATP2B3	6	1
NAA16	5	1
ZNF703	3	3
BCKDK	4	4
TNIK	5	1
VAC14	4	4
NUP88	4	4
SCML2	5	5
SETD7	3	3
MAPRE2	2	2
STAM2	3	3
ZNF629	6	5
PAF1	4	4
MARCKSL1	3	3
SOAT1	3	3
PHACTR4	4	4
METTL3	4	4
HADH	4	4
TRMT5	6	6
STRN4	4	3
NOM1	3	3
GTF3C4	5	5
ACP2	6	6
DBR1	4	4
SPOP	3	3
TIMM50	2	2
ATG3	3	3
BRD4	6	5
PTPN23	4	4
PPHLN1	8	8
QRICH1	3	3
ANGEL2	2	2
IKBKG	2	2
SRSF6	6	3
ABCB7	5	5
ATG7	6	6
EDC3	6	6
ADD3	6	5
BLVRA	4	4
CHCHD2	1	1
TIPRL	3	3
ERCC2	5	5

SMAD5	3	3
TMOD2	6	4
WDR11	4	4
RPS21	2	2
ZNF768	6	5
CADM1	6	6
GGH	4	4
SUZ12	5	5
SEH1L	2	2
MEAK7	2	2
SEPHS2	7	5
ARFGAP2	3	3
SNRPN	5	5
PPP6C	3	3
ARFIP2	3	3
PDPR	5	5
P4HA2	4	4
ZNG1A	3	3
PPP3CA	4	4
ACACA	7	7
SESN2	4	4
SCAF8	4	1
FASTKD5	5	5
PPAN-P2RY11	4	4
ERAL1	3	3
NDRG3	3	3
ECH1	2	2
ABRAXAS2	4	4
MAT2B	3	3
PREB	3	3
AASDHPPT	4	4
SLC7A1	2	2
PRKCD	5	5
PSME4	3	3
UBE3A	3	3
TTC27	4	4
SPECC1L	3	3
WDR6	5	5
WDR3	4	4
PRPF39	4	4
MRPL4	3	3
IVNS1ABP	4	4
LTN1	4	4
PARVA	3	3
MAP1S	6	6
ATG4B	3	3
UBE4B	4	4

REPS1	3	2
PDK1	2	2
ELP2	6	6
FBXL18	3	3
OGFR	3	3
MVK	3	3
SHTN1	5	5
EXOC5	4	4
ITGA5	2	2
ARPC1A	3	3
ABLIM1	3	3
RPL17-C18orf32	3	3
OBI1	4	4
ACAD11	5	5
NELFCD	4	4
NOL10	6	6
STUB1	3	3
BAG3	6	6
BRAT1	2	2
RTF1	5	5
PIP5K1A	3	2
NIF3L1	4	4
TBK1	3	3
UBXN4	1	1
TLE4	4	2
SLC1A4	3	3
PPOX	3	3
NARS2	5	5
WDR4	3	3
INTS3	5	5
P3H1	5	5
ATG5	2	2
POLA2	3	3
CCNK	3	3
PAPSS2	4	4
AKR1A1	3	3
METTL13	5	5
ETFA	3	3
SRRM1	2	2
RANBP6	4	1
DYNC1LI2	2	2
HSD17B12	3	3
CD2BP2	4	4
KIF2C	5	4
RNF214	4	4
MRPS9	2	2
FAH	4	4

MRPL38	3	3
SRSF7	4	3
IRGQ	3	3
DNAJC8	3	3
SGTA	3	3
ATAD1	3	3
BCL2L13	4	4
RPL19	2	2
INTS11	3	3
IVD	5	5
DDX24	6	6
TPX2	5	5
MRPS5	3	3
SCP2	3	3
ARHGAP17	5	5
ARFGAP1	3	3
NDUFAF7	5	5
CCNT1	3	3
RUFY1	6	6
TARBP1	3	3
CDC42BPA	5	5
PRIM2	5	5
M6PR	1	1
CIAO1	3	3
CRLF3	2	2
DCAF7	3	3
SHOC2	4	4
ARF3	4	2
SRSF1	4	4
LNPK	3	3
QTRT1	3	3
TRMT13	3	3
GABPA	2	2
MPRIP	4	4
MARK2	4	2
CCZ1	3	3
PLIN2	3	3
MFAP1	2	2
RRS1	5	5
TCF25	6	6
VWA8	4	4
LRRFIP1	3	2
SERPINF1	3	3
DPP9	6	6
NOP9	3	3
MSTO1	3	3
GLOD4	3	3

GLO1	5	5
YME1L1	3	3
EIF5A	4	4
ANAPC5	3	2
XPNPEP3	4	4
RPL14	4	4
CHTF18	4	4
LANCL1	4	4
QNG1	3	3
ELP3	4	4
RING1	3	2
RPS14	3	3
SQSTM1	3	3
AP3B2	6	2
FBXO7	2	2
CHD2	3	2
PRORP	5	5
DDRGK1	2	2
IRF2BP2	2	2
NUP188	5	5
ALDH1A2	4	2
SLC16A1	3	3
AKT1	4	3
TRIM24	4	4
STIM1	4	4
COIL	4	4
TBC1D13	4	4
NR2F2	3	1
ASCC3	3	3
SATB2	3	3
LAMP1	4	4
MTMR6	3	3
HIRIP3	2	2
LAMP2	3	3
FASTKD2	3	3
RBBP5	6	6
VPS35L	2	2
FKBP15	3	3
LRRC41	2	2
LYN	5	4
GTF3C3	5	5
HSPBP1	4	4
GNAO1	2	1
THOC5	2	2
LEMD2	3	3
COL1A2	5	5
TUBGCP3	6	6

ERC1	4	4
PCID2	2	2
ATL2	2	2
HSDL1	3	3
SLC38A2	3	3
NAF1	2	2
HEATR6	2	2
ARMC6	2	2
GCLC	3	3
EXOC3	5	5
CAB39	3	2
LIMD1	2	2
MTCH2	2	2
GGA1	3	2
SCRN1	5	5
RADX	3	3
TCAF1	3	3
CXADR	3	3
HLA-B	3	1
PSMB5	2	2
GNAQ	4	1
KIF3A	5	5
GNA11	4	1
HMGCS1	3	3
RFC4	3	3
CDC42EP1	3	3
PDLIM7	3	3
ASPSCR1	3	3
PARK7	4	4
RPA2	4	4
CYB5B	2	2
H1-0	2	2
ERO1B	3	1
NCKIPSD	3	3
VPS33A	3	3
GGA2	3	2
MAN2B1	3	3
DUT	2	2
PLD3	4	4
SNRNP40	3	3
EXOC2	3	3
MCCC1	3	3
EIF6	2	2
ANAPC4	1	1
IQGAP3	2	2
KNTC1	4	4
DHX8	5	3

NGLY1	2	2
RPS25	3	3
ANK3	5	5
RCL1	5	5
USP24	4	4
CHEK1	4	4
PARS2	3	3
RAI14	4	4
FOXK2	3	2
MAPK6	3	3
CLGN	3	2
PPIB	4	3
POLR1C	3	3
RPRD2	3	3
CLCC1	3	3
TMEM205	2	2
TRA2A	2	2
CC2D1B	2	2
PPP4R2	5	5
FAM114A2	3	3
HMOX2	2	2
UTP14A	3	3
SREK1	2	2
MTHFD2	2	2
FBXO3	2	2
PWP1	2	2
MID1	4	4
LPL	1	1
NAA35	4	4
NAA10	2	2
B4GAT1	3	3
EXOSC9	3	3
IKBIP	3	1
CLPTM1L	3	3
MPP2	4	2
NUP37	2	2
HLA-C	2	1
TLK1	2	2
TMX3	3	3
PHF6	4	4
URB2	5	5
ACSF2	3	3
WASF1	2	2
COPS6	2	2
PITPNB	3	3
GCFC2	4	4
ITSN1	2	2

NUDT21	3	3
NKAP	3	3
NEDD8	3	3
PI4KB	2	2
SAP30BP	3	3
ARF4	4	2
TRA2B	2	2
RETREG3	2	2
ACSL1	4	4
MAPKAPK2	2	2
ARID3B	3	3
LTBP1	2	2
PCCB	3	3
WDR5	1	1
TBCB	4	4
ARMH3	3	3
FIBP	3	3
SMAP1	2	2
H2BC18	3	3
RPS27	2	2
TMTC3	3	3
UTP20	4	4
DHCR24	2	2
EIF4H	2	2
KIF1A	3	3
FLAD1	3	3
GTPBP6	2	2
GPR89A	3	3
SERPINC1	4	4
MDC1	1	1
COL1A1	3	3
L2HGDH	2	2
SEC13	1	1
POLR2A	5	5
SNX4	3	3
RRAGC	3	3
EFL1	4	4
PABPC1L2A	4	1
TPD52L2	2	2
PRKD2	2	1
YTHDC1	6	6
PRC1	4	4
CLPTM1	3	3
PWP2	5	5
VPS51	3	3
ODR4	2	2
RAF1	4	2

RAD23A	4	2
MPP1	3	3
LRRC1	3	1
WDR36	3	3
ARHGDIA	4	4
CHAMP1	4	4
FUCA2	4	4
CDK12	3	3
CBX3	1	1
RABGAP1	2	2
HPS6	2	2
HDAC8	2	2
RPP40	3	3
WDR43	3	3
SLC25A1	3	3
YTHDC2	4	4
RIOK1	3	3
SNRPA1	2	2
TMED9	2	2
ACADSB	4	4
EED	4	4
UCHL1	3	3
CPOX	3	3
TMX1	3	3
DTNBP1	3	3
TASOR	3	3
LRATD2	2	2
STX7	2	2
HIP1	1	1
PCYT1B	2	2
ARL6IP5	5	5
RHOT2	2	1
DNAJC3	6	6
TRAF4	4	4
NRBP1	3	3
GNS	4	4
COQ6	2	2
TMEM165	1	1
FSD1	2	2
PPP1R10	5	5
MARK3	3	1
ZNF598	3	3
POFUT2	3	3
FKBP8	1	1
RBM45	5	5
PYCR1	2	2
RPS11	5	5

TBL1X	4	1
URB1	2	2
C4B	3	3
NPC1	1	1
TMEM263	3	3
VTN	2	2
DUS3L	3	3
RPS20	2	2
B4GALT1	2	2
RRP15	2	2
TMEM209	1	1
IRF3	2	2
AKR1B15	2	1
ERCC4	2	2
BRCC3	1	1
EXOC8	3	3
RPL11	2	2
HEXB	4	4
VPS16	2	2
CACTIN	2	2
IRF2BP1	2	2
GBA1	4	4
THUMPD3	3	3
ATP1B1	3	3
BIRC2	3	3
RPL24	2	2
CLN5	3	3
NT5DC3	2	2
ANKRD13A	2	2
XYLB	2	2
CCDC86	3	3
PRPF38B	3	3
COG1	5	5
ITGA2	1	1
EPN1	3	3
VAPB	1	1
CSNK2B	2	2
ACTR10	3	3
RPS23	2	2
AKAP17A	3	3
USP22	2	2
PRIM1	3	3
ATP6AP1	6	6
CTSD	3	3
RPL27A	2	2
MICALL1	3	3
NCK1	3	3

SLC39A7	1	1
TRAPPC12	3	3
HACL1	4	4
PSMB6	3	3
PTPA	3	3
SELENOI	2	2
PROCR	3	3
THBS3	3	2
BAIAP2L1	2	2
ROCK2	4	2
USP19	2	2
RIF1	2	2
ASMTL	2	2
MED15	2	2
PIK3R1	2	2
MSI1	2	1
TTC1	1	1
MAP2K4	3	3
UBAP2	2	2
CC2D1A	4	4
HLA-A	2	1
FYN	3	1
NR2F1	3	1
DVL2	3	3
TRAF6	3	3
MAPK14	3	3
P3H4	1	1
MAP3K7	2	2
SMAP2	3	3
PHAX	2	2
NUF2	2	2
SAAL1	2	2
RBM33	2	2
PPIL2	2	2
RPL13A	4	4
UCHL3	2	2
HUWE1	2	2
EBP	1	1
HS1BP3	1	1
RBMXL3	2	1
WASHC4	2	2
DDX11	2	2
GHITM	2	2
PPA2	2	2
MTAP	1	1
SKIC2	4	3
INTS14	2	2

MPHOSPH10	3	3
CCNB1	3	3
ETFDH	2	2
NCAPD3	2	2
TIPIN	1	1
COG2	1	1
EIF2B5	2	2
RMND5A	1	1
ORC3	3	3
NRF1	2	2
RRBP1	4	3
RPAP1	1	1
POLR3B	2	2
PDXK	2	2
ZNF207	3	3
FDPS	3	3
ME1	2	2
RPL29	2	2
TSFM	3	3
ARHGAP22	3	3
HBA1	2	2
TRUB1	3	3
MYL6B	3	1
MAEA	2	2
RRP9	3	3
RBM42	3	3
REXO4	2	2
TOE1	3	3
ATP6V1C1	3	3
SRSF3	2	1
ACSF3	2	2
HPF1	2	2
SRFBP1	1	1
AP3M2	4	1
TMEM214	2	2
ULK3	3	3
CDS2	1	1
PSMB1	2	2
OGFOD1	2	2
GPATCH11	3	3
FARS2	2	2
RSRC2	3	3
MYL1	3	3
AUP1	2	2
ZYG11B	1	1
RANBP10	2	2
U2AF1L4	1	1

NCAM1	2	2
SEC62	2	2
ZNF24	4	4
MAZ	1	1
PPIP5K2	3	3
ARPC1B	2	2
FHOD1	3	3
TRMT6	3	3
ANKRD28	1	1
WDR70	3	3
THAP11	3	3
RPS28	2	2
PRKD3	2	1
GAK	2	2
ZC3HC1	2	2
DHRS7	2	2
RABEP1	2	2
HDAC6	2	2
TRAPPC8	2	2
LASP1	2	2
DPH2	1	1
GXYLT1	2	2
KNG1	1	1
JMJD6	2	2
BAG4	2	2
EIF2B1	2	2
UBE2Q1	3	3
CSNK1G3	3	3
TFB1M	2	2
RMDN3	2	2
NFXL1	2	2
NCBP3	2	2
ZCCHC3	3	3
GNL3L	3	3
RAP1GDS1	3	3
RALY	2	2
CERS2	1	1
CERT1	2	2
GFUS	1	1
MAGED4B	2	1
DHX40	2	2
TRNT1	2	2
SLC35B2	2	2
TSSC4	1	1
CD276	2	2
GNA13	2	2
NBN	3	3

CEP43	2	2
TRIM65	1	1
EGLN1	3	3
RHOT1	2	1
GGCX	2	2
PIP	2	1
SMARCD1	3	2
ITIH3	3	2
RABGGTA	2	2
CNOT10	2	2
CDC7	2	2
ESS2	1	1
PDPK1	3	3
TTC4	2	2
NLRX1	3	3
POLR1A	3	3
REPIN1	2	2
PCOLCE	2	2
PBK	2	2
NOL7	2	2
MBNL1	2	2
SCLY	2	2
BAZ1A	3	3
PSMD8	2	2
MEN1	2	2
BABAM2	2	2
YIPF5	1	1
ANP32A	3	1
WWP2	2	2
FUCA1	3	3
PTPRF	2	2
COG6	2	2
MOCS3	1	1
STRIP1	2	2
PSPH	2	2
F2	2	2
NHERF1	3	3
CRAT	3	3
PAAF1	2	2
SURF6	2	2
HP	1	1
SUGP2	2	2
TAMM41	1	1
H4C14	2	2
STK10	2	1
RB1	2	2
RBM15B	3	2

OXA1L	2	2
LRP1	2	2
SLC39A14	1	1
PBDC1	4	4
NAGK	2	2
PPP6R1	1	1
MKRN2	2	2
PIH1D1	2	2
GMPPB	2	2
APOA4	1	1
ADD2	3	1
XAB2	3	3
EZH2	1	1
FBLN1	1	1
DDTL	1	1
ISOC1	1	1
VPS26B	2	1
PALLD	2	2
HSPA13	3	3
C2CD5	3	3
CARNMT1	2	2
MAP1B	2	2
SRPK2	3	2
RER1	1	1
RACGAP1	3	3
VPS52	2	2
KHDRBS3	3	1
NEMF	2	2
INPP5K	1	1
CLIP1	3	3
PDK3	5	5
DCXR	3	3
PNKP	1	1
XPO6	3	3
MIF	1	1
TDRKH	1	1
OS9	2	2
HLCS	3	3
SMG9	2	2
ALDH4A1	2	2
ARAP1	2	2
ARHGEF7	2	2
RPS5	2	2
XRCC4	1	1
DDX10	3	3
PAK1IP1	2	2
RANBP9	2	2

GPSM1	2	2
LIPA	1	1
ALKBH5	2	2
KAT7	1	1
SBDS	2	2
TMEM33	2	2
PRKCI	1	1
OCIAD1	1	1
TRIM32	1	1
INTS8	2	2
FOCAD	3	3
UFSP2	1	1
AURKB	1	1
WDR46	1	1
UPF2	3	3
NUB1	4	4
TOX4	2	2
LIMS1	1	1
CAB39L	2	1
VRK3	2	2
TRAFD1	1	1
AATF	1	1
ALG5	2	2
RPS17	1	1
RAB5C	2	2
CHMP7	2	2
GYS2	1	1
EXOC6	3	3
GPT2	4	4
GATD3	1	1
RPL22	1	1
TMED10	1	1
SOD1	2	2
ATXN3	2	2
MSI2	2	1
POMK	1	1
RFT1	2	2
RNMT	3	3
CALM3	1	1
GPAA1	2	2
SLC38A1	2	2
TAF4	1	1
CPT1A	3	3
ECI2	1	1
TBC1D10B	2	2
ALG6	1	1
ERP29	2	2

CRTAP	3	3
UBXN7	3	3
BPNT2	1	1
METTL8	1	1
THNSL1	2	2
TM9SF4	2	1
TTC5	1	1
MIPEP	2	2
LNPEP	2	2
IDI1	2	2
PDHX	2	2
TAF6L	1	1
SAR1A	1	1
TRIP4	2	2
RARS2	2	2
AMOTL1	2	1
LACTB	2	2
OSGEP	1	1
BCAT2	1	1
AMY2B	1	1
YY1	2	2
PSMF1	1	1
PTER	4	4
WDR75	1	1
EIF4E	2	2
TTF2	1	1
PTGFRN	2	2
NUCB1	2	2
IGLL5	1	1
COQ9	1	1
PGM2L1	2	2
SPCS1	1	1
CWC22	3	3
SNX32	2	1
ADPRS	2	2
SERPIND1	1	1
INPP1	2	2
PRUNE1	1	1
ST3GAL1	1	1
NISCH	2	2
TUBGCP4	2	2
TTK	1	1
ETFB	2	2
INTS6	2	2
PTGR3	1	1
MICU2	1	1
YES1	3	1

DNTTIP2	2	2
ASCC1	3	3
SELENOO	1	1
CORO1A	3	2
TRABD	1	1
DDX55	1	1
AKT2	3	2
EPB41L5	1	1
PTDSS1	1	1
BIN1	1	1
MAU2	1	1
PARG	1	1
PREPL	2	2
COG8	2	2
CZIB	2	2
OSBPL1A	2	2
LARP4	1	1
COL6A1	2	2
DECR1	1	1
PI4KA	3	3
CDC20	2	2
ECSIT	1	1
SYPL1	1	1
RFX5	1	1
GTF2E1	2	2
NEDD4	2	2
CKM	3	1
CBX8	3	3
CYCS	2	2
ZC3H4	1	1
INTS9	2	2
TIMELESS	1	1
RALYL	2	2
REPS2	2	1
HAUS6	1	1
MRPL3	2	2
ICMT	2	2
KIF23	1	1
SASS6	2	1
RAD54L2	1	1
WDR59	1	1
MVB12A	1	1
PIGK	4	4
CA8	1	1
RAB35	1	1
DDX59	1	1
PRRC2C	2	1

COG3	3	3
UTP6	2	2
GMPR2	2	2
DDX31	1	1
FAHD2A	1	1
SEMA3E	2	2
CCDC22	3	3
MED25	1	1
IFT74	1	1
GNPDA1	3	3
PEDS1-UBE2V1	2	2
ATP6	2	2
SNX18	1	1
HAX1	2	2
PAWR	1	1
CTCF	2	2
NDUFV3	3	3
CHURC1-FNTB	2	2
HS2ST1	2	2
KRT78	1	1
ANKLE2	1	1
NIT2	1	1
BMP1	2	2
ATP5MF-PTCD1	1	1
NDRG2	1	1
DCAF1	2	2
BCAT1	2	2
SRSF10	1	1
RIPK1	1	1
NMT2	2	1
ERH	1	1
AS3MT	1	1
NUDCD3	1	1
CYRIB	1	1
DIPK2A	2	2
FDFT1	1	1
BAG5	1	1
HIBCH	2	2
KLHDC10	1	1
NDC80	3	3
S100A8	1	1
MTF2	1	1
GTF3C1	3	3
STAT5A	2	1
ATP6V0D1	2	2
NDC1	3	3
ANAPC1	2	2

BCR	1	1
NSUN5	2	2
C9	1	1
PRPF4B	1	1
GTF2A1	1	1
SLC39A10	2	2
SNRPD3	1	1
NPTN	1	1
CEPT1	1	1
F11R	1	1
TBC1D9	1	1
RPS6KA5	1	1
ECT2	2	2
PAN2	1	1
DOLPP1	1	1
SMYD3	2	2
PON2	1	1
ADPGK	1	1
RBBP6	1	1
CARS2	2	2
IGF2R	3	3
PALD1	3	3
BCL7A	1	1
LGALS3	1	1
MKI67	1	1
LMO7	2	2
HECTD1	2	2
MIB1	1	1
LRCH2	2	2
PDCD2L	1	1
DERL1	1	1
MAP7D1	1	1
HIP1R	1	1
NID1	1	1
DCAF13	1	1
DIMT1	1	1
H1-10	1	1
TBC1D24	2	2
PRPF38A	2	2
SRSF9	2	2
DHPS	1	1
RNF113A	2	2
SERPINA10	1	1
SCRN2	1	1
SEC61A1	1	1
IMPACT	1	1
BCAP31	1	1

RTN3	1	1
SDE2	1	1
ND5	1	1
NCAPH2	1	1
PACC1	1	1
LYZ	1	1
EIF3K	1	1
LPGAT1	1	1
GTPBP2	1	1
NOL11	2	2
CHDH	1	1
ND2	1	1
SSR1	2	2
VPS50	2	2
VPS37A	1	1
PALM	1	1
HAUS8	1	1
EML2	1	1
SLC2A1	1	1
USP8	1	1
METTL17	1	1
PHKB	1	1
SYVN1	1	1
DHX37	1	1
NEFL	2	1
WDFY1	2	2
TAOK1	1	1
ESF1	3	3
PHF10	1	1
MED14	1	1
TM9SF3	2	2
SCD	1	1
SLC33A1	1	1
POGLUT2	1	1
MAP7D3	1	1
EIF2B2	1	1
CENPB	1	1
EIF2AK4	1	1
UPF3B	2	2
RPS6KB1	2	2
KLC4	2	2
METTL14	1	1
FAM184B	2	1
SNAP47	1	1
GALNT7	1	1
GNPAT	1	1
PHC2	2	2

PKN1	1	1
LZTFL1	1	1
DAD1	1	1
FAU	1	1
APOH	1	1
CDK19	1	1
RPL38	1	1
LRPAP1	1	1
CRBN	1	1
FNBP4	1	1
DENND6A	2	2
RABGEF1	1	1
RAB11FIP1	2	2
UBR7	2	2
CDKN2AIP	1	1
SLC29A1	1	1
ARRB2	2	2
MVD	1	1
WDR41	1	1
RPF1	1	1
CCDC93	1	1
RPF2	1	1
MBOAT7	1	1
CNNM4	1	1
PEX5	1	1
INTS2	1	1
SMN1	1	1
CLIC4	3	1
SKIC8	1	1
SUSD5	1	1
FADS1	1	1
WASF2	2	2
TNKS1BP1	1	1
ATG9A	1	1
DERL2	1	1
THOC3	2	2
NUDT12	1	1
IQCB1	1	1
SORT1	1	1
YOD1	1	1
HIBADH	1	1
TEAD1	1	1
TCF7L2	1	1
PDP1	1	1
PHKG2	1	1
QRSL1	1	1
NIPSNAP1	3	3

PPP2R5E	1	1
FOXA2	1	1
RNF25	1	1
TPBG	1	1
RABL6	2	2
GLE1	1	1
TINAGL1	1	1
FIGNL1	1	1
GTF2H2C	1	1
ABI2	1	1
RASA1	1	1
DPH5	1	1
COPE	1	1
EXOSC4	1	1
DLG1	1	1
VRK2	2	2
NIBAN2	1	1
RAD9A	1	1
BTRC	1	1
POLR1G	1	1
MRPL39	1	1
TMED1	1	1
SGSH	1	1
HIF1AN	1	1
TSR3	1	1
USP28	1	1
METAP1	2	2
GCAT	1	1
DACH1	2	2
SKP2	1	1
MALT1	1	1
NAPA	1	1
CASC3	1	1
AFTPH	1	1
MINDY3	1	1
AFP	1	1
NFKB2	1	1
ZWILCH	1	1
SERPINA1	2	2
CD47	1	1
NPM3	1	1
ACSS2	1	1
MYO3B	1	1
NAXE	1	1
POLE2	2	2
MAN1B1	1	1
GSTM3	1	1

TOP3B	1	1
SUCLG1	1	1
TM9SF2	2	1
CLU	2	2
FHL1	1	1
LPP	2	2
TSEN34	1	1
MAP2K7	1	1
MTCH1	1	1
AGTPBP1	1	1
MTIF2	1	1
LAMB1	2	2
ALG11	1	1
AKAP8	2	2
OTUD4	1	1
ATP5PO	1	1
NEU1	1	1
ARHGAP35	1	1
GDPGP1	1	1
TSC22D1	1	1
FAM120B	1	1
SELENBP1	1	1
RNF123	1	1
FLG2	1	1
PIK3R4	1	1
HAUS5	1	1
SMYD5	1	1
CD63	1	1
SEC16A	1	1
ACOX1	1	1
NIPBL	2	2
COX1	1	1
GPATCH4	1	1
MARS2	2	2
SLU7	1	1
NEPRO	1	1
YAP1	1	1
CNOT11	1	1
UCKL1	1	1
STX18	1	1
ALG10	1	1
NAA30	1	1
ANAPC2	2	2
RAD18	1	1
UTP3	1	1
HMGB2	2	2
AFF4	1	1

SLC25A10	1	1
PPP4C	1	1
EXOSC2	1	1
ABHD12	1	1
SESN1	1	1
CAAP1	1	1
ADO	2	2
ZSCAN29	2	1
KLHDC4	1	1
BSDC1	1	1
ERAP1	1	1
VPS11	1	1
TRIM27	2	2
REL	1	1
VPS37C	1	1
ABCC1	1	1
GUF1	1	1
TDP2	1	1
TOR1AIP2	1	1
PXDNL	1	1
CCNA2	2	2
GOPC	1	1
DNASE1	1	1
RXRB	1	1
PGRMC1	1	1
COMT	1	1
ZMYM3	1	1
DCP2	2	2
CHD6	2	1
UNG	1	1
ARPC3	1	1
TBC1D9B	1	1
GPALPP1	2	2
PSMB2	1	1
COG7	1	1
DOCK1	1	1
CYP17A1	1	1
GUSB	1	1
TSNAX	1	1
PHF8	1	1
CREB1	2	2
UBE4A	1	1
SIRT1	1	1
INPPL1	1	1
PRMT6	1	1
SMAD4	1	1
LUM	1	1

SLC30A9	1	1
ZMYM4	1	1
EVI5L	1	1
CDKAL1	1	1
NOTCH2	1	1
RDH11	1	1
PAK4	1	1
EXOSC8	1	1
SPTLC2	1	1
APRT	1	1
IFT56	1	1
DIRAS2	1	1
MYLK3	1	1
CRK	1	1
PACSIN3	1	1
PCYOX1L	1	1
PRUNE2	1	1
DCPS	1	1
LDB1	1	1
BLM	2	2
GTF2H4	1	1
MOCOS	1	1
BNIP2	1	1
SLC12A6	1	1
ATP13A1	1	1
SYP	1	1
RBPJL	1	1
SCARB1	1	1
WASL	1	1
RAD51C	1	1
PAFAH1B2	1	1
PATL1	2	2
KATNA1	2	2
KIF16B	1	1
MAPKAP1	1	1
UBR4	1	1
CDK16	1	1
MIER1	1	1
MTERF2	1	1
APLP2	1	1
MYO1E	1	1
AKR1E2	1	1
WFS1	1	1
TBC1D23	1	1
MPST	1	1
MBD3	1	1
NF2	1	1

LSM5	1	1
FARP2	1	1
DPM1	1	1
CCS	1	1
CRTC2	1	1
SMAD9	1	1
CDK13	1	1
PML	1	1
UTP4	1	1
PLA2G4A	1	1
CYTB	1	1
HERC6	2	2
MELK	1	1
HSPE1-MOB4	1	1
EBF1	1	1
FRK	1	1
BICD1	1	1
KPLCE	1	1
CCDC18	2	2
GRK2	1	1
CCNY	1	1
MRTO4	1	1
OPTN	1	1
IMP4	1	1
SDHB	1	1
PGAM5	2	2
GAN	1	1
RAB15	1	1
IPO13	1	1
PTDSS2	1	1
SAP130	1	1
RPL34	1	1
STX5	1	1
TMED8	2	2
ACSS1	1	1
TXLNB	2	2
GALNT1	1	1
VMP1	1	1
FADS2	1	1
MAP3K3	1	1
PIK3C3	2	1
PSMB7	1	1
TMEM30A	1	1
CDC40	1	1
TCP11L1	1	1
AMFR	1	1
MAPT	1	1

DNAJC16	1	1
SPINDOC	1	1
ASB6	1	1
OXSM	1	1
PLEKHG4	1	1
MPHOSPH8	2	2
UTP18	1	1
COX2	1	1
LSM4	1	1
MAP3K4	2	2
MFN2	1	1
IKBKB	1	1
MTPAP	1	1
ECI1	1	1
TCIRG1	1	1
TOMM34	1	1
CTDSPL2	1	1
NEMP1	2	2
SS18	1	1
PEX14	1	1
RCC1L	1	1
H2AX	1	1
EIPR1	1	1
SERPING1	1	1
BABAM1	1	1
KNOP1	1	1
CCDC51	1	1
SPG7	1	1
TRAM1	1	1
LMF2	1	1
CALR3	1	1
PIGU	1	1
WBP2	2	2
KCNH5	1	1
MNT	1	1
GLDC	1	1
CEP104	1	1
ADA	1	1
TRMT2A	1	1
SFXN4	1	1
PDXP	1	1
ANKRD34B	1	1
ZNF266	2	1
MIDEAS	1	1
SRR	2	2
ALG1	2	2
POLR1E	1	1

DCAF8	1	1
MMS22L	1	1
HBG1	1	1
ERI1	1	1
TACC3	1	1
QPRT	2	2
PLCH1	2	2
SH3BP4	1	1
DDAH1	1	1
MTERF3	1	1
SMG8	1	1
OXNAD1	1	1
ELAVL2	2	1
OTULIN	1	1
MICU1	1	1
H3C1	1	1
KIAA2012	1	1
FBXL19	1	1
SMG5	1	1
NAGLU	1	1
TRIM41	1	1
TMPRSS13	1	1
GLIPR1	1	1
ELOVL5	1	1
HABP2	1	1
MEX3D	1	1
NOP53	1	1
BRPF1	1	1
FKBP9	1	1
LRRIQ1	1	1
DOCK11	1	1
TSPAN3	2	2
UBR2	1	1
CLTA	1	1
CNTRL	3	3
SLC25A25	1	1
ANO6	1	1
MORC3	1	1
BTN1A1	1	1
EMSY	1	1
RNF17	1	1
TTC28	1	1
UBAC2	1	1
CHST9	1	1
ZNHIT6	1	1
HOOK1	1	1
SEPTIN3	1	1

ARMC9	1	1
ARID1A	1	1
FASTKD3	3	3
BPIFA3	1	1
MDN1	1	1
DNAJB6	1	1
PAPOLG	2	1
ERGIC3	1	1
GRB10	1	1
TTL	1	1
BTF3	1	1
AP2S1	1	1
C2orf16	1	1
EI24	1	1
CNOT2	1	1
SCMH1	1	1
ACAP2	1	1
SLC35E1	1	1
TBC1D5	1	1
SCRN3	1	1
MORF4L1	1	1
MRPS30	1	1
PTGR2	1	1
CBX2	1	1
TXNL1	1	1
RNF139	1	1
MCOLN3	1	1
AIP	1	1
AVEN	1	1
CEP97	1	1
ZC3H7A	1	1
IGHMBP2	2	2
CLCN3	1	1
MAP2K5	1	1
GFM2	1	1
ARSA	1	1
DCD	2	2
ELOA	1	1
FOXRED1	1	1
CFAP61	1	1
NCEH1	1	1
TAX1BP1	1	1
TLCD3A	1	1
MOXD1	2	2
TBC1D22A	1	1
CGN	1	1
AAK1	1	1

ING2	1	1
RABGAP1L	1	1
FAM78B	1	1
FAM25G	1	1
KATNB1	1	1
AHNAK	2	2
DIS3L2	1	1
VWA5B1	2	2
VAPA	1	1
NALCN	1	1
FN3KRP	1	1
GTPBP3	1	1
PKP4	1	1
WIZ	1	1
ARMC8	1	1
HSF2	1	1
PPP2R5B	1	1
INTS10	1	1
SETD4	1	1
WDR37	2	2
ZNF746	1	1
PIAS4	1	1
ACTR8	1	1
GNB1L	1	1
OBSCN	2	2
ANKHD1-EIF4EBP3	1	1
GUCY1B1	1	1
DLGAP5	1	1
LDHAL6B	2	1
ZNF622	1	1
ZNF567	1	1
CNTNAP4	1	1
ERBIN	1	1
TSSK4	1	1
IFNG	1	1
TF	1	1
CDIPT	1	1
NIN	2	1
KLK11	1	1
SH3PXD2A	1	1
ARG1	1	1
MYBPC3	1	1
FOXP1	1	1
RIPPLY1	1	1
CLEC16A	1	1
RENBP	1	1
VIPAS39	1	1

IL23R	1	1
PURB	1	1
TBCK	1	1
MYO1H	2	2
SLC20A1	1	1
SEMA4F	2	1
PAXBP1	1	1
LTF	1	1
SLFN11	1	1
SHPRH	1	1
RPL30	1	1
RSBN1L	1	1
EXD3	1	1
EFCAB14	1	1
ASXL3	1	1
B4GALT7	1	1
SEPTIN10	1	1
TRAF2	1	1
SLFN14	1	1
HMCN1	1	1
PRKCA	1	1
VPS37B	1	1
SUMO1	1	1
DNA2	1	1
ITGA2B	2	2
ATRIP	1	1
MRM3	1	1
TMEM132C	1	1
CEP164	1	1
DUOX2	2	2
FRMPD2	1	1
ITIH4	2	2
SNX25	1	1
RPL28	1	1
LEKR1	1	1
C5orf22	1	1
SNX7	1	1
LOXHD1	1	1
RPAP2	2	2
HPX	1	1
GP5	1	1
MCL1	1	1
GK	1	1
POMGNT1	1	1
ATG4C	1	1
LCT	1	1
ABCA2	1	1

CCDC171	1	1
CCDC88C	1	1
FLT1	1	1
FAM186B	1	1
NECAP1	1	1
GNPTAB	2	2
PARD6G	1	1
ZNF585B	1	1
DMD	1	1
GCNT3	1	1
DNAJC21	1	1
TC2N	1	1
ZZEF1	1	1
DDHD1	1	1
ATP2C2	1	1
CCDC158	1	1
KIF12	1	1
MBTD1	1	1
PPFIA3	1	1
CENPF	2	1
UBE2M	1	1
SYNE3	1	1
SERF1A	1	1
POLR1F	1	1
BUD13	1	1
TSPEAR	1	1
NWD1	1	1
RABGAP1L	1	1
CDK18	1	1
MINPP1	1	1
TNRC6A	1	1
XDH	1	1
ACY1	1	1
ULK1	1	1
ASCC2	1	1
EXOSC7	1	1
GTF2H1	1	1
YDJC	1	1
RPS26	2	2
DAPK3	1	1
C10orf55	1	1
PAN3	1	1
SETX	1	1
ATF7IP2	1	1
NLE1	1	1
MAF	1	1
PUS7L	1	1

KRT23	1	1
TBC1D2	2	2
UTP15	1	1
DMAP1	1	1
TENM2	1	1
NYNRIN	1	1
NAV1	1	1
LSR	1	1
BRD7	1	1
FGA	1	1
TBC1D8B	1	1
IFIT5	1	1
ARHGEF40	1	1
VPS72	1	1
FTH1	1	1
RASAL2	2	2
RPL36	1	1
CHRAC1	1	1
ST3GAL4	2	2
HYDIN	1	1
CAPNS1	1	1
CDCA5	1	1
EYA2	1	1
BIRC6	2	2
SORBS1	1	1
PCDHA3	1	1
HPS5	1	1
ELL3	1	1
ERCC3	1	1
PXYLP1	1	1
NFATC2IP	1	1
CCL5	1	1
HAS1	1	1
CDK5	1	1
TSGA10	1	1
RESF1	1	1
SLC30A5	1	1
NOL8	1	1
IREB2	1	1
FBN3	1	1
TNFAIP8L3	1	1
NBR1	1	1
ECEL1	1	1
RPIA	1	1
SMCO2	1	1
IFRD2	1	1
PRSS23	1	1

BPIFB2	1	1
CDY1	1	1
NAP1L5	1	1
CCDC141	1	1
PLXND1	1	1
A1BG	1	1
EVPL	1	1
KIF21A	1	1
AHSG	1	1
MYO1F	1	1
DDX49	1	1
EXD2	2	2
SH3BP5	1	1
HOXD9	1	1
DNHD1	1	1
USP30	1	1
FAM169A	1	1
MAST2	1	1
ROR2	1	1
FBXO10	1	1
HAUS4	1	1
ZNF618	1	1
E2F3	1	1
RPL35	1	1
TRAPPC13	1	1
PPP2R3A	1	1
WWC1	2	1
SRPX	1	1
HERC4	1	1
MYOF	1	1
SAP30	1	1
HERC2	1	1
PROS1	1	1
SNX29	1	1
MRPS18C	1	1
EMC2	1	1
TRO	1	1
DNAH8	1	1
LRRTM3	1	1
CENPE	1	1
USP36	1	1
PLCB4	1	1
BRDT	1	1
ARHGAP31	1	1
ITGB8	1	1
PPP1R15A	1	1
ZNF638	1	1

FN1	1	1
COL11A1	1	1
PCDH19	1	1
ITPR1	1	1
DNAAF1	1	1
FITM1	1	1
GABRG3	1	1
APBB1IP	1	1
GRHL3	1	1
LRP6	1	1
RAB18	1	1
EML6	1	1
C1S	1	1
OVOS2	1	1
CYP2C18	1	1
SMG6	1	1
SHANK3	2	1
TTN	5	4
WDR55	1	1
ARHGEF17-AS1	1	1
PATJ	1	1
LYRM4	1	1
LRRC15	1	1
UTY	1	1
FHDC1	1	1
ICE1	1	1
C6orf132	1	1
TAOK3	1	1
LRRC14B	1	1
MYADM	1	1
UGT1A9	1	1
GNPTG	1	1
NEB	1	1
MAP9	1	1
RSPO3	1	1
NADSYN1	1	1
VPS13D	1	1
CC2D2A	1	1
MRPS21	1	1
PLCZ1	1	1
SPINT1	1	1
MGAT2	1	1
SETDB1	1	1
ANKRD39	1	1
PIK3R5	1	1
LAMA5	1	1
CNGB3	1	1

SLCO1B3	1	1
ZNF566	1	1
CFAP57	1	1
FNDC3A	1	1
PIAS2	1	1
MSL2	1	1
REXO2	1	1
ARHGEF18	1	1
FGD5	1	1
CALML3	1	1
SCARA5	1	1
ZNF550	1	1
UNC5C	1	1
THADA	1	1
BMP3	1	1
C16orf78	1	1
ICE2	1	1
ABHD5	1	1
DZANK1	1	1
C17orf99	1	1
PNMA8B	1	1
ASTN2	1	1
TMEM160	1	1
SETD2	1	1
DNAH1	1	1
ASH1L	1	1
TTC38	1	1
STON1-GTF2A1L	1	1
MACC1	1	1
USP6NL	1	1
SPIB	1	1
ART4	1	1
NUP210L	1	1
PPFIBP1	1	1
NCBP2	1	1
MPO	1	1
CEP135	1	1
FBXL16	1	1
BBS5	1	1
FAM166A	1	1
FSD1L	1	1
MED1	1	1
CCDC121	1	1
TDRD3	1	1
FBXO39	1	1
LARP6	1	1
POLR2F	1	1

ERAS	1	1
CYTH2	1	1
TNS3	1	1
C2orf72	1	1
LUZP4	1	1
PWWP3B	1	1
ZNF292	1	1
ZFHX3	2	2
KIF21B	1	1
CR1	1	1
CPLX3	1	1
CCDC34	1	1
CTIF	1	1
NFATC1	1	1
CGNL1	1	1
ONECUT1	1	1
ABHD17C	1	1
SYNE1	1	1
TUT4	1	1
FRMPD3	1	1
LRP2	1	1
PIWIL4	1	1
ADGRF4	1	1
OPN1MW	1	1
SLC25A28	1	1
WDR87	1	1
B3GAT2	1	1
HERC5	1	1
MYCBP2	1	1
ITGAV	1	1
SLC16A10	1	1
ZMAT2	1	1
TMEM132B	1	1
CKAP2L	1	1
OTUD7A	1	1
HEG1	1	1
FBXL20	1	1
RNF216	1	1
DPF1	1	1
MYO5C	2	2
WNT9A	1	1
ARHGAP29	1	1
PMS2	1	1
OBSL1	1	1
SENP2	1	1
TANGO6	1	1
PHACTR2	1	1

SEC22A	1	1
C19orf44	1	1
WDR72	1	1
SDHAF3	1	1
LY75-CD302	1	1
RNF170	1	1
ATP10A	1	1
TACC2	1	1
NTHL1	1	1
DNASE2B	1	1
SCN9A	1	1
GASK1B	1	1
ATF6B	1	1
SLC6A11	1	1
PLD5	1	1
CLEC4D	1	1
DGKA	1	1
OR52E2	1	1
RASAL1	1	1
MLH1	1	1
THSD7A	1	1
SLC13A3	1	1
DYM	1	1
CERK	1	1
COBL	1	1
GABBR1	1	1
TXNDC2	1	1
SLC4A10	1	1
LUZP2	1	1
SLC4A8	1	1
ARFGEF2	1	1
SPAG1	1	1
FAM135B	1	1
UVRAG	1	1
RGS18	1	1
RNF141	1	1
DNAAF10	1	1
UNC80	1	1
GPR119	1	1
ATAD5	2	2
USP35	1	1
DDX51	1	1
NLRP13	1	1
FGF17	1	1
LRP1B	1	1
HOOK2	1	1
DNAH7	1	1

POLK	1	1
C1QTNF7	1	1
WDR17	1	1
ENDOU	1	1
PRR14L	1	1
FOXL2NB	1	1
UACA	1	1
CAMKV	1	1
NAV3	1	1
GPR108	1	1
CEP295	1	1
UBR5	1	1
ZNF780A	1	1
TMC8	1	1
NAIP	1	1
NUAK1	1	1
MYBPC2	1	1
USP16	1	1
RIGI	1	1
RCHY1	1	1
DIXDC1	1	1
CUBN	1	1
PCF11	1	1
PPFIA1	1	1
TMCO3	1	1
NRXN2	1	1
CSTB	1	1
NBEAL2	1	1
ZNF532	1	1
SOX11	1	1
RIC1	1	1
TRPM7	1	1
PRAG1	1	1
OR4A15	1	1
FAM83E	1	1
SLC39A8	1	1
RAB13	1	1
SIPA1L1	1	1
LRP5	1	1
WDR7	1	1
ASAP2	1	1
ERFE	1	1
C12orf60	1	1
DLEC1	1	1
COL6A3	1	1
C2CD3	1	1
SPR	1	1

PCLO	1	1
HECTD2	1	1
SLC27A6	1	1
RAI1	1	1
PIKFYVE	1	1
NCCRP1	1	1
RASL10B	1	1
AGMAT	1	1
ZCCHC4	1	1
MLLT6	1	1
MYO3A	1	1
GIP	1	1
FOXM1	1	1
CCDC38	1	1
SPAG17	1	1
STK11	1	1
TLR7	1	1
PHLDA2	1	1
KIAA1614	1	1
TCAF2	1	1
MITF	1	1
CNNM2	1	1
ZNF526	1	1
ADAL	1	1
CXorf58	1	1
ZNHIT2	1	1
RAB1A	1	1
INF2	1	1
IGSF8	1	1
OR8G1	1	1
OR2AK2	1	1
ANPEP	1	1
SPTBN5	1	1
CCDC146	1	1
NALF1	1	1
CYP1A2	1	1
QRFPR	1	1
SH2D7	1	1
SLC4A2	1	1
HS3ST1	1	1
RAB40C	1	1
NBEAL1	1	1
EXOSC6	1	1
NPFFR2	1	1
NINJ1	1	1
COL14A1	1	1
STOML1	1	1

NCKAP1L	1	1
TMTC1	1	1

Figure 2L

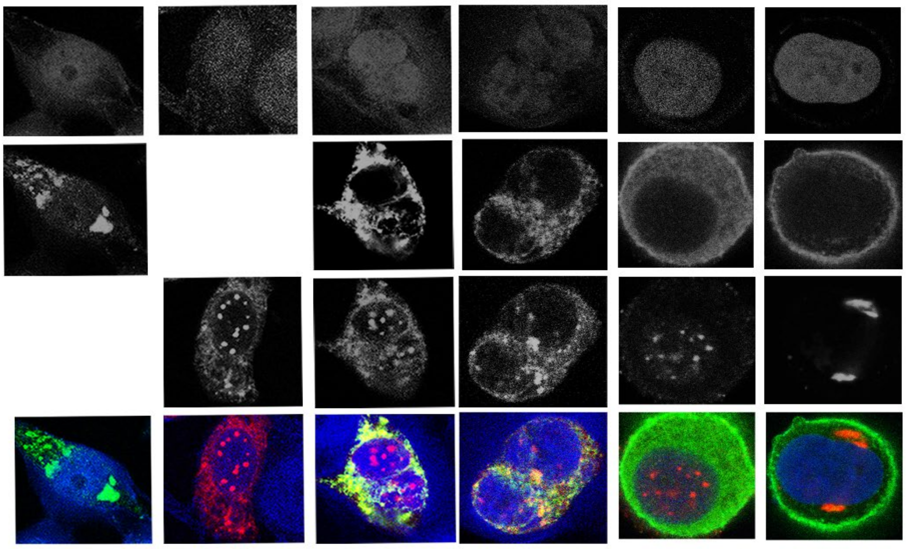

Figure 2M

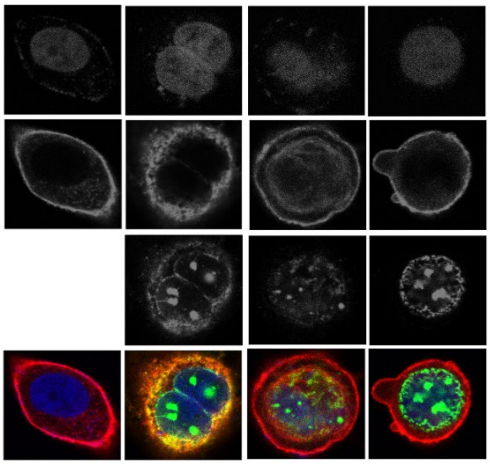

Figure 3D (Right)

h	EV			Myc-SPOP-OE		
0	1	1	1	1	1	1
1	0.755116804	0.736211162	0.508092325	1.12790575	0.866972039	1.406771414
3	0.598243336	0.685135788	0.430382252	1.165509963	1.014273046	0.887254295
5	0.147485594	0.503605567	0.293626569	1.009433464	0.773154243	0.442161246
7	0.055365396	0.316624691	0.089466138	0.533994836	0.485678062	0.221792469
9	0.00674626	0.006212747	0.009414998	0.04628791	0.062747988	0.052546957

Figure 4A

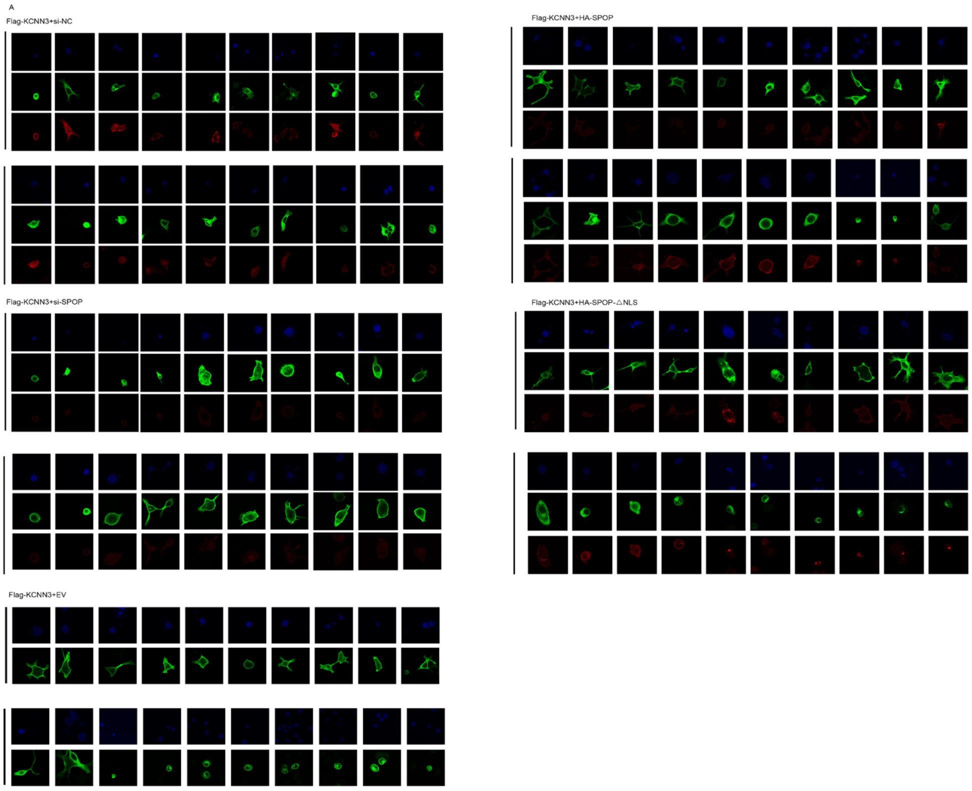
A
Flag-KCNN3+si-NC
Flag-KCNN3+HA-SPOP
Flag-KCNN3+si-SPOP
Flag-KCNN3+HA-SPOP-△NLS
Flag-KCNN3+EV

Figure 4B

Total KCNN3

Flag-KCNN3	Flag-KCNN3 +SPOP-KD	Flag-KCNN3	Flag-KCNN3 +HA-SPOP	Flag-KCNN3 +HA-SPOP-△NLS
119.459	166.537	131.822	190.821	178.496
129.466	210.619	174.74	183.002	162.61
170.405	185.891	141.408	106.291	113.496
109.212	202.306	183.345	196.833	183.048
190.283	190.37	166.254	119.218	162.303
180.114	192.686	140.78	110.624	169.706
128.096	184.083	161.743	118.87	172.981
238.834	138.786	170.494	164.827	183.66
178.243	195.794	178.868	159.155	159.749
153.271	135.113	166.64	105.161	158.741
204.968	197.57	169.783	170.628	165.943
136.128	203.086	178	201.821	139.115
157.144	174.178	163.137	193.434	179.61
201.664	180.831	177.893	193.434	148.762
199.346	137.005	157.752	153.491	155.143
160.979	181.865	135.597	157.917	120.31
174.699	164.461	157.774	169.006	173.65
142.02	171.015	132.639	163.168	144.827
212.001	131.343	131.784	169.18	120.719
198.237	168.199	186.56	179.288	171.513

Membrane KCNN3

Flag-KCNN3	Flag-KCNN3 +SPOP-KD	Flag-KCNN3	Flag-KCNN3 +HA-SPOP	Flag-KCNN3 +HA-SPOP-△NLS
15.397	73.611	71.527	60.503	60.815
26.882	71.145	67.335	48.588	54.154
53.191	68.013	50.469	35.544	32.201
18.929	76.985	75.109	54.814	64.382
49.537	77.953	65.514	43.695	56.185
47.365	78.884	72.688	30.582	65.892
38.979	65.3	67.365	48.483	66.417
63.254	60.994	72.421	53.763	69.971
52.176	78.721	72.416	57.432	57.86
52.094	62.132	76.454	32.124	55.457
66.939	84.772	72.542	61.731	55.599
34.464	83.117	71.118	67.126	16.991
46.442	72.846	81.524	69.912	51.541
48.101	81.114	79.416	67.488	38.902
63.231	73.893	73.495	46.121	54.087
55.061	83.354	77.591	51.693	48.945
53.299	68.802	68.766	52.446	51.608
51.31	72.047	67.209	50.242	7.66
72.603	73.619	63.139	41.148	29.33
74.112	77.01	78.796	62.689	48.528

Figure 4B				
Cytosol KCNN3				
Flag-KCNN3	Flag-KCNN3 +SPOP-KD	Flag-KCNN3	Flag-KCNN3 +HA-SPOP	Flag-KCNN3 +HA-SPOP-△NLS
104.062	92.926	60.295	130.318	117.681
102.584	139.474	107.405	134.414	108.456
117.214	117.878	90.939	70.747	81.295
90.283	125.321	108.236	142.019	118.666
140.746	112.417	100.74	75.523	106.118
132.749	113.802	68.092	80.042	103.814
89.117	118.783	94.378	70.387	106.564
175.58	77.792	98.073	111.064	113.689
126.067	117.073	106.452	101.723	101.889
101.177	72.981	90.186	73.037	103.284
138.029	112.798	97.241	108.897	110.344
101.664	119.969	106.882	134.695	122.124
110.702	101.332	81.613	123.522	128.069
153.563	99.717	98.477	125.946	109.86
136.115	63.112	84.257	107.37	101.056
105.918	98.511	58.006	106.224	71.365
121.4	95.659	89.008	116.56	122.042
90.71	98.968	65.43	112.926	137.167
139.398	57.724	68.645	128.032	91.389
124.125	91.189	107.764	116.599	122.985

Figure 4C

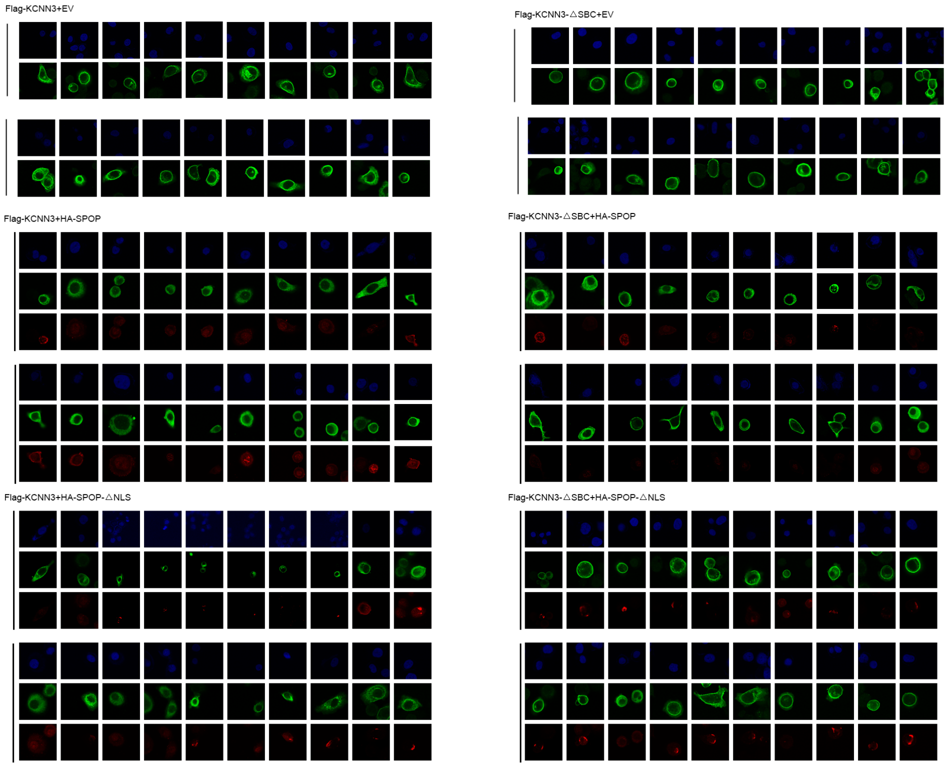
Flag-KCNN3+EV
Flag-KCNN3-△SBC+EV
Flag-KCNN3+HA-SPOP
Flag-KCNN3-△SBC+HA-SPOP
Flag-KCNN3+HA-SPOP-△NLS
Flag-KCNN3-△SBC+HA-SPOP-△NLS

Figure 4D

Total KCNN3					
Flag-KCNN3	Flag-KCNN3 +HA-SPOP	Flag-KCNN3 +HA-SPOP -△NLS	Flag-KCNN3- △SBC	Flag-KCNN3-△ SBC +HA-SPOP	Flag-KCNN3-△ SBC +HA-SPOP-△NLS
162.134	139.259	140.42	174.27	158.725	159.185
153.125	171.24	153.551	179.353	179.278	142.327
141.36	144.454	141.767	151.9	129.797	158.736
152.404	149.539	179.025	148.85	141.62	143.216
164.5	166.919	174.115	165.846	145.887	144.348
157.107	155.479	164.855	140.121	118.014	161.532
144.774	164.949	145.381	152.109	171.279	151.779
150.592	148.047	138.333	186.946	152.519	144.261
151.211	146.348	133.004	162.559	160.068	142.058
173.472	145.773	136.755	157.799	173.693	166.616
169.394	154.532	144.605	150.115	159.559	153.207
169.816	136.82	136.674	160.06	167.638	163.437
159.249	143.037	162	155.263	175.892	136.58
169.658	140.537	179.385	175.233	179.778	156.077
179.717	150.376	176.276	166.636	175.698	151.816
175.594	176.267	156.822	173.427	192.317	148.061
166.928	174.825	180.519	160.619	136.343	155.282
174.628	179.61	158.977	148.026	159.358	142.767
145.585	172.035	174.105	153.162	168.243	154.517
146.541	160.781	127.261	170.074	179.391	164.446

Membrane KCNN3					
Flag-KCNN3	Flag-KCNN3 +HA-SPOP	Flag-KCNN3 +HA-SPOP -△NLS	Flag-KCNN3- △SBC	Flag-KCNN3-△ SBC +HA-SPOP	Flag-KCNN3-△ SBC +HA-SPOP-△NLS
60.645	29.192	39.516	76.691	64.508	62.418
69.741	68.764	53.946	84.529	81.365	58.157
51.809	37.082	37.579	60.269	53.51	59.223
55.431	37.473	52.228	54.667	63.435	46.21
67.855	75.09	64.612	74.172	67.961	57.041
61.526	59.973	37.386	51.466	48.545	71.516
45.812	57.48	39.902	64.324	55.292	70.6
55.978	36.964	28.816	84.895	57.841	57.274
53.35	37.825	25.142	79.744	78.036	42.351
55.014	36.365	33.269	71.855	65.102	82.94
63.496	41.195	48.467	53.28	57.191	64.662
66.437	29.114	39.822	71.494	70.143	60.575
50.101	36.489	51.917	64.683	49.234	58.417
70.019	31.108	39.643	82.806	68.881	80.185
69.09	52.789	45.973	77.954	61.77	61.151
74.458	54.658	30.736	79.842	67.402	52.675
66.856	60.31	19.708	73.789	52.651	58.673

Figure 4D

Cytosol KCNN3					
Flag-KCNN3	Flag-KCNN3 +HA-SPOP	Flag-KCNN3 +HA-SPOP-△NLS	Flag-KCNN3 -△SBC	Flag-KCNN3-△SBC +HA-SPOP	Flag-KCNN3 -△SBC +HA-SPOP-△NLS
101.489	110.067	100.904	97.579	94.217	96.767
83.384	102.476	99.605	94.824	97.913	84.17
89.551	107.372	104.188	91.631	76.287	99.513
96.973	112.066	126.797	94.183	78.185	97.006
96.645	91.829	109.503	91.674	77.926	87.307
95.581	95.506	127.469	88.655	69.469	90.016
98.962	107.469	105.479	87.785	115.987	81.179
94.614	111.083	109.517	102.051	94.678	86.987
97.861	108.523	107.862	82.815	82.032	99.707
118.458	109.408	103.486	85.944	108.591	83.676
105.898	113.337	96.138	96.835	102.368	88.545
103.379	107.706	96.852	88.566	97.495	102.862
109.148	106.548	110.083	90.58	126.658	78.163
99.639	109.429	139.742	92.427	110.897	75.892
110.627	97.587	130.303	88.682	113.928	90.665
101.136	121.609	126.086	93.585	124.915	95.386
100.072	114.515	160.811	86.83	83.692	96.609
102.26	128.069	108.213	85.326	95.76	78.457
98.415	112.563	125.971	86.597	100.526	96.059
99.96	104.298	91.122	95.223	100.219	91.464

Figure 4E

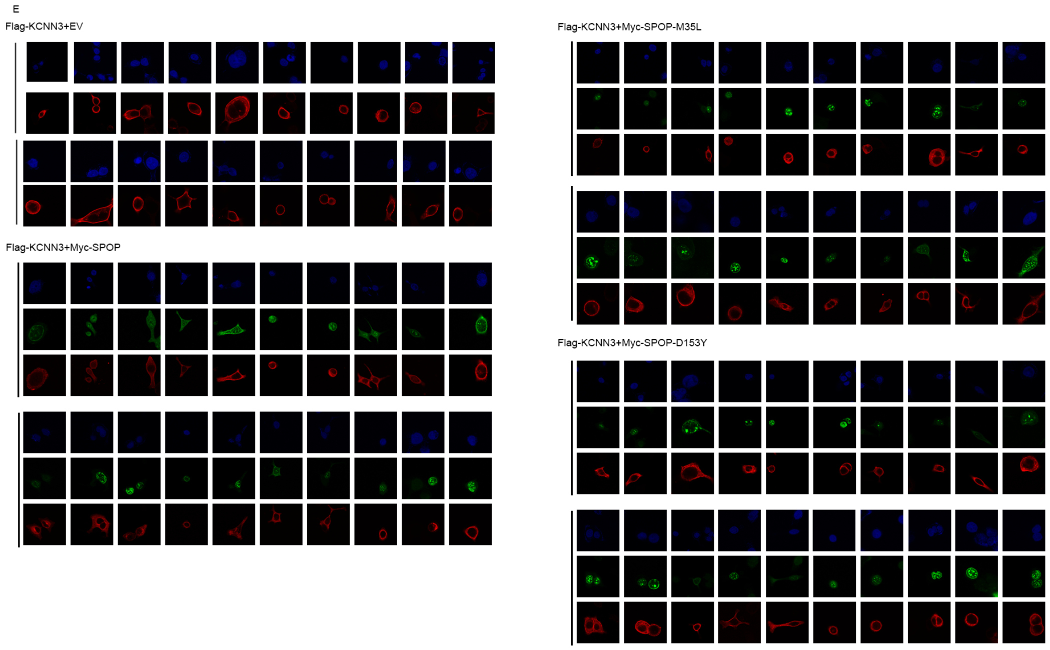
E
Flag-KCNN3+EV
Flag-KCNN3+Myc-SPOP
Flag-KCNN3+Myc-SPOP-M35L
Flag-KCNN3+Myc-SPOP-D153Y

Figure 4F

Total KCNN3			
Flag-KCNN3	Flag-KCNN3 +Myc-SPOP	Flag-KCNN3 +Myc-SPOP-M35L	Flag-KCNN3 +Myc-SPOP-D153Y
132.134	155.78	141.49	171.24
147.343	155.78	155.327	146.476
130.299	151.509	151.095	136.259
126.128	139.61	167.731	143.037
119.68	164.788	136.069	141.965
137.757	142.091	125.428	121.538
137.295	132.474	135.702	118.155
151.326	145.203	154.341	162.175
166.34	136.374	154.152	163.088
141.772	162.333	154.308	154.101
138.309	156.668	140.275	147.018
152.096	158.171	160.905	167.144
152.249	115.008	158.423	148.763
135.528	112.898	163.324	147.905
109.902	146.712	162.918	131.714
154.903	135.13	155.369	137.029
137.924	159.813	177.472	160.167
151.683	173.302	139.147	140.47
157.896	156.78	124.302	146.013
158.464	143.78	122.385	150.626

Membrane KCNN3			
Flag-KCNN3	Flag-KCNN3 +Myc-SPOP	Flag-KCNN3 +Myc-SPOP-M35L	Flag-KCNN3 +Myc-SPOP-D153Y
48.874	26.531	36.505	50.306
69.156	28.177	50.855	63.142
51.75	29.591	40.467	54.808
50.177	33.418	56.005	49.403
54.789	19.699	42.399	54.536
61.504	19.632	51.54	41.527
64.87	37.215	61.395	42.455
62.91	24.938	54.244	69.782
69.873	29.376	42.687	63.811
62.778	37.656	63.008	52.511
73.216	28.241	57.733	50.5
65.551	31.448	49.102	54.129
78.276	37.007	52.865	64.597
70.899	26.459	48.602	53.695
53.006	21.366	72.074	55.622
76.752	50.237	44.77	55.706
64.407	49.599	76.045	62.702
69.937	41.477	56.762	51.339
53.32	35.666	57.175	61.796
56.996	56.827	39.894	64.14

Figure 4F

Cytosol KCNN3

Flag-KCNN3	Flag-KCNN3 +Myc-SPOP	Flag-KCNN3 +Myc-SPOP-M35L	Flag-KCNN3 +Myc-SPOP-D153Y
83.26	129.249	104.985	120.934
78.187	127.603	104.472	83.334
78.549	121.918	110.628	81.451
75.951	106.192	111.726	93.634
64.891	145.089	93.67	87.429
76.253	122.459	73.888	80.011
72.425	95.259	74.307	75.7
88.416	120.265	100.097	92.393
96.467	106.998	111.465	99.277
78.994	124.677	91.3	101.59
65.093	128.427	82.542	96.518
86.545	126.723	111.803	113.015
73.973	78.001	105.558	84.166
64.629	86.439	114.722	94.21
56.896	125.346	90.844	76.092
78.151	84.893	110.599	81.323
73.517	110.214	101.427	97.465
81.746	131.825	82.385	89.131
104.576	121.114	67.127	84.217
101.468	86.953	82.491	86.486

**Figure 4H**	Total		
Flag-KCNN3	1	1	1
Flag-KCNN3+SPOP-KD	0.946427096	1.230583279	0.64057052
Flag-KCNN3+HA-SPOP △	1.084808795	1.347858694	0.759733081
Flag-KCNN3+HA-SPOP- NLS	1.00581166	1.067287725	0.922009723
	Cell membrane		
Flag-KCNN3	1	1	1
Flag-KCNN3+SPOP-KD	1.650275568	1.510717669	1.568588278
Flag-KCNN3+HA-SPOP △	0.545935978	0.197906343	0.334642493
Flag-KCNN3+HA-SPOP- NLS	0.561211443	0.045256683	0.500782494
	Cytoplasm		
Flag-KCNN3	1	1	1
Flag-KCNN3+SPOP-KD	0.105749979	0.089335575	0.04239877
Flag-KCNN3+HA-SPOP △	1.734757747	1.709119945	1.266563425
Flag-KCNN3+HA-SPOP- NLS	1.804581471	1.216727459	2.056564155

Figure 4J

current density at +30 mV

KCNN3	KCNN3+SPOP	KCNN3-SBC	KCNN3-SBC +SPOP	KCNN3 +SPOP-D153Y
53.10	46.50	66.05	50.99	72.47
63.00	76.17	70.84	63.88	85.95
66.26	36.77	104.74	55.44	68.16
64.50	68.58	59.58	63.36	67.10
63.09	49.80	108.43	59.13	85.25
69.86	67.61	64.26	85.63	66.28
58.21	41.59	39.47	62.93	65.42
113.10	34.44	107.08	76.26	67.76
82.28	41.61	65.82	80.39	90.95
70.88	55.31	82.77	78.42	33.70
65.71	64.58	60.16	80.49	63.23
73.48		50.19	93.95	
77.83			86.29	
			65.08	
			73.95	
			68.62	

Figure 5C

si-KCNN3(down)	si-SPOP（up)	promote HCC
MADD	SPEF1	RP11-40C6.2
RGPD6	LMNTD2	GPC3
SEPTIN5	LINC01409	AKR1B10
TRIM46	LINC00115	UBD
LRP5	PERM1	MDK
SCAMP5	LOC112267874	MTND4P12
ALPK2	RNF223	PLVAP
CD99	SCNN1D	OR2I1P
LINC00205	HNRNPCL2	SPP1
ARID3A	FHAD1	STMN1
EPAS1	FAM131C	CDC20
SH3BP5L	LINC01772	LYZ
BCAM	MFAP2	FTH1P7
SPIRE1	LOC105376806	SPINK1
HBP1	MIR6084	TOP2A
MBTPS2	KIF17	THY1
SEMA3B	EPHA8	RP5-940J5.9
RAB11FIP5	NCMAP	CD24
EBP	CRYBG2	SFN
KIF21B	THEMIS2	PDZK1IP1
CACNA1H	XKR8	LINC00152
PFKFB4	SNORA16A	TSPAN8
PRR36	COL16A1	RRM2
MARCHF8	SPOCD1	HSPB1P1
PKD1L1	DCDC2B	MIR4435-2HG
IQCN	FAM167B	ALG1L
PGPEP1	LCK	LCN2
RAD21	FNDC5	CXCL10
CNOT8	HEYL	CAPG
FBXL21P	SLC2A1-DT	TROAP
---	CFAP57	UBE2T
ABCB4	LINC00853	CD34
RNF2P1	LDLRAD1	ZWINT
FUT2	TMEM61	VWF
TSNARE1	FOXD3	FTH1P20
LINC01608	PIFO	MUC13
SORBS1	VTCN1	EEF1A2
WDR31	TBX15	NQO1
BEX2	ITGA10	RP11-452N17.1
PLA2G12B	GJA5	CENPF
TBC1D8B	CCDST	PRC1
CREB5	TRIM46	CDK1
---	LOC101929703	TK1
MEF2D	RAB25	GBA

GOLPH3	MIR9-1HG	RP11-334E6.12
SPAG4	PEAR1	RP5-890E16.4
TSPYL1	MPZ	IFI27
ZRANB1	DDR2	HLA-H
ZNF81	FAM78B	HULC
MAGEB3	ZNF648	CENPM
ABCB10	PCAT6	BIRC5
MMAB	BTG2	EPS8L3
KDM6A	FMOD	E2F1
VEGFA	KISS1	RBP7
SENP7	KLHDC8A	COL4A1
SLC2A4	MIR29B2CHG	BLVRA
SEMA6B	TGFB2-AS1	ROBO1
NUCB1	LYPLAL1-DT	ST8SIA6-AS1
IRX3	HLX-AS1	AC104534.3
ACSF2	STUM	LGALS4
---	SLC35F3	PPIAP22
RC3H2	LOC105373211	APOC2
DENND5A	LOC100130331	HNRNPCP2
PFKFB3	KIF26B	HMGA1
MATCAP2	LOC101929643	FTH1P8
SLC19A3	TRIB2	RP11-1143G9.4
NUP210	LRATD1	MMP11
SNTB1	OSR1	SPC24
MID1	MATN3	NUDT1
PTPRG	FAM228B	RNASEH2A
PALM	LOC105374333	ACSM1
GADD45B	SLC30A3	CTB-63M22.1
ALDH3A2	SIX3	CCNB2
SHANK2	SIX2	FABP5
SYTL1	LINC01816	HKDC1
SCN9A	ATP6V1B1-AS1	TMEM150B
SDC2	DYSF	ERICH5
GAPDH	TLX2	MCM5
PLSCR4	LRRTM4	MCM2
TNXB	CD8A	GMNN
TMEM30A	TEKT4	TM4SF4
ABCA5	FER1L5	KIFC1
BPTF	CRACDL	AC005255.3
EML2	LOC105373511	RP11-667K14.4
RTN2	LINC01159	S100A10
TMC4	CHCHD5	CKS1BP3
ACY1	FOXD4L1	CENPW
SYT17	LOC105373591	KIAA0101
GEMIN8	RAB6C-AS1	HLA-A
MASP1	TUBA3E	TYMS

CIB2	LOC105373716	EIF5AP4
WASF2	WIPF1	MYBL2
SSBP3	LOC105373785	UBE2S
SNX33	DNAH7	CAP2
KCNB1	C2CD6	AGAINST
GDF1	KANSL1L-AS1	UBE2SP2
OS9	CFAP65	RGCC
VWA8	KCNE4	CPVL
KLHL5	NMUR1	LAPTM4B
FAM149A	KLHL30	TMSB10
MOB3A	ERFE	LAMC1
FER1L4	ITPR1	H3F3AP4
PHLPP2	BALR6	AURKB
WFDC2	SATB1	THBS4
KNL1	NEK10	CD74
RTF1	CCDC13	AC239868.2
HECTD4	ACKR2	AC239868.3
ATRX	CLEC3B	BOLA2B
RPL3	GNAT1	KPNA2
HUWE1	SEMA3B-AS1	ANXA2
BDKRB2	ZMYND10	RP11-10G12.1
---	TNNC1	LGALS3
SAGSIN1	STX19	DBNDD1
TLE2	GCSAM	TPX2
CRELD1	LOC105369194	CYP7A1
IL17RC	EFCAB12	PKMYT1
ITIH4	FOXL2NB	RECQL4
VPS13B	ZBBX	CDCA3
MAP2K6	SOX2	HLA-DRA
CLMN	BCL6	RPS28P7
SGCE	MUC4	COL4A2
AGAP1	LOC107986031	AKR1C3
NEAT1	LOC105374339	ESM1
TENT4B	IDUA	HLA-DQB1
CTDSP2	CCDC96	CDCA5
PDXDC2P-NPIPE	ODAM	HSPB1
SRC	UNC5C-AS1	RGS5
AP2B1	GLRA3	NEK2
PHGDH	LOC105377581	NELFE
ALPK3	FLJ38576	SULT1C2
ULK1	LOC102724943	LMNA
VAT1L	GCNT4	CRNDE
CD81	PCSK1	NDUFA4L2
JMJD1C	ERAP2	PAFAH1B3
YPEL2	REEP2	TMEM98
GAS2L3	MZB1	COA6

ADGRB3-DT	SLC4A9	CDT1
ZFP14	PCDHB1	CDKN2C
DGCR2	SYNPO	TCF19
LRP10	CCDC69	PEG10
ANKRD13A	HAND1	TREM2
APBA1	LOC107986467	HRCT1
TCF7L1	LOC107986488	HULC
PRUNE2	DSP-AS1	RPL7P1
CNTN1	LOC105374924	PABPC1
CTTNBP2NL	LOC105374923	DTNA
GNB4	LOC105374937	CCT3
ADAMTS10	H2AC6	MCM6
ANKZF1	H3C6	TMEM106C
POU2F1	ZKSCAN8P1	ASPM
PTGER3	GABBR1	FOXM1
PHKA2	TRIM31	ENPP2
ARAP1	TRIM15	SQLE
SEC24D	MICA-AS1	SNRPE
LINC02338	LY6G5C	GOLM1
CD2AP	PSMB8	PEA15
LGSN	KIF6	ELOVL2
SERPINA5	PRPH2	FEN1
HOOK2	TTBK1	FABP5P7
FLVCR1	DLK2	NUSAP1
PCDHB16	ADGRF5	SMYD3
SH3PXD2B	ADGRF1	SMIM4
SLC25A25-AS1	C6orf141	SNRPB
RBMS2	LOC101927293	COL15A1
BARX2	LGSN	PVT1
VSIG10	IMPG1	SLC51B
NPNT	LOC112267857	SPARCL1
AGO4	TPI1P3	SNRPEP2
VASH1	RSPH4A	TP53I3
GSDME	LOC101927919	CRIP1
RIN2	RAET1G	SPATC1L
ITPRIP	ULBP1	ETV4
NDUFA4L2	PRKN	PDGFA
NEK9	MEAT6	NEU1
KIF3B	PDE10A	TMEM45B
LRRC37A11P	LINC00473	RNF185-AS1
RCOR1	AFDN-DT	CKS2
PLD5	LOC112267971	CTSA
PTPRK	PRKAR1B	KIF20A
SERINC5	ADAP1	MCM3
---	LINC02983	GLMP
PLCB1	COL28A1	PCNA

ABCC6P1	LOC105375172	NSMCE2
NOVA2	CDCA7L	ASF1B
TLE5	LOC102724143	MKI67
KIF1B	HOTTIP	NME1
TAOK1	EVX1-AS	KRTCAP2
TLCD4	EVX1	NDC80
SPARC	INMT	HLA-DPB1
NIPSNAP1	GHRHR	SCAMP3
TGFA	ADCYAP1R1	RPL8
TCP11L2	SFRP4	RACGAP1
---	TARP	MCM4
SERINC2	TRGC2	TAGLN2
NECTIN2	TRGC1	CYSTM1
APOE	TRGJ1	CLIC1
GAREM2	TRG-AS1	SPARC
BSDC1	SUGCT	RP11-295G20.2
RAB6A	MIR6838	HN1
LCN15	AEBP1	AC005943.6
LMO7-AS1	LOC101060341	RAMP1
RAB33B	LOC105375322	CDH13
RCBTB1	LOC105375328	NCAPG
NBEA	SPDYE7P	SEZ6L2
MAN2A1	CCDC146	TUBA1B
FRG1BP	GRM3-AS1	ARSEP1
ERP27	LOC101409256	PKN1
AMN1	BHLHA15	DPP4
IGF1R	MIR93	UGT1A6
MTSS1	MIR4658	MAD2L1
KIAA0232	NYAP1	KRT23
MDK	ZASP	VPS72
AMBRA1	ACTL6B	MCAM
QPRT	MUC3A	BAX
RERE	LRGUK	BOLA2
LHPP	TAS2R5	C12orf75
TMEM30B	PIP	CPQ
DPYSL2	LOC107986856	NTPCR
TMEM45A	ZNF467	VIL1
C8B	ASIC3	LAGE3
EXT1	LINC01287	RP11-756A22.7
HERPUD1	PTPRN2-AS1	SF3B4
LGALS14	FAM167A	THAI
MAT1A	FGF17	RP11-345J4.6
ZNF417	HR	SHFM1
ITGAL	PHYHIP	RP3-461F17.3
CLDN3	LOC105379326	GRN
SLC6A19	STAR	RRS1

GATM	LOC105375825	SSR2
TNIP1	LINC01301	RP11-316M1.12
SUMF2	ADHFE1	ANXA2P2
PDZK1	CA13	CDC45
MAL2	MIR3610	COX4I2
SLC38A3	PCAT1	NUF2
OLMALINC	KCNK9	APOA1BP
SND1	MAPK15	CNIH4
EIF4B	MIR4664	PYCARD
PDE9A	SMPD5	STXBP6
FBXL2	WDR97	AP003391.1
MCL1	FOXH1	ENAH
ADAMTSL4	RLN1	HDAC11
EHD2	CD274	MND1
CLIC4	SPAG8	PSMD4
LDLRAD4	HRCT1	CKLF
LRATD2	FAM95B1	COMMD4
ICA1L	FAM27E4	SORT1
PLCD1	LOC107987007	CCDC34
AASS	LINC01410	GPNMB
WDR72	FAM27E3	NCAPH
PPP1R9A	LOC101927191	PIGC
FBXL4	PCA3	HMGN2P5
TNIK	FRMD3-AS1	EPHX1
ZMYND8	LOC107987098	RPL37P6
RHOT1	ABCA1	HLA-F
CHRD	LPAR1	MAN2B1
SLC43A3	LOC107987116	ATOX1
ACSL3	LHX6	CHEAP
CHD9	PIP5KL1	CENPU
---	CERCAM	ITGA6
SMAP2	LINC02913	TNFRSF4
ARHGEF40	FAM78A	RPL30
KDM4B	AK8	UQCC2
C4B	PIERCE1	SQSTM1
OSBP	CARD9	HLA-DQA1
DTX4	NALT1	TMCO1
---	PTGDS	IQGAP3
SLC39A5	CYSRT1	RMI2
PGM1	ENTPD8	H2AFZ
UTRN	LOC105376380	RP11-345J4.5
PTPRJ	GATA3	SNRPC
PDK3	STAM-DT	SOX9
C2orf16	NEBL-AS1	RPL36A
TMEM59	GPR158-AS1	UGT2B11
NF1	FXYD4	FAM83D

LINC01659	CABCOCO1	OLFML2B
GPR137C	SPOCK2	SLC44A3
FBXW11	GLUD1P3	U91328.1
FASN	SFTPA2	RP11-620J15.3
EIF4E3	MBL1P	BSG
BCOR	DYDC2	CCL15
BBS10	SNCG	HMMR
FAHD2A	IFIT2	RGS10
RBCK1	HTR7	CPE
MAPK9	KCNIP2	SUMO2
RNF150	MIR9851	GNAZ
P4HA2	LOC105378550	CLN3
MRNIP	CTAGE7P	MILK
ATF7IP	LINC02870	CXCL9
PLEKHG5	ADAM8	ITIH2
ZBED1	LRRC56	ILF2
MVD	LMNTD2-AS1	LGALS3BP
---	BRSK2	HIST2H2AA4
MORC4	LOC105376514	TLCD1
CYP4F23P	DUSP8	TOMM20
LIMD2	KRTAP5-AS1	SNX8
SLC29A2	KRTAP5-1	MRPL24
CYP4V2	OR2AG2	PTP4A3
F2R	OR7E14P	RNASE1
---	DCDC1	XXbac-BPG116M5.17
ACAP3	KIAA1549L	TNFRSF12A
PASK	FJX1	VAT1
CTNND2	RTN4RL2	RPL7P9
CREBRF	UBE2L6	SCNM1
CAPN12	GLYATL2	BOP1
GRASLND	CD6	HLA-DMA
ATRNL1	SNORD28	PECAM1
PAXIP1-DT	CCDC88B	UFC1
MTMR12	SPDYC	SCAMP5
COL4A3	EFEMP2	GBP2
NADK2	GPR152	NR2C2AP
MAPK13	CABP4	APOA2
HSF4	ACY3	NT5DC2
CTC1	P2RY2	ITPKA
RAB8B	MAP6	GPAA1
DDOST	UVRAG-DT	RP11-641D5.1
JUP	MYO7A	HMGB2
MBL2	LOC107984429	RPL39P3
CRYL1	LOC107984428	CDCA8
MYRF	FZD4-DT	SNCG
FNDC3B	SNORA8	RP11-61N20.3

EHBP1-AS1	BIRC3	HSP90AB1
FBXO25	TMEM123-DT	EEF1A1P6
---	CASP4	BCAP31
---	GRIA4	LINC01296
GALNS	APOA5	ZFAS1
HACE1	RPL23AP64	GM2A
MMP11	TRIM29	ARPC5
CECR2	ROBO3	H3F3A
THBS3	OPCML	ROMO1
PGD	LOC107987374	PODXL
FADS1	LPAR5	LEF1
DUSP8	PIANP	POLR2K
ABCA7	C1RL	NPM1
GCA	LOC102724146	RP11-215A21.2
DAB2	KCNJ8	CKS1B
ESPN	IRAG2	TXN
MBOAT7	DNAI7	KIAA1462
PLSCR3	LINC00941	HIST1H2BK
RAB3GAP2	LOC105378248	PFDN6
KLF9	SLC38A4-AS1	RPL41
CHMP1B	PCED1B	TM4SF1
PLEKHG2	LOC101927241	LTB
KLC4	CCDC65	HLA-DPA1
---	KCNH3	DTL
C2orf72	NCKAP5L	RP11-284F21.10
RAPGEF1	GPD1	IFI27L1
TULP4	ITGB7	TMED3
---	HOXC13-AS	PBK
ARL2-SNX15	LOC105369775	G6PD
CTNND1	HOXC11	MAGED2
ZFTA	ZNF385A	ABHD12
ENPP2	PPP1R1A	PSMB9
MYO9B	USP30-AS1	RELB
KCNK6	FOXN4	MYL6B
SPRING1	PLBD2	HLA-B
LINC01719	LINC02463	DARS2
FBN1	SIRT4	RAB11FIP4
SLC6A16	NRAV	PLA2G4C
MXI1	CABP1	ANKRD29
ERRFI1	CFAP251	DBP
ANXA4	LRRC43	PIGT
SMIM10L2B	LOC107984450	ANLN
GHR	MMP17	ATAD2
CEBPA	C1QTNF9B	MRPS21
WASL	PDX1	EIF3E
---	LOC105370203	NOTCH3

SEC24A	TPTE2P3	CKAP4
H1-0	LOC105370274	DYNLRB1
C1orf115	GPC5-AS1	CENPA
CCDC68	MCF2L-AS1	RHNO1
ONECUT2	LOC107984705	SNRPD1
PFN2-AS1	MIR6717	ATP6V1F
PHF2	SALL2	NPM3
ITGA5	EFS	ZNF28
---	ADCY4	PHLDA2
---	LINC00648	RPLP0P6
TRIOBP	LOC105370532	UCK2
PIK3IP1	JDP2-AS1	RFC4
CXXC5	ISM2	KIF2C
MPZL3	FBLN5	TRIM31
UBXN6	HHIPL1	RP1-241P17.4
SEMA6A	BEGAIN	APOBEC3B
SHFL	MOK	RHOC
CHD2	IGHVIII-38-1	PRPF6
ORAI3	LOC102723407	MTX1
PCAT7	FMN1	SERF1B
EXPH5	DISP2	SRXN1
IGSF3	STARD9	COX6B1
KLHL36	TGM5	SNRPD2
KIF12	DUOX1	TACC3
ARFGEF3	SHF	LYPD1
KLF12	LINC00926	RP11-443P15.2
ENTPD2	APH1B	CCNA2
TXNIP	ITGA11	PRIM1
STRA6	LOXL1	RNF157
SECISBP2L	CYP1A2	LMNB1
EIF4BP6	ISL2	MRPS23
ZCCHC14	ADAMTS7	TMEM9
MBTPS1	WDR93	PBXIP1
---	IDH2-DT	PKM
CDKN2C	LINC01579	GDF15
SIDT2	RGS11	PLK1
HPS3	GNG13	SAC3D1
GOLGA8A	SNORA64	RBM34
TMED7	DNASE1L2	MEA1
ANO6	ATP6V0C	RAMP2
BCL2L2	MMP25-AS1	RPL23AP42
RAB2B	CASP16P	RPN2
LAMP2	VASN	GTF2IP4
PLIN2	UBALD1	CDC25C
DHCR7	SMIM22	NENF
NADSYN1	LOC105371094	IFI30

H19	PLA2G10	CACYBP
MAP3K15	SMG1-DT	CTHRC1
PAM	ANKS4B	FAT1
DNAJC6	VWA3A	HIGD1B
HSD17B4	NPIPB5	TUBG1
BACH1	LCMT1-AS1	HLA-DRB6
EML4	GDPD3	SLC41A3
ZDHHC20	ZNF843	RPL38
DYNC1LI2	ABCC11	KIF4A
SYT11	CHD9NB	SPC25
FN1	MT1L	PAQR4
IDH1	PLLP	KIAA1522
LYPD5	DRC7	UQCRB
DGAT1	LOC105371308	LSM2
TPH2	CES4A	RP11-464D20.2
---	FBXL8	ATP5G1
ZSWIM6	TPPP3	H2AFX
VAV2	C16orf86	FDPS
EMB	TSNAXIP1	EHMT2
TMEM37	HYDIN	MMP14
SLC2A14	LOC105371361	SNRPGP2
PTPRM	SNAI3	ATP6AP1
ZBTB4	LOC101927793	CADM1
PPP2R3A	CDH15	LOXL4
SALL2	LOC101930112	TPM3
PUM1	LINC02166	SELM
RBM12	GAS8-AS1	HLA-DMB
GALNT3	FAM157C	CUTA
APOH	LOC101927999	Metazoa_SRP
ESYT1	RPH3AL	S100A13
INSIG1	LOC105371430	VAMP5
ABCA1	C17orf97	TOMM6
PMEL	SCARF1	SLC50A1
IL1R1	RILP	ZNF706
TBC1D9	SLC16A13	FAM83H
DKK3	CD68	TESC
PCDHB9	MYO15A	RPL37
RBP4	LOC105371712	EXOSC4
KMT2C	EFCAB5	HIST1H1C
TTC3	LOC646030	MARCKS
TLE6	TNS4	BMS1P8
XDH	KRTAP3-1	RP11-713M15.2
FAM110A	ZNF385C	BOLA3
FUT1	C17orf113	PXMP4
ADM2	RAMP2	PPDPF
LRRC8A	TMEM106A	NME2

STIMATE-MUSTI	CCDC103	EPRS
GOPC	GFAP	FLYWCH2
GC	FMNL1-AS1	PSPH
EIF2AK3	TBKBP1	RPL39L
XIST	LRRC46	NRM
ZNF688	HOXB8	RPS21
PNPLA3	NXPH3	FANCI
TSPAN12	TMEM92	ALYREF
ERGIC1	MYCBPAP	DRAP1
ZNF571	ABCC3	PLP2
SH3PXD2A	TMEM100	PDZD11
AGPAT2	LIMD2	FLVCR1
LINC03048	LINC02097	YWHAZ
HPN	MYO15B	TUBA1C
LYPD6	ITGB4	ATP1A1
CDKL5	LGALS3BP	MAZ
MARF1	LOC100129503	NHP2
KMT2E	TSPAN10	NDUFB9
DNHD1	LOC102724615	PRAP1
NCOA6	MAPK4	IFI6
HDAC3	STARD6	C15orf48
PDPR	LINC01541	AATF
HOXD9	LOC105372235	CLDN15
CHD7	LOC102723811	RPL35P5
ZNF337	PCSK4	GMPS
CRYZL2P-SEC1(	ATP8B3	HMGN1P37
ERC1	LOC100288123	ARL2
ZNF654	SHD	LINC00511
TP53INP2	SEMA6B	HJURP
LMTK2	MIR7-3HG	ATP1B1
LPIN3	C3	PRCC
---	PRR36	MCM7
GAS6-AS1	CTXN1	H2AFJ
FRY	LOC105372273	TMCO3
FNBP1L	RTBDN	UQCRH
CLDN7	LOC105372281	UXS1
JDP2	MIR23AHG	RFX5
ARID5B	NANOS3	EIF3H
FGL1	IL27RA	PSMB4
BNIP3L	PALM3	AP1S1
PDE3B	CYP4F8	PPIA
BNIP3P1	BST2	CHAF1A
SARDH	BISPR	MSH2
IPMK	KCNN1	RRM1
SRPX2	ZNF90	LAMA3
RPN2	CHST8	RPLP2

PLEKHG6	FXYD5	CTSS
CRABP1	ETV2	EXO1
PDE2A	DPF1	FBL
HNF4A	PPP1R14A	TRMT112
G6PD	DYRK1B	IRAK1
SMTN	TGFB1	BCAM
KCNH8	GRIK5	DAPK2
SEMA5B	CEACAM1	NFKBIE
GFPT1	BCL3	ARPC1B
P4HB	BCAM	COL1A1
DHCR24	APOC1P1	FLAD1
S1PR1	ZNF296	NUP37
GK	NKPD1	SDC2
KIDINS220	FOXA3	TOMM7
SEC31B	PPP5D1P	TRNP1
COL6A2	DACT3	CENPH
RND2	SULT2B1	DTYMK
MYH14	FAM83E	EMC3
WIPI1	HSD17B14	RPS20
PHF13	LOC101059948	SAE1
LYPD6B	CGB3	IL32
GSTM2	CGB1	PLOD3
CEP170	CGB5	TECRP1
TMEM141	CGB8	RAN
TMEM121B	CGB7	ATRN
GOLGA8J	NTF4	CDC6
TMEM63C	KCNA7	RPL7
ATP8B3	GFY	PSMG3
SLC29A4	KLK10	MANEAL
CNOT1	KLK14	XRCC1
---	VSIG10L	RPL23AP65
TUBA1A	LINC01530	ANAPC11
FOXD3-AS1	TTYH1	GABRD
SEPTIN10	DEFB132	C8orf59
TPM4P2	LOC643406	VARS
SCRN1	SNORD17	AL158801.1
YIPF2	DZANK1	DCAF13
HAVCR1	CFAP61	MPC2
KDM5A	DUSP15	UHRF1
FSCN1	TTLL9	LRRC1
GPR161	BPIFB1	TRAPPC2B
SCARA3	ZNF341	CD63
COL12A1	LOC105372602	SUB1
WIPF1	MYL9	TCEB1
ZMYM3	TGM2	COMMD5
ZSWIM4	MMP9	TXNRD1

ONECUT1	SLC12A5-AS1	SHISA4
ROBO1	SLC12A5	RP11-452F19.3
ZNF609	LOC105372632	HEXB
OTOG	CD40	PYGB
AGA-DT	BCAS1	DYNLL1
GDPD1	LOC102723814	RPS4X
ORM2	FNDC11	MRPL9
MAGI1	SAMD10	TYMSOS
BAALC-AS1	CH507-42P11.6	SMIM11
VKORC1	TCP10L	ECT2
SPAG9	RUNX1	RPLP1
OCRL	DSCAM	TM7SF2
---	DSCAM-AS1	FAM189B
HK2	MX2	VPS28
CALD1	RSPH1	P4HA2
AKR1D1	LINC00319	H2BFS
DNMT3L-AS1	POM121L15P	MESP1
NIPAL3	LOC112268289	SLC52A2
BICC1	TBX1	DUSP9
C4A	LOC107985532	IFI27L2
AKAP13	ZNF280A	BRMS1
C1QTNF3	CES5AP1	HNRNPA1
ALDH1L2	CCDC188BP	FAM222A
GCKR	FAM230I	ANXA13
KIF3C	PCAT14	IL3RA
ZFP64	SLC5A1	CKB
---	HMOX1	CXorf36
RNASE4	RASD2	OIP5
ERO1A	RAC2	NDUFS6
LIMCH1	LOC105373027	CCDC107
MSMO1	SNORD139	GLA
IFITM3	CYP2D7	AP1M2
---	A4GALT	TATDN1
DPYD	IL17REL	CDKN2AIPNL
JRKL	LOC105373096	PDGFB
ZCCHC2	LINC00685	STC1
---	LOC105373102	BUB1
HDHD3	CD99P1	VIM
NID1	PCYT1B	LAMA4
PRRC1	ARX	RPS2P55
CCSER2	LOC105373154	RPS18
GTF2IP1	PRAF2	HSPA4
CILK1	EZHIP	ASPH
NAV2	RIBC1	LMCD1
BMT2	CXorf49	FOXRED2
TSPAN18	CXorf49B	OLA1

FRAT1	RTL5	PRIM2
PRSS22	FAM226B	COX6C
SNX9	FAM226A	TMEM50A
TLR6	CCDC160	RP11-161H23.5
PPP1R1A	MAGEA11	ATP5J2
ACSL4	WASIR1	CTSC
RSF1	GYG2P1	ZNRD1
LINC02035	VCY1B	ITGB1BP1
NSD3	LOC107987399	C6orf48
SLC24A1	TRNL1	ENSA
RNF208	ND1	NME1-NME2
MTMR4	TRNQ	ADAM15
ACOT12	ND2	HSPG2
AFF1	TRNW	STK39
SLF2	COX2	TRAC
OGFRL1	TRNK	CD163L1
MSRB2	ATP8	PPT1
ICAM3	ATP6	ST14
LOX	COX3	FAM103A2P
PPP6R2	ND3	CDK5
WDTC1	ND4L	RFWD2
ITIH5	ND4	OLFM2
---	ND5	SPCS2P4
TBX2-AS1	ND6	ORMDL2
LAMP1	TRNE	LSM8
PAK1	CYTB	RP11-51O6.1
PRKAR2B	TRNP	UBE2Q1
NIPAL2		NAA20
HOXC6		SEMA3F
FGF11		COX7C
APLN		RFXANK
RAP2C		SOWAHA
LRRC37BP1		GSTA4
POU2AF1		POLD1
EIF2AK1		TTC39A
CA3		UCP2
RBL2		LYPLAL1
ACSS2		FTH1
GGT7		NMB
LMTK3		H2AFV
CYP4F3		RPL15P3
ABCG4		GINS2
FGFR1		PYCR1
PLXNB2		NDUFA1
TBC1D9B		PFDN2
HMGCR		SLC39A1

SSH2	CALR
DOCK1	AC016739.2
RBM43	ATP1B3
PRKCH	SLC38A6
PCNX1	NOL7
PKLR	PHF19
LRRC31	NES
HID1	ZNF687
ITM2B	AIM1L
ZCCHC24	BYSL
ZBTB10	TMEM101
LNPK	RBM8A
SORT1	CPD
SIMC1	EIF3K
WDR6	TMEM147
TGFBI	ATP5L
SMPDL3A	ZIC2
CLSTN3	EXOSC5
CDH6	SCD
ARRDC3	RPP21
SLC30A5	APOLD1
ZMIZ1-AS1	CBR1
SEPTIN8	PFDN4
NEDD9	SPSB2
SLCO4C1	ATP5J2-PTCD1
BTBD8	LGALS9
ACP5	ACLY
ACAD10	AP3B1
CACUL1	DAP3
BEND7	SHARPIN
TMPRSS6	EFNA4
INHBE	FAM50A
ACVR2B	RPL32
MBNL2	CD2BP2
SAP30L	HACD3
SPG11	S100A6
PRELID2	ECSCR
BACE1	TPGS2
ZNF608	GPANK1
SATB1	AC005943.2
ATP9A	SOX4
AGPAT3	NGFRAP1
FBLN5	AKR1C2
SEMA4B	HOMER3
MFGE8	DLGAP1-AS1
ZNF710	RNASET2

LMO4	LIG1
GABBR1	MPZL1
PRKAA2	CDK4
---	ACBD6
CROCCP3	UFD1L
---	CCT6A
DMTN	EIF3D
ITCH	NPM1P27
LRRC8D	GARS
RBMX	CFL1
NPTXR	FBLN7
APOB	RBM42
MPST	TWF2
LOXL4	HLA-DRB1
GOLGB1	ASNA1
LINC00299	EEF1A1P5
TRIP11	RPL24P4
CAPN7	CTC-246B18.10
TBC1D5	DUXAP8
---	POLA2
TMEM140	CCT5
POU6F1	RALY
FDPS	FAM111B
ABCC6	COL1A2
LANCL1	PIR
LINC03040	HTATIP2
GTF2I	FBXO32
ENO3	APLN
DST	GGH
SMARCC2	ADCK2
IL1RAP	SERPINI1
PLXNB1	TDRKH
F5	SERPIND1
TBCEL	SLC39A3
B4GALNT2	MRPL13
DAB1	TMEM38B
AMOTL1	SLC39A7
LTBP1	MAF1
SLFN13	TRIM52-AS1
ATP1B2	CLDN7
ADGRL1	AC009065.4
ZFYVE16	PARVB
UBE4A	GJA5
SFTPD-AS1	TP53BP2
STX17	C1orf198
FSD1	C8orf33

C19orf73
LINC01607
MKRN1
UACA
MCAM
SCARB1
TRPC4AP
UBE2R2
TNFAIP8L1
SIX4
CMTM3

CEP250
LPCAT1
BMF
PABPC1L
SLC2A10
LINC01419
LINC02580
MTUS1
ADGRB2
EXOC6B
PRDX2
VHL
FDFT1
GALT
C11orf52
TNNT1
TP53BP2
ZXDC
USP34
SLIT2

ZNF764
GJB1
RGPD5
SMIM14
NDRG1
BICD2
KCNH7
SERPINI1
SORBS2
CLASP2
PDXDC1
PLEKHA1
MX1
BTG3
WDR76
KIAA1429
SMARCE1
C16orf59
H1F0
MAPK3
FKBP11
NABP2
FAM3B
NDUFA13
FABP4
PUF60
KIAA0196
GGTA1P
EIF6
MRPL17
FHIT
TUBB
COPA
SRD5A3
SNHG6
DBN1
KNSTRN
LMAN2
NPC2
RP11-73M18.2
B9D1
TIMM9
FANCD2
ZNF544
CENPN
LOXL2
NOMO2
CCHCR1
RPL27A
STT3A
GPATCH4
ITGB3BP
CCDC86
FLVCR1-AS1
CBX1
CCDC167
RNF187
RNF181
PET100

PTGFR
KLC1
MCF2L
F10
ACADSB
SUSD3
FOXO3
IGF2
CROT
NOXA1
MACF1
DCAF11
CMPK2
LPIN1
MYCN
MBOAT2
CBL
TET2
HDAC5
BTBD3
ZNRF1
TOP2B
ZBTB5
PLEKHM1
DDIT4
CUTALP
KIAA2026
MBTD1
FLRT3
XPO7
LRCH2
PJA2
DNAJC18
N4BP2
SLC35E2A
ALAD
B4GAT1
MIR600HG
MVK
GRB10
TP53INP1
SOGA1
SGPL1
PTP4A3
COL4A5
AGRN
ITGB4
SERPINH1
RAD21
CCDC80
RRAGD
LL22NC03-N14H11.1
RPL27
UGGT1
ATP6V1E1
SERPINB1
NCSTN
PSENEN
HIST2H4A
DNASE2
NDUFC2
MRPL55
CYB5R1
THOC3
RPS27
LYRM4
COX7B
RAD51C
LAMTOR4
CHEK1
PMF1
TUBA1A
TARBP1
ISG15
C19orf53
IFI35
IMPDH2
RTN2
PARP1
TBC1D7
RPS14
FOLR2
STIP1
B3GNT5
TMEM14C
PPIH
C17orf58
DSN1
IGBP1
RPL19
H2AFY
SPNS1

BEX4
ARMCX3
ASAP1
OAZ2
IMPDH1P5
KREMEN1
DCHS2
SLC25A23
ITPR3

SPINT1
CFAP44
TRIQK
FZD4
IDUA
IL13RA1
PLXNC1
SLC44A1
PRG4
C1RL
SERPIND1
ZC3HAV1L
SHE
SUFU
PPFIBP2
CLDN6
TRIM32
NEK6
SPON2
SLC6A13
OXR1
CCDC154
SPOCK2
RNF152
CDC42SE1
SERPINF2
MARK4
NPPB
FYCO1
UBE4B
PHLDB2
GDF11
PGK1
PIAS2
PRKACB
CCDC92
RPL35A
TOPORS-AS1
ATP5E
CAPNS1
CSNK2B
RPS10
PPP2R1A
NECAB3
TMEM14B
UBL7
SCARA3
RPS7
MYEOV2
RPL39
PSMD10
GTF2A2
TALDO1
GINS1
RTP4
DCTPP1
TTC9
RASSF3
CSE1L
EHD4
HAX1
MRPS16
HMGN4
VRK1
PPM1G
RP4-706A16.3
BCL2L12
NHP2L1
LAMTOR2
C7orf50
SMYD2
AKR1C1
ATP5G2
C8orf76
KIFAP3
SRC
DNAJB11
ZMAT5
C17orf49
C1orf54
C2orf54
DLGAP5

KDM3A	TGM3
ST6GAL1	EIF2D
USO1	MTHFD1L
SCARB2	SLC35B2
CCNG2	SOX9-AS1
AFDN-DT	BOLA1
APOC2	TFPT
PPP1R13L	CYTH2
C11orf80	HM13
FAM171A1	MAPRE1
RGP1	GSN
MPDZ	LSM4
DDX17	RP11-480I12.5
ITIH2	NDUFA7
CDKN1A	ANO10
SOX4	RPL14
KIF5C	PITPNA-AS1
ORM1	PSMB3
NPDC1	ALG8
FZD7	BUB1B
MST1L	SKA1
RNF144A	PPIB
CHRNB1	GOLPH3L
MLLT6	FAM49B
PPFIA3	FAM195B
---	HLA-DQA2
---	DDX41
PDE6A	TRAPPC2L
EIF2S3	NACA
CORO7-PAM16	RPL6P27
TJP3	BLOC1S1
NKX3-2	PTK2
PRKD2	RABIF
BLTP1	ATP5H
PYGL	GLUL
TRIM9	ZP3
ZRSR2P1	RP11-452I5.2
---	C1orf43
CTSD	CASK
CD276	CD3D
TMEM178B	RAP2A
GPD1L	ANXA11
EBLN3P	ATP6V1C1
MED13L	C1orf106
COL7A1	GNPAT
---	GGCT

RNF213	MMP9
P4HA1	PDCL3
MEX3A	SRP14
PCDHGA10	B3GNT3
ULK2	RAD51
SLC22A31	UBL5
---	HLA-C
SSH3	SPAG4
VPS13A	RUVBL2
RAB3B	PSMC1P1
HERC1	SLC12A5
CRABP2	POLR2G
HMG20B	RNU4-2
FBLN7	HEXA
---	HCST
REEP6	QARS
ATAD2B	MRPL14
LTK	F5
SNX30	CCT4
CYP51A1	DPY30
PTPN3	C6orf47
RAB6B	HAUS4
POFUT1	UBE2Q2
STARD4	TAP2
PRKD1	TOMM40
FZD3	RP11-579D7.2
SLC16A10	ARPC1A
TUBB4A	DDAH2
ZNF862	TAX1BP1
ZER1	PLXND1
LINC01943	AP000349.2
GLYATL1	C20orf27
MYO5C	CASC5
RN7SL113P	DLL4
VPS13C	C19orf48
ADAMTS19	CDKN2B
MUC12	LASP1
---	CHCHD3
---	EEF1D
MEIS3	TGFBI
EGFR	HLA-DRB5
NEURL1B	CAPN2
SEC16A	HNRNPA1P48
KPNA5	AC012146.7
ARHGEF12	CHMP2A
MTURN	CKAP2

KANK1	ALDOA	
NBPF14	EMCN	
MKNK2	COPG2	
B4GALT1	MGP	
PTPRZ1	PHLDA3	
C5orf15	EIF3CL	
PCDHGB5	EMC2	
TTC33	PSME1	
ENO2	SPA17	
ATN1	SPATS2	
FGFR3	NREP	
PLCXD1	RP11-465N4.4	
OSBP2	ACP5	
EPOR	PSMD14	
TMEM191A	RPL36	
DENND1A	MFSD5	
MYORG	AFP	
KLHL28	CEP131	
SCIN	CTB-25B13.12	
ARHGAP44	NSUN5	
EFHB	IGF2BP2	
ACSL6	CMSS1	
MTTP		6-Sep
AMACR	FAU	
CPN1	PRKDC	
EIF3L	RPL28	
---	PHB	
C3	NOMO1	
VPS26C	SNRPB2	
SOWAHB	PYCR2	
CASKIN2	C15orf39	
CLK3	SNF8	
PTPRU	TSTD1	
APBA2	AC007318.5	
SLC7A10	CD248	
TSPAN33	JMJD4	
MYO7A	RPL24	
RDH11	FKBP1A	
DYRK1B	OPTN	
KLF3	PA2G4	
PRR15	ZNF775	
GNG7	CTD-2540B15.11	
REEP2	UBE2M	
SERPINF1	WBSCR27	
TAB2	KDELR3	
GLCE	PIK3R2	

A1CF
PANX3
MBNL3
RBFOX2
TMCO3
MOXD1

INKA2
NRK
UXT-AS1
SLC16A7
BICDL2

PTBP3
NSL1
SLC30A10
ADGRG2
MFAP2
TRHDE
SYNPO
ITGA1
SESN1
RNF217
RHOBTB3
ARHGEF10L
KRT23
TGOLN2
MINDY1
TBC1D8
SC5D
CBLB
ZDBF2
XYLT2
FBLIM1
ENAH
AFDN
ERCC5
ARL10
TCP11L1
P2RX4
PKIA
CTPS2
ATG2B
TUBB3
TM4SF4
IDI1
GNS
RPL13AP5
AKIP1
DPM3
METTL13
NUTF2
RPL14P1
CNPY2
ADM2
ALDH3A1
DYNC1H1
TMEM132A
NEDD4L
RBM3
ICAM2
RPSA
TRIM16
BANF1
NDRG3
BUD31
COPS6
PSMC4
IK
GJA1
FAM20B
RPS11
SUCO
TBCB
MYL12B
TMEM258
WDYHV1
POLR3C
DNPH1
HMGN2
COX20
PRDX5
LY96
TBC1D16
SUMO2P1
ALG3
PLAU
SMO
HIGD2A
TAP1
SLC35F6
GPSM1

NR1D2	CBX8
HLA-B	GTF2IRD1
GNPTAB	TMEM14A
PKHD1	SSR3
TANC2	OSGIN1
SLC23A2	SLAMF8
WNK1	LRPAP1
ANKRD9	EFTUD2
TPD52L1	TAX1BP3
ABTB1	ACTN4
AKNA	CRNKL1
PAK5	MIF4GD
NDST1	ALMS1
TLE1	CLEC14A
CRYZL2P	AIDA
---	CAV1
SLC26A2	RPL26L1
RIMKLB	MAP1S
NXPH4	TM4SF5
ZFHX3	LCMT1
TACR1	RP11-49K24.6
ADAMTS5	TRAF2
ETV1	STYXL1
TCAF1	RPL29
RALGPS1	S100A4
EPHA7	PTMAP5
LINC00261	RPL23
---	PAPSS1
GCHFR	UTP14A
AFF4	NAP1L1
B4GALNT3	SUPT4H1
PCSK6	EIF3M
SGK1	PSMB5
CRIM1	HTATSF1
C2	P3H4
ITGA6	SUSD4
---	PMVK
CNTRL	PTENP1
CCDC69	DAXX
LGMN	SAMD1
PCSK9	NDUFA2
ZBTB7B	HNRNPA3
MAVS	RP11-556E13.1
NR2C2	ARPC3
SHC2	PIK3IP1
ACAD11	TRIM28

TFPI
TCEA3
PKD1P6-NPIPP1
BMPR2
HLA-A
GAMT
SNX19
GNAZ
VPS13D
RORC
BHLHE40-AS1

AHCYL2
SEL1L
SH3D21
ADAMTS20
GLP1R
SAYSD1
MKI67
THRB
SETBP1
ZFAND6
ATP8B2
AKR1A1
PSD3
LRP1
FABP1
ST14
UPK1A-AS1
TUB

TMEM198B
RASA4B
DACT1
RALGDS
PRKX
ENTPD7
BTAF1
GREB1L
CCDC186
ACACA
RCAN3
PHEX
OTUD5
DLX4

SND1
CETN2
STAT1
TBCE
SKA2
TSPAN4
BZW2
CD151
CSTF2
WBSCR22
TNFRSF21
POC1A
SH3BGRL3
RP11-386G11.10
RPS16
SMARCA4
RAC3
ALG1
ELOVL1
RPLP0
PSMA6
HDGFRP2
SLC43A2
UBE2Q2P6
NCAPH2
TCEB2
PDIA3
ZNF585A
BRIX1
CCT7
CLTA
DAD1
ZCRB1
LINC00665
C6orf1
METTL5
SNRPD3
PSEN2
TSPAN15
RPS23
AC016292.3
APOE
ARSE
LINC01604
CANT1
CUEDC1

PDE5A
PEG10
GTPBP2
FGF10
ARRDC1
HOXA13

PTAR1
ATP7B
GPC6
EVI5L
RAB26
CCDC125
COL9A2
SPEF2
FKBP9
SIK2
ARL6IP6
RNASEK-C17orf49
CORO2A
TENM2-AS1
ODF3B
PEX7
EGLN3
MTHFR
BRSK2
POMT1
DARS1-AS1
CCNL2
RIMS3
GRIPAP1
MT-RNR2
APOC3
TRANK1

SAMD11
GNS
APPBP2
CPM
TRIM56
RAPGEF2
LINC00265
LAMTOR4
SLC6A9
LMBRD2
TSHZ2
DNAJC19P9
CD36
CGREF1
DRG1
IRF6
HMGN1
COX8A
TOMM22
UBALD2
ADSL
ANKRD39
DDOST
SPCS1
COX7A2
MYL6
SPTSSA
OLFML2A
C6orf89
CTNNBL1
MAGOHB
PDGFRB
RAC1
NDUFS4
RAD51AP1
SRP9
INHBB
PPP1R11
OST4
MRPS17
TRIP13
RP11-128M1.1
C12orf73
CENPL
ABCC6P2
PSME2
MKKS
PARPBP
KCTD17
AGPAT1
S100P
ALDH1A1
ATRAID
YIF1B
GLB1
FXYD5
S100A11

LINC02770	RPL22L1
TMBIM4	SOX12
---	TBCAP1
ARCN1	CHCHD6
---	COX6A1
CYP2U1	DBNDD2
F7	PDE6D
BMPR1A	RUVBL1
ZMYM4	BPNT1
C14orf132	CCDC64
GUCY1A2	CTSH
SLC16A9	RPL6
C1orf210	NSL1
FLNC	SLC6A8
DNAJB9	SHC1
EPHA3	RPL41P2
PDE4DIP	CCNE1
HOOK1	DHRS7
KDF1	MANBAL
RNF44	RPS19
VWA5B2	SEC61G
ELOCP2	DPCD
AHSG	YIPF3
MAPK6	GAS5
RHOU	PTGES3
ZNF792	HGH1
MAF	DCDC2
SLC7A11	PEX11B
CRKL	RPN1
SREBF1	FTH1P23
EFNA1	RP11-111M22.3
SLC50A1	ATP6V0E1
SHF	PSMD2
HPX	BROX
UGT2B4	C9orf16
DMBX1	SCPEP1
YIPF6	KIF23
UBE2Q2P1	PTMA
ADCY9	CANX
WEE1	CH17-13I23.3
PPARA	NDUFAF2
---	GNB2L1
GABRA2	ZSCAN16-AS1
GGCX	SPON2
NISCH	SLC25A39
KNG1	PRR11

PCDHA4
HULC
SIN3A
PTGS1
ATP2B4
RAPGEF5
CP
CHDH
LINC02348
SV2A
STC1
SYNGR1
WIPF2
PREX2

SNAP91
TACC1
MAP2
FUT11
MACO1
EIF4A2
CA9
PRRC2C
TMCC3
ZNF710-AS1
CRACD
MRPL23-AS1
TPI1P1
MAP3K20
GCNT1
AARSD1
F11R
UBE2W
CERS4
COL1A2
CDC42BPB
JAML
OIP5-AS1
CCDC80
MFSD9
AKAP9
PLXNA3
SLC6A8
PNCK
DUSP9
TMEM209
ZCCHC10
IGSF8
TEX264
CCDC28B
EMC4
MRPL47
RPL23AP82
WBP5
FAM127B
C12orf45
CRTAP
CYC1
RIPK2
NCAPG2
TMEM183A
NSMCE1
AGBL5
PLOD1
TRAIP
AC092171.4
DERL1
PYGO2
NUDT2
SLC25A6
TATDN3
ARV1
SMS
SSR1
TPPP3
NSA2
FKBPL
RP11-680F8.1
C17orf89
TXNDC12
DEPTOR
FBLN1
IFT52
TIMM17B
MIS18A
BEX2
TSNAX
NUDCD2
POLR2L
YBEY
TRIM16L

GLB1	NDUFB6
TCIRG1	UBE2A
DLX1	MAP2
UBR3	NDUFA8
NHSL1	HAUS1
HECA	UBXN1
NHERF1	ISG20
TRPV4	CPSF3
SEC31A	PDCD2L
PHIP	RPS24
ARNT2	VPS33A
SKP2	ABCB6
SNTA1	ATP6V0B
GRB7	CHMP4B
ERBB2	PES1
APBB3	NEDD8
H1-12P	STAP2
SERPINA1	MRPL21
IL18	RPL31
FGFR4	KLHDC3
KCNN3	UBA52
RGPD8	CTNNA1
EDIL3	FST
TBC1D24	MRPL15
ARFGEF1	SECTM1
PUM2	FN3K
---	HRAS
CREB3L2	THEM6
NDST2	NFKBIL1
PARD6G	RP11-369J21.12
CCDC160	GRAMD1A
KLB	RBBP4
---	BAMBI
PLOD1	COCH
CD24	RPL37A
ZFX	C1QA
DOCK7	COA3
ADGRL2	DCK
FIGNL2	DTD1
PITPNM1	RPL5
PAFAH2	TPP1
SLC25A42	RRP9
CGN	C15orf40
ZNF217	CALM3
FAM184A	DUSP23
FRAS1	LAMP2

KLF5
CIC
NBR1
RALGAPA2
PLOD2
BMP8B
MTR
PDGFD
EPHB4
ARMCX2
ACACB
RAP2A
ARFGEF2

FYB2
FURIN
LINC01451
TMPRSS9
LSS
DOCK11
GPC3
HILPDA
ZSWIM8
LAMC1
TUBB8B
ABLIM3
SLC5A9
TMEM86A
LPCAT3

LRP1B
DNM1
SPTBN1
LMBR1L
ANXA13
CENATAC
ASNS
LMNTD2-AS1
TMEM176B
ATXN1
RREB1
SLCO1A2
MTMR10
LINC02404
THSD7A
SMAD9
GPKOW
NDUFA12
C1QTNF5
DOLK
LRP11
PTGFRN
RPL4
MARCKSL1
EZH2
RP11-35N6.1
ZNF581
SEMA3G
SMOX
RPL12
ARRDC2
C11orf98
DHX9
TEAD2
SWAP70
TUBB2A
TIPRL
DCTN2
ZMAT2
PPIC
XRCC6
PNMA6A
AC016747.3
C10orf35
PDCD5
UCHL5
CD320
PHPT1
NIPAL2
SYNGR2
SNRPF
PTRH2
SNAPIN
LINC00467
TOR3A
MORN2
GRK6
ALKBH2
ERGIC3
PDIA6
THOC7
CLN6

KDM7A
TRPC1
PPFIA1
USF3
NEBL
SLC15A1
DENND10P1
HSPA6
SFTPD
DYDC2
TIAM2

GRK2
OLFML3
MARCHF2
CCDC33
SLC20A2
ARG1
SIPA1L3
CDS2
ADNP
PLAGL2
INSIG2
TRAPPC6A
PATJ
ZBED3
TTPA
TRIM71
PACS1
EBF4
TRIM2
WBP2
SEC14L6
ATF6B
MAN2B1
FRAT2
ANO4

MED12
TAB3
PDIA4
C1QTNF6
CBS
GAA
SEMA4G
APPAT
TMEM97
RFC3
UQCR10
TSEN15
CH17-340M24.3
EMP1
RP3-417G15.1
AC093673.5
ATIC
DTNBP1
RHOA
SLC29A1
LSM7
GGPS1
FUCA2
SAMM50
PSMB10
POLR2J2
TBCC
DHFRP1
TIMM10
ATP6V1D
C14orf166
CENPK
PDAP1
VBP1
ACP6
ATP6V1H
BAG6
C1orf35
BCAS4
EEF1G
IMMP2L
PMS2P1
SYNGR1
PSMC3
ARF1
BPGM
FTL
SNX15
SKA3
SOX18
TMEM164
RPP40
RPS6
FAM103A1

UBXN7
TERLR1

EPHA1
UBXN4
SPOPL
RORA
ADAP1
PGGHG
VAT1
COL1A1
ST3GAL5
SERPING1
TLR3
KHNYN
NORAD
CMIP
IGF2R
CHST11
MAN1A1

CDC42BPA
DUSP18
IRF2BP2
GNAI2
LINC00963

CNNM4
CALM3
MACROH2A2
DDX42
DLX6-AS1
PHF21A
CORO6
KMT2D
OLFM3
NHLRC3
HMX2
BPHL
WASF3
EFNA3
SEC23A
CBR4
BAZ2A
FXYD3
SLC35E2B
FIBP
NDUFA4
RP11-475C16.1
CCL18
COPZ1
RPS13
HELLS
RPL23A
TAF9
CSTB
EFCAB11
NDUFS8
RAB4A
ARF3
DPP3
PRKCSH
NUDT14
AIMP2
CTSD
HCFC1R1
RP11-977G19.10
GEMIN6
RUSC1
TRBC2
UGT1A10
TPR
ARHGEF39
CAPRIN1
LAPTM5
MAGED1
RPS3AP6
ADIPOR1
BTF3
CORO1B
HSPB11
SEC11C
FASN
RRP15
ENY2
APIP
TMED9
RP11-138I1.4
FKBP9
CDC123
CLGN
CMTM7

KIZ
ABCC9
DNAAF5
FUNDC2
EEF1A2
SNX25
ZC3H6
ZNF224
USH1C
SEMA3D
CYP4F12
TNFSF10
RAB43
SLC1A1
NONOP2
C14orf39
CKB
STAU2
CCDC170
RNF38
IL11RA
SLC44A3
KRAS
SCART1
ZNF292
SLC17A5
ETNK1
SPECC1L
CNTN4
RIPK4
WHAMMP1
DCP1B
GLIS3
CALCOCO1
WDR59
PRDM16
GLUD1
PROS1
CDS1
WDFY3
RTN4RL2
C5
HGSNAT
OBSCN
GIPR
NINL
EIF4BP7
EXOC4
RPL15
PRR34-AS1
SLC27A5
PVALB
SLC46A3
AGAP5
DTX4
CD5L
HPX
AP000355.2
KCNK5
TAF1C
SIK1
RP11-510N19.5
MYO7A
FAM150B
IGKC
RP11-715H19.2
AGAP4
LINC00969
UBAP1L
RUFY3
SLC10A1
RPS12P21
RP11-394B2.1
PODN
MT-CO2
SULT1B1
LY6G5B
IGKV3-11
ACOT12
RP11-458F8.4
RP11-286N22.16
ZNF160
ADRA2B
DPH1
TPT1-AS1
GBP7
DDX3Y
TENM1
IL4R
FOSB
CACNB2
COX6B1P4

TMEM184A
OPN3
S100A14
RCOR2
ERBB4
EPHX2
ANPEP
PEX11A
DYRK2
SLC35D2
SMARCC1
RNF145
SLC44A2
OR2A20P
TBC1D25
CDH1
TAFAZZIN
ANKRD6
TSTD2
KCNMB4
PIGZ
GCLM
EML6
BCAN-AS1
RAB20
LPIN2
TMEM170B
CNN3
LINC02177
HSPG2
GAPDHP32
CDPF1P1
NIPA1
GPSM1
ASGR2
WAC
MST1
PCYOX1
HERPUD2
PCDHB11
CTSF
FAM8A1
STPG3-AS1
UNC119B
SYNE1
LTA4H
PTGIS
SMUG1P1
CROCCP2
CTB-89H12.4
SLC39A5
CDA
FNBP4
CCBE1
ACAP3
IGHV3-15
RP11-34P13.16
MT-ND1
CES3
IGLC3
FANCC
TMEM184A
EVL
COX6A2
CPED1
ETFDH
MT-ND3
MTND2P28
SPAG5-AS1
RP11-423H2.3
LINC01320
LINC00106
ASPDH
RSRP1
SCARNA12
SLC3A1
SLC11A1
IGKV1-27
ADAT2
MRC1
FGB
ACCS
AGER
RP4-568C11.4
NDRG2
AMN
TPRG1
IGHV5-51
ENGASE
RP11-119D9.1
VNN1
RP11-347C12.1

TINAGL1	CYP3A5
CNIH1	C1R
METTL7A	PNN
CFAP95	LILRB5
SCOC	LTB4R
TMEM88	RP11-384K6.2
SCNN1A	SORL1
SERPINA6	ADAMTS1
DLC1	CDK11A
CDK14	EBF4
ZNF846	SLC25A18
KCNC3	ARGLU1
PRR15L	SH3BP5-AS1
RFK	FGL1
TRIM5	SLX1B-SULT1A4
RETREG2	AUTS2
VGLL4	TGM2
GPC1	AP006285.7
SLC45A4	GRAMD1C
ERBB3	IGKV1-9
PAQR9	SRRM2
ARID1B	PLXNB1
DUSP16	AMDHD1
CASK	AZGP1
KIAA1958	HAPLN4
LRRC37A4P	RP13-516M14.10
ARPIN	CITED2
MYO5A	FPR1
FAM199X	IGHV3-33
GYS1	TMEM25
BRSK1	IL1RL1
GOLGA2	RP11-258F1.2
HIPK1	RNF152
CLDN1	ARHGAP10
MROH1	CCL21
SERTAD2	TRPV4
AK4	APOA5
PRRG2	SERPINE1
---	VEGFA
SYT7	PRSS1
---	KAZN
C2orf15	MT-ND5
PI4KA	MCC
ENOSF1	OGDHL
MAP3K1	AKR1C8P
CLTCL1	C1RL-AS1

RAB37
AACS
IRF6
RHOT1P1
ZNF160
SMAD5
ING4
CTBS
SPACA6
AMBP
TSPAN6
NLGN2
F2
STRC
TENM1
WRN
AMFR
TRAPPC9
GLDC
HHAT
COL18A1
IDH3G
VEZF1

B4GALT5
KIF1C
FGB
FGA
PLD3
PIK3R1
BCORL1
SIX3
CBX7
ABCA2
FILIP1L
PHC1
PCOLCE2
TC2N
SGSM1
SHTN1

CASKIN1
ALCAM
PANK3
RALBP1
DLG3
CLASRP
BCL6
BCHE
CIDEB
RNF165
GABBR1
RP11-496I9.1
SPTBN2
WASH5P
PRSS2
C14orf180
MAT1A
TRPM8
RP11-42O15.3
EPHX2
SLAIN1
AFG3L1P
ADRA1B
N4BP2L1
CYP21A1P
HEPACAM
NDUFA6-AS1
PPP1R3B
ANK3
PALM3
RASGEF1B
SNRPGP15
RP11-609D21.3
CYP3A43
PLSCR4
AC009120.6
IGHV2-5
MALAT1
RP11-203J24.9
KRT17P4
PLCH2
CPEB3
NAMPT
RND3
SKAP1
SYT9
LRP5L
IGKV2-24
NEIL1
MT-ND2
SATB1

LDLRAD1	CBFA2T3
TMX4	RP11-164J13.1
ZHX2	GOLGA8N
ANG	RP11-394O4.5
ASGR1	FAM160B2
LAPTM4A	FAM151A
COPA	PNPLA7
SARAF	PHYHD1
RNF122	PHYKPL
ZMYM2	LINC00174
ZNF514	PAMR1
AMDHD1	AC004540.4
F12	FNDC4
KAT6A	MT-CYB
ZNF117	STAG3L5P
AMOT	TRIM66
GIT1	ADGRA3
TM7SF2	GGT5
DDI2	GPM6A
ADAMTS12	SEC14L4
KLC3	RP4-669L17.10
FOXH1	GADD45B
THRA	YJEFN3
HAUS4	RP11-817J15.3
---	UNC13D
ALDH2	MST1
HVCN1	DOCK5
DZIP1	EGR1
GFRA1	RP11-274B21.3
NFE2L1	ADAMTS2
SLC23A1	RP11-830F9.5
BTBD2	PBLD
KIAA1614	KCND3
RBM6	CCDC84
AS3MT	UGT2B17
DNAAF9	HK3
HIRA	GOLGA6L5P
RB1	SPRR3
EEIG2	NPY1R
CPE	EPOR
PID1	PLA2G4B
CTTNBP2	RP11-250B2.3
USP9X	BGN
TTC39C	C19orf66
GLIS2	IGLC2
TMSB15A	PTOV1-AS2

UNC13C	CYP21A2
MAP2K5	TSLP
RALGAPB	HHIP
PIK3C3	IGF1
NALT1	MTHFD2L
ANK2	MUC3A
INF2	ALDH8A1
LAMB2	ZFP36
PRKD3	SAMD5
HTT	GOLGA8B
COG3	DEFA3
SRGAP2	PEMT
CLINT1	C8A
ARRDC4	ERRFI1
DBN1	FAM229A
PLD1	RP11-256L6.3
CFAP161	PRELP
CHN2	GCH1
DHTKD1	RP11-676J12.7
TEX15	uc_338
RHOF	AF064858.6
PPP2R2C	BCL3
TEAD2	B3GAT1
MUC3A	AKAP17A
CERCAM	ATF3
ACOX1	BMPER
APAF1	JCHAIN
APLP2	RP11-166B2.1
DACH1	SNRPGP10
KCNJ3	MT-ND4
SLC16A13	EXOC3L4
TM9SF5P	LINC00659
PAPPA	MZF1
PNISR	SERPINA3
---	ACADVL
SBK1	AP006285.6
---	NEU4
POLR2A	RP11-390F4.3
MAGED2	ABCC9
ATP11A	CPT1B
SPICE1	MT1P3
---	LCN12
FMNL1-DT	AQP3
GARRE1	OLFML3
TCAF1P1	RSPO3
SLC25A28-DT	PTPRS

OSGIN2
NPC2
PLEKHG1
ZNF281
MYO1D
TTC39A
TTYH3
QSER1
R3HDM4
PCDHGB3
PLS3-AS1
HHEX
LINC01331
TTC9
NANOGP1

CPNE5
PLEKHA2
MARVELD3
SDAD1P1
ECHDC2
DENND11
SREBF2
SLC13A5
HELZ
KRT19
PI4K2B
ZSCAN2
PROX1-AS1
YEATS2
DCAF7
CCDC18-AS1
PRXL2A
TUBB2B

GAB2
NFATC4
ZNF385B
NOTCH2
DNAH11
WDR91
BTD
APLP1
CCDC183
MYO18A
TM9SF2
RP11-228B15.4
GSDMB
RP11-175B9.3
LENG8
IGLV2-8
RP11-575L7.8
LHX2
ORM2
RP1-232P20.1
ACADL
SLX1A-SULT1A3
ACADS
ADH1B
DTX1
PLGLB1
WASH7P
HSD11B1
AGXT2
WDR27
RP11-159D12.2
LSMEM1
REC8
IGHG4
C8orf46
BX842568.1
IGKV3-20
CTC-529I10.1
RP11-43N16.4
IP6K3
MAN1C1
RP4-631H13.6
C6
HOGA1
CHST4
CLDN10
CACNA1H
SMG1P7
SPG20
TIAF1
COL27A1
RP11-1259L22.2
CTD-2619J13.8
PNISR
ATHL1
PDGFRA
MUC6

NYNRIN
INPP5J
HMGB1P41
MOGAT3
TSC22D1
SLC7A2
YPEL3

NREP
TMEM94

MON2
ELOVL2
RAB24
SFXN3
TRAPPC1
CNR1
HOOK3
CARHSP1
TNS1-AS1
ERV3-1
SYNJ2BP
GJB2
MYEF2
ANKIB1
MCOLN3
PIGN
SPTAN1
LINC02846
UNC5B
CDH23
TMEM106B
CRAT
PRDX5
PHACTR4
BST2

COL4A4
AFP
ACADVL
SLCO1B1
ASXL1
PRAF2
MEGF8
PTPRS
TBC1D1
RP11-295P9.3
COX7CP1
RP11-196G11.1
USP9Y
FBP1
NPC1L1
LIPG
RP11-632K20.7
NKTR
CYP2B7P
TPPP2
PGA4
C1orf228
AL161668.5
SLC25A37
PPP1R3C
KMO
CCL23
CTC-524C5.2
OAT
HOOK2
RIC3
LPIN3
HAL
ACACB
ABCA9
RP11-115J16.1
NPIPB3
IGKV1D-39
RP4-583P15.15
F11-AS1
TNS2
LIFR
DIRAS3
FAM193B
IGLV2-11
RP11-404G16.2
SRSF11
SLC13A5
MLXIPL
AC004538.3
HPGD
RP11-238F2.1
CAMK2B
NRG1
CCL19

IGFBP1	GSTZ1
LRIG1	IGHG2
HIVEP2	MTATP6P1
RNF32-AS1	TMEM45A
FYN	KIAA0895L
PDCD4	MFAP3L
ADCY6	RP11-524F11.1
ARNT	ACSM5
HMGCS1	RP11-69E11.4
DAP	HERC2P2
PLBD2	CCNL1
ZKSCAN1	GSTT2B
PTPN9	IGLV3-25
TPI1	RCAN1
AMOTL2	TBX15
HR	IGKV1-5
CFHR1	AZGP1P1
KCNT2	STARD5
PLEKHH2	STAG3L5P-PVRIG2P-PILRB
TRIM6-TRIM34	RP3-508I15.20
TF	CYP4A11
MED13	RP1-102E24.8
PDZK1P1	CLDN2
ALDH7A1	SULT1E1
CPQ	RP11-415J8.3
DECR1	CTD-2240E14.4
SMAD7	COLEC11
---	DUSP1
LRRN1	SEC31B
PROX1	TDO2
FGG	AKR7L
WSB1	PRSS8
KDM6B	LRG1
PTPRF	ABLIM3
EPB41L1	AC138035.2
TULP3	NR4A1
MSI2	IGLV1-51
SMARCA1	SOCS3
NCOA2	TTTY14
CDK19	NTF3
HMGN4	MBL2
SERPINA7	AFM
MAPK3	IGKV2-28
RASSF5	CNTN3
USP11	ZC3H13
MMP16	RP11-499P20.2

---	CD1D
KMT2A	RP11-66N24.3
SOX13	SMIM24
SEL1L3	MT-ATP6
DCAF10	G0S2
TSPAN9	ASMTL-AS1
C8A	AASS
SCD	SYT7
PPFIA4	CSAD
TP53I11	DNAJC12
PAIP2B	GPD1
DYNC1H1	KBTBD11
HLA-C	MPPED1
EPS8L2	ID1
CCNG1	CYP1A1
BBS1	MT-ND4L
DENND4A	EFHD1
FBXO2	SLC39A14
PRLR	GNMT
KDM5B	IGHA2
---	CTB-50L17.14
GSE1	HAND2-AS1
CHD6	IGHM
PPP1CB-DT	TUBE1
FLRT2	RASD1
BBX	CTD-2619J13.27
NPC1L1	HPN-AS1
SLC35E1	C1orf168
---	CTD-3092A11.2
ASB1	STEAP4
BNIP3	PLGLB2
SPATA6	FXYD1
RXRA	CBS
GTF2IP4	SLC17A9
TNKS	RRN3P1
MMP15	ADAMTSL2
---	MAPK8IP3
TPT1-AS1	ALOX12P2
FBXO21	CFP
PCGF3	CCNL2
BRD3	IGKV1-33
DUSP22	RP11-115C10.1
PRDX3	CSRNP1
CEBPG	MYOM2
ZBED4	MT1XP1
C4orf3	ACSM3

PITX2	C14orf105
SERF2	ACAD11
PARM1	AGAP6
EPB41L4B	ANXA10
HLA-DQB1	NR1I2
UBR4	FAM134B
RNF112	STEAP3
SPECC1	SLC7A2
LRP2	CNTFR
PROC	SAA4
PPM1A	LINC01018
SNCA	ECHDC2
TRAM2	DNHD1
SYNE4	RP11-361L15.3
GATA6-AS1	GLYATL1
ACSL1	SNX29P2
ARHGAP45	KRTCAP3
TNS1	HP
TTC7A	ACSL1
RAB32	CLRN3
SEC24B	DES
SCN1B	CTC-505O3.2
STXBP4	FAM163B
MAP4K4	CTB-79E8.3
HCG27	ITIH4-AS1
NDRG2	ADGRG7
MRTFB	PILRB
ATP7A	C4A-AS1
CD9	C4B-AS1
TRRAP	PDXDC2P
LONP1	LINC01002
CMTM4	IDO2
MYO6	F9
ANO5	LPAL2
NOL4L	SLC28A1
RPS6KA3	RP4-763G1.2
RIMKLA	CYP4A22
---	AC009963.3
---	ABCA8
---	CSF3R
AARS1	NPIPB4
GLG1	PGA3
HERC2	GHR
RAB5B	IGFBP3
SH3BP2	IGLV1-40
PLS3	MT1DP

SMAD6
GNB1
OLFM2
BCAR3
AK7
HABP2
ADD3
SLC25A1
SLC19A2
RTCA-AS1
SUSD4
DNM2
NDRG4
FHIP2A
MLXIPL
NHLRC2
XRN1
SLC25A36
RGL3
CYFIP2
DYNC2I1
INSR
BHLHE40
FADS2
DOP1A
PATL1
NAB2
HEYL
NPC1

TMCC1
TMEM151A
NUMB
EEA1
ALDOA
MAN1B1
CTXND1
STARD10
CLASP1
SLC43A1
DERL1
LCN12
NOL3
NCALD
COL27A1
ORM1
PAN2
WDR72
BHMT
CTD-3080P12.3
CYP2D7
CAPN3
MBL1P
DEFA1B
BBOX1
DHODH
MFAP4
IGSF9
GYS2
MAMDC4
MT-ATP8
INMT
C1QTNF1
IFITM10
PROZ
PLAC8
DEFA1
CYP2C9
AKR1D1
TAT
GBA3
SRD5A2
PLIN5
RP11-419C5.2
EPO
PLIN1
C11orf96
FITM1
ADH4
SRPX
AC099668.5
ALPL
ESR1
DBH-AS1
FOLH1B
IL1RAP
LINC00238
CYP8B1
CYR61
FAM180A
TMEM82

DAPK1
ZKSCAN8
ENTPD4
CCDC126
SERAC1
ANTXR2
SESN3
COL16A1
CDX2
SAP25
HLCS
DPYSL5
SYCP2
ATG9A
IBTK
USP32
ATOSA
MYH10
FRK
STAG3L5P-PVRIG2P-PILRB
WDR33
PCTP
SLCO2A1
OSBPL2
LAMA5
ASS1
PKM
MYO5B
GSDMB
MEF2C
MEIS2
GPT2
SYT8
MATN3
DIPK1B
RNF24
PCDHA10
UBE2H
ZNF516
CITED2
SEPTIN6
SULT1A1
NIPBL
ADGRV1
STMN3
CRAMP1
FAM198A
RP11-328K4.1
RP11-231C14.4
ADCY1
ATOH8
AGAP9
S100A12
SLC25A27
AC006128.2
KRT13
NBPF8
RP11-622A1.2
LINC01595
XAF1
CES4A
DIO3OS
GSTM2
RP11-132A1.6
CTD-2619J13.5
FCGR2B
GREM2
GLYAT
NEAT1
MAGI2-AS3
NGFR
S100A9
SLC6A13
FAM99B
CTD-2537I9.12
FAM13A
IGLV2-14
NFKBIZ
AC068535.3
TMEM27
OR10J6P
TNFSF14
FAM83A-AS1
FLJ22763
RP11-326C3.2
STAB2
AC104809.2
C3P1
LPA
HGF
MME
KCNN2

MIR210HG
G0S2
ANO9
ADSS1
LPGAT1
BTG2
INPPL1
PIK3C2A
TMEM200B
GCC1
KCNS3
AHNAK
ABCG1
ASAP3
PSAT1
SPTLC3
PRSS53
MAST1
TPP1
LINC01163
B4GALNT4
SCX
BCL2L11
VIL1
ANKFY1
LGALS8
WASF1
SIPA1L1
SPIRE2
ST6GALNAC6
ZNF204P
GPR155
ABRAXAS1
REST
ESYT3
SMC5
ZFYVE1
ARID4A
COL5A2
TSPAN31
B4GALNT1
APOBEC3F
STON1
MTCP1
CYTH1
HAND2
GCKR
C8orf4
CYP39A1
LRCOL1
AVPR1A
LINC01348
OIT3
LYVE1
AADAT
CYP2A6
RP4-564F22.6
PLIN4
PLGLA
RP3-342P20.2
PRSS53
DPT
NPIPB5
LCAT
CYP26A1
ADAMTS13
LY6E
ANGPTL6
IGHA1
PCK1
CYP2B6
ENO3
SFRP5
RDH16
SAA2-SAA4
CXCL12
WAS
RP11-6B4.1
LINC01370
INS-IGF2
CYP2E1
CFHR3
AC005077.14
MT1A
POY
HEPN1
GNAO1
RP11-290F5.1
ATF5
MTND4P20
DNASE1L3

ZNF318
PPP1R3B
DENND10
ESRP1
SLC39A10
CYB5R3
SUN2
SBF1
MPV17L
VAMP4
MMAA
CPNE2
ABHD15
COL14A1
ADAM22
BRAF
PIKFYVE
ZNF704
JAG1
KCNJ8
RNMT
CFAP43
PNMA3
WTAPP1
TRIM7
EEF2
CCR1
VASH2
CYP27A1
TMBIM1
DNAJB2
LLGL2
SMO
KLHDC10
LINC02532
FAXDC2
CRISPLD1
FAM171B
VTN
TNFAIP1
NPHP4
USP35
FAM13A
CLCNKA
PNRC1
PCDH17
ECM1
MT1JP
OXT
RP11-434D9.1
MST1L
CCL14
SDS
GDF2
CYP2C19
LINC00844
CYP2C8
CDHR2
APOF
CXCL2
DCN
FAM65C
NNMT
APOA4
S100A8
MST1P2
CHRD
KDM8
HBA2
MEG3
MT1L
C7
DBH
GLS2
MOGAT2
IGF2
UROC1
MT2P1
VIPR1
RNA5SP216
PLA2G2A
RP5-966M1.6
NAT2
SLC22A1
ASPG
MT2A
HBA1
MT1E
HAO2
CLEC1B
BCO2
HGFAC

XPNPEP1
MYBPHL
DCAF5
HS2ST1
ADAM10
FAM162A
OBSL1
BPNT2
PLEKHA8
PPM1E
ZNF443
FAM20A
P2RY8
KIAA1549
FAM153A
LINC02701
KDM3B
TCF4
PON1

UGGT1
PRDM16-DT
VLDLR
CFH
TNK2
SPATA9
EPCAM
SBF2
ECE1
CLSTN1
IDH2
ATRN
CUX2
HAMP
AGT
PCNX2
LGI2
TEAD1
LTBP4
FAM117B
MST1P2
PPP1R26
RFX7
NAALAD2
FCGRT
MINPP1

HSD17B13
CHRNA4
ADRA1A
COLEC10
HBB
THRSP
CYP2A7
PTH1R
GCGR
SLCO1B3
IGFALS
MT1X
C9
CRP
CRHBP
CYP3A4
CNDP1
CXCL14
TTC36
SLC25A47
MARCO
SAA1
LINC01093
MFSD2A
AC132217.4
FAM99A
CLEC4M
FCN3
CLEC4G
SAA2
MT1F
FCN2
LINC01554
CYP1A2
GSTM1
MT1M
MT1G
HAMP
MT1H

AKAP6
RAB11FIP1
KIAA1143
CCDC103
ZFYVE9
FAM153CP
ZSWIM5
ZNF496
CCDC159
MINDY4
UBE2L6
CAT
ALS2CL
LRBA
CELSR1
UBXN2B
PARP8
ALS2
PFKL
ARHGEF25
BCAT1
AHSA2P
RALGPS2
TBL1X
CDHR5
MFSD6
FBLN1
AP3D1
ASAP2
PPP2R2B
CPT1A
SLC22A9
TMEM135
GOLGA8B
MEX3D
N4BP2L1
GALNT6
NR5A2
NAGS
GPCPD1
SDCBP2
CNOT11
USP22

PLXND1
STOX2

LDLR

USH2A
ZNF587
SLC31A1
RAB11FIP4
PPP1R3C
QARS1
KLF11
SHPRH
HIPK3
TMEM45B
WWC2
STC2
SCHLAP1
MXD1
INHBB

LARGE2
HIP1
NKTR
TPCN1
ABCD4
EPB41L5
ABI3BP
DMXL2
TRMT9B
SALL4
SQLE
TMEM132A
C6orf89

NCKAP5

SLC16A3
WDR26

SENP3-EIF4A1
SLC12A2
NUP58
PRKAB2
NGEF
PBXIP1
MIB1
SMAD4
GIPC2

SOAT1
LRFN1
YPEL5
EIF4EBP2
MPL
PDK1
ESRP2
SLC7A6OS
KIAA0319L
NR2F2-AS1
ID2
PRICKLE4
DENND2B
EMID1
DSP
GNAS
PARD3B
OTULINL
SLC2A1
SIAE
LDHA
AFF3
RHOBTB1
FGFRL1
ACHE
SNAP25
DCUN1D4
MPP2
GAB1
SELENOP
NOP53
CPD
DNAH10OS
SLC46A1
EXTL3
JMY
MXD3
TEF
LMBRD1
RBPJ
SDC3

R3HDM2
SEPHS2
FAM168A

CD14

RAB17
ARHGAP35
RTL10
DCTN1

LRP6
SLC22A23
AKAP11
ARVCF
DDHD2
KIF13B
ADH5
MPZL2
EXD2

LTBP3
EDEM1
MLLT10
TESK2
CFI
FHIP1A
PDGFA
TFR2
CLU
NRCAM
TNNI2
CXADR
TUG1
CDC14B
RAPGEF6
TUT4
MAP1B
SYNE3
ZMIZ1
ATF2
CARF
KIAA2013

ID2-AS1
SLC27A3
FOXN3
MISP
ALB
GNAQ

RAB3A

KLHL24
P2RY4
ZNF395
GYG2
BSPRY
GPR146
TMEM74
BCO2
TMEM63A
CRLF1
SPINK1
DPP4
TRPM3
HEG1
ALDH18A1
DBNL
NAT14
PLCE1
TPR
ATP2B2
STKLD1
HSD17B3
UNC119
ALDOC
EIF4G3
EIF2AK2

MAB21L4
RICTOR
GPAM
NR3C2
KIAA0040
GOLGA2P5
TMEM123

Figure 5D	si-NC+EV		si-KCNN3+EV		si-NC+KCNN3-OE	
KCNN3	0.512	0.52	0.338	0.251	4.534	4
BCAM	0.337743521	0.3	0.224805619	0.2	0.482215523	0.4
SATB1	0.832631474	0.8	0.549141558	0.5	1.768774339	1.7
GABBR1	0.114	0.1	0.004	0.004	0.159	0.2
MYO7A	0.008	0.008	0.007	0.007	0.017	0.02
MUC3A	0.232	0.2	0.232	0.2	1.205	1.3

Fig 5I

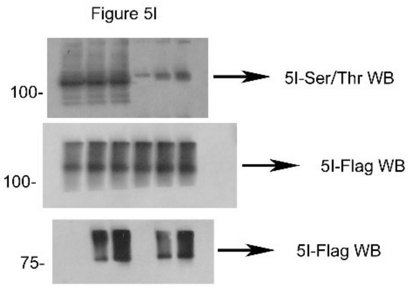

Fig 5J

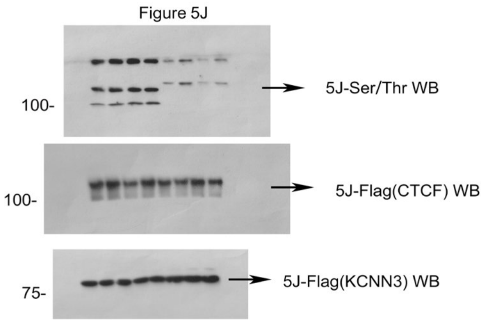

Fig 5M			
SATB1 promoter		SATB1-ΔCCCTC promoter	
EV	CTCT-WT	EV	CTCF-WT
0.777263	0.433744	1.192798	0.974234
1.078585	0.483675	0.959799	1.121828
1.126422	0.324399	1.01694	1.224395
1.01773	0.332822	0.830463	1.100752

Fig 5N			
EV	CTCF-OE	TCF-S/D-O	CTCF-S/A-OE
0.975919	0.519329	1.358493	0.254661
1.036231	0.455059	1.707825	0.243586
1.106507	0.47422	1.493893	0.311351
0.881343	0.548104	1.549368	0.259735

Fig 5E(right)			
KCNN3	SATB1	KCNN3	SATB1
4	0	1	1
4	0	1	1
4	0	4	1
3	0	2	3
2	0	3	1
3	0	1	1
3	2	1	1
4	0	1	1
4	3	1	3
4	2	1	3
4	0	1	1
4	4	3	4
3	3	1	1
4	3	2	1
4	3	1	2
3	2	2	1
3	2	1	1
4	4	2	1
3	2	1	2
4	3	1	1
4	3	3	1
0	0		
0	0		
0	0		
3	0		
0	0		
3	0		
0	0		
2	0		
1	0		
3	1		
0	2		
0	0		
1	3		
1	2		
1	2		
3	2		
1	1		
1	1		
1	1		
1	1		
1	3		
1	1		
1	1		

Fig 5E(left)

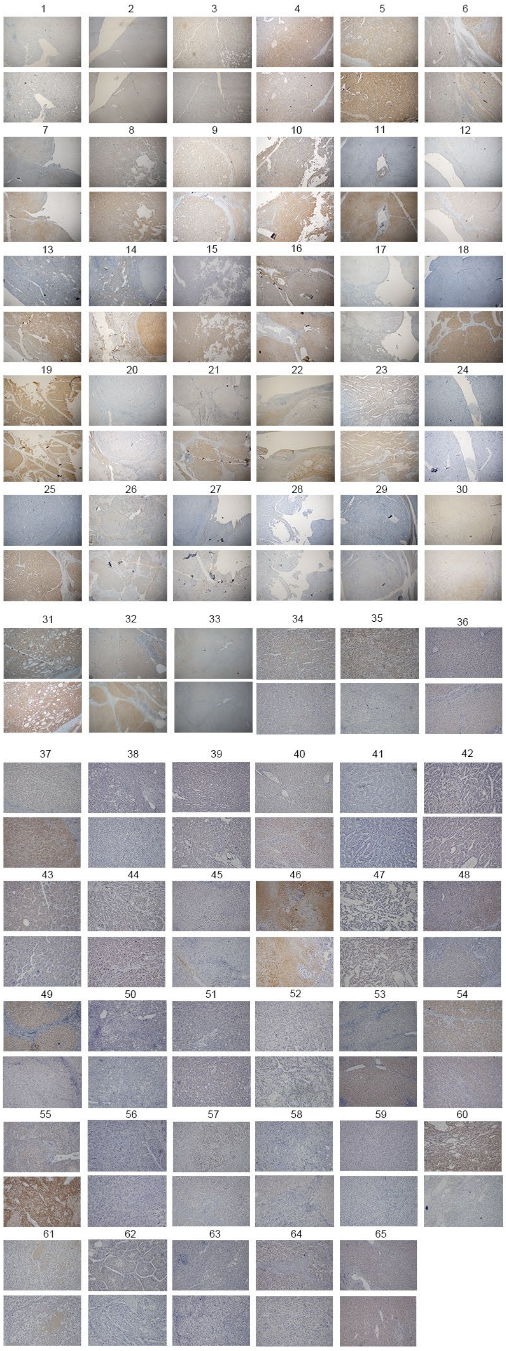
1 2 3 4 5 6
7 8 9 10 11 12
13 14 15 16 17 18
19 20 21 22 23 24
25 26 27 28 29 30
31 32 33 34 35 36
37 38 39 40 41 42
43 44 45 46 47 48
49 50 51 52 53 54
55 56 57 58 59 60
61 62 63 64 65

Figure 5F	EV+si-NC		
SATB1	2.076	3.586	5.366
KCNN3	0.472	0.338	1.826
VIMENTIN	49.452	45.929	46.443
N-cadherin	2.121	2.938	3.634
E-cadherin	4.499	4.842	1.226
β-catenin	5.428	5.815	9.462
	KCNN3-OE+si-NC		
SATB1	9.658	11.01	10.544
KCNN3	5.046	5.589	5.642
VIMENTIN	63.013	74.324	65.792
N-cadherin	8.73	6.697	10.86
E-cadherin	0.998	0.767	1.09
β-catenin	4.394	7.266	0.147
	EV+si-SATB1		
SATB1	1.119	1.194	1.747
KCNN3	0.648	0.669	1.044
VIMENTIN	28.973	22.571	21.098
N-cadherin	1.229	1.363	0.375
E-cadherin	26.766	19.814	1.232
β-catenin	30.32	1.026	92.319

Figure 5K	EV		
CTCF	0.72551124	0.897762829	0.96069258
SATB1	2.412827037	2.007392179	1.884255364
c-Myc	1.476147361	1.416652663	1.457512999
	CTCF-OE		
CTCF	5.741180842	3.165947869	5.304598283
SATB1	0.922463381	0.955950122	0.857383076
c-Myc	0.54129433	0.90639465	0.73952291
	CTCF-S/D-OE		
CTCF	2.849172558	3.448853865	2.822626033
SATB1	2.044917407	1.892308361	1.925019645
c-Myc	2.240424733	2.001336488	2.500118404
	CTCF-S/A-OE		
CTCF	6.539815793	4.138907482	2.126159752
SATB1	0.532710943	0.95037292	0.863189966
c-Myc	0.641417097	0.592780364	0.152286925

Figure 5H		
si-KCNN3 (down)	TF about SATB1	SATB1(up in HCC)
CTTN	SPI1	BBX
WRAP53	CTCF	TTC14
GMIP	ATF2	SLC12A6
ABLIM1	BACH2	SP4
GMIP	BCL6	PPP3CA
EI24	BRD3	ATM
EI24	BRD4	FOXJ3
ABLIM1	CBFB	MBNL1
WDR75	CDK7	EIF2C3
WDR75	CDK9	KIAA1109
ZC3H4	CEBPA	CYLD
LARP1	CEBPB	NEK1
SLC4A4	CREBBP	EPS15
SLC4A4	CTCF	PTPN4
BAIAP2L1	E2F1	FCHSD2
RALY	EGR1	CENPC1
RREB1	ELF1	MIER1
XPC	EP300	ZNF267
XPC	ERG	CLASP1
FRK	ETS1	TMX3
RBM10	FLI1	IFT80
SHCBP1	FOXP1	CRYBG3
ADGRG6	GATA2	PIK3CA
DMAP1	GATA3	KIAA0430
DMAP1	GATA3	RSF1
RBM5	HDAC1	KIAA0495
SRRM2	HDAC2	PIGK
CTNND1	IRF1	ALG10B
RBM10	KLF1	BAZ2B
RNF113A	LMO2	TP53BP1
RNF113A	MAFK	FAM126B
RFFL	MAX	UBR1
SMARCA4	MAZ	KIAA1468
TP53BP1	MED1	CCDC82
SRRM2	MEF2A	OTUD4
SRRM2	MYB	REV1
SRRM2	MYC	UBA6
TOM1L1	MYH11	CCNT2
RANBP3	NFIC	WDR35
SRRM2	POLR2A	EPC2
SRRM2	POU2F1	TET2
NHSL3	RAD21	TMEM30A
ARID1A	RARA	MOBKL1B
CDK13	RUNX1	FTSJD1

SMARCA4	RUNX1T1	RLF
RFFL	RUNX3	PGBD4
MIEF1	SP1	SLTM
AATF	SPI1	DDX6
AATF	SPIB	PPP1R12A
SNAPIN	STAG1	SRRM2
GATAD2A	STAT1	GCA
BCL7B	TAL1	DNAJB14
ZC3H13	TCF12	TRIO
LARP1	TCF3	ZRANB2
AATF	TFAP4	NR2C2
SNX1	VDR	GCC2
AHNAK	YY1	FBXL3
KIAA1671	BRD4	DYNC1LI2
KIAA1671	EP300	ZBTB1
RANBP3	ETS1	SECISBP2L
RANBP3	MED1	CSNK1G3
SNAPIN	RUNX1	TRIM34
RREB1	MYC	ARL13B
FXR1	CTCF	BOD1L
PURB	CTCFL	SFRS13A
PURB	EGR1	SF3B1
NOP56	EP300	PREPL
SRRM2	GATA1	ZNF397
MTUS1	KMT2A	USP24
OSBPL3	MAX	VPS39
ATG9A	PBX1	VPS13C
ATG9A	RAD21	ZMYM6
NUP98	RUNX1	RASA1
FXR1	SUZ12	SMARCAD1
RRP8	UBTF	FRYL
ZMYND8	ZBTB7A	ZC3H6
ADGRG6	BRD4	SFRS2B
NES	CTCF	ATXN7
NIPBL	EP300	PARP8
PSEN1	GATA3	NFKB1
GPKOW	JUND	NPAT
BICC1	MAX	TRIM44
MAP4K4	MXI1	FAM98B
DENND2B	POLR2A	ALS2CR8
MAP4K4	RAD21	ADAM17
TRIM28	SIN3A	SOCS4
CTNND1	SMC3	IRF2
IRS2	SPI1	ACAP2
MARCKSL1	TAF1	ZNF397OS
IRF2BP2	TCF12	ZBTB6

SUPT6H	TEAD4	ETAA1
SUPT6H	YY1	CDC42SE2
PRKD3	ARNT	CREBBP
PRKD3	BRD4	C11orf30
NOP56	CEBPB	HIPK1
SPEN	CTCF	KLHL28
SPEN	E2F1	CLCN3
ADD1	EGLN2	YTHDC1
ARHGEF5	EP300	TRIP12
CBLB	FOS	RBM26
CBLB	FOXA1	SMG1
ARHGEF35	FOXM1	SPTY2D1
ARHGEF35	GATA3	ARFIP1
TMF1	HIF1A	AFF1
BCL7B	JUN	EXOC1
LARP1	JUND	TANK
ZMYND8	KDM5B	VPS4B
PABIR1	MAX	POLI
PABIR1	MED1	RNF41
PPP4R3A	MYC	ZZZ3
ARID1A	NR2F2	RBBP6
QSER1	PGR	MAPKBP1
QSER1	POLR2A	SETD5
AHNAK	SIN3A	PKN2
LARP1	SPI1	SRRM1
ZMYND8	SRC	PHTF1
MAP1B	STAG1	CASP8
ATRX	TFAP2A	PRKD3
ATRX	TFAP2C	ITSN2
CPSF2	XBP1	RICTOR
CPSF2	CTCF	CCDC52
CPSF2	CTCF	INPP5B
KHSRP	CTCF	CLIC4
SLC19A3	MBD2	CREB1
SLC19A3	CDK9	SCLT1
SON	CDX2	PIK3C3
BAP1	CEBPB	EFCAB7
BCL7B	CTCF	C18orf25
CTTNBP2NL	EGR1	ATMIN
NSUN2	HINFP	CP110
SEC16A	MED12	EFHC1
SEC16A	MYC	FYTTD1
NSUN2	POLR2A	LUZP6
RETREG2	SMC3	CUL3
NEMF	SP1	ERCC4
NEMF	TAF3	API5

AHNAK	TCF7L2	PAPOLG
THOC5	VEZF1	ACTR2
SON	CTCF	CTDSPL2
TRAF2	JUND	KIAA0664P3
OSBPL3	CTCF	SMARCA5
SNX1	ATF2	SRBD1
SF3B2	BCOR	HIVEP2
MDC1	CTBP2	KIAA1632
HERC1	CTCF	KIAA0494
KIAA0930	E2F6	SNX13
ARFGEF2	EGR1	SRCAP
NUFIP2	EP300	KIDINS220
SON	FOXA1	RSBN1
RRP8	FOXA2	LOC100132247
GCFC2	FOXP1	ELF2
TRAF2	HDAC2	SNRNP27
CASP7	HNF1B	ARID1B
DAXX	JUND	WDFY3
DAXX	KDM4A	PHC3
SMARCAD1	MAX	PIKFYVE
SMARCAD1	NANOG	IKZF4
LRRC47	OTX2	C10orf118
BAIAP2L1	POLR2A	C14orf118
FOXK1	RAD21	MAP3K7
NOL8	RBBP5	RUFY3
NOL8	REST	DNAL1
NOL8	SAP30	ZNF500
CCM2	SIN3A	RNF141
CCM2	SMAD1	RANBP2
NSUN5P2	SMAD4	TEAD1
NSUN5P2	SNAI2	CDK17
SNRNP200	SP1	ZDHHC21
SNRNP200	SPI1	ADAT1
SNRNP200	SRF	TBC1D5
PPIP5K2	SUZ12	ZMYM1
PPIP5K2	TAF1	C21orf91
PABIR1	USF1	RBM43
LRRC47	ZFP42	SP140L
SLC9A1	FOXA2	CEP170
UBXN7	CTCF	PRKACB
EEF2	OTX2	LCMT2
LEO1	CTCF	PURB
LEO1	GTF2I	CNOT8
CLASP1	PCGF2	SIN3A
CLASP1	SPI1	ROCK1
GJA1	ETV1	MAP3K2

GJA1	CTCF	KIAA1826
RANBP2	CTCF	EIF2C4
PCF11	SPI1	HIVEP1
FOXK1	EZH1	MAML3
NUP153	JUN	AQR
NUP153	BRD4	FUBP1
RANBP2	BRD4	DMTF1
RPS6KA4	KLF1	RNF11
RPS6KA4	CTCF	ZC3H7A
ZFYVE16	SPI1	SSH1
THRAP3	CTCF	THUMPD1
AEBP2	WDR5	ZNF654
AEBP2	AR	DDX5
AEBP2	BMI1	ZFP161
FOXK2	BRD2	ZBTB11
FOXK2	BRD4	HP1BP3
AFDN	CDK9	RBM7
WWC1	CTCF	C4orf41
VCPIP1	EP300	CHD2
PCF11	ETS1	NCK1
XRN2	JUN	AP1G1
SRPRA	MYC	ARAP2
SRPRA	NCOR1	JAK1
SRPRA	PHF8	TRAF3IP1
ARHGAP17	RNF2	C14orf106
ZFYVE16	SPI1	MEF2A
PPP6R1	SUMO2	C14orf135
ITGB4	TBL1X	MFN1
RRP8	TRIM28	ZMYM2
LRRC47	ZNF263	UVRAG
LRRC47	CTCF	NFATC2IP
AKAP8L	EZH1	BAG5
AHNAK	FOXA1	PDPR
CTCF	FOXA2	SPEN
ITGB4	JUND	RGPD4
SHB	RAD21	ZNF597
MARCKSL1	YY1	USP48
TOMM20	ZBTB7A	NUMA1
TRIM35	CEBPB	ZNF646
TRIM35	CTCF	THRAP3
EPS15L1	EP300	ING3
BAIAP2L1	FOXA1	RWDD4A
CTCF	FOXA2	LOC100271836
SON	LMNB1	ALPK1
SON	MAX	GOLGB1
ABRAXAS1	MAZ	ZMYM4

ABRAXAS1	MYC	KIF3A
RANBP10	NKX2-1	SNIP1
	RAD21	MSL3
	SP1	ARHGEF12
	SPI1	MBTPS1
	CTCF	SFRS12
	BRD4	ABI2
	CTCF	GIGYF2
	GATA3	NR3C2
	NOTCH1	TNRC6A
	ELF1	SMAD1
	EP300	TRA2A
	FLI1	TMEM167B
	GABPA	INPP4A
	EWSR1	SWAP70
	CTCF	CTCF
	SPI1	ANKRD13C
	BRD4	BTBD10
	CTCF	SOX6
	KDM4C	JRKL
	BRD4	PELI1
	CBFB	WDR47
	CREBBP	MOBKL1A
	CTCF	FBXW11
	DDX5	C10orf18
	DUX4	SETX
	ETS1	ZNF24
	FOXA1	PRPF38B
	FOXP3	NAF1
	GATA1	ARHGAP5
	KLF9	KBTBD2
	MYH11	RECQL
	NR3C1	TCF12
	NRIP1	ZNF641
	OTX2	MTX3
	PAX3	DPP8
	SMARCA4	TMF1
	SPI1	DCAF5
	CTCF	TANC1
	FOXA1	ZNF92
	HNF1B	LOC400657
	SPI1	FCHO2
	CEBPB	VWA5A
	GATA1	QKI
	SPI1	SYNE1
	CBFB	CRAMP1L

CEBPA	UBLCP1
MYH11	SFRS18
MAZ	ZNF326
AR	LOC284441
CBX8	WHAMML1
CTCF	ACVR2A
ERG	SLC30A7
EZH2	HMG20A
FOXA1	UBE3A
FOXP1	RBM25
HDAC2	LOC144438
HDAC3	MFAP3
RUNX1	NSUN3
SUMO2	RSBN1L
SUZ12	SNAP23
CTCF	DNAJC10
EP300	NARG2
SPI1	ATR
SPI1	BDP1
BRD4	KDM4A
CTCF	MYSM1
CTCF	PAN3
BRD7	C14orf43
CTCF	TSTD2
FOXA2	ZSWIM6
MYC	CUL5
SOX2	ZFC3H1
SPI1	MYCBP2
TFAP2C	SYNGAP1
TP73	ZNF182
CTCF	PLEKHM1P
SPI1	MLL5
CTCF	KIAA0226
SPI1	REV3L
SPI1	EIF2AK3
BCL6	DR1
BCOR	ZNF330
CREBBP	LMAN1
SPI1	LARP7
FOXA2	RAB5A
EP300	BIRC6
POLR2A	PPP1CB
RAD21	NAB1
TAF1	ZNF434
YY1	CDKN2AIP
ZBTB7A	KDM5A

CTCF	INO80D
ELK3	LOC100129550
ETS1	PGGT1B
FLI1	ATF2
FOS	ARL6IP5
JUN	YTHDF2
	GRINL1A
	INO80
	CSNK1A1
	SS18
	ZNF192
	EIF4G3
	ZNF136
	KIAA0467
	C7orf42
	ZAK
	PPIL4
	DHX15
	GLCCI1
	TRIM52
	WDR7
	S100PBP
	BCL10
	RAB3GAP1
	PACRGL
	RBBP4
	ZBTB26
	STAG3L3
	STX12
	PAPSS1
	UEVLD
	DNAJC13
	SLMAP
	FBXO11
	FBXO34
	RPE
	LIN7C
	C5orf41
	ACTR3
	SAMD8
	CREBZF
	PRPF40A
	GLYR1
	ZBTB44
	ZNF12
	ATG2B

DCP1A
SON
RNPC3
FLJ45340
STIM2
NUDT21
DCAF17
EXOC5
SPRED1
YLPM1
DYRK1A
THAP6
LOC285033
MED13L
HECA
PARVA
USP10
PIK3C2A
CGGBP1
BMPR2
ANKRD12
GTF3C1
SUPT7L
CELF1
MAML1
FAM179B
AZI2
ZBED5
GMCL1
SFRS2IP
NEDD1
MSL2
ZFP91
PAFAH1B2
PHF21A
RAB33B
RNF4
AKIRIN1
GPATCH8
CEP63
RBM16
RGPD3
HAUS3
BMP2K
ZNF17
ARMC8

ATP2C1
ANKRD11
ORC4L
BAZ2A
MOBKL2C
SMEK2
MBD5
LRBA
TMEM184C
RP2
DIS3
PHIP
PLDN
CBL
USP34
LOC400027
ALG10
RBM27
TOP1
MAN1A2
CDC40
APPL2
TGFBRAP1
NCOA1
ATXN1L
ZNF721
SENP6
CCDC111
MEX3C
UBXN7
TEP1
CFLAR
INADL
C1orf109
PPM1B
CLK4
CCNI
ANKRD6
VRK2
NFAT5
LRRFIP1
WDR92
ZDHHC20
USP33
TARDBP
NUMB

KIAA1712
HERC2
RTF1
ZNF484
HIPK3
TNRC6B
PPP3R1
DNAJC27
EVI5
RBM12B
ATF7IP
OGT
STK4
CTBS
OXSR1
DPY19L1
MON1B
ZDHHC17
RSPRY1
FAM13B
C15orf29
MDM1
NCKAP1
ZNF800
KPNA6
MUDENG
BRD8
IMPACT
SLC7A6OS
DOCK1
G3BP2
RCHY1
EYA3
RFTN1
SMC4
LEPROT
ZNF236
CLINT1
ZEB1
ERBB2IP
AHCYL2
CASC4
EHBP1L1
PHF12
CRLF3
NPHP3

SEPT7	
USP15	
CWC22	
BRMS1L	
KTN1	
SMARCC2	
ZFP106	
CMTM1	
MTF1	
	7-Mar
HIAT1	
RQCD1	
UTP3	
KRR1	
ZNF680	
CDK11A	
BCLAF1	
PJA2	
PUM2	
CCNT1	
AFTPH	
MATR3	
FBXO33	
PCF11	
C2orf49	
GSTCD	
C2orf67	
RAB2B	
MKL2	
TBRG1	
RYK	
SOS2	
UBA3	
SGMS2	
PPP2R5E	
DNAJC24	
KIAA1009	
ZNF37B	
TMEM170A	
TTC30B	
PWWP2A	
FBXW7	
AKAP11	
GOPC	
DENND5A	
KIAA0090	

PLSCR4
NDE1
KIAA0240
XRN1
C2orf60
ARL6
CHD6
PLEKHA3
WDR36
LATS1
TMEM106B
TRA2B
APPBP2
PAPD4
BRPF1
CYFIP1
ERCC3
FAF2
PNRC2
CHIC2
NAA30
SPATA13
RSRC1
CDC42BPA
LUC7L2
TNPO1
SKIL
MBD1
MDFIC
RIF1
C3orf17
MED1
ZNF319
CHD4
MAP2K1
YIPF5
MIOS
SAMD9
PKD2
ZNF621
HNRNPH3
USP42
C19orf55
TAB2
RBMXL1
C11orf57

TRAF6
SEPT2
RNF111
RAB28
TMOD3
ARID1A
EXD2
LASS6
LEMD3
SP1
SETD7
DHX36
ZNHIT6
RBM15
GTF3C3
TTC21B
HNRNPUL2
ZNF107
LIMS1
ZNF142
ZCCHC11
SNRNP200
OBFC2A
PDLIM5
BBS10
ZNF407
MRE11A
PARP11
PER2
BPTF
CAB39
FRG1B
RNF169
C4orf29
MED14
AGTPBP1
ZNF384
BBS4
TAF1C
SOCS5
CWF19L2
ZNF776
TRIM62
PKD1
DCBLD2
ZNF175

NEAT1
IREB2
GRSF1
MAP4K5
GPD2
RFX7
ZNF785
CLCC1
SPG11
MLL3
TBC1D8
NAA40
ICMT
RAPGEF2
PPIP5K2
ZC3H4
KDELC2
MLL
BTBD12
REST
ZKSCAN5
C3orf38
PRDM2
C2CD3
OFD1
PAQR3
YTHDC2
CCDC14
ATP11B
RNF146
FCF1
RBAK
PPP1R8
GTF3C4
PARP3
G2E3
SFRS1
INPPL1
WDTC1
SLAIN2
ZNF616
SFRS4
LRRC8B
SP3
MLL2
ZBTB25

ZNF224
CDYL
NECAP2
CLDND1
TJP1
VPS13D
TRRAP
TRAF1
TTC30A
CEP120
GPATCH2
MKLN1
STRN3
WDR82
POLR2A
RPAP2
GMEB1
USP8
MTA2
RNF145
BCL9L
MORC3
LENG8
GOSR1
CHTF8
TAF13
PRR12
DDX3X
AHDC1
BTN2A1
SPCS3
NSD1
ANAPC1
RSPH3
SETD1A
BAT2L1
CASD1
NUPL1
ZBTB43
ADAM10
SLC30A6
AAGAB
ARF3
KIAA0947
RNF6
CADPS2

USP38	
PCNP	
ANKRD36	
HELZ	
ATAD2B	
LSG1	
PIK3R4	
IQGAP1	
	6-Mar
ZFHX3	
STXBP3	
SGPP1	
POLK	
BBS7	
NIPBL	
SLC35A5	
C9orf102	
KIAA0586	
ZNF417	
DPY19L3	
ARID2	
TRIP11	
MZF1	
N4BP1	
ANKRD32	
CPSF7	
CBLB	
SEMA4B	
IQCB1	
CBFB	
TNFAIP8	
EIF4G2	
TBC1D9	
RAPGEF6	
HEATR5B	
KIAA1012	
CEP290	
TRAK2	
SH3GLB1	
TRAF3	
MYNN	
FRMD6	
TNPO3	
ANKRD5	
METTL4	
ZNF718	

ZNF41
HEXIM1
GUSBP1
PDCD6IP
C11orf61
CWC25
ZNF548
PRMT10
GOLGA2
LOC388692
USP28
RRN3
TRIM23
CNOT4
SYNRG
SP100
DOCK10
NUP54
TM2D1
ZNF791
FRMD4B
MAP3K3
DDX18
GNG12
GALNT10
SLC35F5
FANCM
BTBD1
LRCH1
SAV1
ZADH2
FAM188A
ANKRD42
ZNF20
ARPP19
RFWD3
C12orf51
IFT57
ZNF638
NFKBIZ
NAA15
SCAND2
RNF160
ATP10D
B3GNT2
CCDC50

KIAA0562
BTF3L4
SRPK2
TRIM32
BACH1
ORC2L
WHSC1L1
GLG1
GPBP1L1
VPRBP
HERC3
GSK3B
ZDHHC7
STAG1
PLEKHA2
STAM2
HNRNPU
WDR73
ZBTB40
CPSF2
MACF1
C14orf138
ENTPD4
SELT
SLC30A5
QSER1
TCP11L2
KIAA1267
SLC35E2
HNRPLL
RIC8A
FAM111A
GFPT1
VKORC1L1
SR140
CPSF6
MYST4
EPM2AIP1
LIN54
ZNF148
ZNF138
USPL1
ITGAV
CPNE8
ZNF23
JMJD1C

FAM119B
CHD1
PTP4A2
LOC100132707
TRIM56
HEATR5A
HMGXB3
TRIM5
FYCO1
IPO8
PIBF1
WDR5B
RAPGEF1
ATL3
TLR3
SCYL2
SLU7
ZNF619
TMX1
METT5D1
C11orf46
AP4E1
SFRS11
HNRNPR
TMEM87B
MEIS1
CCNDBP1
TRANK1
ZKSCAN2
SMNDC1
CAMK2D
ANXA5
ZNF276
BTBD7
LRCH3
MFSD8
DUSP18
TCERG1
SPIN3
TNIK
KIAA1530
KCTD18
AEBP2
CSTF2T
SAMD9L
ZDHHC5

RAB12
COPB1
CDV3
KHDRBS1
FGD6
C10orf26
CNOT6
SMAD2
LINS1
CCDC55
NLRP1
SNAPC3
NCBP1
HIVEP3
GAB1
TERF2IP
UBN2
HNRNPK
FBRS
PRDM10
ZFYVE16
STAMBP
ZNF689
SPRED2
ATRX
NBEAL1
MIER3
ZNF609
HARBI1
CAP1
SDCCAG1
VPS8
PHRF1
TAF1
NOL9
C9orf80
GTF2H3
PUM1
CASP8AP2
KPNA4
HNRNPA2B1
FKBP15
SETD1B
ERC1
JHDM1D
ZKSCAN4

RNF214
SART3
SGK269
KIAA0776
EEF2K
ARHGAP29
C16orf72
RBM5
FAM65A
PCNX
SH3BGRL
PMS2CL
RFX1
GPR176
COL4A3BP
ODF2L
GTPBP8
STX17
MSI2
SYNJ1
PNN
WDR19
STK36
NHLRC2
AP1AR
ZNF639
SRGAP2
PRKRIR
NIPAL3
SPATA5
TMEM194A
AKAP13
STRN
CHD8
CHD9
KRCC1
TNKS2
DNAJB4
ALS2CR4
HNRNPH1
KIAA0831
SKI
ABL1
UBE2H
PTGFRN
TBC1D15

MBP
CYR61
SMC1A
RAB23
POLR3E
RBL2
FOSL2
FAM116A
FAM48A
ROD1
CNBP
USP4
SMCHD1
C1orf103
ZC3H12C
RERE
HNRNPD
USP46
LDB1
RGPD6
SLC30A9
AGFG1
AASDH
CSDE1
YY1
FANCF
AFG3L1
C4orf21
C5orf44
MAPKSP1
WTAP
SUPT6H
ZBTB49
PLCL2
HCFC1
AHNAK
TRAM2
AKAP2
CEP164
RRAGC
SMURF2
ZNF317
MECOM
RNMT
TYW1
CALCOCO1

AAK1
GEMIN5
NUFIP2
GNAI3
RBM33
DYNLL2
TNS1
ARHGAP23
SF1
WDR11
C3orf63
ZNF354B
RNF44
UBXN4
ARIH1
BBS1
KIF2A
NOC3L
ASXL2
COPB2
RCOR1
PDCL
EID1
RBM14
C1orf83
WWC3
GDE1
FAM118B
SENP1
TBK1
CLCN6
TAF1B
BNIP2
CCNG2
ZFYVE20
ZNF644
KIAA2026
EPC1
KDM3B
TAX1BP1
NUP98
ANKRD50
HERC2P2
SLC12A4
BAZ1B
POLR2B

CAMSAP1
C7orf60
JAK2
TSHZ1
MAST4
UNKL
CNOT2
FKTN
PURA
TMEM43
FBXL17
STAG3L2
YPEL2
UGCG
TTF2
ZCCHC4
ZNF778
ATN1
PIGN
LRIG2
CAND1
TLR1
AGGF1
DOCK4
SASH1
FTO
TMEM87A
BAT2
TIA1
KDM5C
FEM1B
C14orf105
TECPR2
MTMR2
ZNF708
CAST
VHL
MSRB3
GEN1
FAM73A
FBXL14
NF1
FLI1
MTF2
CDK13
TWISTNB

C16orf57
SAMD4B
GPR107
HSPBAP1
OSMR
SMAD4
DUSP11
PALB2
ATG4C
SACM1L
TMED4
STK17B
SENP7
KIAA0317
ATF1
NKTR
ASTE1
CEP110
APC
PMS2L11
ZNF292
CLASP2
ZNF629
MCM9
PLEKHM1
GTDC1
ZNF436
LOC645676
FPGT
SNAPC1
MPHOSPH9
ZNF280D
WWC2
WDFY1
CAPZA1
NUS1
CCNL1
COPS8
KIAA1328
RSRC2
WDR52
TRAPPC6B
KIAA1949
RHOT1
FKRP
FAM114A2

YWHAG
ZNF493
TRIM66
KBTBD4
ATXN1
APH1B
ABCC1
KDSR
GTF2A1
EPHA2
CMTM6
MLLT6
LOC441208
PVRL3
IRAK4
C6orf72
LUZP1
CDC42EP3
C16orf63
LOC146880
SNAPC5
C1orf144
ALMS1
ZNF225
SEPT10
C8orf4
BCAR1
ADO
ZBTB7A
SLC4A7
MFSD1
MCL1
DCUN1D1
QTRTD1
ZNF587
PRKRA
FBXO42
BAHCC1
ARL6IP6
KCTD10
ORMDL1
FBXO30
RC3H2
MAPK8IP3
ZMPSTE24
YEATS2

LOC647979
SUN1
ASB3
IL15
RNF168
SNTB2
CCDC93
CLOCK
CYTH3
SERINC1
ALKBH8
UBE3C
PPWD1
EP300
SNX6
HDHD2
CHMP2B
BICD1
ELF1
NIPA2
TSPYL1
TFCP2
C12orf23
C7orf23
TMEM127
FAM192A
EXOSC10
AKAP10
DENND4A
PTAR1
DCP2
ZNF19
ARID4B
MAFK
ARHGAP42
CCDC132
ZFP90
DMXL1
SPAST
KIAA0182
MFAP1
CTTNBP2NL
PHLDB2
PCSK7
SF3B3
TOPBP1

SOCS6
DHX40
USP9X
USP25
MPHOSPH8
EIF2C1
SIAH1
MGA
ETS1
WDR41
DDX23
HNRNPA3
CDC42BPB
TAOK1
KDM2A
USP32
NRF1
MAPK1IP1L
GNB1
MYO6
MRFAP1L1
RABGAP1
ZC3H18
FAM172A
BAZ1A
RNGTT
C13orf23
SDAD1
MKNK1
SPAG9
RUNDC1
ANAPC10
SIKE1
DCBLD1
TTC8
RB1
LYSMD3
ATP7A
VPS41
DDX42
MED23
SEC14L1
PLEKHM3
GTF2IRD2P1
SLC25A24
ZNF532

SFMBT1
RCBTB2
SFPQ
ANKRD28
RAB14
CCDC76
RIOK3
MTMR9
SFRS2
RBM12
PTPN21
C15orf57
RIT1
APPL1
OAZ2
TRIM2
HMGN4
PPP1R2P3
ASH1L
FAM102A
MAPK1
SF3A1
DAZAP2
ACVR1
GSPT1
MAPRE2
FAM117B
LRRC57
SBNO1
NR3C1
CHST14
GABPB1
RNF207
MYLIP
JKAMP
FNBP4
C14orf4
UBP1
ETV6
GIT2
ZNF510
NUB1
PGBD3
C13orf31
TXLNG
ESYT2

INTS2
PDGFRA
POM121C
JMJD7-PLA2G4B
TBC1D23
ATXN2L
PASK
RHBDD1
CSNK2A1P
COG6
DCLRE1B
ABCE1
NAA35
YWHAB
DHFRL1
GNL3L
CIC
MBIP
PHF13
C18orf10
ARHGAP27
RAB5B
ASAP2
VEZT
CCNK
BIRC2
RBM45
TMED8
ZNF26
CDK11B
LUC7L3
TRAK1
EIF4H
MKRN1
CYBRD1
PPIP5K1
ZBTB17
LRRC8C
RNF180
SEC22B
PNPLA8
FGFR1OP2
ZNF180
NMD3
USP3
HAUS2

IPP
ZNF264
MAGI1
TBL1XR1
FUT11
ADNP
RNF2
ZSCAN29
TCEB3
DNAJC14
MED17
TRIM38
HELQ
DARS
RIN2
PRRG1
DPF2
FAM76A
CSNK1G1
MGC2752
BRD2
XRCC5
FBXO38
UBTF
ANKS1A
UBE2E3
POLM
TMEM165
RAB21
TMEM194B
PHACTR4
CALU
LOC100170939
ZFYVE26
SEMA5A
HIATL1
PCNT
LRRC41
NHLRC3
QRICH1
FAM193A
GK5
ADRBK1
LRRCC1
OGFOD1
HOOK1

SEC24D
PRPF4B
POMGNT1
PER3
TYW3
DAPK1
FAM76B
MARK3
MITD1
LOC550112
ZBTB24
ECT2
GTF2H1
NECAP1
SPA17
UBFD1
ADPGK
ZBTB3
DNM1L
ZNF207
PHLPP2
GGA3
LOC440354
CRY1
COPS2
MYO5A
ZBTB8A
VEZF1
PPP1R12B
PHF6
WIPF2
SPTLC2
SEPT8
ZNF845
PRDXDD1P
SLC10A7
SEC23IP
UIMC1
ZNF146
UBE2J1
KIAA0232
ANKRD40
EMB
ABCC5
KLF3
ARHGAP17

CDK12
FAM135A
HUWE1
RNF19B
TTC5
GTF3C2
FBXL19
PPP1R2
GDAP2
SLC4A1AP
TMEM128
WNK1
DLG1
TRIM41
FBXO36
IFI16
UBE2D3
ZNF362
ANKRD17
ZBTB4
TRMT11
MRFAP1
LOC100133331
POT1
BTN2A2
PGAP1
BRD7
MARK2
MAPK8
USO1
ZC3H15
DCK
CSGALNACT2
MAP3K1
HEG1
TOP1P1
PML
TNFSF13B
CCDC115
ITGA3
KIAA1033
CCDC66
ST8SIA4
KLHL36
BCL2
ZNF187

MTMR6
FBXL20
POLR1A
KIAA0753
NOTCH2
HMBOX1
STK10
AP1S3
BBS9
AGRN
C5orf51
ZNF567
WDR3
DDX19B
FANCL
DYRK2
ZFHX4
STX7
WDR60
USP45
DFFA
EZH1
MON2
DOCK5
CLEC16A
RASSF5
NCAPD3
METTL14
SYNCRIP
UBE2Z
WDR48
WDR37
SPOPL
FKBP14
C11orf58
ZNF410
PDS5B
LIG4
NXF1
PRRC1
ZNF558
STK11IP
C5orf43
DENND2D
CDK14
PARN

MAX
ACBD3
NCDN
DDHD2
AHI1
CASP1
UBN1
GBE1
DHX38
ZNF160
HEATR6
C20orf194
ARL5A
COG7
SERINC3
FUBP3
POC5
PRKAA1
CDR2
THAP2
ZFR
WWP2
TTC9C
ATG16L1
SLC7A6
BET1L
SERTAD2
ZNF805
EAF1
ZNF767
SLC5A3
ZNF608
DHX8
ARHGAP1
AMMECR1L
PLSCR1
WDR45L
ADNP2
FBXO28
ZUFSP
CDH2
NEK7
SLC9A7
DDX46
ETF1
ARPC2

HNRPDL
KLHL8
EDC4
ARID4A
RELT
UFM1
FRS2
PARG
MUS81
ARMC9
ZNF211
TM9SF3
EXT2
FAM102B
ZMYND8
ZNF670
RBMS2
CXorf38
KLF7
FRMD8
KIAA0907
GNPDA2
RLIM
RNF38
USP47
TMEM39A
KIAA0355
AHCYL1
TMEM185B
YES1
ARHGAP32
TFAM
RALGAPB
CCPG1
TRNT1
PPP1R9B
SNRK
OSBPL2
CD164
R3HDM1
TLR2
C7orf46
CMIP
STK38L
ATG5
SGTB

TXLNA
EPB41
SBF2
NFIB
CAPRIN1
MTRF1L
SCRN3
PRPF38A
15-Sep
SYNPO
ARMCX2
SLC39A6
ELMO2
DDB1
SLC25A46
STK17A
WSB1
HMGXB4
ZFYVE9
EIF4E
OBFC1
RAD50
CCDC75
RAPGEF5
FAM168A
TMEM133
TUBGCP5
CTSO
MALT1
ZNF230
SMARCD1
SMAD3
LOC148189
C10orf84
ZNF516
IPMK
NOD1
NUP160
NCBP2
GIN1
ARHGEF6
RGP1
KCTD7
CKAP5
MTMR3
PAK2

CCDC88A
ITPR3
ASPRV1
SERPINB9
METTL2B
POLE3
RAB35
USP19
OPA1
PRPF39
MYST3
GNA13
PHC1
CTNND1
MMADHC
PGS1
PITPNM1
RNASEH1
TUG1
ACAD8
KIAA1430
NUP153
PARP14
AASDHPPT
TMTC3
SMAD7
CPEB2
RAP1B
TMEM188
CRTC3
ZNFX1
WAPAL
LOC642852
CCAR1
NFE2L2
ERMAP
ATP6V0A2
TOR1A
FAM3C
XPO6
CEP192
BAHD1
ZNF2
TSPAN3
LAPTM4A
PTPN12

SCOC
MEIS3P1
PRKCI
DCAF12
MOBKL3
C1orf52
EHBP1
GIMAP2
XPO1
FAR1
CAPRIN2
SUDS3
SLFN5
CBX5
TMEM48
UTP20
YWHAQ
KIAA1704
IFT140
TCF4
KLHL9
FASTKD2
POFUT2
TRUB1
ZNF664
DMXL2
KIAA1370
C15orf58
PPP6C
PTPN11
GON4L
C5orf15
GRIPAP1
ZW10
STXBP5
DCAF16
PHF17
FMNL3
TRIM13
EIF4E3
KLHDC5
C3orf19
RNF40
NUP43
LOC401397
OSBPL8

SLK
EML3
GNB4
ZBTB41
AMPD3
MBTD1
CCDC96
TLK2
ZNF248
C3orf64
ZNF8
DCUN1D3
ZNF543
RPAP3
TCP11L1
VPS11
KCTD9
KIAA0247
C1D
EDEM1
SFRS7
ACVR1B
MDN1
DTWD1
RGPD1
PANK4
IWS1
MIB1
EPS8
ATAD1
C12orf4
TMEM25
PEX26
C2orf43
WDR90
WIPF1
RAB8B
KCTD5
PBRM1
PDCD10
GAPVD1
ZDHHC8
CYP20A1
TGOLN2
SMC5
ZMYND11

VPS24
LMBR1
LCORL
WHSC1
SNX19
THAP1
C1orf58
TSN
FAM160A2
TWSG1
C15orf44
TRIM4
TUBGCP4
BMI1
MKS1
ZNF304
SLC37A3
LRRC37A2
CCDC102A
UACA
ZNF195
PLK4
ELMOD2
PDIK1L
CREB3L2
LOC729082
GCC1
TMCO4
ZNF841
GPN1
HIF1A
KIAA0649
USP1
PATL1
TTC37
TET3
TSPYL4
RAD18
DYNC1H1
BMS1
SF3B2
PTPN9
FAM104A
SSFA2
NUP35
FAM200A

BAT1
ESCO1
ENTPD1
TECPR1
GUCY1A3
SYNE2
ZNF202
MYH9
KIAA0174
AFF4
PIP5K1A
AFAP1L2
SH2B3
PTPRA
NMI
UBTD2
CCDC121
CLUAP1
ZNF562
PDGFC
SLC2A11
CTDSP2
HUS1
SFRS6
PIGG
SPG7
KIAA0652
BSDC1
MBD2
URGCP
ZFP36L1
KIAA0895
SLFN11
ZNF830
KLHL20
CASP4
TBC1D19
MSN
GTF2E1
MAP3K14
HACE1
HDGFRP3
TOPORS
FXR1
PTPN1
CHM

RBM22
WRN
DZIP3
ANKIB1
FZD6
MFSD9
PHTF2
KLHL24
GOLGA3
ARL2BP
UTP14C
ATP6V1A
TXNDC12
EFEMP1
IKBKB
SUZ12
NUP107
ELK3
ANKMY2
ARCN1
SDCCAG8
IFT46
GNAI2
METTL8
REEP3
PPIG
MAT2B
CCDC84
ZNF674
INTS4
SFRS5
SNX25
MYO5C
TLK1
FBF1
EIF2AK2
RPS6KA4
NCRNA00115
DGKA
ZNF550
KIAA1279
KATNAL1
CCDC99
PDCD7
CDC42SE1
C3orf34

NDFIP2
UHRF1BP1
UBL3
SLC35A3
RABL2A
ZNF324B
ADCY7
TLN1
RAB3IP
ZZEF1
RARB
MED18
ZNF193
WBP11
PIP5K1C
SEC24B
SP2
LOC100129637
STX2
KIAA1958
ADAMTS9
NBPF1
CARD6
STARD3NL
TES
ITGA4
DNAJC6
METTL9
TMEM131
TMEM17
ZC3H11A
MYST2
ATE1
TADA2B
GORASP1
PPCS
CDK8
ANKLE2
CXorf23
ANKH
NLK
NEO1
SYNJ2BP
C2orf68
SH3RF1
ARHGAP24

PLA2G12A
CHMP5
NDNL2
MADD
TRAPPC10
GABPA
ZNF512
TATDN2
BTN3A3
WWTR1
ZNF592
SEPN1
ZNF79
ZNF432
UBE3B
TSC22D2
KCTD11
CAMSAP1L1
USP12
HINFP
TOP2B
SPTLC1
AKD1
RC3H1
PLK3
DSG2
MNDA
GRB14
HSPA13
SLC36A1
SENP2
ANKFY1
ZNF273
MKL1
BET1
ANO5
SLC9A9
C17orf85
LZTFL1
TFE3
TAOK2
CLK3
C11orf49
MESDC1
TMEM167A
KIAA0922

DDHD1
BICD2
ZNF496
ATG12
RAB10
DOCK7
FGD4
MASTL
ZNF765
TRIM37
PEX13
CENPBD1
DCAF7
SMEK1
C4orf33
PUS7L
ISY1
LATS2
SASS6
DENND1B
CAV1
SRFBP1
PAPOLA
ARHGAP21
EP400
TASP1
AP3B1
ZNF223
ARGLU1
SPOP
PIP4K2A
C2CD2L
MTMR15
RDH14
SIRT1
RUFY2
RFX5
LYRM2
ZWILCH
NRAS
LMBRD2
ZNF546
M6PR
NCRNA00174
C22orf30
NPEPPS

NEXN
GBF1
FKBP9
DCTN5
DBR1
GATAD2B

**Figure 6A**	EV	EV+KCNN3-OE	SPOP-WT +KCNN3-OE
Migration-Huh7	588	970	653
	532	980	685
	585	953	611
Migration-HepG2	592	679	578
	573	754	519
	573	723	452

	SPOP-M35L +KCNN3-OE	SPOP-D153Y +KCNN3-OE	KCNN3-△SBC +EV	SPOP-WT +KCNN3-△SBC
Migration-Huh7	1024	1179	802	821
	1093	1104	831	790
	1099	1092	793	720
Migration-HepG2	721	797	722	794
	703	829	722	722
	721	847	747	742

**Figure 6B**	EV	EV+KCNN3-OE	SPOP-WT +KCNN3-OE	SPOP-M35L +KCNN3-OE
Invasion-Huh7	618	940	550	878
	591	825	680	988
	572	935	678	927
Invasion-HepG2	511	688	566	702
	445	685	455	704
	554	761	453	694

	SPOP-D153Y +KCNN3-OE	KCNN3-△SBC +EV	SPOP-WT +KCNN3-△SBC
Invasion-Huh7	1104	931	927
	1165	977	920
	1228	980	934
Invasion-HepG2	759	705	675
	786	680	762
	786	649	658

**Figure 6C**	EV	EV +KCNN3-OE	SPOP-WT +KCNN3-OE	SPOP-M35L +KCNN3-OE
Huh7	0.080	0.218	0.090	0.313
	0.081	0.223	0.090	0.338
	0.081	0.214	0.088	0.328
HepG2	0.196	0.251	0.115	0.282
	0.165	0.245	0.163	0.297
	0.157	0.268	0.129	0.303

	SPOP-D153Y +KCNN3-OE	KCNN3-△SBC +EV	SPOP-WT +KCNN3-△SBC
Huh7	0.342	0.250	0.230
	0.337	0.259	0.233
	0.376	0.246	0.222
HepG2	0.331	0.270	0.270
	0.347	0.279	0.271
	0.328	0.268	0.288

**Figure 6D**	EV +si-NC	KCNN3-OE +si-NC	KCNN3-OE +si-SATB1	CTCF-OE +si-NC
Migration-Huh7	739	1148	774	577
	759	1110	812	578
	857	1127	771	605
Migration-HepG2	934	1147	836	746
	781	1179	865	692
	864	1118	995	645

	CTCF-OE +si-SATB1	CTCF-S/D-mut +si-NC	CTCF-S/D-mut +si-SATB1
Migration-Huh7	484	967	789
	425	1075	837
	412	1275	808
Migration-HepG2	563	1157	854
	570	1268	934
	622	1225	912

**Figure 6E**	EV +si-NC	KCNN3-OE +si-NC	KCNN3-OE +si-SATB1	CTCF-OE +si-NC
Invasion-Huh7	786	1084	778	518
	691	1037	692	604
	799	1125	701	527
Invasion-HepG2	924	1121	886	754
	851	1186	905	656
	811	1151	926	703

	CTCF-OE +si-SATB1	CTCF-S/D-mut +si-NC	CTCF-S/D-mut +si-SATB1
Invasion-Huh7	416	1139	728
	416	1099	749
	463	1036	696
Invasion-HepG2	534	1142	926
	600	1103	850
	607	1140	905

**Figure 6H**	EV+si-NC	KCNN3-OE +EV+si-NC	KCNN3-OE +SPOP-WT+si-NC	KCNN3-OE +SPOP-D153Y +si-NC	KCNN3-OE +SPOP-D153Y +si-SATB1
Bioluminescence intensity ($x10^4$ photons/sec/mouse)	5060	9890	4240	17600	12100
	6880	13000	5000	25400	12900
	4230	11400	5970	22500	8750
		11400	5960	18500	

Figure 6I

days	EV+si-NC			KCNN3-OE+EV+si-NC			
0	14.7	16.1	17.3	14.7	16.5	18.3	19.1
7	20.1	21.6	22	17.8	19.6	21.4	22
14	20.3	22.4	23.7	18.7	20.5	23.4	23.2
21	19.6	21.7	21.8	18.7	20	23.4	22.9
28	20.4	22.4	22.9	19.1	19.8	23.5	23.5

	KCNN3-OE+SPOP-WT+si-NC				KCNN3-OE+SPOP-D153Y+si-NC			
0	17.5	15.8	16.1	14.2	18.3	17.2	16.4	15.7
7	20.8	21.2	19.5	17.7	22.1	21	20.5	19.3
14	22.5	22.2	20.5	18.4	22.9	20.5	20.7	19.2
21	20.6	22.1	20.8	19	22	19.9	19.7	18.9
28	21.2	22.9	20.6	19.3	22.8	18.8	19.3	19.5

	KCNN3-OE +SPOP-D153Y+si-SATB1		
0	18.5	16.4	16.7
7	22.6	20.2	19.8
14	23.4	21.4	20.6
21	24.5	22	21
28	24.9	22	21

Figure S1C

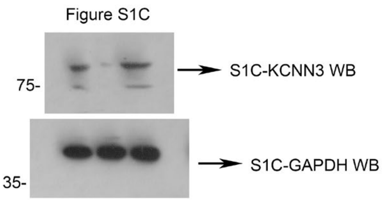

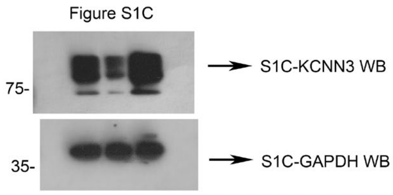

Figure S1D

huh7	si-NC+EV		si-KCNN3+EV		si-NC+KCNN3-OE	
	11.28	12.658	5.955	5.385	127.453	130.749
hepG2	si-NC+EV		si-KCNN3		si-NC+KCNN3-OE	
	10.332	12.654	3.367	2.559	134.552	130.648

Figure S1F

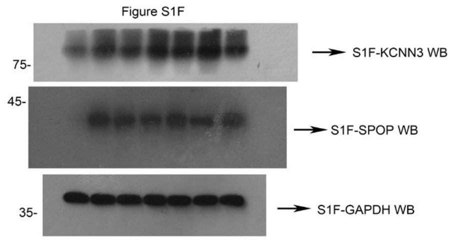

Figure S1H-I

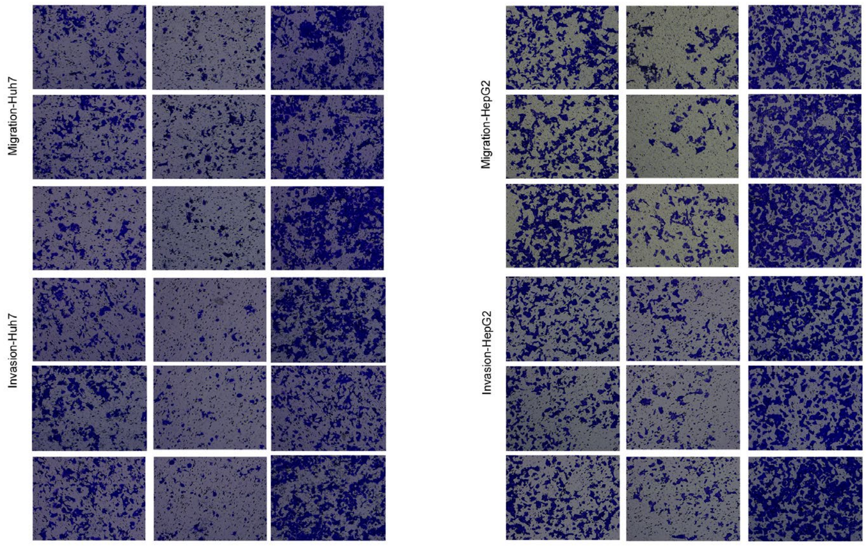
Migration-Huh7
Invasion-Huh7
Migration-HepG2
Invasion-HepG2

Figure S3O

	IgG			
Flag-KCNN3+DMSO	1.088613	0.799139	4.646516	0.247387
Flag-KCNN3+BAPTA-AM	0.246306	2.011904	0.802171	2.30269
Flag-KCNN3-ΔSBC+DMSO	0.445681	0.798084	0.613822	0.249302
Flag-KCNN3-ΔSBC+BAPTA-AM	0.153952	0.130753	0.127581	0.100858
	IP			
Flag-KCNN3+DMSO	250.9087	227.3788	249.8795	142.3953
Flag-KCNN3+BAPTA-AM	304.4976	302.045	310.6083	332.8635
Flag-KCNN3-ΔSBC+DMSO	160.8251	128.1773	108.118	68.35219
Flag-KCNN3-ΔSBC+BAPTA-AM	203.8463	268.8314	246.2796	223.5455

Figure S4A

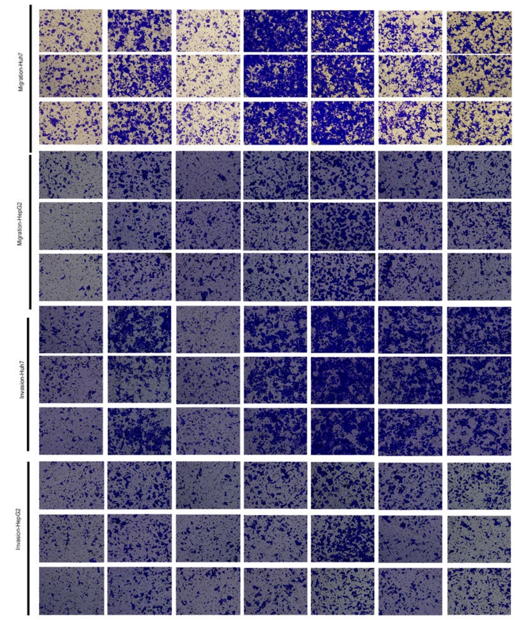

Figure S4D

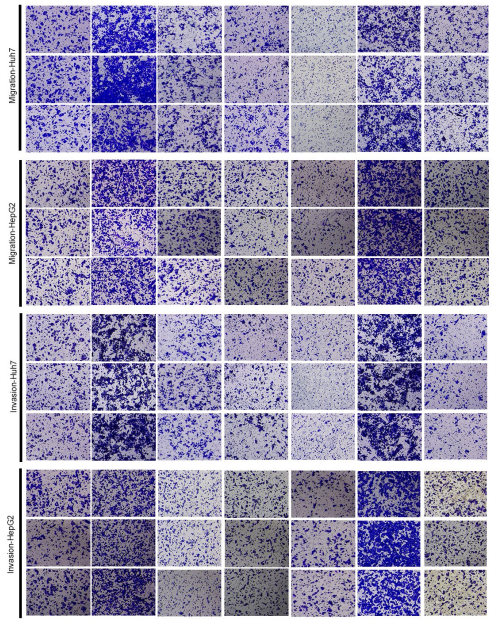

Figure S5A

Edelfosine concentration（μM）	Cell viability (%)		
160	99.8	99.9	99.7
80	99.5	99.7	99.1
10	30	32	37
2	7	11	15

Figure S5B

Edelfosine concentratio	Cell viability (%)		
160	99.2	98.5	98.9
80	98.6	98.6	98.5
10	41	41	39
2	5	13	10

Figure S5D

	KCNN3-OE +si-NC+DMSO	KCNN3-OE +si-NC+edelfosine	CTCF-OE +si-NC+DMSO	CTCF-OE +si-NC+edelfosine
Migration-Huh7	1121	772	771	633
	1004	744	834	665
	951	703	821	685
Migration-HepG2	1100	923	742	561
	1056	834	656	580
	1227	930	734	506

	CTCF-S/D-mut +si-NC+DMSO	CTCF-S/D-mut +si-NC+edelfosine	CTCF-S/D-mut+si-SATB1+edelfosine
Migration-Huh7	917	921	572
	949	974	565
	1035	1059	601
Migration-HepG2	1116	1214	834
	1134	1295	880
	1199	1189	800

**Figure S5E**	KCNN3-OE +si-NC+DMSO	KCNN3-OE +si-NC+edelfosine	CTCF-OE +si-NC+DMSO	CTCF-OE +si-NC+edelfosine
Invasion-Huh7	983	642	683	533
	877	622	661	573
	984	682	663	533
Invasion-HepG2	1052	830	578	506
	1041	891	680	493
	1133	954	611	482

	CTCF-S/D-mut +si-NC+DMSO	CTCF-S/D-mut +si-NC+edelfosine	CTCF-S/D-mut +si-SATB1+edelfosine
Invasion-Huh7	853	996	546
	936	975	549
	858	854	621
Invasion-HepG2	1019	1082	629
	1176	950	687
	1379	1040	771

**Figure S5F**	NC	si-KCNN3
Integrated flourescence sognal in nucleus of CTCF	66.154	66.172
	68.761	108.109
	67.24	67.68
	106.138	65.812
	72.426	63.226

**Figure S5G**	CTCF-WT	CTCF-S/D-MUT
Integrated flourescence sognal in nucleus of CTCF	132.577	127.611
	129.701	127.211
	138.33	131.605
	133.813	132.566
	134.134	137.391

Figure S5C

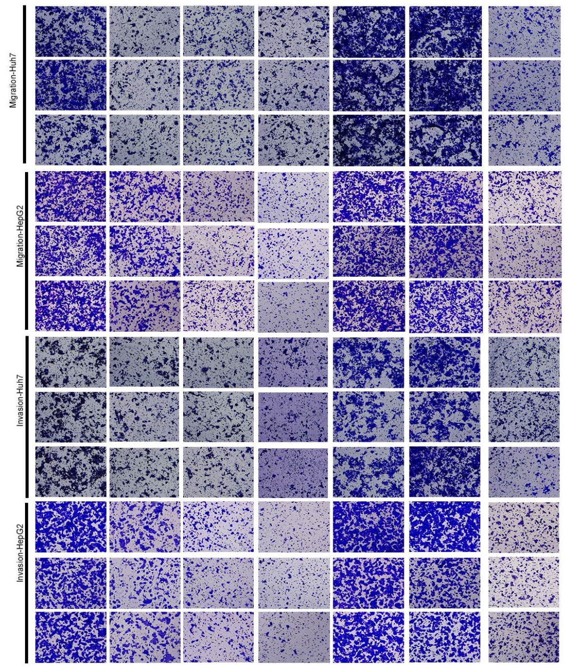

Migration-Huh7
Migration-HepG2
Invasion-Huh7
Invasion-HepG2

Figure S6A	OXA		
160	19	20	21
80	47	32	30
10	55	56	67
2	60	61	60
0.1	89	88	91

Figure S6B	OXA		
160	4.7	2.5	7
80	9.1	8.8	9.7
10	60	51	54
2	58	55	57
0.1	96	100	100

Figure S6D	si-NC			si-KCNN3		
160	18	22	23	2.1	2.2	1.7
80	34	34	32	10	11	6.6
10	54	60	59	23	25	24
2	58	66	61	62	64	61
0.1	87	90	89	88	84	79

Figure S6E	si-NC			si-KCNN3		
160	7	6.7	6	0.8	0.3	0.8
80	9	11	12	11	10	11
10	54	51	55	32	38	32
2	60	60	58	51	40	50
0.1	97	100	100	66	65	68

Figure S6F	si-NC	si-KCNN3
migration	1162	867
	1131	948
	1159	927
invasion	1115	823
	1038	833
	1048	857

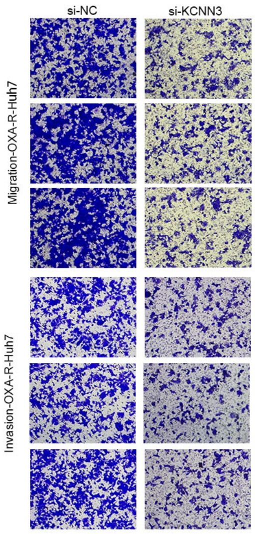

Figure S6G	si-NC	si-KCNN3
migration	1074	759
	1360	587
	887	501
invasion	854	599
	935	512
	948	453

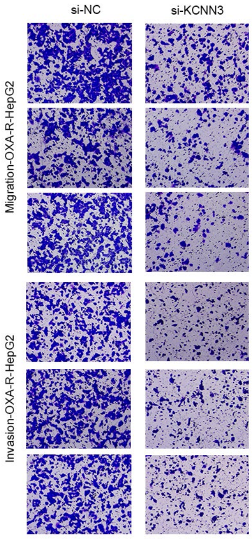

Figure S6H	si-NC	si-KCNN3
Huh7	0.227	0.099
	0.312	0.116
	0.248	0.137

Figure S6I	si-NC	si-KCNN3
HepG2	0.166	0.117
	0.151	0.121
	0.149	0.115

Figure S6J

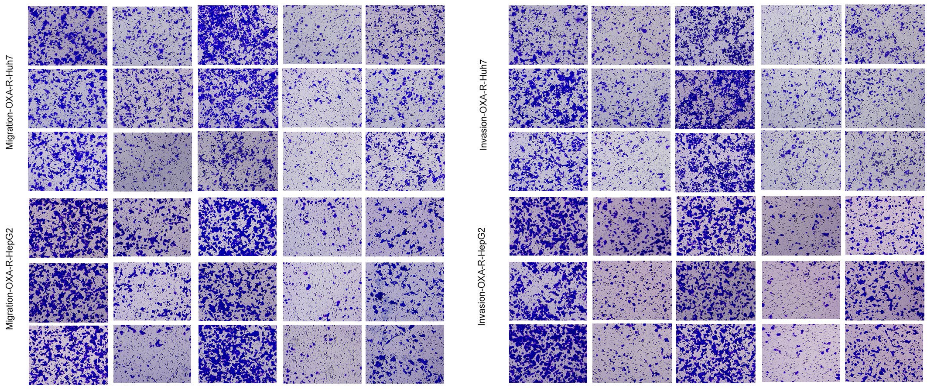

Migration-OXA-R-Huh7
Migration-OXA-R-HepG2
Invasion-OXA-R-Huh7
Invasion-OXA-R-HepG2

**Figure S6K**	OXA-R+EV +DMSO	OXA-R+SPOP-WT +DMSO	OXA-R+SPOP-D153Y +DMSO	OXA-R+SPOP-WT +Edelfosine	OXA-R+SPOP-D153Y +Edelfosine
Migration-OXA-R-Huh7	994	651	1166	499	761
	876	750	1048	502	725
	965	543	1092	423	732
Invasion-OXA-R-Huh7	797	622	988	499	770
	829	606	1064	438	742
	936	539	987	468	734

**Figure S6L**	OXA-R+EV +DMSO	OXA-R+SPOP-WT +DMSO	OXA-R+SPOP-D153Y +DMSO	OXA-R+SPOP-WT +Edelfosine	OXA-R+SPOP-D153Y +Edelfosine
Migration-OXA-R-HepG2	1027	628	937	455	755
	951	695	979	433	758
	970	508	951	440	722
Invasion-OXA-R-HepG2	1008	600	794	477	570
	945	583	853	437	640
	968	543	763	422	626
