# Supplementary material for: SPOP-mediated K27-linked non-degradative ubiquitination of KCNN3 suppressing HCC progression via the CTCF-SATB1 axis
Source: Cell Death Dis. 2026 May 10;17(1):612. doi: 10.1038/s41419-026-08765-3 (PMC13324860; doi:10.1038/s41419-026-08765-3)

Figure2A

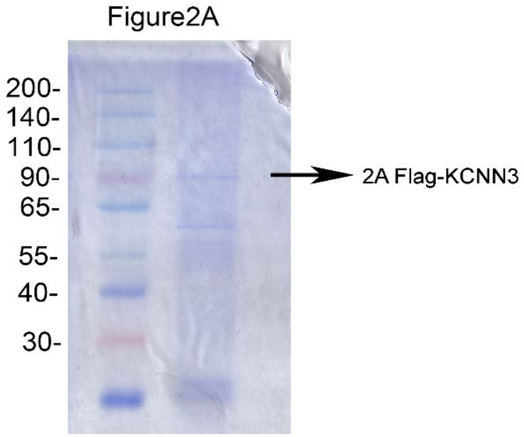

Figure2C

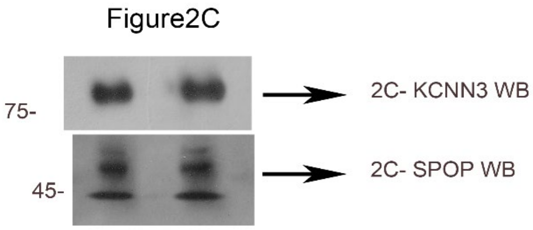

Figure 2F

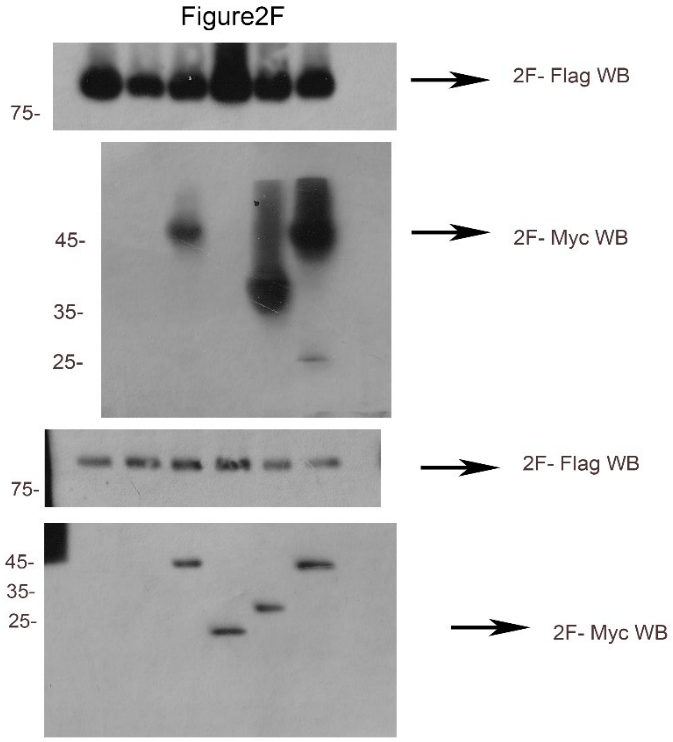

Figure 2B

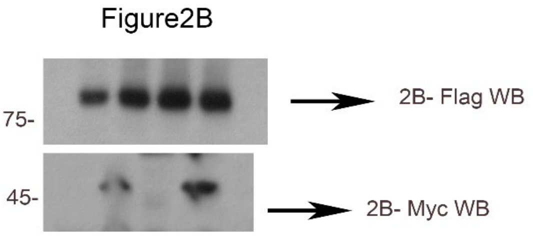

Fig 2D

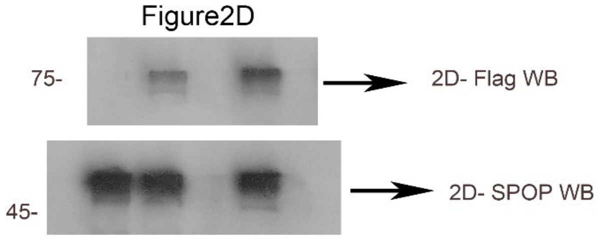

Figure 2H

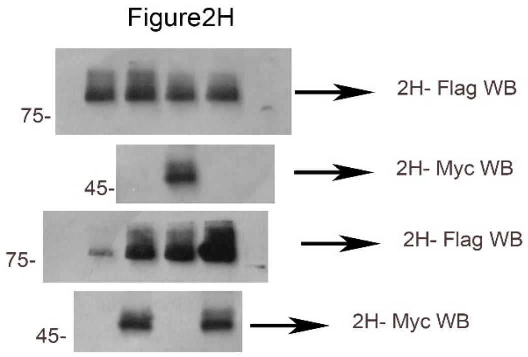

Figure 2J

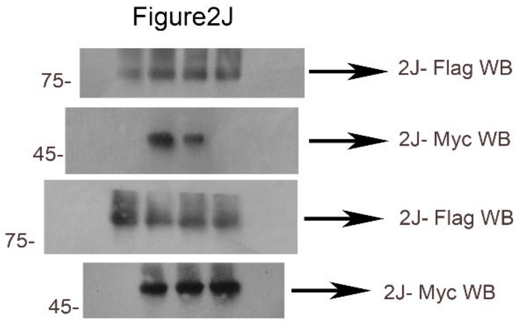

Figure 2I

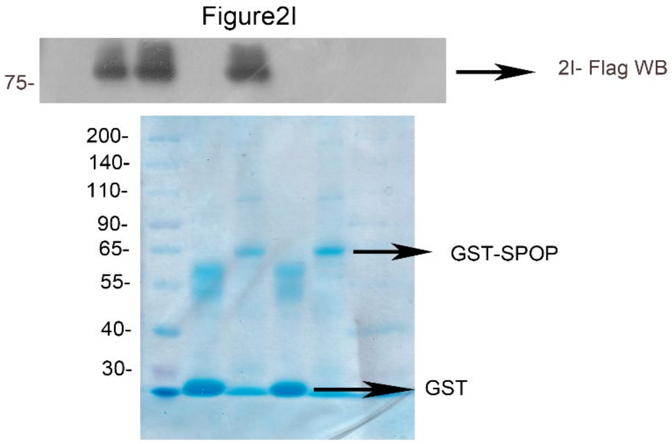

Figure 3A

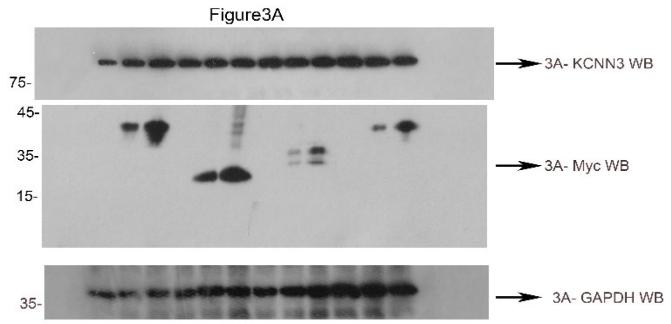

Figure 3B (left）

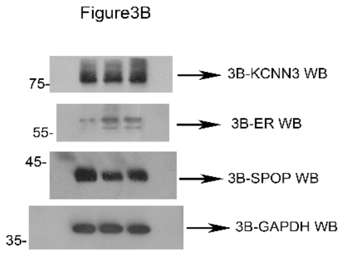

Figure 3E

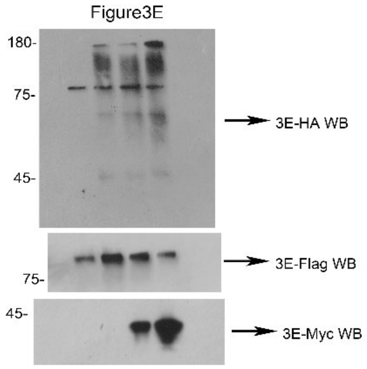

Figure 3F

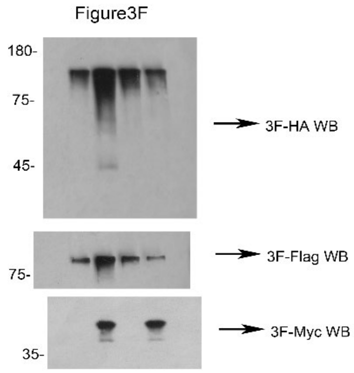

Figure 3J

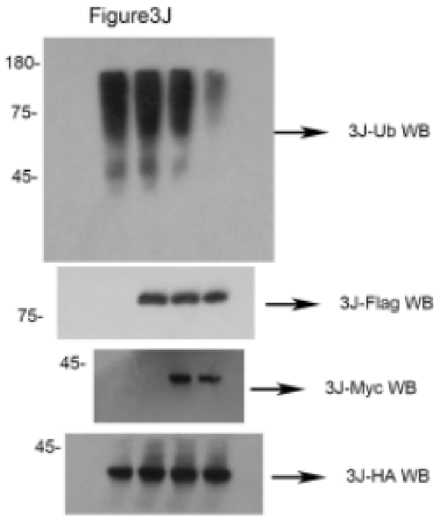

Figure 3L

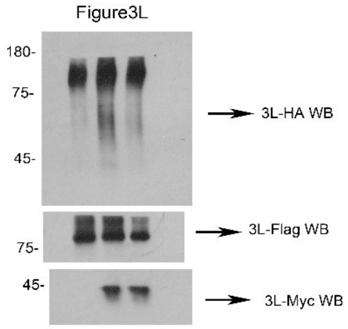

Figure3B (right)

Figure3B

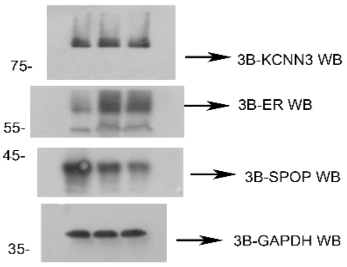

Figure 3C

Figure3C

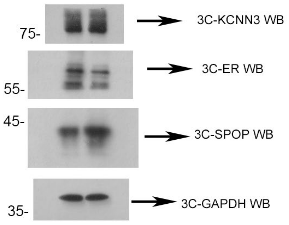

Figure3C

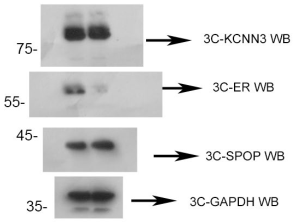

Figure 3G

Figure3G

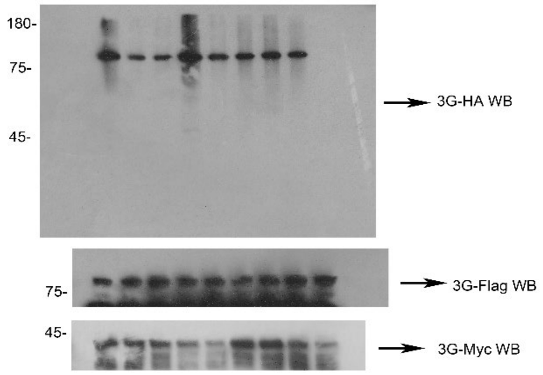

Figure 3D (left)

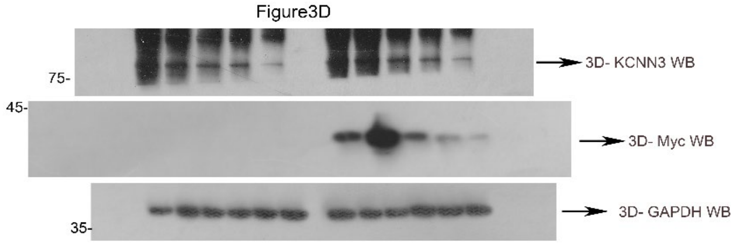

Figure 3H

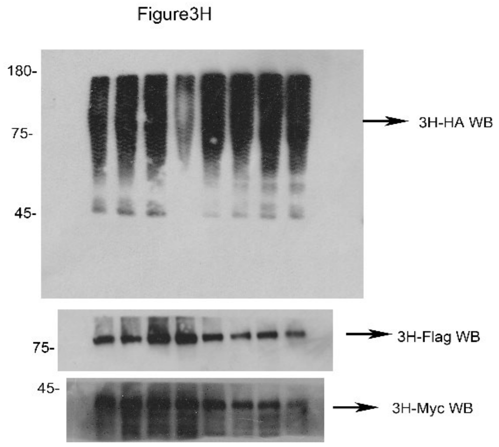

Figure 3I

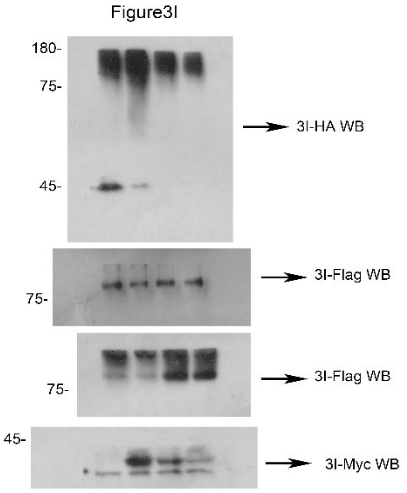

Figure 4G

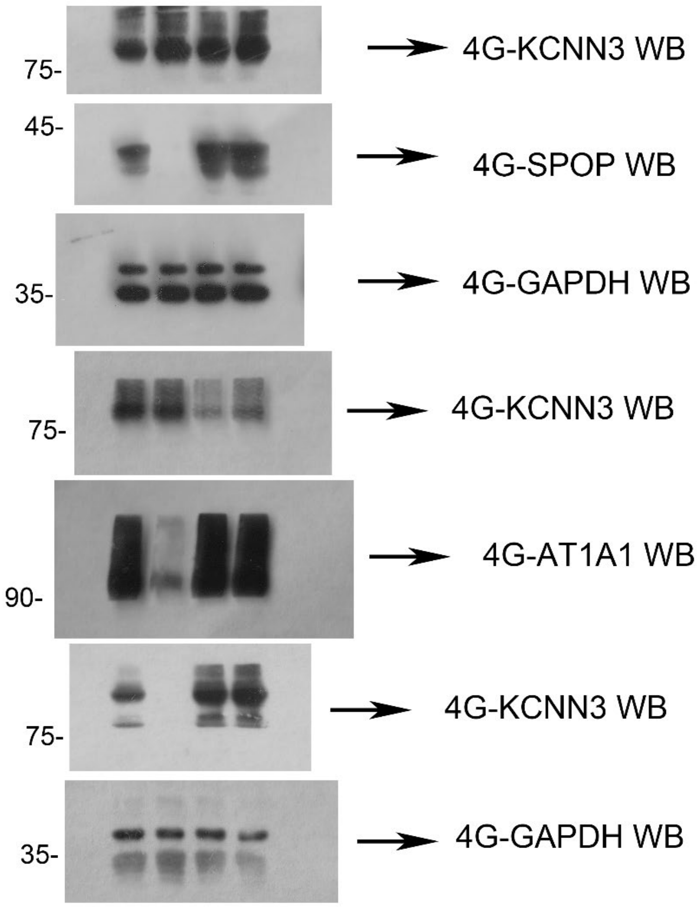
75-
4G-KCNN3 WB
45-
4G-SPOP WB
35-
4G-GAPDH WB
75-
4G-KCNN3 WB
90-
4G-AT1A1 WB
75-
4G-KCNN3 WB
35-
4G-GAPDH WB

Fig 5I

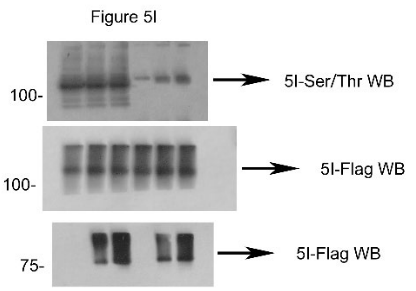
Figure 5I
100-
5I-Ser/Thr WB
100-
5I-Flag WB
75-
5I-Flag WB

Fig 5J

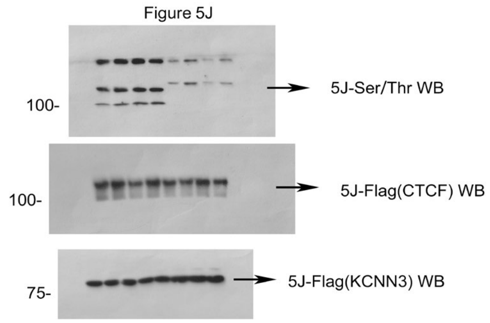
Figure 5J
100-
5J-Ser/Thr WB
100-
5J-Flag(CTCF) WB
75-
5J-Flag(KCNN3) WB

Figure S1C

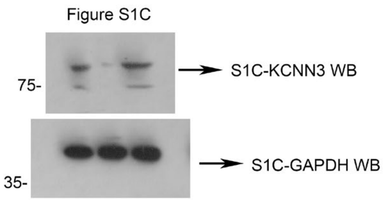

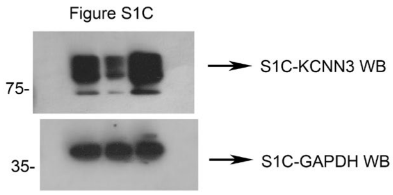

Figure S1F

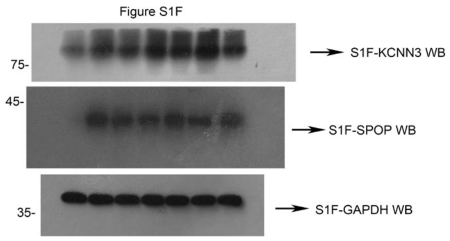

Figure S2D-F

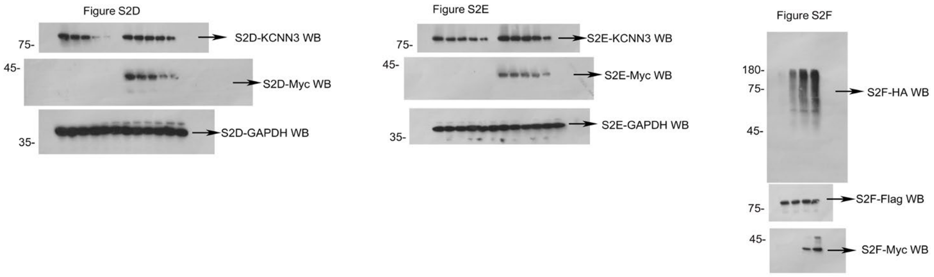

Figure S2G-I

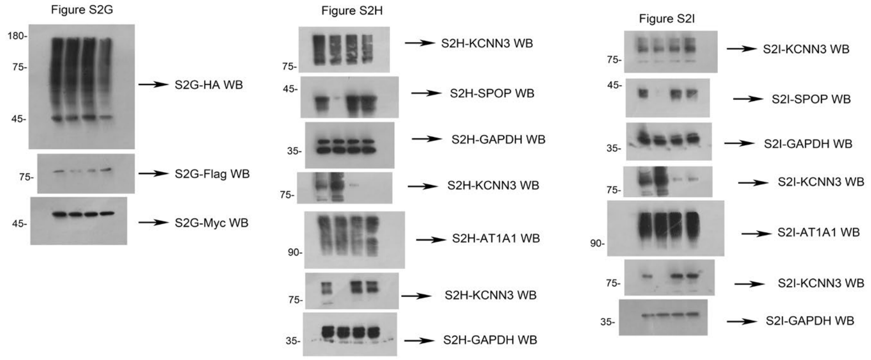

Figure S3N

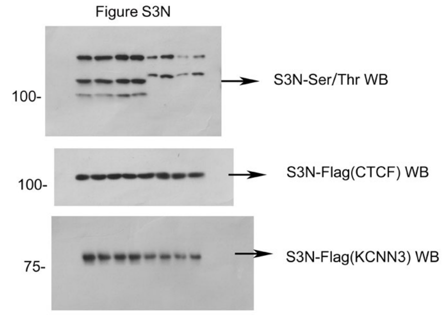

Figure S4C

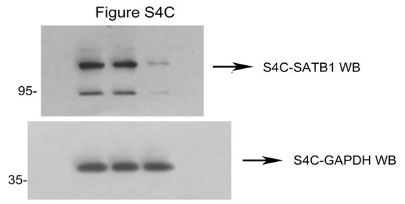

Supplement: Supplementary file 2 — Original Data [file 41419_2026_8765_MOESM2_ESM.pdf]
