# Supplementary material for: SPOP-mediated K27-linked non-degradative ubiquitination of KCNN3 suppressing HCC progression via the CTCF-SATB1 axis
Source: Cell Death Dis. 2026 May 10;17(1):612. doi: 10.1038/s41419-026-08765-3 (PMC13324860; doi:10.1038/s41419-026-08765-3)
Supplement: Supplementary file 3 — Supplementary materials1 [file 41419_2026_8765_MOESM3_ESM.pdf]

Supplementary Information for

SPOP-mediated K27-linked ubiquitination of KCNN3 suppressing hepatocellular carcinoma progression via CTCF-SATB1 axis

This file includes:

Table S1: The RT-qPCR primers, siRNA sequence and construction primers for KOD-Plus Mutagenesis Kit information used in this study

Table S2: Antibody information

Table S3: Chemicals

Supplementary Fig. 1: Bioinformatics analysis of KCNN3 in HCC

Supplementary Fig. 2: The ubiquitination sites of KCNN3 mediated by SPOP

Supplementary Fig. 3: The GO/KEGG enrichment pathway of KCNN3-related genes

Supplementary Fig. 4: HCC-associated SPOP mutations and KCNN3-ΔSBC can compensate for the inhibitory effect of SPOP-WT on KCNN3-induced progression in HCC via CTCF-SATB1 axis

Supplementary Fig. 5: The specific inhibitor of KCNN3, Edelfosine, can effectively inhibit the role of the CTCF-SATB1 signaling axis in the migration and invasion of HCC cells

Supplementary Fig. 6: HCC-associated SPOP mutations can compensate for the inhibitory effect of SPOP-WT on KCNN3-induced progression in OXA-R HCC

Table S1: The RT-qPCR primers, siRNA sequence and construction primers for KOD-Plus Mutagenesis Kit information used in this study

SYBR®Green RT-qPCR primers	
KCNN3	RTF: 5'-CCAACCCCTCCAGCTCTT -3'<br>RTR: 5'-GTTGGCTTTGGGGAAGGT -3'
SATB1	RTF: 5'-GTGGAAGCCTTGGGAATCC -3'<br>RTR: 5'-CTGACAGCTCTTCTTCTAGTT -3'
GAPDH	RTF: 5'-CATGGCCTTCCGTGTTCCTA-3'<br>RTR: 5'-CCCTCAGATGCCTGCTTCA-3'
N-cadherin	RTF: 5'-TCCACCATATGACTCCCTGTTAGT -3'<br>RTR: 5'-CAGAAAACTAATTCCAATCTGAAA -3'
E-cadherin	RTF: 5'-CCTACAATGCTGCCATCGCCTAC -3'<br>RTR: 5'-GGGTAACTCTCTCGGTCCAGTCC -3'
BCAM	RTF: 5'-CCTCGTCGTTGCTGTCTTCT -3'<br>RTR: 5'-TTGCAGATAGCAGGCCACTC -3'
GABBR1	RTF: 5'-CTCAGAAGGTTGCCAGATCATA -3'<br>RTR: 5'-GGCGTTCGATTCACAGATGG -3'
MUC3A	RTF: 5'-GTCTTGGTCCTGAAGCCTGTT -3'<br>RTR: 5'-CACGGGAGTCGAGGTGTAAG -3'
MYO7A	RTF: 5'-GACGTGTGTTTGCAGACTGG -3'<br>RTR: 5'-GTGCATAGGCTTGATGTGCG -3'
CTCF	RTF: 5'-AGGTGACACTATAGAATACAGCAGGAGGGTCTGCTATC -3'<br>RTR: 5'-GTACGACTCACTATAGGGAGTGTGGCTTTTCATGTGACG -3'
c-Myc	RTF:5'-TCAAGAGGCGAACACACAAC -3'<br>RTR:5'-TAACTACCTTGGGGGCCTT -3'
**siRNA oligonucleotide sequences**	
si-SATB1 -1	5'-CCAUUGUGAACAGUACUUA -3'
si-SATB1-2	5'-CUAUCAGUCCUCAAUUGGU -3'
Si-KCNN3	5'-GAAUGUCAUGUAUGACUUA -3'
si-SPOP-1	5'-GGAUGAUGUAAAUGAGCAA-3'
si-SPOP-2	5'-GGGCUUCUCCCUGA UGACAAGCUUA-3'
si-NC	5'-UUCUCCGAACGUGUCACGUTT-3'
**Construction primers for KOD-Plus Mutagenesis Kit**	
K603A-R	5'-CCTCTGGTGTTTCCTCACTTTGGCA -3'
K603A-F	5'-GCGTTCCTCCAAGCTATCCACCAGT -3'
K633A-R	5'-GGAAAGGTCCACCAGAGTGTTGGCT -3'
K633A-F	5'-GCGATGCAGAATGTCATGTATGACT-3'
K654A-R	5'-CTCCAGGTCTTCGCTCCGGTCATTG -3'
K654A-F	5'-GCGCAGATTGGCAGCCTGGAGTCGA -3'
K654R-F	5'-CGCCAGATTGGCAGCCTGGAGTCGA-3'
SPOP-ΔMATH-F	5'-TCTGTCAACATTTCTGGCCAGAATA-3'
SPOP-ΔMATH-R	5'-GATCTGTGTGTAGCACCAACTCTCA-3'
SPOP-ΔBTB-F	5'-TCCGTGGAGAACGCTGCAGAAATTC-3'

SPOP-ΔBTB-R	5’-CTCAGGAACCTTTACCATGTTCATG-3’
SPOP-ΔNLS-F	5’-CTCGAGTACCCATACGACGTACCTG-3’
SPOP-ΔNLS-R	5’-AGGGCACTGTGCTGAAGCCAGAGAG-3’
SPOP-M35L-F	5’- CTGTGGACCATCAATAACTTTAGCT-3’
SPOP-M35L-R	5’- GTAGGAGAATTTCACTACCTTGATC-3’
SPOP-D153Y-F	5'- TACAAGCTTACCCTCTTCTGCGAGG -3'
SPOP-D153Y-R	5'- ATCAGGGAGAAGCCCGTTGGCCTCA -3'

Table S2: Antibody information

Antibody	Cytokine Species	Cat. No	Source	Application/ Dilutions
Anti-KCNN3	Rabbit	TD13233	Abmart	WB: 1:1000 IHC: 1:50
Anti-SATB1	Rabbit	T55078	Abmart	WB:1 :1000 IHC: 1:100
Anti-SPOP	Rabbit	16750-1AP	Proteintech	WB: 1:5000 IHC: 1:250
Anti-ATP1A1	Rabbit	380790	Zenbio	WB: 1:1000
Anti-Ub	Rabbit	10201-2-AP	Proteintech	WB: 1:1000
Anti-Flag	Rabbit	20543-1-AP	Proteintech	WB: 1:20000 IF: 1:200
Anti-Flag	Mouse	66008-4-Ig	Proteintech	IF: 1:500
Anti-HA	Mouse	66006-2-Ig	Proteintech	IF: 1:400
Anti-Myc	Rabbit	16286-1-AP	Proteintech	IF: 1:200
Anti-ER	Rabbit	21244-1-AP	Proteintech	WB: 1:500
Anti-SPOP	Mouse	68216-1-Ig	Proteintech	WB: 1:2000 IF: 1:200
Anti-GAPDH	Rabbit	AC001	Abclonal	WB: 1:8000
Anti-HA	Rabbit	81290-1-RR	Proteintech	IF: 1:500
Anti-Mouse	Donkey	AS033	Abclonal	WB: 1:8000 IHC: 1:200
Anti-Rabbit	Donkey	AS038	Abclonal	WB: 1:8000 IHC: 1:200
Anti-Rabbit-488	Donkey	AS035	Abclonal	IF: 1:200
Anti-Mouse-647	Goat	AS059	Abclonal	IF: 1:200
Anti-Flag beads	Mouse	M2	Sigma	IP
Phospho-(Ser/Thr) Phe	Rabbit	9631	Cst	WB: 1:1000

Table S3: Chemicals

Name	Cat. No	Source
Edelfosine	HY-108610A	MCE
oxaliplatin	HY-17371	MCE
CHX	S7418	Selleckchem
DAPI	C0065	Solarbio

BAPTA-AM	A1076	Sigma Aldrich

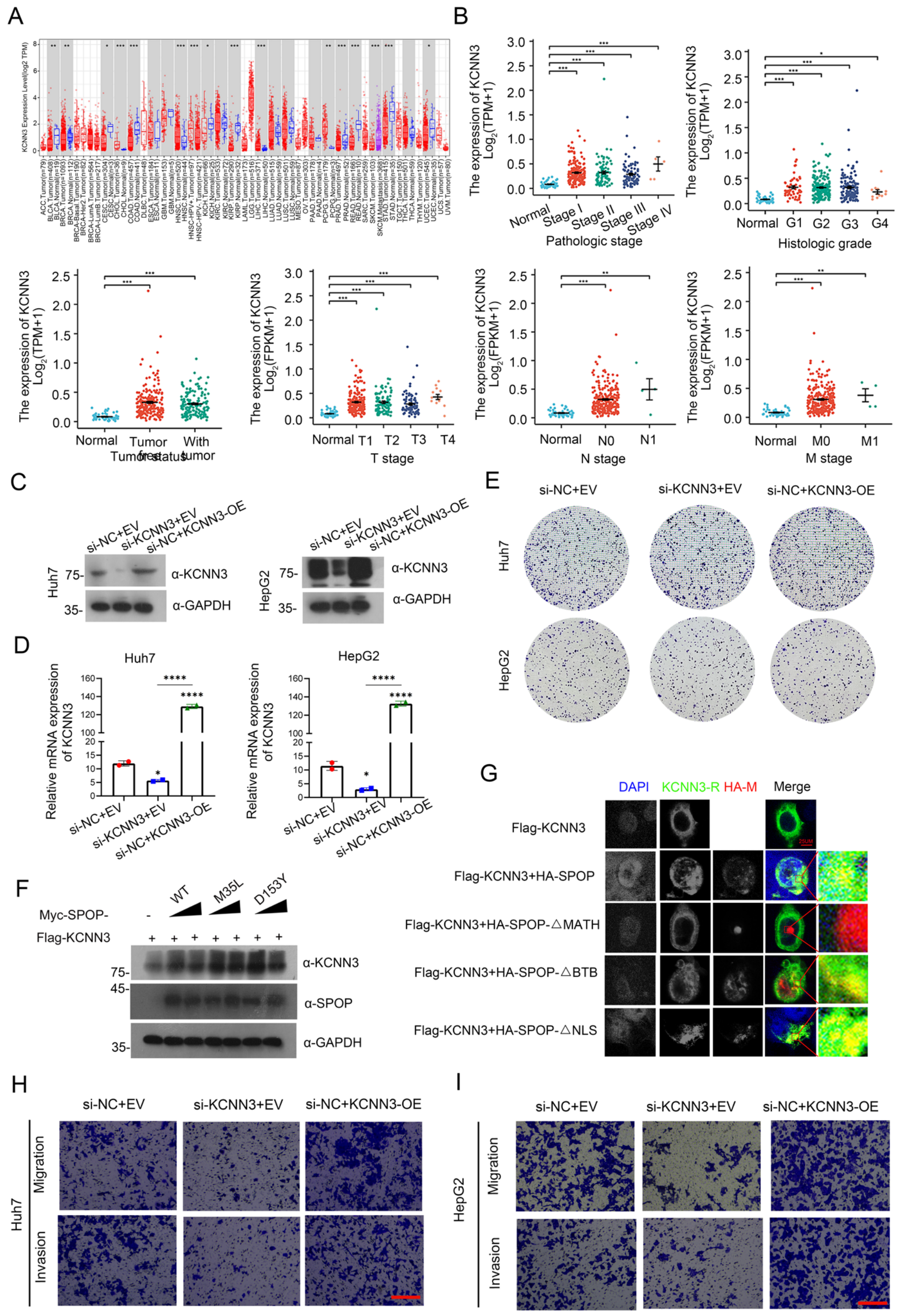

Supplementary Fig. 1 Bioinformatics analysis of KCNN3 in HCC

A. Pan-cancer analysis of KCNN3 in the TCGA database

B. In the TCGA database, KCNN3 was closely related to the clinical characteristics of HCC.

C-D. The protein and mRNA expression effects of KCNN3-OE and si-KCNN3 are verified in Huh7 and HepG2 cells.

E. Representative images of colony formation assays after transfection of si-NC+EV, si-KCNN3+EV

and si-NC+KCNN3-OE in Huh7 and HepG2 cell lines.

F. SPOP-WT, M35L and D153Y did not degrade the protein levels of KCNN3.

G. SPOP-WT, ΔBTB, and ΔNLS were co-localized in the cytoplasm with KCNN3, and the co-localization of SPOP-ΔMATH with KCNN3 was decreased. Scale bar, 25 μm

H. Representative images of transwell (migration and invasion) assays after transfection of si-NC+EV, si-KCNN3+EV and si-NC+KCNN3-OE in Huh7 cell lines. Scale bar, 500 μm.

I. Representative images of transwell (migration and invasion) assays after transfection of si-NC+EV, si-KCNN3+EV and si-NC+KCNN3-OE in HepG2 cell lines. Scale bar, 500 μ

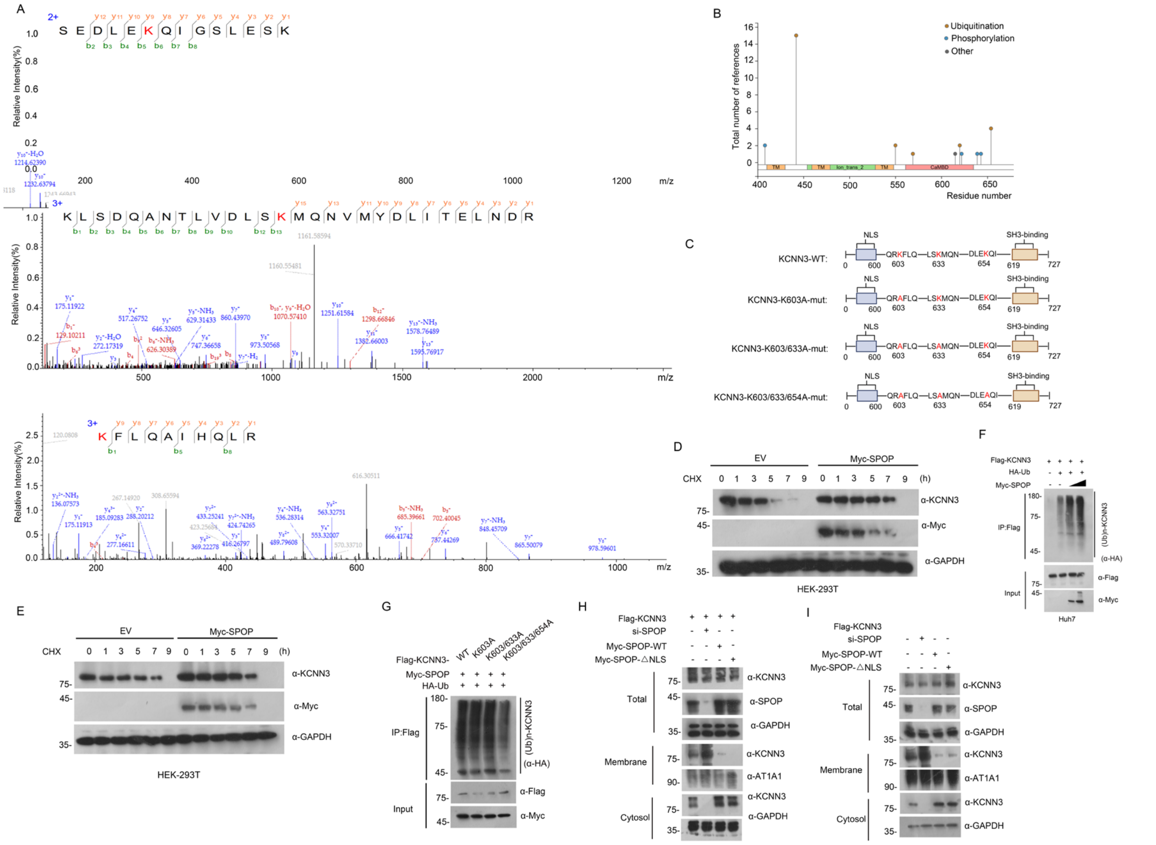

Supplementary Fig. 2 The ubiquitination sites of KCNN3 mediated by SPOP

A. Ubiquitination omics analysis of KCNN3.

B. Prediction of ubiquitination sites of KCNN3 in KCNN3 (human) (phosphosite.org).

C. Structural model diagram of KCNN3-K603A-mut, KCNN3-K603/633A-mut and KCNN3-K603/633/654A-mut.

D-E. HEK-293T cells were transfected with Flag-KCNN3 and/or not co-transfected with Myc-SPOP and obtained WCLs at different time points after treatment with 50μg/ml cycloheximide (CHX) and detected by western blotting.

F. WCLs and CO-IP samples of anti-Flag antibody were obtained from Huh7 cells transfected with Flag-KCNN3, Myc-SPOP and HA-Ub, and detected by western blotting.

G.The ubiquitination levels of KCNN3-WT, KCNN3-K603A, KCNN3-K603/633A and KCNN3-K603/633/654A.

H-I. Two independent plasma membrane isolation assays.

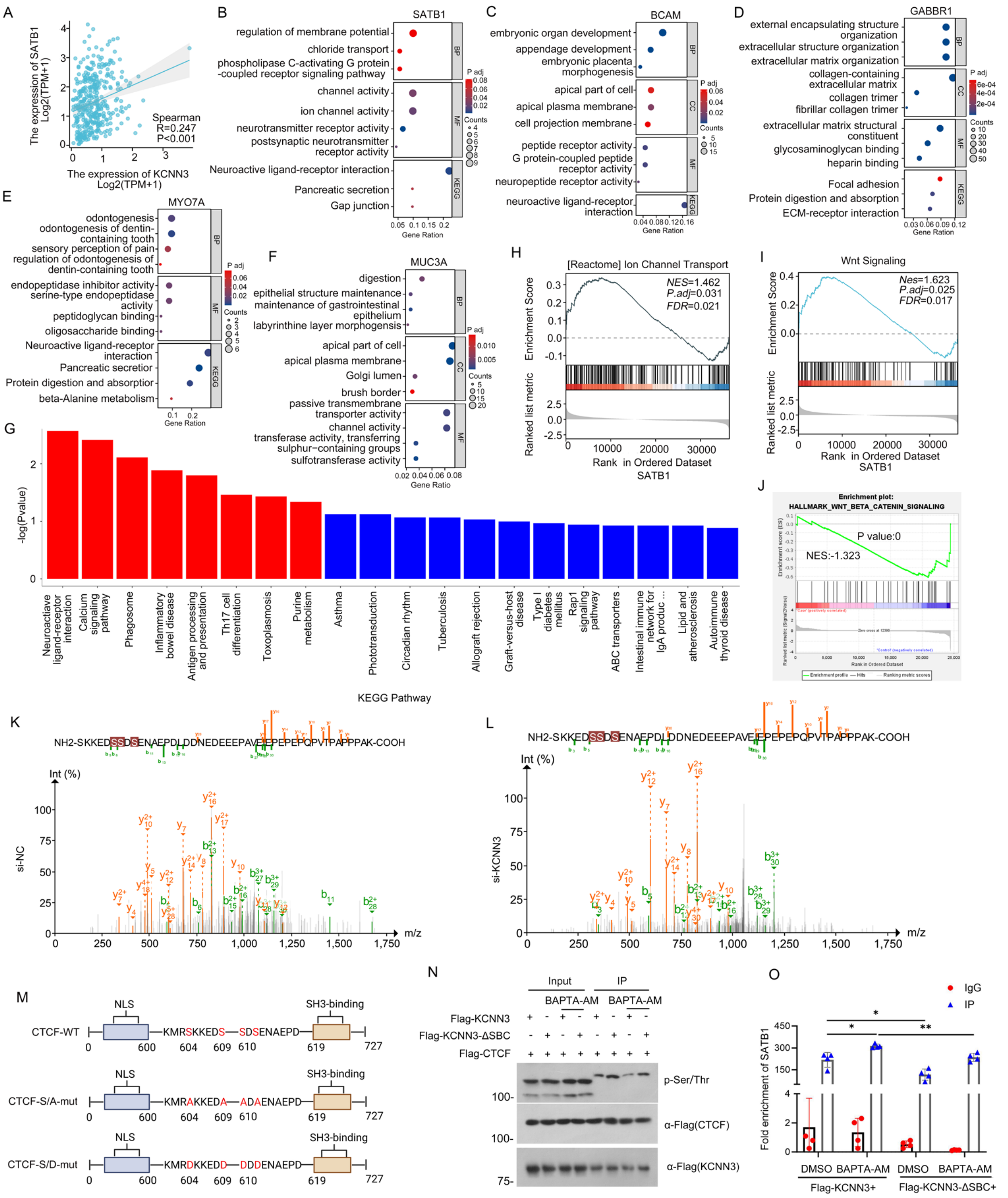

Supplementary Fig. 3 The GO/KEGG enrichment pathway of KCNN3-related genes

A. TCGA database showed that KCNN3 was positively correlated with SATB1.

B-F. The KEGG analysis of SATB1, BCAM, GABBR1, MYO7A and MUC3A.

G. The KEGG analysis of KCNN3.

H-I. The GSEA analysis of SATB1 are enriched to ion channel transport and Wnt signaling pathway.

J. The GSEA analysis of KCNN3 are enriched to Wnt β-catenin signaling pathway.

K-L.The phosphorylation peak chart of proteomic analysis showed that the phosphorylation of Ser 604/609/610/612 in CTCF was altered by KCNN3 knockdown.

M. Structure diagram of CTCF-WT and its mutants (CTCF-S/A-mut and CTCF-S/D-mut).

N.WCLs and CO-IP samples of anti-Flag antibody were obtained from HEK-293T transfected

with Flag-KCNN3, Flag-CTCF, Flag-KCNN3-ΔSBC and/or without dealt with BAPTA-AM and detected by Ser/Thr phosphorylated and α-Flag antibodies by western blotting.

O. RIP-qPCR assays of Huh7 cells transfected with indicated plasmids.

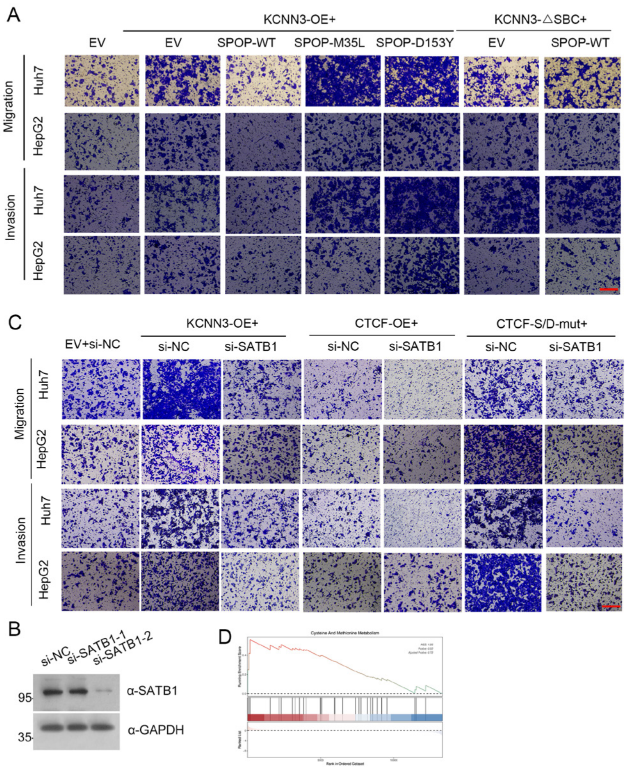

Supplementary Fig. 4 HCC-associated SPOP mutations and KCNN3-ΔSBC can compensate for the inhibitory effect of SPOP-WT on KCNN3-induced progression in HCC via CTCF-SATB1 axis

A. Representative images of transwell (migration and invasion) assays after transfection of indicated plasmids in Huh7 and HepG2 cell lines. Scale bar, 500 μm.

B. Western blotting analysis of SATB1 knockdown.

C. Representative images of transwell (migration and invasion) assays after transfection of indicated plasmids in Huh7 and HepG2 cell lines. Scale bar, 500 μm.

D. The GSEA analysis of KCNN3 are enriched to hsa00270 methionine metabolism (methionine cycle for SAM synthesis) pathways .

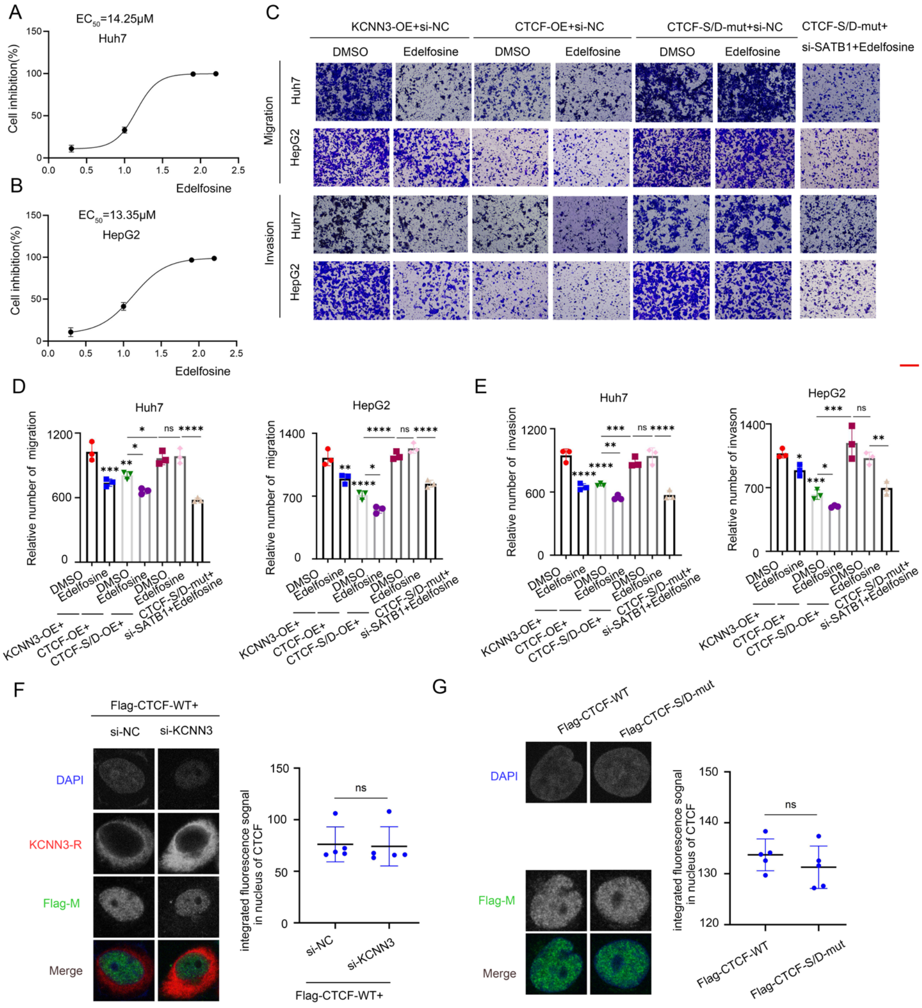

Supplementary Fig. 5 The specific inhibitor of KCNN3, Edelfosine, can effectively inhibit the role of the CTCF-SATB1 signaling axis in the migration and invasion of HCC cells

A. Measurement and analysis of Edelfosine's EC_{50} in Huh7.

B. Measurement and analysis of Edelfosine's EC_{50} in HepG2.

C-E. Migration and invasion experiments have demonstrated that Edelfosine can inhibit the EMT effect of KCNN3 and partially inhibit the EMT effect of CTCF on HCC, but lose the effect on CTCF-S/D-mut. Scale bar, 500 μm.

F. Representative cellular immunofluorescence images of Huh7 cells stained with α-KCNN3-R, α-Flag-M and DAPI after transfection of the indicated plasmids (Left). The statistics of 5 cells in each group, the integrated fluorescence sognal in nucleus of CTCF were analysed (Right). Scale bar, 25 μm

G. Representative cellular immunofluorescence images of Huh7 cells stained with α-Flag-M and DAPI after transfection of the indicated plasmids (Left). The statistics of 5 cells in each group, the integrated fluorescence sognal in nucleus of CTCF were analysed (Right). Scale bar, 25 μm

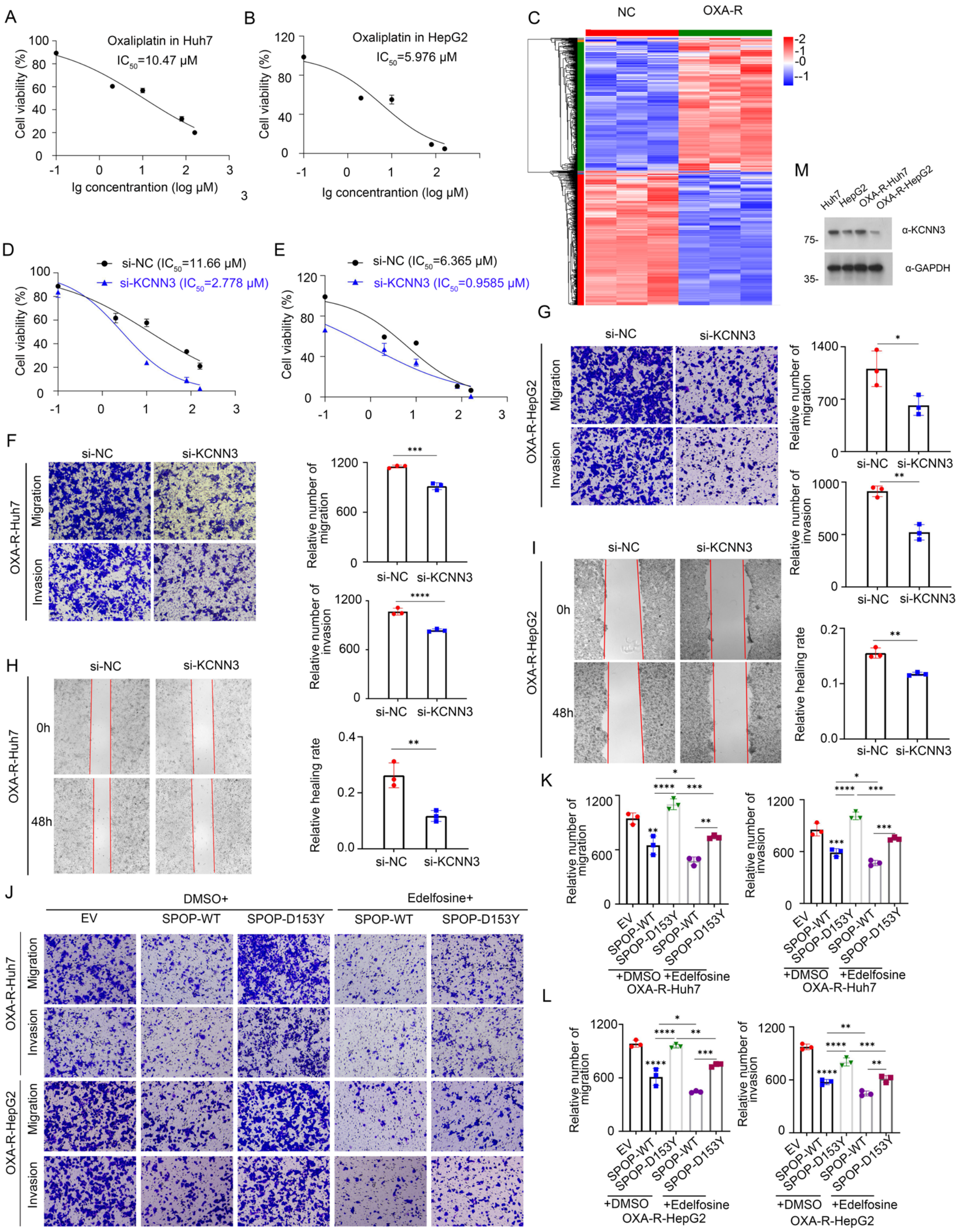

Supplementary Fig. 6 HCC-associated SPOP mutations can compensate for the inhibitory effect of SPOP-WT on KCNN3-induced progression in OXA-R HCC

A. The IC_{50} of oxaliplatin in Huh7 and OXA-R-Huh7.

B. The IC_{50} of oxaliplatin in HepG2 and OXA-R-HepG2.

C. The heatmap of OXA-R HCC RNA-seq.

D. The IC_{50} of oxaliplatin in Huh7 after transfection of si-NC and si-KCNN3.

E. The IC_{50} of oxaliplatin in HepG2 after transfection of si-NC and si-KCNN3.

F. Representative images of transwell (migration and invasion) assays after transfection of si-NC and si-KCNN3 in OXA-R-Huh7 cell lines (Left). Statistical data of migration (Top) and invasion (Bottom) assays in OXA-R-Huh7 cell lines. Scale bar, 500 μm.

G. Representative images of transwell (migration and invasion) assays after transfection of si-NC

and si-KCNN3 in OXA-R-HepG2 cell lines (Left). Statistical data of migration (Top) and invasion (Bottom) assays in OXA-R-HepG2 cell lines. Scale bar, 500 μm.

H. Representative images of the wound healing assay after transfection of si-NC and si-KCNN3 in OXA-R-Huh7 cell lines (Left). Statistical data of the wound healing assay in OXA-R-Huh7 cell lines (Right).

I. Representative images of the wound healing assays after transfection of si-NC and si-KCNN3 in OXA-R-HepG2 cell lines (Left). Statistical data of the wound healing assay in OXA-R-HepG2 cell lines (Right).

J. Representative images of transwell (migration and invasion) assays after transfection of indicated plasmids in OXA-R-Huh7 and OXA-R-HepG2 cell lines. Scale bar, 500 μm.

K-L. Statistical data of transwell (migration and invasion) assays after transfection of indicated plasmids in OXA-R-Huh7 and OXA-R-HepG2 cell lines.

M.The baseline expression of KCNN3 in Huh7 cells, HepG2 cells, OXA-R-Huh7 cells and OXA-R-HepG2 cells detected by western blotting.
